# Inhibition of virally induced TFEB proteasomal degradation as a host-centric therapeutic approach for coronaviral infection

**DOI:** 10.1126/sciadv.adv4033

**Published:** 2025-06-04

**Authors:** Travis B. Lear, Mads B. Larsen, Bo Lin, Benjamin R. Treat, Qing Cao, Áine N. Boudreau, Karina C. Lockwood, Irene Alfaras, Jason R. Kennerdell, Laura Salminen, Daniel P. Camarco, Yao Tong, Jing Ma, Jie Liu, Jay X. Tan, Ferhan Tuncer, John J. Villandre, Lucas Hertzel, Michael M. Myerburg, Yanwen Chen, Claudette St. Croix, Yusuke Sekine, John W. Evankovich, Simon M. Barratt-Boyes, Toren Finkel, Bill B. Chen, Yuan Liu

**Affiliations:** ^1^ Aging Institute, University of Pittsburgh/UPMC, Pittsburgh, PA 15219, USA.; ^2^ Vascular Medicine Institute, University of Pittsburgh, Pittsburgh, PA 15213, USA.; ^3^ Department of Infectious Diseases and Microbiology, School of Public Health, University of Pittsburgh, Pittsburgh, PA 15213, USA.; ^4^ Department of Medicine, Division of Pulmonary, Allergy and Critical Care Medicine, University of Pittsburgh, Pittsburgh, PA 15213, USA.; ^5^ Department of Cell Biology, University of Pittsburgh School of Medicine, Pittsburgh, PA 15219, USA.; ^6^ Center for Biologic Imaging, University of Pittsburgh School of Medicine, University of Pittsburgh, Pittsburgh, PA 15261, USA.; ^7^ Department of Medicine, Division of Cardiology, University of Pittsburgh, Pittsburgh, PA 15213, USA.

## Abstract

The endolysosomal pathway plays an evolutionarily conserved role in pathogen clearance, and viruses have evolved complex mechanisms to evade this host defense system. Here, we describe a previously unidentified aspect of coronaviral infection, whereby the master transcriptional activator of lysosomal homeostasis—TFEB—is targeted for proteasomal-mediated degradation upon viral infection. Through mass spectrometry analysis and an unbiased small interfering RNA screen, we identify that TFEB protein stability is coordinately regulated by the E3 ubiquitin ligase subunit DCAF7 and the PAK2 kinase. We derive a series of novel small molecules that interfere with the DCAF7-TFEB interaction. These agents inhibit virus-induced TFEB degradation and demonstrate broad antiviral activities including attenuating severe acute respiratory syndrome coronavirus 2 infection in two animal models. Together, these results delineate a virally triggered pathway that impairs lysosomal homeostasis in the host. Small molecule E3 ubiquitin ligase DCAF7 inhibitors that restore lysosomal function represent a novel class of host-directed, antiviral therapies useful for current and potentially future coronaviral variants.

## Teaser

Pharmacological prevention of DCAF7-mediated TFEB protein degradation opposes coronaviral infection.

## INTRODUCTION

The beta-coronavirus severe acute respiratory syndrome coronavirus 2 (SARS-CoV-2) (CoV-2) has driven the global COVID-19 pandemic since 2019, which has resulted in over 700 million cases and over 7 million deaths (*1*). CoV-2 is distinct from other circulating human coronaviruses in its virulence and severity, with a subset of patients developing life-threatening acute respiratory distress syndrome (ARDS) (*2*–*4*). Moreover, CoV-2 has demonstrated rapid mutagenicity, resulting in numerously distinct subtypes that have continued to compete and supplant each other as the dominant strain (*5*, *6*). The therapeutic response has been rapid and extensive, resulting in several therapies approved, authorized, or under evaluation for the treatment of COVID-19. Key among these have been direct antiviral agents molnuprivir and nirmatrelvir (Paxlovid) (*7*, *8*). Molnuprivir and nirmatrelvir both impair the proper function of key CoV-2 proteins, helping to blunt viral replication and infectivity. However, these agents suffer severe drawbacks both in off-targeting effects and loss of efficacy among emerging mutant strains (*9*–*12*). Vaccination efforts targeting the CoV-2 surface Spike protein have been extremely effective; however, continued viral evolution has diminished vaccine efficacy against previously unidentified variants, with the persistent concern of a fully vaccine-evasive variant (*13*). Given the substantial drawbacks in viral-directed therapeutic efforts, strategies have arisen that seek to modulate “host factors” to combat infectivity (*14*). CoV-2 infection leads to the direct disruption and hijacking of over 300 human proteins, including key proteins for infection, such as ACE2, and cellular antiviral pathways (*15*). Among these cellular processes affected by CoV-2 infection is the autolysosomal system, a key part of antiviral host defense (*16*).

The autolysosomal system, and the closely related xenophagy, directly responds to microbial threats, including actively eliminating pathogens through the endolysosomal pathway (*17*, *18*). Master transcriptional control of endolysosomal function rests with the transcription factor EB (TFEB), which regulates genes involved in all stages of autophagy (*19*–*21*). Various stressors stimulate the nuclear translocation of TFEB, whereupon it can activate >400 target genes containing the CLEAR (coordinated lysosomal expression and regulation) regulatory motif (*22*). Collectively, these gene products work to increase lysosomal number and function, endocytic activity, lysosomal exocytosis, and maintain lysosomal acidification (*23*). TFEB’s role in host defense is evolutionarily conserved as it is known to play a prominent role in the response to bacterial pathogens in organisms such as *Caenorhabditis elegans* (*24*). In many cases, pathogens have evolved sophisticated mechanisms to disrupt or evade the host’s autophagic machinery (*25*–*27*); intriguingly, a recent study demonstrated that beta-coronaviruses hijack lysosomes as part of their replication cycle (*16*). In an attempt to oppose these viral actions, we hypothesized that enhancing TFEB’s activity may be a means to restore endolysosomal function and improve host immunity.

As a key mediator of lysosomal homeostasis, TFEB activity is tightly regulated through several mechanisms. One key mechanism controlling TFEB’s activity is its subcellular cytoplasmic sequestration, achieved primarily through posttranslational modification and, more specifically, through phosphorylation, which is dependent on cellular nutrient (amino acid, glucose, etc.) status (*28*). Several kinases have been noted to act on TFEB; however, the action of the mechanistic target of rapamycin (mTOR) complex 1 is a primary regulator of TFEB localization. During nutrient-rich conditions, mTORC1 phosphorylates TFEB, preventing TFEB from translocating to the nucleus. This phosphorylation acts through distinct molecular means to exclude TFEB from the nucleus, including obfuscation of a TFEB NLS signal, TFEB’s recruitment to the 14-3-3 cytosolic chaperone complex, and modulation of a nuclear export signal (*22*, *29*–*31*). Upon nutrient scarcity, TFEB phosphorylation is reduced, facilitating its nuclear localization and activation of its catabolic transcriptional program. This is accomplished both by the inactivation of the mTORC1 assembly, reducing its kinase activity, and through lysosomal-dependent calcium release, activating the phosphatase calcineurin to act on TFEB (*32*, *33*). In addition, some studies have noted TFEB can be regulated pretranslation, either through stabilization of its mRNA or by transcriptional activation stimulated by the unfolded protein response pathway (*34*, *35*). Although some have noted that TFEB protein is also susceptible to ubiquitination and its turnover occurs through the proteasome (*36*–*38*), this area has not been well studied.

The ubiquitin-mediated proteasomal degradation apparatus is used in numerous pathways to mediate selective turnover of proteins. Protein ubiquitination occurs via an enzymatic cascade in which the small protein ubiquitin is activated by linkage to an E1 (ubiquitin-activating) enzyme and then transferred to an E2 (ubiquitin-conjugating) enzyme. An E3 ubiquitin ligase then orchestrates the transfer of ubiquitin to a free amine group on the N terminus of a protein substrate or to an internal lysine of the substrate (*39*), thus marking the substrate protein for proteasomal degradation. The concept of a molecular motif termed a “degron” has been observed to function as a signal to dynamically mark a protein for recognition by the ubiquitin proteasome, often through modification such as phosphorylation (*40*). It is estimated that more than 80% of proteins undergo ubiquitin-mediated degradation, so the selection of specific substrates by ubiquitin E3 ligases in response to specific stimuli is a crucial factor in protein stability and cell regulation (*41*). Furthermore, ubiquitin E3 ligases are intricately tied to immunological response to infection and are frequently hijacked by different viruses (*42*, *43*). As proteasomal-dependent protein turnover is recognized as a major cellular mechanism regulating transcription factor activity (*44*–*46*), we questioned whether TFEB turnover was modulated by viral infection.

Here, we report that coronaviral infection triggers TFEB protein degradation through the E3 ligase DCAF7-dependent ubiquitination. A small molecule that blocks virally induced TFEB degradation is shown to have potent antiviral activity.

## RESULTS

### Viruses induce proteasomal-mediated degradation of TFEB

**Fig. 1.**
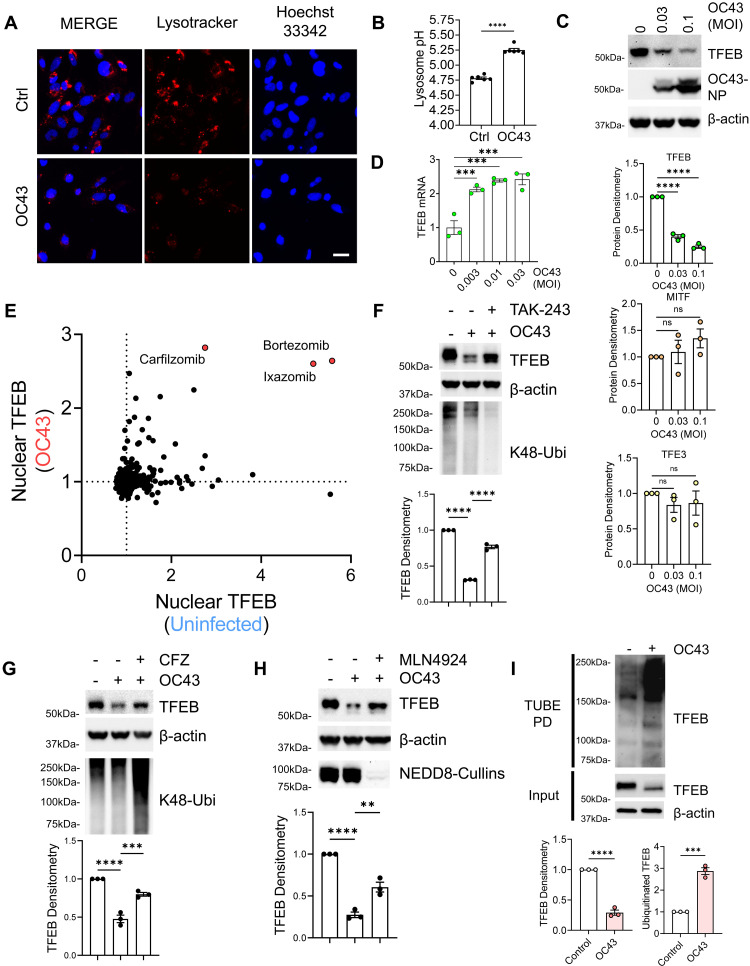
Viral infection triggers TFEB ubiquitin-proteasome degradation. (**A**) Fluorescence imaging of LysoTracker signal from BEAS-2B cells infected with beta-coronavirus OC43, 48 hours. Scale bar, 50 μm. (**B**) Quantification of lysosomal pH (lysosensor yellow/blue) in BEAS-2B cells infected with OC43 for 7 hours. Data represent means ± SEM (*n* = 4). (**C**) Immunoblotting of BEAS-2B cells infected with OC43 (48 hours postinoculation) and probed for key lysosomal transcription factors. TFEB and homolog protein densitometries were quantified; data represent means ± SEM (*n* = 3). (**D**) Quantitative PCR analysis of TFEB mRNA from BEAS-2B cells treated with increasing MOI of OC43 (48 hours). (**E**) FDA-approved compound screening for enhancers of TFEB nuclear localization under uninfected conditions and in the setting of coronavirus (OC43) infection. BEAS-2B cells stably expressing TFEB-EGFP were analyzed following exposure to a library of ~1100 FDA-approved drugs. Log-fold change in nuclear TFEB signal for each compound is shown in two independent screens, one under basal conditions and one following OC43 infection. The top three hits (red dots) identified from both screens are known proteasomal inhibitors. (**F** to **H**) Immunoblotting analysis of TFEB levels from BEAS-2B cells treated with OC43 coronavirus at indicated dose and time with cotreatment of inhibitors of the UPS, TAK-243 (F), CFZ (G), and MLN4924 (H). TFEB densitometry was calculated. Data represent means ± SEM (*n* = 3). (**I**) Immunoblot analysis of polyubiquitinated TFEB signal isolated from TUBE PD eluate from BEAS-2B cells infected with or without OC43 (0.3 MOI, 24 hours). Ubiquitinated TFEB and input TFEB protein densitometries were calculated; data represent means ± SEM (*n* = 3). Not significant (n.s.) *P* > 0.05; **P < 0.01; ***P < 0.001; ****P < 0.0001 as indicated by two-sided unpaired *t* test [(B) and (I)], one-way ANOVA with Dunnett’s multiple comparisons [(C) and (D)], or one-way ANOVA with Tukey’s multiple comparisons [(F) to (H)]. MOI, multiplicity of infection.

To begin to understand how coronaviruses are able to modulate lysosomal function, we inoculated human airway cells with human coronavirus strain OC43, a strain associated with the common cold (*47*). Consistent with previous observations (*16*), we noted a marked disruption of lysosomal homeostasis, including viral-induced alkalization (Fig. 1, A and B). Next, we probed the protein levels of the key transcriptional regulators of lysosomal function and unexpectedly noted a dose- and time-dependent reduction of TFEB protein levels after viral infection, a decline that was not observed among other homologous proteins (MITF, TFE3, TFEC, etc.) (Fig. 1C and fig. S1, A to D). A marked TFEB protein decrease was also observed upon inoculation with the alpha-coronavirus 229E (fig. S1, E and F). This decrease in protein levels occurred without a concomitant reduction in TFEB transcription (Fig. 1D and fig. S1G). These results suggest that viral infection likely triggers the posttranslational degradation of TFEB, which may impair the cell’s ability to maintain lysosomal function.

To explore the biological pathways that might regulate TFEB protein decline in response to viral infection, we performed an unbiased, high-throughput chemical screen using a library of Food and Drug Administration (FDA)–approved molecules (~1100 compounds) to measure nuclear TFEB protein in cells stably expressing an enhanced green fluorescent protein (EGFP)–tagged TFEB chimeric protein. The screen was performed in both uninfected cells, as well as in cells infected with OC43. From these two independent screens, we noted that the top hit compounds, namely, molecules that could maintain nuclear TFEB levels, all represented chemical inhibitors of the proteasome (Fig. 1E). Furthermore, TFEB protein was substantially preserved during viral infection when cotreated with inhibitors of ubiquitin-proteasome system (UPS), including carfilzomib (CFZ; proteasome inhibitor), TAK-243 (ubiquitin E1 inhibitor), and MLN4924 (NEDD8 E1 inhibitor) (Fig. 1, F to H). To further investigate whether ubiquitination is involved in this process to regulate TFEB protein levels, we enriched polyubiquitinated proteins using Tandem Ubiquitin Binding Entities (TUBE) pulldown (PD) and observed a notable increase in endogenous ubiquitinated TFEB protein following viral infection (Fig. 1I). Together, these results suggest that coronaviral infection leads to reduced TFEB protein levels, likely mediated through the ubiquitin-proteasome pathway.

### The E3 ligase DCAF7 mediates TFEB degradation

**Fig. 2.**
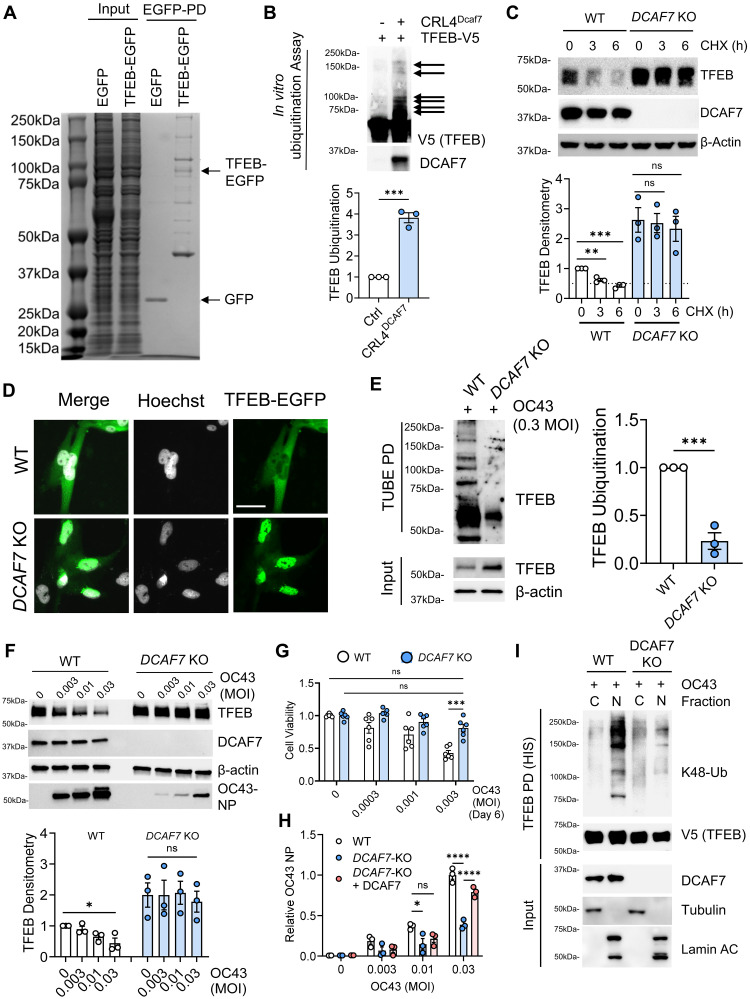
E3 ubiquitin ligase DCAF7 controls TFEB protein stability at baseline and during viral infection. (**A**) Coomassie protein staining of TFEB-EGFP PD prior to mass spectrometry analysis for TFEB-interacting proteins. EGFP only and TFEB-EGFP are indicated. (**B**) In vitro TFEB ubiquitination assay. Briefly, in vitro synthesized TFEB protein was incubated with E1, E2, ubiquitin, and Cul4-DDB1-Rbx1 complex, in the absence or presence of recombinant DCAF7. TFEB ubiquitination was quantified; data represent means ± SEM (*n* = 3). (**C**) Western blot analysis of a CHX chase in WT or *DCAF7* KO BEAS-2B cells. TFEB protein was quantified; data represent means ± SEM (*n* = 4). h, hours. (**D**) Fluorescence microscopy of WT or DCAF7 KO BEAS-2B cells expressing TFEB-EGFP to measure TFEB subcellular localization. Scale bar, 10 μm. (**E**) Immunoblotting of TUBE PD from WT and *DCAF7* KO BEAS-2B cells infected with OC43 (24 hours, 0.3 MOI). TFEB ubiquitination was quantified; data represent means ± SEM (*n* = 3). (**F**) Immunoblot analysis of WT or *DCAF7* KO BEAS-2B cells infected with OC43 for 48 hours. TFEB protein was quantified; data represent means ± SEM (*n* = 3). (**G**) Quantification of cell viability (WT and *DCAF7* KO) at day 6 post-OC43 infection. Data represent means ± SEM (*n* = 6). (**H**) OC43 NP as measured by an in-cell enzyme-linked immunosorbent assay (ELISA) in WT, *DCAF7* KO, or *DCAF7* KO BEAS-2B cells reconstituted with DCAF7 with increasing MOI of OC43. Data were normalized to WT MOI = 0.03 treatment and represent means ± SEM (*n* = 3). (**I**) Immunoblotting of transfected TFEB (V5-HIS-tagged) following PD from the cytosolic and nuclear fractions of WT or DCAF7 KO BEAS-2B. n.s. *P* > 0.05; *P < 0.05; **P < 0.01; ***P < 0.001; ****P < 0.0001 as indicated by two-sided unpaired *t* test [(B) and (E)], one-way ANOVA with Tukey’s multiple comparisons [(C) and (F)], or two-way ANOVA with Tukey’s multiple comparisons [(G) and (H)]. MOI, multiplicity of infection.

As E3 ubiquitin ligases specifically recognize substrates and catalyze their ubiquitination, we therefore sought to identify the E3 ubiquitin ligase regulating TFEB degradation. TFEB-EGFP PD and tandem mass spectrometry (MS/MS) analysis (Dryad DOI: 10.5061/dryad.p2ngf1w2s) detected the protein DCAF7 as the major E3 ubiquitin ligase present in the TFEB-EGFP PD samples (total 449 proteins detected) (Fig. 2A). DCAF7 is a substrate receptor for the Cul4-based CRL ubiquitin E3 ligase complex, and aside from a few studies, the mechanistic function of DCAF7 remains largely unknown (*48*–*50*). Recombinant DCAF7 protein facilitated the Cul4 E3 complex-mediated in vitro polyubiquitination of TFEB (Fig. 2B). In CRISPR-Cas9 *DCAF7* knockout (KO) cells, TFEB exhibited a substantially prolonged protein half-life (Fig. 2C) and increased nuclear localization (Fig. 2D). Ectopic DCAF7 expression decreased TFEB protein levels in a dose-dependent manner, without affecting other related transcription factors (fig. S2A). Furthermore, DCAF7 demonstrated preferential binding to TFEB protein over TFE3 and MITF, suggesting that TFEB is the preferred substrate among MiT family members and related basic helix-loop-helix (bHLH) transcription factors (fig. S2, B and C). These observations are consistent with recent observations that MITF and TFE3 have unique pathways controlling their protein stability, distinct from TFEB protein (*51*). The bioinformatic tool, BDM-PUB, predicted a key ubiquitin acceptor site within TFEB (Lys^232^) (*52*). Mutation of this site increased TFEB protein stability and nuclear localization, provided resistance to viral-induced protein decrease, protected cells from viral-induced cytopathy, and reduced DCAF7-mediated ubiquitination (fig. S3, A to F).

In the absence of viral infection, *DCAF7* KO cells demonstrated an approximately twofold increase in their TFEB protein levels compared to wild-type (WT) control cells (Fig. 2C). This supports a role for DCAF7 in also regulating TFEB levels under basal, noninfected conditions. This observation is also consistent with our chemical screens where we observed increased nuclear TFEB levels in both infected and uninfected cells following the addition of proteasomal inhibitors. During viral infection, *DCAF7* KO cells displayed reduced TFEB polyubiquitination with higher and more stable TFEB protein levels when compared to WT cells (Fig. 2, E and F, and fig. S4, A and B). *DCAF7* KO cells were also resistant to the cytopathic effects of OC43 infection (fig. S4C) and exhibited increased viability postinfection (Fig. 2G and fig. S4D). In *DCAF7* KO cells, the maintenance of TFEB protein also correlated with a substantial decrease in viral load (Fig. 2H and fig. S4, A, B, and E). Consistent with our previous data, TFEB homologs TFE3 and MITF protein levels were insensitive to viral infection or DCAF7 deletion (fig. S4F). The infection of *DCAF7* KO cells also resulted in notably less functional virus, as measured by a viral spreading assay (fig. S5, A to C). To further validate the impacts of DCAF7 on TFEB protein and viral infection, we reexpressed DCAF7 in *DCAF7* KO cells. Reconstitution of DCAF7 in *DCAF7* KO cells abrogated the protective KO phenotype, demonstrating comparable levels of viral infection to those seen in WT cells (Fig. 2H and fig. S6, A and B). Following coronavirus challenge, the DCAF7-TFEB protein-protein interaction increased in the nucleus, as measured by proximity ligation assay (PLA) (fig. S7A). We observed that preservation of TFEB protein in *DCAF7* KO cells is specific to the nuclear compartment, suggesting the nucleus is likely the site of TFEB degradation (fig. S7B). We noted extensive TFEB polyubiquitination in the nuclear compartment that was absent in DCAF7 KO cells (Fig. 2I), and an accumulation of ubiquitination-resistant TFEB mutant protein (K232R) in the nuclear compartment during viral infection (fig. S7C).

### PAK2 primes TFEB for DCAF7-dependent degradation

**Fig. 3.**
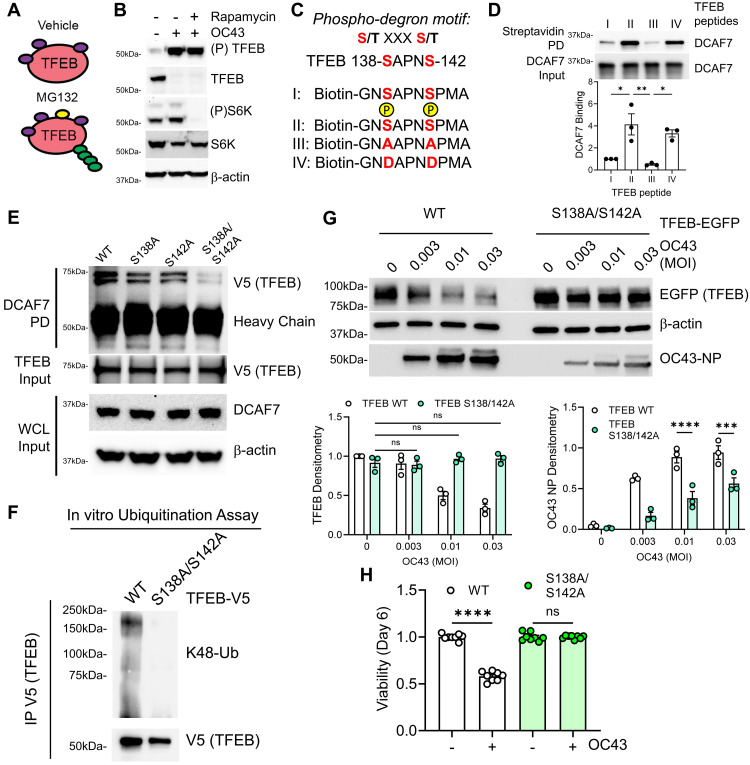
Infection enhances TFEB phosphorylation to serve as a phospho-degron. (**A**) Schematic of TFEB phosphoproteomics experiment. BEAS-2B cells were treated with vehicle or the proteasomal inhibitor MG132 (1 μM) for 4 hours prior to LC-MS/MS analysis of TFEB phosphorylation status to determine putative phosphorylation sites. Candidate sites are listed in table S1. (**B**) Immunoblotting of cells infected with OC43 (0.3 MOI, 72 hours) with or without mTOR inhibition (10 nM rapamycin) and phosphorylated TFEB (Ser^142^, Millipore). (**C**) TFEB sequence corresponding to a known phospho-degron motif (*56*). Sequence of biotin-tagged TFEB peptides corresponding to the putative phosphorylation site. (**D**) TFEB peptide binding assay with recombinant DCAF7. TFEB peptides were conjugated to streptavidin beads and used as bait for DCAF7 protein. DCAF7 densitometry calculated; data represent means ± SEM (*n* = 3). (**E**) Binding assay of TFEB (S-A) mutants with DCAF7. (**F**) In vitro TFEB ubiquitination assay of V5-tagged WT or S138A/S142A TFEB. Briefly, in vitro TnT synthesized TFEB protein was precipitated from the synthesis mixture and incubated with E1, E2, ubiquitin, Cul4-DDB1-Rbx1 complex, and recombinant DCAF7. TFEB ubiquitination was examined with immunoblotting. (**G**) Immunoblot analysis of BEAS-2B cells transfected with either WT TFEB or the double serine phosphomutant TFEB for 18 hours prior to OC43 infection (at indicated MOI for an additional 72 hours). TFEB and OC43 NP densitometry were calculated; data represent means ± SEM (*n* = 3). (**H**) Viability assay of BEAS-2B cells stably expressing TFEB-EGFP WT or phosphomutant (S138A/S142A), following OC43 infection (0.3 MOI, 6 days). Viability determined by CellTiterGlo2.0 measurement; data represent means ± SEM (*n* = 8). n.s. *P* > 0.05; *P < 0.05; **P < 0.01; ***P < 0.001; ****P < 0.0001 as indicated by one-way ANOVA with Tukey’s multiple comparisons [(D) and (H)] and two-way ANOVA with Tukey’s multiple comparisons (G).

The Cullin-based E3 ligase complex typically targets phosphorylated substrates (*53*–*55*), and TFEB nuclear translocation is known to be regulated by phosphorylation status (*23*). We therefore examined whether TFEB phosphorylation could generate a phospho-degron for subsequent ubiquitin-proteasomal degradation. Through phosphoproteomics, we identified several TFEB phosphorylation sites substantially enriched with proteasomal inhibition (MG132), making them candidate phospho-degrons (Fig. 3A and table S1). Of these candidate sites, serine-138 and serine-142 fit the pattern of a classic E3 ligase phospho-degron motif: S/T XXX S/T (*56*). These sites have been described as targets by the mTOR pathway (*31*), a known regulator of TFEB phosphorylation (*30*, *31*, *57*).

We hypothesized that viral infection might alter the phosphorylation of TFEB, marking the protein for ubiquitination. Following viral infection, we observed a notable increase in phosphorylated TFEB, along with our previously described drop in total TFEB protein levels (fig. S8A). Intriguingly, the increase in phosphorylated TFEB persisted even with effective suppression of mTORC1 signaling. We noted that mTORC1 inhibition, by either rapamycin or Torin1 treatment, did not rescue the decline in TFEB protein levels during viral infection nor did these interventions alter TFEB protein levels under baseline conditions (Fig. 3B and fig. S8, B to E). Moreover, phosphorylated TFEB accumulated in the nuclear compartment upon viral infection and persisted even upon mTORC1 inhibition (fig. S8F). Together, these data suggest that, in this specific context, mTOR does not appear to be the relevant kinase underlying virally induced TFEB phosphorylation and degradation.

To further establish phosphorylation of serine-138 and serine-142 on TFEB as a phospho-degron for DCAF7 recognition, we generated phosphodeficient TFEB constructs with double serine to alanine mutations (S138A/S142A). This double mutant, hereafter termed the TFEB phosphomutant, demonstrated markedly reduced phosphorylation following viral infection (fig. S9A) and resulted in reduced DCAF7-TFEB interaction in peptide and protein binding assays (Fig. 3, C to E). In addition, the double TFEB phosphomutant further weakened this interaction when compared to mutation of each residue alone and was resistant to DCAF7-mediated ubiquitination (Fig. 3, E and F, and fig. S9B). After expression in cells, we noted that the TFEB phosphomutant was resistant to degradation following OC43 infection and its persistent expression decreased viral load and enhanced cell viability (Fig. 3, G and H). In contrast, mutation to a phosphomimetic residue (Ser->Asp) destabilized TFEB protein levels in a DCAF7-dependent manner (fig. S9C).

**Fig. 4.**
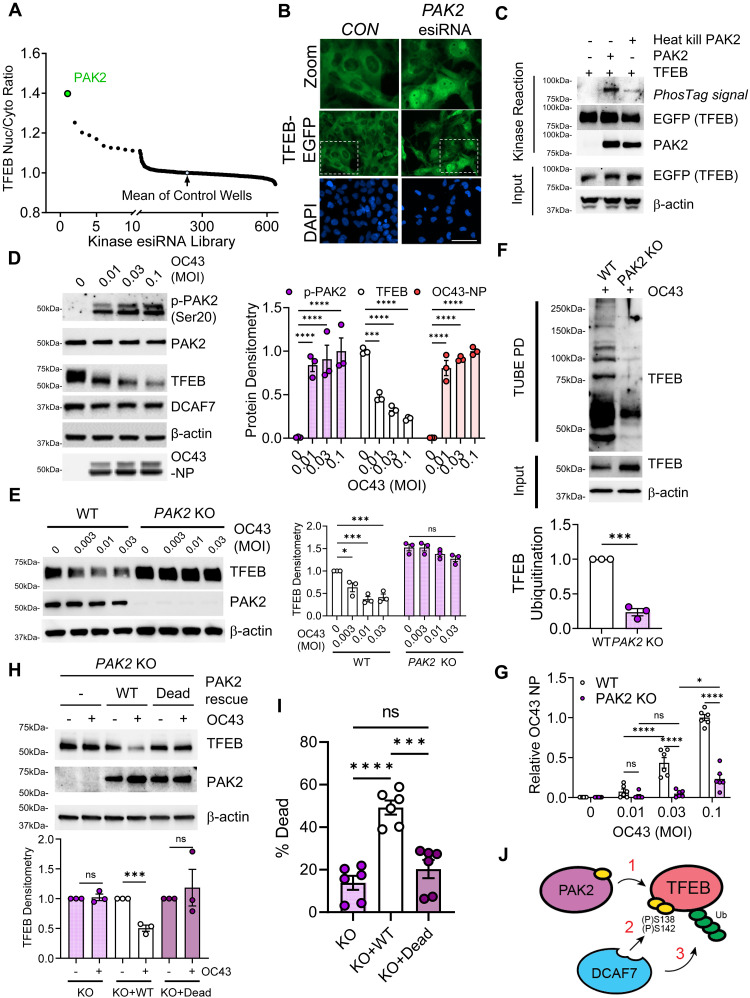
Viral infection activates PAK2 kinase to prime TFEB for degradation. (**A**) High-content imaging screen of kinase regulators of TFEB-EGFP nuclear localization. The kinase PAK2 was identified as a top regulator of TFEB nuclear levels. (**B**) Representative images of TFEB-EGFP localization following control or *PAK2* RNAi treatment. Boxed area is shown at higher magnification (zoom). Scale bar, 50 μm. (**C**) Immunoblot analysis of in vitro kinase assay with PAK2 and TFEB. Phosphosignal detected by the Phos-tag Biotin Probe (Fujifilm) chemical system (*95*). (**D**) Immunoblotting demonstrating PAK2 activation (serine-20 phosphorylation) in BEAS-2B cells following OC43 infection (48 hours). Protein levels were quantified; data represent means ± SEM (*n* = 3). (**E**) Immunoblotting of TFEB in WT or *PAK2* KO BEAS-2B cells following infection with OC43 (48 hours). TFEB was quantified; data represent means ± SEM (*n* = 3). (**F**) TUBE PD assay from WT or *PAK2* KO BEAS-2B cells following 24 hours of OC43 infection (0.3 MOI). Ubiquitinated TFEB was quantified; data represent means ± SEM (*n* = 3). (**G**) Quantification of infection through an in-cell ELISA assay of WT or *PAK2* KO BEAS-2B cells (OC43, 48 hours). Data represent means ± SEM (*n* = 6). (**H**) Immunoblotting of *PAK2* KO cells reconstituted with WT or kinase “dead” [T402A (*101*)] PAK2 followed by OC43 infection (72 hours, 0.3 MOI). TFEB was quantified; data represent means ± SEM (*n* = 3). (**I**) Viability assays of *PAK2* KO cells reconstituted with WT or inactive PAK2 enzyme and infected with OC43 (6 days, 0.3 MOI). Viability represented as percent decrease relative to control. Data represent means ± SEM (*n* = 6). (**J**) Schematic of PAK2-induced priming phosphorylation (step 1), required for DCAF7-mediated binding (step 2), and TFEB polyubiquitination (step 3). n.s. *P* > 0.05; *P < 0.05; ***P < 0.001; ****P < 0.0001 as indicated by one-way ANOVA with Tukey’s post hoc [(E), (G), (H)], two-way ANOVA with Tukey’s post hoc (D), or two-sided unpaired *t* test (F).

Because our data did not support a role for mTOR in priming TFEB for protein degradation, we next sought to identify the relevant kinase by using a custom small interfering RNA (siRNA) library targeting all 600 kinases and high-content imaging to measure nuclear TFEB protein abundance. This unbiased approach uncovered p21-activated kinase 2 (PAK2) as a key determinant of TFEB localization (Fig. 4, A and B, and fig. S10A). PAK2 was sufficient to catalyze the phosphorylation of TFEB in an in vitro kinase assay (Fig. 4C). OC43 coronavirus infection markedly increased PAK2 activation (phospho-serine-20), which correlated with a decline in TFEB levels (Fig. 4D). We generated *PAK2* KO cells and noted an increase in TFEB protein levels at baseline conditions. Furthermore, *PAK2* KO notably mitigated the virally induced decline in TFEB protein, and endogenous TFEB ubiquitination was substantially decreased in *PAK2* KO cells during viral infection (Fig. 4, E and F). Similarly, following coronavirus infection, *PAK2* KO cells displayed reduced viral load and improved overall cell viability (Fig. 4G and fig. S10B). Furthermore, similar to *DCAF7* KO cells, TFEB preservation in *PAK2* KO cells was confined to the nucleus, further supporting a model of nuclear TFEB degradation (fig. S10C). Through protein binding assays, we noted that PAK2 and TFEB could directly interact, and we defined a critical region within TFEB’s bHLH domain required for PAK2 binding (fig. S10, D to G). Last, the protective effects of *PAK2* ablation were lost following WT PAK2 reconstitution, whereas a kinase dead mutant of PAK2 was ineffective (Fig. 4, H and I). Similar to *DCAF7* KO cells, we noted that the protein levels of TFEB homologs TFE3 and MITF were insensitive to viral infection and unaffected by *PAK2* KO (fig. S11, A and B). Together, this suggests PAK2-mediated TFEB phosphorylation generates a critical molecular motif for substrate engagement by the DCAF7-E3 ligase complex for subsequent ubiquitination and proteasomal degradation (Fig. 4J).

### Small molecule DCAF7 inhibitors modulate in vitro and in vivo TFEB activity

From these data, we hypothesized that small molecule inhibition of PAK2 or DCAF7 could prevent TFEB protein degradation, leading to preserved endolysosomal activity and the potential to improve the host’s antiviral response. To date, it has been difficult to generate specific PAK2 inhibitors without notable cytotoxicity (*58*, *59*). As such, we focused on novel small molecules targeting the E3 ubiquitin ligase DCAF7. DCAF7 harbors a conserved WD repeat domain within its C terminus which is a key substrate binding domain (*60*, *61*). We hypothesized that a small molecule that bound to this WD repeat domain could disrupt DCAF7’s interaction with TFEB. We constructed a DCAF7 homology model using the Nurf55 WD domain crystal structure (2XYI.pdb) (*62*) and screened 3 million compounds (ChemDiv Inc.) as potential ligands in silico (fig. S12A). The top score-ranking molecules were selected and further evaluated in cellular assays.

**Fig. 5.**
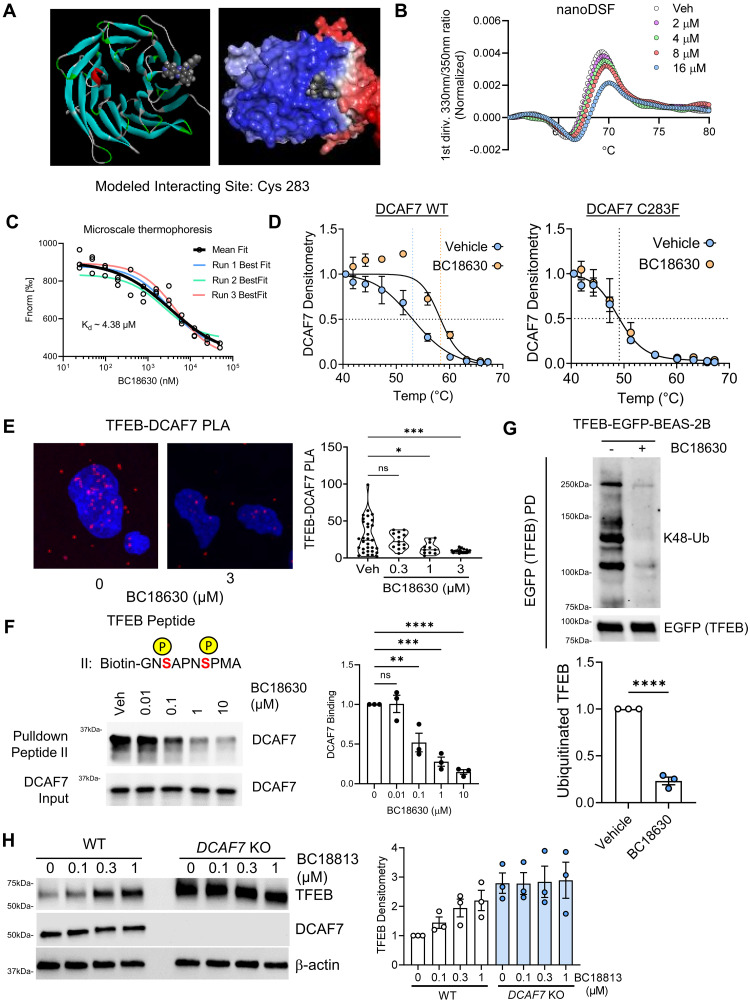
Small molecule DCAF7 inhibitors disrupt DCAF7-TFEB binding and activate TFEB protein level. (**A**) Representation of BC18630 structure docking with the original DCAF7 homology model. BC18630 is predicted to interact with DCAF7 between the β strands of the WD propeller domain. (**B**) nanoDSF of DCAF7 and BC18630. The first derivative of the 350-nm/330-nm fluorescent ratio is displayed for each BC18630 dose, showing shift consistent with direct target engagement. (**C**) MST of DCAF7 and BC18630. Fluorescently labeled DCAF7 protein was incubated with BC18630, heated across a temperature gradient, and thermophoretic fluorescent change measured. Normalized fluorescence of heated versus unheated (Fnorm) was determined and plotted against BC18630 concentration to calculate a binding coefficient. (**D**) CETSA assay of endogenous DCAF7, and reconstituted DCAF7 with mutated predicted interacting site (Cys^283^Phe) expressed in cells and incubated BC18630. Immunoblotted DCAF7 protein densitometry was quantified (*n* = 3). (**E**) PLA of TFEB and DCAF7 in BEAS-2B cells treated with BC18630. The TFEB-DCAF7 association is indicated by red dots. Data represent mean and interquartile range (*n* = 11 to 25 cells). (**F**) Peptide binding assay with BC18630 competition. Biotin-labeled phosphorylated TFEB peptides (Ser^138^ and Ser^142^) were conjugated to streptavidin beads and incubated with DCAF7 protein that was preincubated with BC18630. After binding and washing, the eluate was analyzed for DCAF7 signal and quantified. Data represent means ± SEM (*n* = 3). (**G**) TFEB cellular ubiquitination assay with BC18630 treatment. TFEB-EGFP protein was immunoprecipitated and ubiquitinated TFEB signal was quantified; data represent means ± SEM (*n* = 3). (**H**) Immunoblot analysis WT or *DCAF7* KO BEAS-2B cells treated with increasing concentrations of BC18813 (18 hours). TFEB was quantified; data represent means ± SEM (*n* = 3). n.s. *P* > 0.05; *P < 0.05; **P < 0.01; ***P < 0.001; ****P < 0.0001, or *P* value noted, as indicated by one-way ANOVA with Dunnett’s post hoc [(E) and (F)] or two-sided *t* test (G).

Our initial top hit, termed BC1753, dose-dependently increased TFEB protein and nuclear localization and increased LC3-marked autophagosome formation (fig. S12, B to D), consistent with our observation that DCAF7 regulates basal TFEB levels (see Fig. 2, C and G). We executed multiple rounds of hit-to-lead and subsequent lead optimization and generated two closely related candidate DCAF7 inhibitors, BC18813 and BC18630 (figs. S12E and S13, A to E). Redocking of BC18630 demonstrated an interaction between the β strands of the propeller domain of DCAF7 (Fig. 5A). This interaction pattern (WD6) is similar to observations in a previous study that identified an allosteric inhibitor of the related ubiquitin ligase SCF(Cdc4) (*63*). Direct target engagement between our compounds and DCAF7 was confirmed through a number of complementary biophysical assays, including ectopic and endogenous thermal shift assay, nano differential scanning fluorimetry (nanoDSF), microscale thermophoresis (MST), and surface plasmon resonance (Fig. 5, B and C, and fig. S14, A to G). Moreover, informed by the predicted compound interacting site from redocking, we generated mutant DCAF7 constructs where a key hypothesized BC18630 binding site was mutated (Cys^283^Phe). This mutant DCAF7 construct was expressed in the background of *DCAF7* KO cells and compound binding was assessed using thermal shift assays. We noted that altering the predicted binding site abrogated the ability of BC18630 to bind to DCAF7 protein, strengthening the biophysical basis of our model (Fig. 5D and fig. S14, E and G). We also confirmed the absence of target engagement for the donor structure protein (i.e., Nurf55) used for the initial modeling (fig. S14, H and I).

To assess the specificity and potential liability of these compounds, we also tested their effects on known protein substrates of other closely related E3 ligases to DCAF7. We noted that our DCAF7 inhibitors selectively increased nuclear TFEB protein levels, without altering other E3 substrates (fig. S15A). In addition, both compounds exhibited minimal cellular toxicity at concentrations below 10 μM (fig. S15, B and C). DCAF7 inhibitors also demonstrated limited off-targeting activity against a wide range of channels, transporters, and kinases, which are known safety liabilities (table S2). Both BC18813 and BC18630 largely reduced the measured nuclear protein-protein interaction between DCAF7 and TFEB, reduced in vitro peptide binding, decreased TFEB ubiquitination, and increased TFEB protein level in the presence of DCAF7 (Fig. 5, E to H, and fig. S16, A and B). We also confirmed that our DCAF7 inhibitors do not nonspecifically affect proteasomal activity (fig. S16C).

**Fig. 6.**
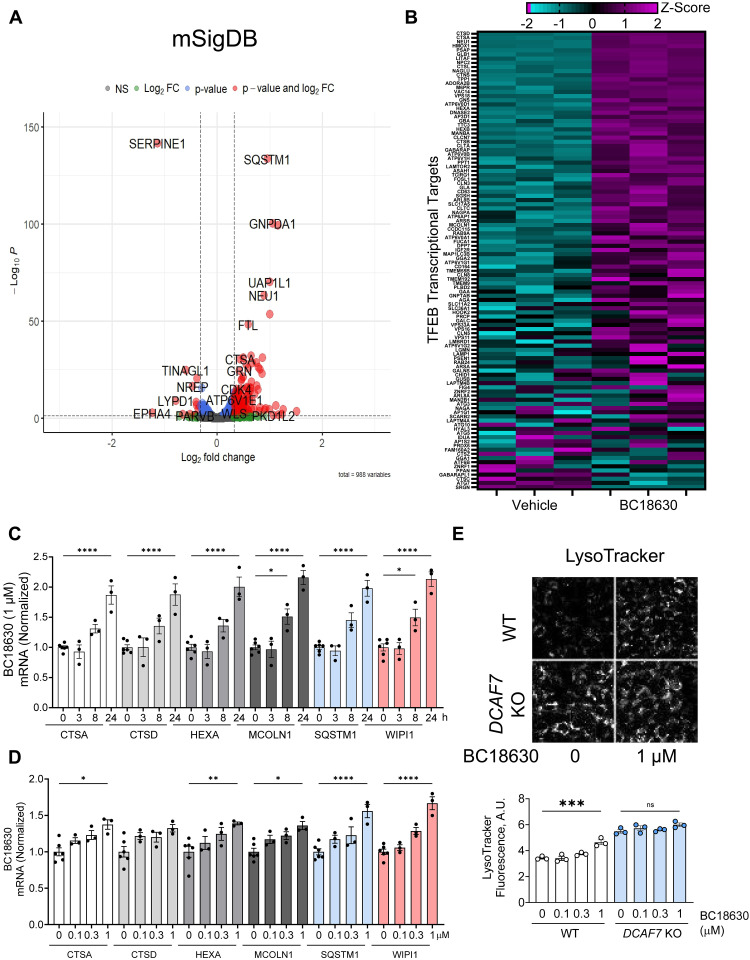
Small molecule DCAF7 inhibition activates TFEB transcriptional program. (**A**) RNA-seq results (GEO accession number: GSE292982) from BEAS-2B cells treated with BC18630 (3 μM) or vehicle for 18 hours. Data are represented as a volcano plot with significance plotted against fold change (*n* = 3 per group). Genes plotted include the mSigDB collection (*102*). (**B**) Heatmap of RNA-seq data from BEAS-2B cells treated with or without BC18630 (3 μM) for 18 hours. Genes shown are previously identified TFEB targets associated with autophagy and lysosomal biogenesis (*103*). Data are *Z* score from *n* = 3 biological replicates. (**C** and **D**) Quantitative PCR analysis of known lysosomal transcriptional targets of TFEB obtained from BEAS-2B cells treated with BC18630 (1 μM) for the indicated time (C) with increasing concentrations (D) and harvested at 18 hours. Data represent fold change in indicated target mRNA levels relative to control treatment; means ± SEM (*n* = 3 to 6). (**E**) Fluorescent micrograph of WT or *DCAF7* KO BEAS-2B cells treated with BC18630 (18 hours) and stained with LysoTracker. Fluorescence was quantified; data represent median LysoTracker signal from each sample, means ± SEM (*n* = 3). n.s. *P* > 0.05; *P < 0.05; **P < 0.01; ***P < 0.001; ****P < 0.0001, or *P* value noted, compared to vehicle or control or as indicated by one-way ANOVA with Dunnett’s multiple comparisons [(C) and (D)] or one-way ANOVA with Tukey’s multiple comparisons (E).

Consistent with the role for DCAF7 in basal TFEB regulation, treatment with our DCAF7 inhibitor substantially increased nuclear levels of TFEB in a time and dose-dependent manner (fig. S17, A to D) in a wide variety of cell types (fig. S18, A and B). To measure the effect of DCAF7 inhibitor on TFEB-targeted transcription, we performed RNA sequencing (RNA-seq) with or without compound treatment (GEO accession number: GSE292982). BC18630 led to a notable increase in transcripts previously identified as TFEB transcriptional targets (*64*) (Fig. 6, A and B, and fig. S19, A to C), including multiple key components of the autophagy-lysosomal pathway (lysosomal proteases, autophagic transport, autophagy receptors) (fig. S20, A and B). DCAF7 inhibitor treatment did not alter the transcript levels of TFEB, DCAF7, or PAK2 (fig. S20C). We validated the effect of DCAF7 inhibition on specific TFEB targets and measured a notable dose- and time-dependent increase in key TFEB-transcriptional targets (Fig. 6, C and D, and fig. S21, A and B). Particularly, we noted that DCAF7 inhibitor treatment increased lysosomal number (LysoTracker) and activity (Magic Red) in a DCAF7-dependent manner as *DCAF7* KO cells were insensitive to these agents (Fig. 6E and fig. S22, A to C). Thus, these small molecule inhibitors of DCAF7 can function to increase basal TFEB levels and lysosomal activity.

### DCAF7 inhibitors attenuate a wide range of viral infections including SARS-CoV-2 infection

**Fig. 7.**
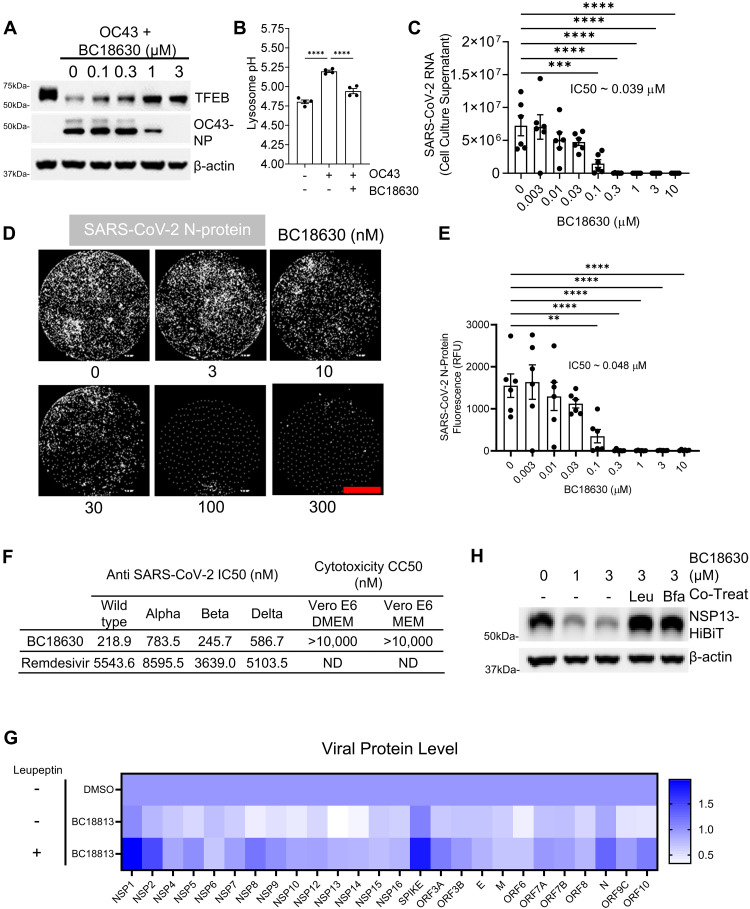
In vitro protection from coronaviral infection using small molecule DCAF7 inhibitors. (**A**) Immunoblot analysis of OC43 (0.3 MOI, 48 hours) infected BEAS-2B cells treated with increasing concentrations of BC18630. (**B**) Quantification of lysosomal pH in BEAS-2B cells infected with OC43 for 7 hours without or with 18 hours pretreatment of BC18630 (100 nM). Data represent means ± SEM (*n* = 4). (**C** to **E**) Cell-based SARS-CoV-2 infection assay with Calu-3 human lung cells, DCAF7 inhibitors, and SARS-CoV-2 (USA-WA-1/2020, MOI 0.01). Viral RNA was detected from cell supernatant (C). Data represent means ± SEM (*n* = 6); IC_50_ determined by sigmoidal nonlinear regression. Cells were fixed and stained for SARS-CoV-2 NP for fluorescence microscopy and quantification of viral signal [(D) and (E)]. Data represent means ± SEM (*n* = 6), and IC_50_ values were determined by sigmoidal nonlinear regression. (**F**) Vero E6 kidney cells were infected with SARS-CoV-2 WT and WHO previously annotated VOCs Alpha, Beta, and Delta along with BC18630 or remdesivir. Infection was quantified, data are IC_50_ estimates, *n* = 4, calculated from nonlinear regression with variable Hill slope. Cytotoxicity (CC_50_) of DCAF7 inhibitor was estimated to be larger than 10 μM, the highest concentration tested. ND, not determined. (**G**) Codon-optimized SARS-CoV-2 viral protein sequences (*65*) were cloned into HiBiT-tagged expression vectors, expressed in BEAS-2B, and their stability landscape was profiled. Viral proteins expressed in BEAS-2B for 18 hours, and then treated with BC18813 (3 μM, 24 hours) with lysosomal inhibition (leupeptin, 2 μM). Data are mean values of *n* = 2 to 4 biological replicates. (**H**) Immunoblot analysis of BEAS-2B cells transfected with NSP13-HiBiT and treated with DCAF7 inhibitor (24 hours) and lysosomal inhibitor leupeptin (Leu, 2 μM) or BFA (0.2 μM). **P < 0.01; ***P < 0.001; ****P < 0.0001 as indicated by one-way ANOVA with Tukey’s post hoc (B) or by one-way ANOVA with Dunnett’s post hoc [(C) and (E)]. Scale bar, 2000 μm.

As our DCAF7 inhibitors demonstrated the capacity to modulate basal TFEB levels and lysosomal activity, we next sought to explore the utility of these compounds in the setting of viral infection, where the DCAF7-dependent degradation of TFEB is markedly augmented. We noted that our small molecule DCAF7 inhibitors effectively prevented virally triggered TFEB degradation and dose-dependently reduced OC43 coronavirus infection (Fig. 7A and fig. S23, A and B) while increasing TFEB-targeted transcripts (fig. S23C). Compound-treated cells also yielded less virus spreading in a dose-dependent manner (fig. S23, D to F). Small molecule BC18813 protection of TFEB protein levels was confined to the nuclear compartment, consistent with our previous observations in *DCAF7* KO cells (fig. S24, A to C). Consistent with previous observations (*16*), coronavirus infection led to a substantial increase in lysosomal pH; however, BC18630 inhibited this virally induced alkalization (Fig. 7B). These inhibitors reduced HCoV-OC43 nucleoprotein (NP) levels at an estimated median inhibitory concentration (IC_50_) of 0.3 μM (fig. S25, A and B). Furthermore, both DCAF7 inhibitors strongly decreased OC43 positive cells (fig. S25, C and D). DCAF7 inhibitors also protected host cells against 229E-induced infection and cytopathic effects (fig. S25, E to I).

Given the apparent efficacy of inhibiting DCAF7 to modulate the response to coronaviruses that trigger the common cold, we next assessed the effect of modulating TFEB in the setting of live SARS-CoV-2 infection. We used BSL3 facilities (IIT Research Institute, Chicago) to test DCAF7 inhibitors in SARS-CoV-2 infection assays. Both compounds largely reduced SARS-CoV-2 viral RNA load and cellular infection as measured by SARS-CoV-2 NP immunofluorescence (Fig. 7, C to E, and fig. S26, A to C). Furthermore, these host-centric strategies demonstrated efficacy against numerous SARS-CoV-2 variants of concern (VOCs), such as Alpha, Beta, and Delta with potency superior to remdesivir (Fig. 7F and fig. S26, D to H). We next sought to understand the potential mechanism by which DCAF7 inhibitors modulated SARS-CoV-2 infection. We examined whether enhanced lysosomal activity affected individual SARS-CoV-2 viral protein(s), thus impairing the viral life cycle. We prepared an expression library of the SARS-CoV-2 proteome to measure changes in the steady-state levels of individual viral proteins with DCAF7 inhibitor treatment (fig. S27A) (*65*). Viral proteins exhibited a broad range of responses, with several key proteins showing a marked decrease in expression upon DCAF7 inhibitor treatment (figs. S27B and S28), including the highly conserved helicase NSP13, required for replication of a wide range of coronaviruses (*66*, *67*). The lysosomal inhibitor leupeptin largely counteracted the effect of our DCAF7 inhibitors in the clearance of viral protein, with numerous viral proteins levels rendered insensitive to DCAF7 inhibitor effect upon leupeptin treatment. Together, this indicates a lysosomal dependence of the antiviral action of our small molecules (Fig. 7, G and H, and fig. S29, A and B).

**Fig. 8.**
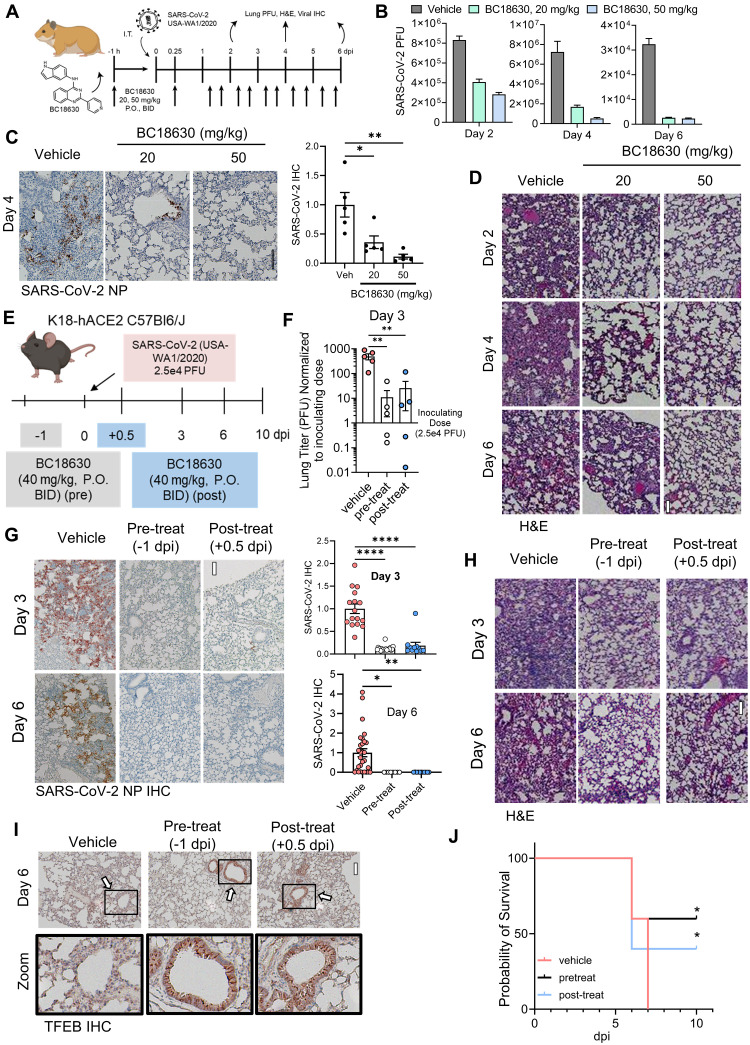
In vivo protection from coronaviral infection using small molecule DCAF7 inhibitors. (**A** to **D**) In vivo SARS-CoV-2 hamster infection model. (A) Five Syrian golden hamsters per group were treated with BC18630 (P.O.) one hour prior to inoculation with SARS-CoV-2 (i.t.). Six hours later, a second dose of BC18630 was administered and then BID for 5 days. Animals were euthanized on days 2, 4, and 6 dpi. (B) SARS-CoV-2 PFU assays from pooled lungs tissue lysate of *n* = 3 animals. Data represent means ± SEM of technical replicates (*n* = 3). (C) IHC of SARS-CoV-2 NP in lung at 4 dpi; signal was quantified from five random fields; means ± SEM. (D) Representative images of H&E-stained lungs. (**E** to **J**) SARS-CoV-2 K18-hACE2 C57BL/6J transgenic mice model with pre- and postinfection BC18630 treatment. (E) Mice infected with SARS-CoV-2 (USA-WA1/2020, 2.5 × 10^4^ PFU) were either given BC18630 (40 mg/kg) 24 hours before infection (pre-treat, BID thereafter), 12 hours after infection (post-treat, BID thereafter), or vehicle treatment. Groups were euthanized on days 3 and 6, with a survival experiment lasting until day 10. (F) Lung viral titer at 3 dpi using plaque assay; initial inoculated dose shown as reference. Data represent means ± SEM of *n* = 5. (G) IHC of SARS-CoV-2 NP in lung at 3 and 6 dpi. Signal was quantified, *n* = 8 to 29 random fields per treatment; data represent means ± SEM. (H) H&E histology of infected mouse lungs. I. IHC of host TFEB protein in lung at 6 dpi. Arrows indicate TFEB staining in airways. (J) Survival analysis, data shown using the Kaplan-Meier plot. *N* = 5 mice per treatment, 0/5 vehicle mice survived, 3/5 pretreated mice survived, and 2/5 posttreated mice survived. n.s. *P* > 0.05; *P < 0.05; **P < 0.01; ****P < 0.0001 as indicated by one-way ANOVA with Dunnett’s post hoc [(C) and (G)] or Fleming-Harrington test (J). Scale bar, 100 μm.

To further characterize the utility of our DCAF7 inhibitors, we next assessed an in vivo model of SARS-CoV-2 infection. We found that BC18630 displayed a superior pharmacokinetic (PK) profile in rodents compared to BC18813, with a high exposure in the lung compartment relative to plasma (fig. S30, A to C). BC18630 persisted in the lung at therapeutic levels up to 8 hours postadministration, which supported a twice daily (i.e., BID) dosing regimen (fig. S30C). As part of an initial assessment of target engagement, we demonstrated that BC18630 administration increased the mouse hepatic endolysosomal capacity, consistent with a compound-induced increase in TFEB activity (*68*–*70*) (fig. S30D). Next, we used a Syrian hamster model of SARS-CoV-2 infection and treated with vehicle or two doses of BC18630 1 hour prior to viral infection (oral BID at 20 and 50 mg/kg for 6 days, respectively) (Fig. 8A). We observed that BC18630 dose-dependently reduced lung viral titer [plaque-forming units (PFU)] at all time points (Fig. 8B). The higher dosing strategy produced a >90% viral load reduction at 4 and 6 days postinfection (dpi). Immunohistochemical (IHC) staining of lung slices for SARS-CoV-2 N-protein (NP) 4 dpi revealed notably reduced NP-positive epithelial staining with BC18630 treatment (Fig. 8C). BC18630 also mitigated the observed levels of lung injury, with the higher dose particularly reducing inflammatory infiltrates (Fig. 8D). Last, we tested the DCAF7 inhibitor intervention in a SARS-CoV-2 human ACE2 transgenic K18-hACE2 mouse model. Mice were treated with compound prior to (−1 day) or post (+0.5 day) SARS-CoV-2 infection (oral BID at 40 mg/kg for 10 days) (Fig. 8E). We observed substantialy decreased viral titer in mouse lungs 3 dpi for both treatments, as well as a notably decreased viral IHC signal and decreased pulmonary injury by histological analysis (Fig. 8, F to H). Consistent with the mechanism of action of our agents, we also noted an increase in TFEB IHC signal in the airways of BC18630 treated mouse lung samples (Fig. 8I and fig. S31). Furthermore, both treatment strategies resulted in notably prolonged survival relative to vehicle (Fig. 8J). In summary, BC18630 appears to decrease lung viral titer, reduce viral protein abundance, and prevent adverse lung pathophysiology in two animal models of SARS-CoV-2. These data demonstrate the utility of pharmacological-based methods to modulate TFEB levels as an antiviral strategy (fig. S32).

## DISCUSSION

Previous studies have demonstrated that many viruses have evolved mechanisms to circumvent or hijack the endolysosomal pathway (*17*, *71*, *72*). Our data demonstrate that viral infection induces the proteasomal degradation of TFEB. We observed that PAK2 activation is a critical priming step for TFEB degradation as this kinase is activated by viral infection, generating a phospho-degron required for subsequent DCAF7 recognition (see the model in fig. S32). Other groups have independently characterized the PAK kinase family, including PAK2, as associated with viral infection, including recent studies with SARS-CoV-2 (*73*, *74*). In the case of HIV, following infection, retroviral-encoded proteins directly bind to PAK2 leading to its activation (*74*). Whether a similar scenario exists for SARS-CoV-2, or whether coronaviruses trigger a more generalized stress response that leads to PAK2 activation, remains to be elucidated. Intriguingly, either PAK2 deletion or genetic/pharmacological DCAF7 inhibition ablated virally induced TFEB degradation, thereby maintaining lysosomal fitness and improving the host response. Although our study has concentrated on boosting TFEB levels during infection, the efficacy of DCAF7 inhibitors to modulate TFEB activity under basal conditions suggests potential application of this approach for other, noninfectious conditions associated with impaired autolysosomal activity. Pharmacological activation of TFEB represents an attractive target for a growing number of diseases (*23*, *75*–*77*).

Even as COVID-19 pneumonia progresses from a pandemic respiratory virus to an endemic respiratory virus, it continues to pose a serious threat to public health (*78*). For example, incident COVID-19–associated hospitalization rates for COVID-19 pneumonia—which reflect “severe” disease with lung injury and need for supplemental O_2_—remain higher than for influenza during the 2023–2024 respiratory viral season in the United States (CDC). This pattern is also true for respiratory virus-associated mortality, where COVID-19 pneumonia continues to pose a larger mortality threat than influenza or Rous sarcoma virus in 2023–2024 (CDC). These epidemiological patterns have emerged despite the widespread availability of vaccines and one approved virus-directed therapeutic. Given the high transmissibility and mutational rate of SARS-CoV-2, critical concerns exist whether virus-directed strategies will retain efficacy against all future coronavirus outbreaks. Our host-targeted small molecule DCAF7 inhibition therapy, which showed efficacy against SARS-CoV-2 in cell and animal-based infection models, can likely supplement current antiviral approaches. Moreover, this host-centric approach has a mechanism of action that supports potential efficacy in future pandemics triggered by currently unknown VOCs.

Cellular OC43 infection leads to lysosome alkalization, however, our pharmacological inhibition of TFEB protein degradation maintained the acidity of the lysosome and its enzymatic capacity in response to β-coronaviral infection. Using engineered exogenous protein expression, we were able to demonstrate that multiple SARS-CoV-2 viral proteins were subjected to lysosomal-mediated protein degradation, and this degradation process was facilitated by our pharmacological approach for TFEB potentiation. Of particular note, several nonstructural proteins (NSPs) underwent expedited protein degradation with BC18630 treatment. This suggests that compound-mediated TFEB activation could potentially impair the viral life cycle through inhibiting NSP-mediated viral RNA genome translation and replication, which leads, in turn, to decreased virion assembly, release, and transmission. The genetic and pharmacological maintenance of TFEB protein levels and the concomitant viral load reduction within a single viral life cycle (estimated 24 hours) supports the proposition that TFEB-mediated lysosomal homeostasis facilitates the efficient clearance of viral proteins as a host-oriented antiviral mechanism. However, we acknowledge that notable differences may exist between exogenously expressed individual viral proteins and viral protein turnover in the setting of infection with intact virus (*79*). The additional mechanisms of action in which BC18630-mediated TFEB regulation affects host response to coronaviral infection necessitates further interrogation in future studies.

Several other recent studies have demonstrated the complexity of TFEB-driven host response to viral infection in various model systems. Contreras *et al.* (*80*) noted that mouse hepatitis virus (MHV) infection induced the nuclear translocation of TFEB, and its homolog TFE3, in HeLa cells stably expressing the murine receptor for MHV, leading to the transcriptional activation of several immune pathways and stimulation of lysosomal exocytosis. In agreement with our findings, TFEB activation following viral infection occurred without a noticeable change in mTORC1 activity. These authors also noted a decline in TFEB levels beginning roughly 16 hours after MHV infection that was DCAF7 mediated. In contrast to our observations in human bronchial epithelial cells infected with common cold viruses, a DCAF7-regulated decline in TFE3 protein was also observed in HeLa cells challenged by MHV. Furthermore, TFEB/TFE3 mediated the host transcriptional up-regulation of genes related to immune and inflammatory response and apoptosis, along with the TFEB/TFE3-activated MHV lysosomal exocytosis, demonstrating that TFEB/TFE3 depletion could reduce caspase-induced cell death and limit the viral release from the infected cells. Another study of SARS-CoV infection (*81*) indicated that translation of the viral open reading frame (ORF) 8b (ORF8b) formed insoluble protein aggregates, therefore inducing endoplasmic reticulum (ER) stress and lysosome damage. This led to TFEB activation and its target gene transcription, eventually resulting in epithelial cell death. ORF8b also triggered inflammasome activation and pyroptotic cell death of macrophages. These studies collectively suggest that, during the early phase of infection, coronaviral pathogens may induce TFEB nuclear translocation and transcriptional activation to restore lysosomal homeostasis. With prolonged exposure to a viral challenge, there is a decrease in TFEB protein, disrupting host homeostasis, affecting both viral infectivity and host cell viability. We speculate that the various host responses observed in different model systems could potentially be influenced by differences in the cell and tissue types studied, virulence of the viral strains, duration of infection, as well as other cellular and viral factors. Nonetheless, our strategy to maintain endogenous TFEB protein levels and activity could serve as a physiological means to strengthen intrinsic host response to invading pathogens with minimal disturbance to overall cellular homeostasis.

As noted previously (*16*), and as observed here, coronavirus infection leads to a deacidification of the host’s lysosome. These observations are broadly consistent with several recent genome-wide CRISPR-based screens, which identified genes involved in endolysosomal function as major cellular determinants of resistance following infection by a wide range of coronaviral strains (*82*, *83*). Similar, it has been appreciated for some time that bacterial infection induces endolysosomal damage (*84*), and more recently, it has been demonstrated that bacteria have evolved sophisticated mechanism of host evasion including direct modification of the V-ATPase required for organelle acidification (*27*). As such, the endolysosomal system plays a critical role in the ultimate fate of host-pathogen interactions. Expectedly, in organisms such as *C. elegans* that lack a cellular immune system, the worm ortholog of TFEB has been shown to orchestrate the predominant response to infection (*24*, *85*). Together with our observations, these studies suggest that TFEB orchestrates an evolutionary conserved host response and that, in the setting of pathogen exposure, therapeutic strategies that stabilize or augment TFEB activity may have widespread benefit.

## MATERIALS AND METHODS

### Contact for reagent and resource sharing

A material transfer agreement needs to be completed to get access to the compound BC18630.

### Materials

Please see the materials list in table S3.

### Experimental model and subject details

#### Cell culture

BEAS-2B and MLE-12 cells from the American Type Culture Collection (ATCC) were cultured in Dulbecco’s modified Eagle’s medium (DMEM)/F-12 media (Gibco) supplemented with 10% fetal bovine serum (FBS) (Gibco) and penicillin-streptomycin (Gibco, 10,000 U/ml, 15140163). HCT-8 (ATCC CCL-244) cells were cultured in RPMI 1640 medium (Gibco, ATCC modification, A1049101) supplemented with 10% FBS and penicillin-streptomycin. MRC-5 (ATCC CCL-171) and MDCK (ATCC CCL-34) cells were cultured in Eagle’s minimum essential medium (EMEM) (ATCC 30-2003) supplemented with 16% FBS and penicillin-streptomycin. KO cell lines were prepared using lentivirus. Sequences for target genes were created with GPP sgRNA Designer (*86*) and cloned into pLENTI-CRISPR-vs2 (*87*). Lentiviral particles were generated by coexpression of sgRNA encoded pLENTI-CRISPR-vs2 with psPAX2 and pMD2.G in human embryonic kidney (HEK) 293T. Target cells were incubated with lentivirus prior to antibiotic selection and generation of monoclonal populations. Validation of KO was determined by immunoblotting and DNA sequencing (see table S2 for list of antibodies and other materials). Cells were treated with compound at indicated doses for indicated times or at the following doses: 20 nM carfilzomib, 2 μM leupeptin, and 0.2 μM bafilomycin A1 (BFA). Cytosolic and nuclear fractions were separated using NE-PER Nuclear and Cytoplasmic Extraction Reagents (Thermo Fisher Scientific).

#### Viral propagation and inoculation

The propagation methods and times are based on ATCC recommendations.

##### Human coronavirus OC43, Betacoronavirus 1, ATCC VR-1558, LOT: 70034234

HCT-8 cells were cultured on T75 flasks to 90% confluence. The growth media were removed, and the cells were washed twice with serum-free medium (RPMI 1640 medium with penicillin-streptomycin). Three hundred microliters of the stock OC43 was diluted in 5 ml of serum-free medium, and the virus dilution was adsorbed on the cells for 1 hour at 34°C with 5% CO_2_. The adsorption was ended by adding 10 ml of serum-free medium on the cells, and the virus was propagated for 5 days at 34°C with 5% CO_2_. The viral supernatant was collected by centrifugation at 1000*g* for 10 min at room temperature (RT). To propagate more virus after the stock solution finished, 1 ml of the supernatant was added in 9 ml of serum-free medium for adsorption on a 90% confluent T175 flask of cells, and it was ended by adding 20 ml of serum-free medium per flask and propagated as previously described.

##### Human coronavirus 229E, ATCC VR-740, LOT: 70034235

MRC-5 cells were cultured on T75 flasks to 90% confluence. The growth media were removed, and the cells were washed twice with serum-free medium (EMEM with penicillin-streptomycin). Three hundred microliters of the stock 229E was diluted in 5 ml of serum-free medium, and the virus dilution was adsorbed on the cells for 1 hour at 34°C with 5% CO_2_. The adsorption was ended by adding 20 ml of serum-free medium on the cells, and the virus was propagated for 4 days at 34°C with 5% CO_2_. The viral supernatant was collected by centrifugation at 1000*g* for 10 min at RT. To propagate more virus after the stock solution finished, 1 ml of the virus dilution was added in 9 ml of serum-free medium for adsorption on a 90% confluent T175 flask of cells, and it was ended by adding 20 ml of serum-free medium per flask and propagated as previously described.

##### Cell inoculation

Compound and virus dilutions were prepared in DMEM low glucose (Gibco, 11885092) with 2% FBS (Gibco) and penicillin-streptomycin (Gibco). Occasionally, specific medium for each cell line (RPMI/EMEM) was used with 2% FBS and penicillin-streptomycin for viral treatments. The calculated multiplicity of infection (MOI) of the virus was 0.3 MOI or as noted in experiment.

##### In vivo PK study of DCAF7 inhibitor

PK studies of DCAF7 inhibitors were conducted at Touchstone Biosciences. Compounds were assayed in Sprague-Dawley (SD) rats. Male SD rats were fed a standard laboratory rodent diet and housed in individual cages on a 12-hour light and 12-hour dark cycle with RT maintained at 22° ± 3°C and relative humidity at 50 ± 20%. Animals were typically fasted overnight before dosing, with food returned after the 6-hour blood samples were obtained. Water was provided ad libitum throughout the study. Animals were dosed via gavage needle for oral administration at 10 mg/kg (10 ml/kg) or via tail vein injection for intravenous administration at 5 mg/kg (2 ml/kg). All blood samples (30 to 80 μl per sample) were taken via appropriate vein (saphenous or jugular vein) at 5, 15, and 30 min and 1, 2, 4, 6, 8, and 24 hours after dosing. Blood samples were collected in Greiner MiniCollect K2EDTA tubes, placed on ice, and within 30 min, centrifuged at 15,000*g* for 5 min to obtain plasma samples.

Exposure of DCAF7 inhibitor to lung tissue was conducted in male CD-1 mice. Mice were fed a standard laboratory rodent diet and housed in individual cages on a 12-hour light and 12-hour dark cycle with RT maintained at 22° ± 3°C and relative humidity at 50 ± 20%. Animals are typically fasted overnight before dosing, with food returned after the 6-hour blood samples are obtained. Water is provided ad libitum throughout the study. Mice were dosed via gavage needle for oral administration at 80 mg/kg (20 ml/kg). All blood samples (25 to 30 μl per sample) are taken via appropriate vein (saphenous or submandibular vein) at 1, 2, 4, 8 and 24 hours after dosing. Blood samples are collected in Greiner MiniCollect K2EDTA tubes, placed on ice, and within 30 min, centrifuged at 15,000*g* for 5 min to obtain plasma samples.

Lung tissue samples were harvested, immediately rinsed in water briefly, and blotted dry with a paper towel. Each tissue was then divided into two portions, one for bioanalysis and the other for biomarker analysis. For bioanalysis, each tissue was weighed and three volumes of phosphate-buffered saline (PBS) buffer (pH 7.4) were added to one volume of each tissue sample, which was homogenized by a tissue homogenizer until fine tissue particles were completely dispersed or emulsified. The tissue homogenate samples were stored at −70°C until analysis.

All plasma samples were prepared as follows: Three volumes of acetonitrile containing the internal standard were added to one volume of plasma to precipitate proteins. Samples were centrifuged (3000*g* for 10 min) and supernatant removed for analysis by liquid chromatography (LC)–MS/MS. Calibration standards and quality controls were made by preparation of a stock solution (1 mg/ml) and, subsequently, a series of working solutions in methanol:water (1/:1, v/v), which were spiked into blank plasma to yield a series of calibration standard samples in the range of 1 ng/ml to 10 μg/ml and quality control samples at three concentration levels (low, middle, and high). All incurred PK plasma samples were treated identically to the calibration standards and quality control samples. LC-MS/MS analysis was performed using multiple reaction monitoring for detection of characteristic ions for each drug candidate, additional related analytes, and internal standard. Compound plasma concentrations were measured to determine a concentration versus time profile. The area under the plasma concentration versus time curve (AUC) was calculated using the linear trapezoidal method. Fitting of the data to obtain PK parameters is generally carried out using noncompartmental analysis. All parameters were expressed for individual animals as well as mean, SD, and coefficient of variation.

Tissue samples were prepared as follows: Three volumes of PBS buffer (pH 7.4) were added to one volume of each tissue sample, which was then homogenized to obtain each tissue homogenate sample. Subsequently, three volumes of acetonitrile containing the internal standard was added to one volume of each tissue homogenate, and the mixture was vortexed, centrifuged (3000*g* for 10 min) and supernatant removed for analysis by LC-MS/MS. Calibration standards were made by preparation of a stock solution (1 mg/ml) and, subsequently, a series of working solutions in methanol:water (1:1, v/v), which were spiked into blank tissue homogenate to yield a series of calibration standard samples in the range of 1 ng/ml to 10 μg/ml. All incurred PK tissue samples were treated identically to the calibration standards. LC-MS/MS analysis was performed using multiple reaction monitoring for detection of characteristic ions for each drug candidate, additional related analytes, and internal standard.

##### RNA-seq of DCAF7 inhibitor-treated cells

RNA-seq was conducted at MedGenome (Foster City, CA, USA). BEAS-2B cells were treated with BC18630 (3 μM, 18 hours) in DMEM low glucose with 2% FBS. Total RNA was extracted and purified using the RNeasy Plus Mini Kit (Qiagen). A Takara SMART-Seq v4 Ultra-low Input RNA kit was used with a NovaSeq sequencer set to a read length of 150. RNA-seq quality control was performed using RNA-SeQC (v1.1.8), RSeQC (v3.0.1), and MultiQC (v1.7). Unwanted sequences, such as mitochondrial sequences, ribosomal RNAs, transfer RNAs, and adapters were removed using Bowtie2 (v2.5.1). The paired-end reads were aligned to the reference Human genome (GRCh37/hg19 release 75) using the STAR (v2.7.3a) aligner. The raw read counts were estimated using HTSeq (v0.11.2). Read counts were normalized using DESeq2 to get the normalized counts. In addition, the aligned reads were used for estimating expression of the genes using cufflinks (v2.2.1). The expression values were reported in FPKM (fragments per kilobase per million) units for each gene. Annotated TFEB transcriptional targets were filtered using independent hypothesis filter (DESeq2). The RNA-seq GEO accession number is GSE292982.

##### Murine model of hepatic lysosomal activity

Lysosomal activity in mice was determined through dextran cascade blue uptake (*69*, *70*). All procedures were approved by the University of Pittsburgh Institutional Animal Care and Use Committee (IACUC protocol IS00024438). Ten- to 12-week-old C57BL/6 mice were given BC18630 (50 mg/kg, daily for 4 days) through intraperitoneal injection. Two hours after the last dose, mice were intravenously injected with dextran cascade blue as previously described (*70*). Mice were then euthanized, and liver tissue was fixed, sectioned, and imaged for dextran cascade blue fluorescence and stained for LAMP1 (lysosome).

#### In vitro SARS-CoV-2 infection assay

SARS-CoV-2 infectivity was assayed through infection of Calu-3 at the CRO IIT Research Institute (Chicago, USA). Human lung cancer Calu-3 cells were maintained in EMEM with 10% FBS. Cells were cultured in a 96-well plate until reaching 80 to 90% confluency. Test articles were tested against WT USA-WA-1/2020 (SARS-CoV-2) in six replicates. Compounds were serially diluted, and cells were pretreated for 24 hours. After pretreatment, the test articles were removed and then incubated with a standardized virus concentration at 37° ± 2°C in 5 ± 1% CO_2_ for 75 ± 15 min. Following the 75 ± 15 min incubation, virus inoculum was removed, cells washed, and appropriate wells overlaid with test or control articles in 0.2 ml of EMEM2 (EMEM with 2% FBS) and incubated in a humidified chamber at 37° ± 2°C in 5 ± 2% CO_2_. At 48 ± 6 and 72 ± 6 hours postinoculation, from each plate, 120 μl of the supernatant from each well was collected for subsequent analysis by quantitative polymerase chain reaction (qPCR). The concentration of virus in the cell culture supernatants was determined by a qPCR assay. Briefly, samples kept at ≤−65°C were thawed and centrifuged to remove cellular debris. RNA was extracted using the Quick-RNA Viral Kit (Zymo Research) according to the manufacturer’s protocol. qPCR was performed using the following PCR cycling conditions: 50°C for 15 min (RT), then 95°C for 2 min (denature), then 40 cycles of 10 s at 95°C, 45 s at 62°C. Virus titer by qPCR was performed according to IITRI SOPs. Assays using SARS-CoV-2 VOCs were conducted at the Viroclinics CRO (Viroclinics Biosciences B.V) using Vero E6 cells. Ten 0.5log_10_ serial dilutions of compounds in infection medium—highest final concentrations were 29.6 μM (BC18630), 28.1 μM (BC18813), and 16.6 μM (remdesivir)—were added to the cells 16 hours prior to infection (pretreatment). On the day of infection, the infection medium was removed from the cells and replaced with a 1:1 mixture of virus and 2x concentrated compound dilutions (highest final concentration same as above). Column 11 contained the virus control wells, and column 12 contained the cell control wells. After an overnight incubation, the cells were fixed and virus infected cells were immunostained with a monoclonal antibody, which targets the SARS-CoV-2 nucleocapsid protein (NP), followed by a secondary goat anti-mouse immunoglobulin G (IgG) peroxidase conjugate and TrueBlue substrate. This formed a blue precipitate on NP-positive cells. Images of all wells were acquired by a CTL ImmunoSpot analyzer, equipped with software to quantitate the NP-positive cells, and infection metrics were quantified by proportion of well surface area covered by viral positive immunostaining. IC_50_ values were calculated using the formula previously described (*88*).

#### In vivo hamster SARS-CoV-2 infection assay

SARS-CoV-2 infectivity was assayed through Syrian golden hamster models of infection. ChemDiv Inc. was contracted to conduct in vivo SARS-CoV-2 infection assays. Animals were allowed to acclimate to their environment for 2 weeks prior to the assay. Briefly, BC18630 was administered by oral route in two doses, 20 and 50 mg/kg, twice a day, for 6 days. The first dose of the drugs was administered 1 hour prior to SARS-CoV-2 infection (10^3^ CPE_50_ of SARS-CoV-2 virus at 100 μl per animal), the next dose was given 6 hours postinfection. Hamsters were euthanized on days 2, 4, and 6 postinfection. Lungs tissues were collected for the antiviral activity that was calculated based on the viral titer (PFU assay) of pooled lung samples. The collected lung tissues were also fixed and stained for pathology analysis.

#### In vivo mouse SARS-CoV-2 infection assay

The efficacy of prophylactic and therapeutic strategies of DCAF7 inhibitor BC18630 administration was tested in a SARS-CoV-2 human ACE2 transgenic mouse model. Briefly, 6- ~ 7-week-old female K18-hACE2 mice (JAX:034860) were assigned to a control group, treated with water, pretreatment group receiving 40 mg/kg (PO) at 1 day prior to viral infection, and BID thereafter, or posttreatment group, receiving 40 mg/kg (PO) at 0.5 day after viral infection, with BID dosing regimen thereafter. All groups were intranasally infected with 2.5 × 10^4^ PFU of WT USA-WA-1/2020 SARS-CoV-2 and were monitored and weighed daily. Mice were euthanized on day 3 and day 6 postinfection for lung viral titer measurement and histological analyses. For survival studies, mice were monitored twice per day and euthanized if weight dropped 20% of the starting point. Survival data were analyzed with the Fleming-Harrington log-rank test, with *P* < 0.05 indicative of statistical significance. All animal experiments were approved by the University of Pittsburgh Institutional Animal Care and Use Committee (IACUC protocol 22010457) and conducted in a BSL3 facility.

#### Panlabs

Off-targeting effects of DCAF7 inhibitors BC18813 and BC18630 were tested with the Eurofins Panlab Safety47 Safety Scan. This panel encompasses several common enzymes and pathways (GPCR, kinase, ion channel, etc.), which are safety concerns for off-targeting of drug candidates and allow anticipation of potential adverse effects (*89*). Effects of BC18813 and BC18630 on this panel were measured, and the IC_50_/median effective concentration (EC_50_) of potential inhibitory/stimulating activity was noted.

### Method details

#### Immunoblotting

Cells were lysed in radioimmunoprecipitation assay buffer supplemented with EDTA-free protease inhibitor tablet (Thermo Fisher Scientific) on ice. Cell lysates were sonicated at 20% amplification for 12 s and centrifuged at 12,000*g* for 10 min at 4°C. Supernatants were collected and normalized for the total protein concentrations, mixed with 6X protein sample buffer, and incubated at 42°C for 10 min. Sample lysate was resolved using 4 to 20% acrylamide PROTEAN TGX precast gels from Bio-Rad and electrophoresed in Tris-Glycine-SDS (TGS) buffer. The proteins were then electrotransferred to nitrocellulose membranes. Blots were incubated in 15 ml of blocking buffer for 1 hour at RT, before incubation in 10 ml of the primary antibody solution (1:1000 dilution) overnight at 4°C. Afterward, three 10-min washes were performed in 15 ml of Tris-buffered saline +0.05% Tween 20 (TBST). Blots were then incubated with 10 ml of the secondary antibody solution for 1 hour at RT. After three 10-min washes in 15 ml of TBST, blots were then developed using the West Femto Maximum Sensitivity Substrate from Thermo Fisher Scientific and imaged using the ChemiDoc Imaging System from Bio-Rad. Immunoblot signal was quantified by using densitometry (ImageJ), in which protein-of-interest intensity was corrected to cognate β-actin intensity and then normalized to the specific experimental control or vehicle treatment. Data were represented as means ± SEM.

#### Quantitative PCR

Total RNA was extracted using the RNA Extraction Miniprep Kit from Bioland Scientific or using the RNeasy Plus Mini Kit from Qiagen following the manufacturer’s protocol. cDNA was prepared using the High-Capacity RNA-to-cDNA Kit from Applied Biosystems. SYBR Green Real-Time PCR Master Mixes from Applied Biosystems were used in qPCR. Primer sequences are detailed in the oligonucleotide table (table S3).

#### High-throughput liquid handling

A Thermo Fisher Scientific custom HTS platform, and Agilent Bravo automated liquid-handling platform were used to transfer contents of an FDA-approved compound library into assay plates. A Biotek EL406 washer dispenser was used to distribute reagents or cell solutions into assay plates. For multiple plates operation, the plate and liquid handling sequence and intervals were controlled through the Agilent VWORKs software.

#### FDA-approved compound library screening

BEAS-2B cells stably expressing TFEB-EGFP were seeded to a final density of 2500 cells per well in a black 384-well plate with glass bottoms. Cells were treated with OC43 for a 24-hour period before exposure to the FDA compound library. The FDA-approved compound library (Selleck, 100 nl per drug) was stamped to a 384-well plate using CyBio Well vario (Analytik Jena). Compounds were then added to cells with the final concentration of 2 μM. After 18 hours of treatment, cells were then fixed in 4% paraformaldehyde (PFA) and counterstained with 4′,6-diamidino-2-phenylindole (DAPI), and fluorescence was detected using Cytation5 High Content Imager (BioTek). Nuclear TFEB fluorescent signal was calculated using Gen5 software (BioTek).

#### Plasmid preparation and cloning

TFEB and DCAF7 coding sequences were subcloned into pcDNA3.1D-V5-HIS (Invitrogen) through TOPO cloning. Point mutants (Lys to Arg; Ser to Ala; Ser to Asp) were generated through the QuikChange II XL Site-Directed Mutagenesis Kit (Agilent). Viral protein cDNA set was a gift from N. Krogan [Addgene plasmids from (*65*)] and was cloned into the Nano-luciferase HiBiT expression system (Promega). All plasmid constructs were verified by DNA sequencing (Genewiz).

#### Plasmid transfection

Plasmid transfections were conducted using nucleofection in BEAS-2B and MLE-12 cells using Nucleofector II (Amaxa). X-tremeGENE HP DNA transfection reagent or Lipofectamine 3000 transfection reagent was used for plasmid transfections.

#### Cell-based ubiquitination assay

TFEB-V5-HIS WT and mutants in pcDNA3.1D or TFEB-EGFP were coexpressed with hemagglutinin-tagged ubiquitin and DCAF7 in BEAS-2B cells for 18 hours, prior to proteasomal inhibition where indicated, lysis and precipitation with Dynabead HIS-resin (Thermo Fisher Scientific) or GFP-Trap beads (ChromoTek). Precipitate was eluted in 1x Laemmli Protein Sample Buffer at 95°C for 10 min and resolved through SDS–polyacrylamide gel electrophoresis (PAGE) immunoblotting.

#### TUBE PD assay

TUBE PD was conducted as previously described (*90*). WT or KO BEAS-2B cells were infected with OC43 at indicated MOI for 24 hours prior to collection in immunoprecipitation (IP) buffer [137 mM NaCl, 2.7 mM KCl, 10.14 mM Na_2_HPO_4_, 1.76 mM KH_2_PO_4_, and 0.25% (v/v) Triton X-100 supplemented with Pierce protease and phosphatase inhibitor cocktail], sonication, and precipitation (12,000 rcf for 10 min at 4°C). Lysate (300 μl) was incubated with 50 μl of TUBE-2 magnetic beads (LifeSensors) for 2 hours prior to washing, elution, and SDS-PAGE and blotting analysis of TFEB protein levels in eluate. Whole-cell lysate input was retained as a control.

#### TFEB mass spectrometry

TFEB-EGFP MLE-12 cells or control MLE-12 cells were incubated with GFP-Trap magnetic resin (ChromoTek) followed by washing and freezing of resin. Samples were processed and analyzed by MSBioworks (Ann Arbor, MI). Briefly, samples were boiled at 100°C for 15 min in 60 μl of 1.5X LDS buffer. The beads were removed and half of each submitted sample was processed by SDS-PAGE using a 10% Bis-Tris NuPAGE gel (Invitrogen) with the MES buffer system. The mobility region was excised into 10 equal sized segments, and in-gel digestion was performed on each using a robot (ProGest, DigiLab) with the following protocol: washed with 25 mM ammonium bicarbonate followed by acetonitrile, reduced with 10 mM dithiothreitol at 60°C followed by alkylation with 50 mM iodoacetamide at RT, digested with sequencing grade trypsin (Promega) at 37°C for 4 hours, quenched with formic acid and the supernatant was analyzed directly without further processing. Half of each digested sample was analyzed by nano LC-MS/MS with a Waters NanoAcquity high-performance liquid chromatography (HPLC) system interfaced to a Thermo Fisher Scientific QExactive. Data were filtered using 1% protein and peptide false discovery rate (FDR) and requiring at least two unique peptides per protein.

For phosphoproteomics studies, TFEB-EGFP MLE-12 cells were treated with vehicle or carfilzomib (1 μM for 4 hours) prior to trypsinization and snap freezing. Cell lysate was sent to MSBioworks. Briefly, samples were boiled at 100°C for 15 min in 60 μl of 1.5X LDS buffer. Beads were removed, and half of each submitted sample was processed by SDS-PAGE using a 4 to 12% Bis-Tris NuPAGE gel (Invitrogen) with the Mops buffer system. The target band (TFEB+GFP) was excised and processed by in-gel digestion using a robot (ProGest, DigiLab). Data were filtered using 1% protein and peptide FDR and requiring at least two unique peptides per protein. The Mass Spectrometry dataset is on Dryad (DOI: 10.5061/dryad.p2ngf1w2s).

#### Immunocytochemistry

BEAS-2B, MRC-5, or HCT-8 cells were seeded in 384-well glass-bottom plates (Cellvis, 5000 cells per well) prior to the indicated treatment. Viral inoculations were treated for 24~72 hours prior to collection. Following treatment, cells were fixed (4% PFA) and permeabilized (0.5% Triton X-100), and the nucleus was counterstained with DAPI or Hoechst 33342. Fluorescent signals were imaged using Image Express (Molecular Devices) for HCS experiments, or a Leica SP8 Confocal. Signal and nuclear-cytosol ratio were quantified using Cell Profiler (*91*).

#### Protein half-life assay

To measure protein stability, the ribosomal inhibitor cycloheximide (CHX) was used to halt protein synthesis, and TFEB protein abundance was measured through immunoblotting. Either WT or *DCAF7* KO BEAS-2B cells transfected with V5-tagged WT or K232R TFEB plasmid were treated with CHX at 0.1 mg/ml for the indicated times before cell lysis and processing for immunoblotting. TFEB protein levels were detected via immunoblotting, and densitometry was performed using Fiji-ImageJ (*92*).

#### Cell viability assessment

Cell viability was tested using the CellTiter-Glo 2.0 Cell Viability Assay (Promega). A 20-μl reagent was dispensed directly into each well of the 384-well tissue culture plates prior to luminescence signal acquisition by a Cytation 5 plate reader. Sample signal intensity was normalized to relevant controls and represented as means ± SEM.

#### TFEB-EGFP nuclear localization protocol

BEAS-2B cells stably expressing TFEB-EGFP were plated at 2500 cells per well in 384-well glass-bottom plates (Cellvis) in DMEM/F-12, glucose (3.2 g/liter; Gibco) containing 10% FBS. Cells were left at RT for 30~60 min before transferring to a standard tissue culture incubator. Six hours later, an equal volume of DMEM/F-12, glucose (1 g/liter; Gibco) containing 2% FBS was added, and the cells returned to the TC incubator. The next day cells were fixed by adding a half volume of prewarmed 8% PFA/PBS containing Hoechst 33342 (10 μg/liter) and incubated for 1 hour. The cells were washed twice in PBS using a Biotek ELx405 automated washer and plates sealed. The cells were imaged using an ImageXpress Micro automated imager using a 10x objective and native camera resolution (Molecular Devices). The nuclear Hoechst and TFEB-EGFP signal were recorded. Using the Hoechst signal to define the nuclei, CellProfiler software was used to calculate the ratio of nuclear-to-cytoplasmic TFEB-EGFP localization for each cell. One field was used per well and the median nuclear-to-cytoplasmic TFEB-EGFP signal reported. For this calculation, the cytoplasmic signal was defined as the average GFP signal in a ring six pixels wide around the nucleus and the nuclear signal was defined as the average GFP signal in the nucleus.

#### Lysosomal number and activity

Cells were plated in 96-well glass-bottom plates (Cellvis) in 100-μl total volume with test compounds and left at RT for 30~60 min to allow cells to attach prior to transfer to a tissue culture incubator. The next day, 70 μl of media was aspirated and 15 μl of media containing Hoechst 33342 (10 μg/ml) and the following reagents added: for LysoTracker Red staining: CellTracker Green 1:1000 and LysoTracker Red 1:2500; for Magic Red activity: CellTracker Green 1:1000 and Magic Red 1:125. The plate was returned to the incubator for 80 min. Next, the media were aspirated and 250 μl of imaging solution added [150 mM NaCl, 5 mM KCl, 1 mM MgCl_2_, 2 mM CaCl_2_, 5% FBS, glucose (1 g/liter), and 20 mM Hepes (pH 7.4)]. The cells were then immediately imaged using an ImageXpress Micro XLS automated imager with 10x objective and the appropriate image filters (Molecular Devices). CellProfiler software was used to calculate the mean LysoTracker or Magic Red signal per cell, using the CellTracker signal as a mask to outline each cell. One field was used per well and the median signal for each well reported.

#### TFEB-EGFP live-cell imaging

For live-cell imaging, BEAS-2B cells stably expressing TFEB-EGFP were plated in glass-bottom 96-well plates in full growth media. The next day, the media were removed and replaced with modified imaging solution [140 mM NaCl, 2.5 mM KCl, 1 mM MgCl_2_, 1.8 mM CaCl_2_, 1 mM sodium phosphate monobasic, nonessential amino acids, MEM amino acid supplements, pen/strep (P/S), GlutaMAX, sodium bicarbonate (2 g/liter), and 2% FBS] and supplemented with Hoechst 33342 (0.05 μg/ml). Cells were incubated for 2 hours, the test drug was added, and imaging was started immediately using ImageXpress Micro XLS equipped with an environmental chamber. TFEB-EGFP nuclear-to-cytoplasmic signal was calculated as described above.

#### Lysosensor yellow/blue staining (pH)

Cells were plated in opaque black 96-well plates in growth media and allowed to attach. The media were then exchanged for DMEM with glucose (1 g/liter), 2% FBS, P/S, and containing the test compounds. The next day, media were removed and 2 μM lysosensor yellow/blue was added to the imaging solution without FBS for 3 min at RT. The cells were then washed three times in the same buffer and incubated in the same buffer for 12 min before the signal was read using a BMG ClarioStar microplate reader (329-15 nm excitation/540-20 nm emission and 384-15 nm excitation/540-20 nm emission). For estimation of pH, wells on the same plate were incubated in potassium-rich buffer containing the ionophores nigericin and monensin with pH 3.5~5.5 [10 mM NaCl, 135 mM KCl, glucose (1 g/liter), 1 mM CaCl_2_, 1 mM MgCl_2_, 10 μM nigericin, 2 μM monensin, and 20 mM MES] to generate a standard curve. Lysosomal pH was determined from the ratio of light excited at 329 nm over 384 nm and interpolated from the standard curve using GraphPad Prism.

#### In-cell enzyme-linked immunosorbent assay assays

BEAS-2B cells were plated in white 384-well plates (164610, Thermo Fisher Scientific) in complete DMEM at 7500 cells per well, and 293A cells were plated in poly-l-lysine–coated white 384-well plates in complete DMEM at 15,000 cells per well. The plates were incubated at RT for 30~60 min before being transferred to a tissue culture incubator. The next day, test compounds and viral inoculates were prepared in glucose-free DMEM with 2% FBS, l-glutamine, P/S, and Hepes. DMEM with glucose (4.5 g/liter), 2% FBS, l-glutamine, P/S, and Hepes was included for determination of nonspecific background signal. The growth medium was removed from cells by flicking the plate upside down a few times and blotting the remaining liquid onto tissue paper. The cells were then washed in glucose-free DMEM with 2% FBS, pen/strep. This medium was removed as described above. Immediately thereafter, 30 μl of compound solution was added using an Agilent Bravo liquid handler and the cells were transferred to an incubator. The next day, cells were fixed by adding an equal volume of prewarmed 8% PFA/PBS and allowed to sit for 30 min. The cells were washed twice in TBS using a Biotek 405TS automated washer. TBS was aspirated and cells permeabilized by adding 15 μl of TBS/0.5% Triton X-100, for 15 min. Cells were washed three times in TBST. TBST was aspirated, and cells were blocked by adding 15 μl of TBST/1% bovine serum albumin (BSA), for 15~60 min. The blocking solution was aspirated, and 15 μl of the primary antibody solution (OC43, EMDMillipore) in TBST/1% BSA was added. The plates were sealed and incubated at 4°C overnight or 2 hours at RT. Next, the cells were washed four times in TBST, the TBST was aspirated, and 15 μl of secondary antibody solution was added [αRabbit-HRP 1:20,000 (Invitrogen) in TBST/5% dry milk powder]. Plates were sealed and incubated for 1 h at RT. The cells were then washed four times in TBST, the TBST was aspirated and 25 μl of ECL (enhanced chemiluminescent) reagent was added. Twenty minutes later, luminescence was measured using a BMG ClarioStar microplate reader. Sample signal intensity was normalized to the experimentally relevant control and represented as means ± SEM.

#### Kinase RNAi library screening

BEAS-2B cells stably expressing TFEB-EGFP were seeded at a final density of 2000 cells per well in 384-well glass-bottom plates before transfection with the MISSION siRNA Kinase panel (Sigma-Aldrich) using the XtremeGene siRNA transfection reagent (Roche). After 72 hours, cells were fixed in 4% PFA and counterstained with Hoechst 33342, and fluorescence was detected using the Cytation5 High Content Imager (BioTek). Nuclear-to-cytosolic ratio was calculated using the Gen5 software (BioTek).

#### In vitro protein binding assays

Protein binding assays were conducted as previously described (*61*). Briefly, PAK2 or DCAF7 protein was immunoprecipitated from 1 mg of BEAS-2B cell lysate using 1:100 antibody dilution (Cell Signaling and Abcam, respectively) or normal rabbit IgG as a control. Protein was precipitated in IP buffer [50 mM tris-HCl (pH 7.6), 150 mM NaCl, and 0.25% (v/v) Triton X-100] for 4 hours at 4°C and then coupled to protein A/G agarose resin for an additional 2 hours. TFE3, MITF, or TFEB binding constructs and mutants were prepared through PCR cloning for in vitro synthesis using TnT expression kits and allowed to bind to immunoprecipitated PAK2 or DCAF7 overnight. Resin was washed, and protein was eluted in 1x Laemmli buffer at 88°C for 5 min prior to immunoblotting analysis.

#### Molecular docking studies

The docking experiments were carried out using the LibDock program from Discovery Studio 3.5 (BIOVIA). Human DCAF7 protein sequence was first input into Discovery Studio 3.5, and a BLAST search was performed to identify the most homologous proteins. From the BLAST search, the proteins 2XYI.dsv (*62*) was selected to construct the homology model using the “Create Homology Models” function. The specific program parameters were as follows: Protein optimize Sidechains: True, Waters: False, Cut overhangs: True, Refine Loops: True, Refine loops Optimization level: High, Refine loops Use DOPE method: True, Parallel Processing: True. The predicted models were then verified using the “MODELER” function. The selected models were then further optimized using the “Minimization” and “Refinement” functions. Protein cavity and potential drug binding site was determined using “from receptor cavities” function (site 1, *X*: −2.469; *Y*: −0.213; *Z*: 1.232, radius: 28.3). Libdock program parameters were: Number of Hotspots: 100, Docking tolerance: 0.25, Docking Preference: High quality, Conformation method: Fast, Minimization Algorithm: Do not minimize, Parallel Processing: True. A library containing 3 million small molecule compounds (ChemDiv Inc.) was used to screen potential ligands for the domain. After the docking study, the top score-ranking molecules were filtered using Lipinski rule of five and selected for further evaluation. We were able to identify and confirm the initial hit compound BC1753 in vitro and initiated a subsequent structural-activity relationship (SAR) campaign. Last, as confirmation, BC18630 is docked back into the DCAF7 structure using the above program and parameters.

#### Cellular thermal shift assay

Our procedure is based on the previous literature (*93*). Untransfected WT BEAS-2B cells [endogenous cellular thermal shift assay (CETSA)] or HEK cells transfected with DCAF7 plasmid overnight (ectopic CETSA) were treated with vehicle or BC18813 (3 μM for 1 hour). Cells were collected and resuspended in 10 ml of PBS supplemented with EDTA-free protease inhibitor tablet. Cell solutions were aliquoted to 10 PCR microtubes evenly. Using the PCR thermocycler generating temperature gradient, each aliquot was incubated at a certain temperature between 40° and 58°C with 2°C interval for 3 min, then at RT for 3 min. Samples were immediately snap frozen in liquid nitrogen and two cycles of freeze-thaw followed. After brief vortexing, samples were transferred to 1.7-ml microcentrifuge tubes for centrifuging at 20,000*g*, 4°C for 20 min. Supernatants were carefully acquired and used for subsequent immunoblotting analysis.

#### Recombinant protein preparation

DCAF7 recombinant protein was generated using the HaloTag Purification System. Briefly, we cloned full length DCAF7 into pFN18A vector (Promega) and transformed *Escherichia coli* KRX (Promega) with the construct followed by induction and purification using HaloTag Protein Purification System (Promega) and following the manufacturer’s recommendations.

#### In vitro ubiquitination assay

DCAF7-mediated TFEB in vitro ubiquitination assay was conducted as previously described (*94*). TFEB protein with V5-tag was in vitro synthesized through TnT kit (Promega). Synthesized TFEB was either directly incubated with ubiquitination mixture or precipitated from TnT mix via IP-V5 prior to ubiquitination assay. The full complement of ubiquitination constituents was assembled: E1 (100 nM), mixture of E2 (~1 μM), ubiquitin (2.5 μM), magnesium/ATP (5 mM), recombinant human CUL4A/NEDD8/RBX1 (~10 nM) (materials from Enzo and R&D Systems), and recombinant DCAF7 (~4 μM); this assay mixture was incubated with TFEB-V5 protein for 90 min at 30°C. Assay was quenched with 3xPSB and analyzed by SDS-PAGE and immunoblotting for TFEB signal.

#### Viral spreading assay

OC43 infectivity was assayed by fluorescence staining of infected cells quantified by automated microscopy. WT or *DCAF7* KO BEAS-2B cells or vehicle or BC18813 pretreated BEAS-2B cells were inoculated with equal amounts of OC43 (0.03 MOI) for 18 hours, 34°C. Following the initial infection, cells were gently but thoroughly washed (3x fresh media) and replenished with full culture media for an additional 72 hours (34°C). Following the second incubation, equal amounts of media supernatant was removed and titrated onto naïve WT BEAS-2B cells in 96- or 384-well optical plate. Following an additional 72-hour incubation (34°C), infected cells were fixed and processed for immunostaining of OC43 NP protein. Cells were imaged with GE InCell2000, and the proportion of infected cells was calculated through CellProfiler.

#### In vitro kinase assay

PAK2 in vitro kinase assay was conducted with recombinant and active PAK2 (Reaction Biology) and was adapted from the Cell Signaling Technologies protocol. TFEB-EGFP pRK5 was expressed in BEAS-2B cells for 18 hours prior to cell lysis in IP buffer [137 mM NaCl, 2.7 mM KCl, 10.14 mM Na_2_HPO_4_, 1.76 mM KH_2_PO_4_, and 0.25% (v/v) Triton X-100 supplemented with Pierce Protease and Phosphatase inhibitor cocktail], sonication, and precipitation (12,000*g* for 10 min at 4°C). Lysate (400 μl) was incubated with anti-GFP beads (20 μl) (GFP-Trap, Chromotek) for 1 hour and washed three times with 1x TBST. Beads were incubated with ATP (0.5 mM) for 30 min, then mixed with PAK2 enzyme (3 ng/ml) in kinase buffer [final concentration: 25 mM tris-HCl (pH 7.5), 10 mM MgCl_2_, 0.1 mM Na_3_VO_4_, 5 mM β-glyercophosphate, and 2 mM dithiothreitol] for 30 min at 37°C. For the heat-kill control, PAK2 kinase was heated at 99°C for 15 min prior to reaction. Following incubation, the assay was quenched with addition of 3xPSB to reach final 1x concentration. Beads were then heated at 95°C for elution, and the eluate was analyzed by SDS-PAGE and blotting with the Phos-tag Biotin Probe (Fujifilm) chemical system (*95*).

#### Peptide binding assay

TFEB peptide binding assays were conducted similar to previously reported (*61*). Biotinylated peptides were generated at CHI-Scientific with sequences corresponding to TFEB protein region 136 to 145. Four distinct types were generated, including unmodified sequence (I), phosphorylation modification at S138/S142 (II), and substitutions of S138A/S142A (III) and S138D/S142D (IV). Peptide (20 μg) was conjugated to magnetic streptavidin resin (20 μl) for 1 hour in binding buffer [137 mM NaCl, 2.7 mM KCl, 10.14 mM Na_2_HPO_4_, 1.76 mM KH_2_PO_4_, and 0.001% (v/v) Tween 20]. Excess peptide was washed out, and recombinant DCAF7 (50 μg) was incubated with peptide and beads for 4 hours. Alternatively, recombinant DCAF7 was preincubated with BC18630 titration for 1 hour before similar incubation with phosphorylated TFEB peptide (II) and beads. Following final wash, bound protein was eluted from beads, and the eluate was analyzed via immunoblotting.

#### Localized surface plasmon resonance

OpenSPR-XT 2-channel system (Nicoya) was used for surface plasmon resonance assays. Recombinantly derived DCAF7 protein (~12 μM) was immobilized onto to a high-sensitivity carboxyl sensor using EDC/NHS reaction; immobilization response was noted at ~3500 RU. BC18630 dilution series was prepared in running buffer [137 mM NaCl, 2.7 mM KCl, 10.14 mM Na_2_HPO_4_, 1.76 mM KH_2_PO_4_, and 0.001% (v/v) Tween 20] and injected at a flow rate of 40 μl/min with an association time of 300 s and a dissociation time of 600 s. DCAF7-specific signal was corrected by subtraction of reference channel. Resulting data were analyzed with TraceDrawer, and binding kinetics were calculated with a 1:1 Langmuir kinetic analysis modeling.

#### Microscale thermophoresis

For MST experiments, purified DCAF7 was fluorescently labeled with the amine-reactive dye RED-MALEIMIDE 2nd Generation (NanoTemper Technologies) according to the manufacturer’s instructions. After labeling, DCAF7 was diluted in PBS as the assay buffer for MST experiments. The diluted DCAF7 was titrated with a twofold dilution series of compound BC18630 starting at 50 μM prior to the samples being transferred to Monolith NT. Automated premium coated capillary chips (NanoTemper Technologies). MST traces were recorded at RT in a Monolith NT. Automated using the MO.Control software (LED excitation power setting 20%, medium MST power). The initial fluorescence intensity data were analyzed using MO.Affinity Analysis software.

#### Nano differential scanning fluorimetry

The Prometheus NT.48 instrument (NanoTemper Technologies) was used to monitor the DCAF7 melting temperatures with various drug concentrations. The capillary cassette was filled with a 10-μl sample and placed on the sample holder. A temperature gradient of 2°C/min from 25° to 95°C was applied, and the intrinsic protein fluorescence at 330 and 350 nm was recorded. The ratio of the recorded emission intensities (Em350nm/Em330nm), which represents the change in tryptophan fluorescence intensity as well as the shift of the emission, was plotted as a function of the temperature. The first derivatives of fluorescence intensity ratio were calculated with the manufacturer’s software (PR.ThermControl). Three replicates were carried out for each condition, and their mean is calculated, exported and graphed in Prism (*96*).

#### Proteasome activity assay

Proteasomal activity assay was conducted with ProteasomeGlo kit (Promega), according to the manufacturer’s instructions and previous methods (*97*). Briefly, BEAS-2B cells were seeded in a 384-well plate to a density of 4000 cells per well and treated with dose course of BC18813 or BC18630 with several wells treated with carfilzomib (250 nM) as a control. Following 18-hour treatment, cells were collected and incubated with ProteasomeGlo lysis buffer and reagent along with luminescent substrates Suc-LLVY-Glo, Z-LRR-Glo, and Z-nLPnLD-Glo for 10 min before luminescence reading on ClarioStar (BMG) to determine proteasome protease activity.

#### HiBiT nano-luciferase stability assay

SARS-CoV-2 viral proteins were cloned into the pFC37K-HiBiT split nano-luciferase system using PCR-based cloning (Promega). All sequences were confirmed by DNA sequencing (Genewiz). Equivalent amount of plasmid was transfected to BEAS-2B cells for 18 hours followed by 24 hours of treatment with BC18813 at indicated concentrations or combination of BC18813 and leupeptin (2 μM) or BFA (0.2 μM). Following treatment, cells were collected and lysed for SDS-PAGE as described above, and the resultant blot was processed for Nano-Glo HiBiT blotting (Promega), according to the manufacturer’s instructions (*98*).

#### Proximity ligation assay

The TFEB interaction with DCAF7 was measured by a PLA using Duolink PLA kit (Sigma-Aldrich). Briefly, BEAS-2B cells were seeded in 384-well glass-bottom plates and treated with increasing concentrations of a DCAF7 inhibitor compound or OC43 virus at indicated doses with carfilzomib cotreatment (240 nM). Following treatment, cells were fixed in 4% PFA, permeabilized, blocked, and incubated with an anti-DCAF7 (Thermo Fisher Scientific) and anti-TFEB (Santa Cruz Biotechnology) antibody overnight. Cells were washed and incubated with PLA secondary antibody, ligase, polymerase, fluorescent dye, and counterstained with Hoechst 33342. The PLA signal was visualized with Leica SP8 confocal, and the PLA signal per cell was calculated using Cell Profiler.

#### Mouse lung viral titer

Murine lungs from the K18-hACE2 SARS-CoV-2 infection were collected from euthanized mice, halved, and frozen in 500 μl of PBS, bead beat for 60 s, and then 100 μl was used for titer using the plaque assay as previously described (*99*).

#### Histology and IHC

Following euthanasia, hamster lung samples were excised and immediately fixed in 10% formalin for 24 hours. Lung tissue was dehydrated with ethanol before embedding in paraffin to generate tissue blocks. Histological staining was conducted at the McGowen Transplant Institute at the University of Pittsburgh. Samples were sectioned using a microtome and processed for staining. Slides were stained for hematoxylin and eosin (H&E) or for IHC of the SARS-CoV-2 NP (Invitrogen, ma17403) or of TFEB (Bethyl Laboratories, A303-673A) at 1:100 dilution following the manufacturer’s instructions. Stained lung slides were visualized using a Cytation5 microscope (BioTek), and representative images were captured. Immunohistochemical quantification was conducted as previously described (*100*). Briefly, image fields were chosen from each condition and RGB files were deconvoluted using ImageJ H-DAB algorithm (*92*). Hematoxylin and DAB signal was thresholded; hematoxylin signal was used to estimate cell count, and DAB mean gray value was calculated. DAB mean gray value per cell number was calculated for each field, and ratios for each treatment normalized to mean vehicle were reported.

#### Synthetic procedure for BC18813 and BC18630

##### *N*-(7-fluoro-1*H*-indol-5-yl)-2-pyridin-4-ylpyrido[2,3-*d*]pyrimidin-4-amine hydrochloride (BC18813) (fig. S12E)

Synthesis of 7-fluoro-1*H*-indol-5-amine (fig. S13A)

Reagents and conditions: (i) KO*t*-Bu, 1-amino-1,3,4-triazole, dimethyl sulfoxide (DMSO), ambient temperature; (ii) NIS, AcOH, ambient temperature; (iii) TMSA, CuI, Pd(PPh_3_)_4_, triethylamine (TEA), tetrahydrofuran (THF), ambient temperature; (iv) K_2_CO_3_, MeOH, ambient temperature; (v) KO*t*-Bu, N-Methyl-2-pyrrolidone (NMP), 50°C; (vi) H_2_, 10% Pd/C, EtOH, ambient temperature.

Step 1. Synthesis of compound 2.

A solution of *m*-fluoro-nitrobenzene 1 (15.4 g, 0.11 mol) and 1-amino-1,3,4-triazole (9.2 g, 0.11 mol) in 100 ml of DMSO was added dropwise to a stirred solution of potassium *tert*-butoxide (12.3 g, 0.11 mol) in 200 ml of DMSO. The reaction mixture was stirred overnight at ambient temperature, quenched with 600 ml of saturated aqueous solution of ammonium chloride, and extracted twice with ether. The combined organic layers were washed with water, dried over magnesium sulfate, and concentrated. The residue was subjected to silica column chromatography eluting with hexane/EtOAc to afford compound 2 (3.3 g, 19%).

Step 2. Synthesis of compound 3.

*N*-Iodosuccinimide (5.24 g, 23.3 mmol) was added portionwise to a stirred solution of compound 2 (3.3 g, 21.1 mmol) in 30 ml of acetic acid. The resulting mixture was stirred overnight at ambient temperature and poured into ice-cold water. The formed precipitate was filtered off, washed thoroughly with water, and dried to afford compound 3 (5.47 g, 92%).

Step 3. Synthesis of compound 4.

Compound 3 (5.47 g, 19.4 mmol) and TEA (5.38 ml, 38.8 mmol) were dissolved in 30 ml of THF. The reaction vessel was evacuated and backfilled with argon several times. CuI (369 mg, 1.94 mmol) and Pd (PPh_3_)_4_ (1.12 g, 0.97 mmol) were added followed by addition of trimethylsilylacetylene (3.22 ml, 23.3 mmol). The reaction mixture was stirred overnight at ambient temperature, filtered through a Celite pad, and evaporated to dryness under reduced pressure. The residue was subjected to silica column chromatography eluting with hexane/dichloromethane (DCM) to afford compound 4 (3.7 g, 76%).

Step 4. Synthesis of compound 5.

Potassium carbonate (1.48 g, 10.7 mmol) was added to a stirred solution of compound 4 (2.7 g, 10.7 mmol) in 50 ml of MeOH. The resulting mixture was stirred at ambient temperature until reaction completion [thin-layer chromatography (TLC) monitoring], filtered through Celite pad, and evaporated to dryness under reduced pressure. Obtained crude compound 5 was used for the next step without further purification.

Step 5. Synthesis of compound 6.

Potassium *tert*-butoxide (2.39 g, 21.3 mmol) was added to a stirred solution of the crude product 5 obtained at the previous step in 30 ml of NMP. The resulting mixture was stirred at 50°C overnight, diluted with EtOAc, and washed three times with water. The combined organic layers were concentrated under reduced pressure, and the residue was subjected to silica column chromatography eluting with hexane/DCM to afford compound 6 (1.57 g, 81% over two steps).

Step 5. Synthesis of compound 7.

A mixture of compound 6 (1.57 g, 8.7 mmol), 10% Pd on charcoal (0.15 g), and EtOH was stirred vigorously in in a hydrogen atmosphere until reaction completion (TLC monitoring), filtered through Celite pad, and evaporated to dryness under reduced pressure to afford (1.24 g, 95%) of the crude product 7 pure enough to be used at the next step.

Synthesis of *N*-(7-fluoro-1*H*-indol-5-yl)-2-pyridin-4-ylpyrido[2,3-*d*]pyrimidin-4-amine hydrochloride (BC18813) (fig. S13B).

Reagents and conditions: (i) DMF, HOBT, EDC·HCl, NH_4_Cl, Et_3_N,12 hours, RT; (ii) 4-pyridinecaboxaldehyde, DMSO, 100°C, overnight; (iii) POCl_3_, PCl_5_, reflux, 12 hours, (iv) aniline A, K_2_CO_3_, DMF, RT, overnight, (v) HCl.

Step 1. Synthesis of compound 9.

A mixture of 2-aminonicotinic acid (11.04 g, 80.0 mmol), HOBT (10.8 g 80.0 mmol), EDC·HCl (11.47 g, 120.0 mmol), Et_3_N (16.16 g, 160.0 mmol), and DMF (200 ml) was stirred overnight at ambient temperature and then concentrated under reduced pressure. The residue was partitioned between DCM (300 ml) and saturated aqueous solution of NaHCO_3_ (300 ml). The organic layer was separated, and the aqueous one extracted with DCM. The combined organic layers were dried over Na_2_SO_4_ and concentrated under reduced pressure. The residue was subjected to silica column chromatography eluting with EtOAc to afford compound 9 (9.27 g, 84%).

Step 2. Synthesis of compound 11.

A mixture of anthranilamide 9 (4.5 g, 32.8 mmol), 4-pyridinecarboxaldehyde (3.86 g, 36.1 mmol), and DMSO (50 ml) was stirred at 100°C for 16 hours, cooled to ambient temperature, and diluted with water (200 ml). Formed precipitate was collected by filtration, washed with water, and dried to afford compound 11 (4.3 g, 58%).

Step 3. Synthesis of compound 12.

A mixture of the compound 11 (2.55 g, 11.37 mmol), PCl_5_ (2.61 g, 12.5 mmol), and POCl_3_ (52.35 g, 341.0 mmol) was stirred and heated under reflux for 6 hours, cooled to ambient temperature, and poured into ice. The obtained solution was neutralized with saturated aqueous solution of NaHCO_3_ and extracted twice with EtOAc. Combined organic layers were washed with water, dried over Na_2_SO_4_, and concentrated under reduced pressure to afford compound 12 (2.60 g, 94%) pure enough to be used at the next step without further purification.

Step 4. Synthesis of compound 13.

A mixture of 7-fluoro-1*H*-indol-5-amine 7 (1.5 g, 9.98 mmol), compound 12 (2.42 g, 9.98 mmol), K_2_CO_3_ (2.06 g, 14.98 mmol), and DMF (10 ml) was stirred at RT overnight and poured into water. Formed precipitate was filtered off, washed with water, dried, and subjected to HPLC purification to afford compound 13 (1.53 g, 45%).

Step 5. Synthesis of compound 14 (BC18813).

A 3 M solution of HCl (1.5 ml) in ether was added to a stirred mixture of compound 13 (0.88 g; 2.25 mmol) and EtOH (20 ml). The mixture was vigorously stirred at ambient temperature for 15 min. The precipitate was filtered off, washed with acetone, and dried to afford target compound 14 (0.92 g, 99%).

^1^H nuclear magnetic resonance (NMR) (400.4 MHz, DMSO-d_6_, δ): 9,11–9.18 (m, 2H); 8.98 (d, *J* = 5.9 Hz, 2H); 8.66 (d, *J* = 5.9 Hz, 2H); 7.75–7,84 (m, 2H); 7.42–7.49 (m, 2H), 6.62 (t, *J* = 2.9 Hz). MS [electrospray ionization (ESI)]: mass/charge ratio (*m/z*) 357.4 [M + H]^+^.

##### *N*-1*H*-indol-5-yl-2-pyridin-4-ylquinazolin-4-amine hydrochloride (BC18630) (fig. S12E)

Synthesis of *N*-1*H*-indol-5-yl-2-pyridin-4-ylquinazolin-4-amine hydrochloride (BC18630) (fig. S13C).

Reagents and conditions: (i) 4-pyridinecaboxaldehyde, DMSO, 100°C, overnight; (ii) POCl_3_, PCl_5_, reflux, 12 hours; (iii) aniline A, K_2_CO_3_, DMF, RT, overnight; (iv) HCl.

Step 1. Synthesis of compound 2.

A mixture of anthranilamide 1 (1.4 g, 10.35 mmol), 4-pyridinecarboxaldehyde (1.22 g, 11.39 mmol), and DMSO (25 ml) was stirred at 100°C for 16 hours, cooled to ambient temperature, and poured into ice-cold water (200 ml). The formed precipitate was collected by filtration, washed with water, and dried to provide compound 2 (1.93 g, 84%).

Step 2. Synthesis of compound 3.

The mixture of the compound 2 (1.57 g, 7.03 mmol), PCl_5_ (1.61 g, 7.73 mmol), and POCl_3_ (32.4 g, 210.9 mmol) was stirred and heated under reflux for 6 hours, cooled to ambient temperature, and poured into ice. The resulting mixture was neutralized with saturated aqueous solution of NaHCO_3_ and extracted twice with EtOAc. Combined organic layers were washed with water, dried over Na_2_SO_4_, and concentrated under reduced pressure to afford compound 3 (1.96 g, 83%) that was used for the next step without further purification.

Step 3. Synthesis of compound 4.

A mixture of indol-5-amine A (1.07 g, 8.10 mmol), compound 3 (1.96 g, 8.10 mmol), K_2_CO_3_ (1.35 g, 9.72 mmol), and DMF (10 ml) was stirred at ambient temperature overnight and poured into ice-cold water. The formed precipitate was filtered off, washed with water, dried, and subjected to HPLC purification to afford compound 4 (0.94 g, 40%).

Step 5. Synthesis of compound 5 (BC18630).

A 3 M solution of HCl (1.7 ml) in ether was added to a stirred mixture of compound 4 (0.94 g; 2.28 mmol) and EtOH (20 ml). The mixture was vigorously stirred at ambient temperature for 15 min. The precipitate was filtered off, washed with acetone, dried, and dissolved in a minimum amount of water. Obtained solution was lyophilized to afford target compound 5 (1 g, 96%).

^1^H NMR (400.4 MHz, DMSO-d_6_, δ): 11.28 (br. s, 1H); 10.74 (br.s, 1H); 9.0 (d, *J* = 5.4 Hz, 2H); 8.78 (d, *J* = 7.8 Hz, 1H); 8.63 (d, *J* = 5.4 Hz, 2H); 8.10 (d, *J* = 8.3 Hz, 1H); 8.07–8.13 (m, 1H); 7.91–7.94 (br. m, 1H); 7.76–7.82 (m, 1H); 7.51–7.54 (br. m, 2H); 7.42 (t, *J* = 2.9 Hz). MS (ESI): *m/z* 338.2 [M + H]^+^.

### Quantification and statistical analysis

Statistical comparisons were performed in GraphPad Prism 9. All statistical details of experiments can be found in the figure legend. Unpaired two-tailed Student’s *t* test was used to compare two groups. Comparisons of more than two groups were tested with one-way or two-way analysis of variance (ANOVA) with post hoc test of multiple comparisons. Significance was determined by *P* < 0.05 or greater and is indicated in figure legends.

### Data and software availability

The published article includes all datasets generated or analyzed during this study.

## Supplementary Materials

### This PDF file includes:

Figs. S1 to S32 Tables S1 to S4 Uncropped blots References
