# Supplementary material for: Inhibition of virally induced TFEB proteasomal degradation as a host-centric therapeutic approach for coronaviral infection
Source: Sci Adv. 2025 Jun 4;11(23):eadv4033. doi: 10.1126/sciadv.adv4033 (PMC12136031; doi:10.1126/sciadv.adv4033)
Supplement: Supplementary file 1 — Figs. S1 to S32 Tables S1 to S4 Uncropped blots References [file sciadv.adv4033_sm.pdf]

Science Advances
AAAS

Supplementary Materials for

Inhibition of virally induced TFEB proteasomal degradation as a host-centric therapeutic approach for coronaviral infection

Travis B. Lear *et al.*

Corresponding author: Yuan Liu, yul119@pitt.edu; Bill B. Chen, chenb@upmc.edu; Toren Finkel, finkelt@pitt.edu

Sci. Adv. **11**, eadv4033 (2025)
DOI: 10.1126/sciadv.adv4033

This PDF file includes:

Figs. S1 to S32
Tables S1 to S4
Uncropped blots
References

Supplemental Figures:

Figure S1

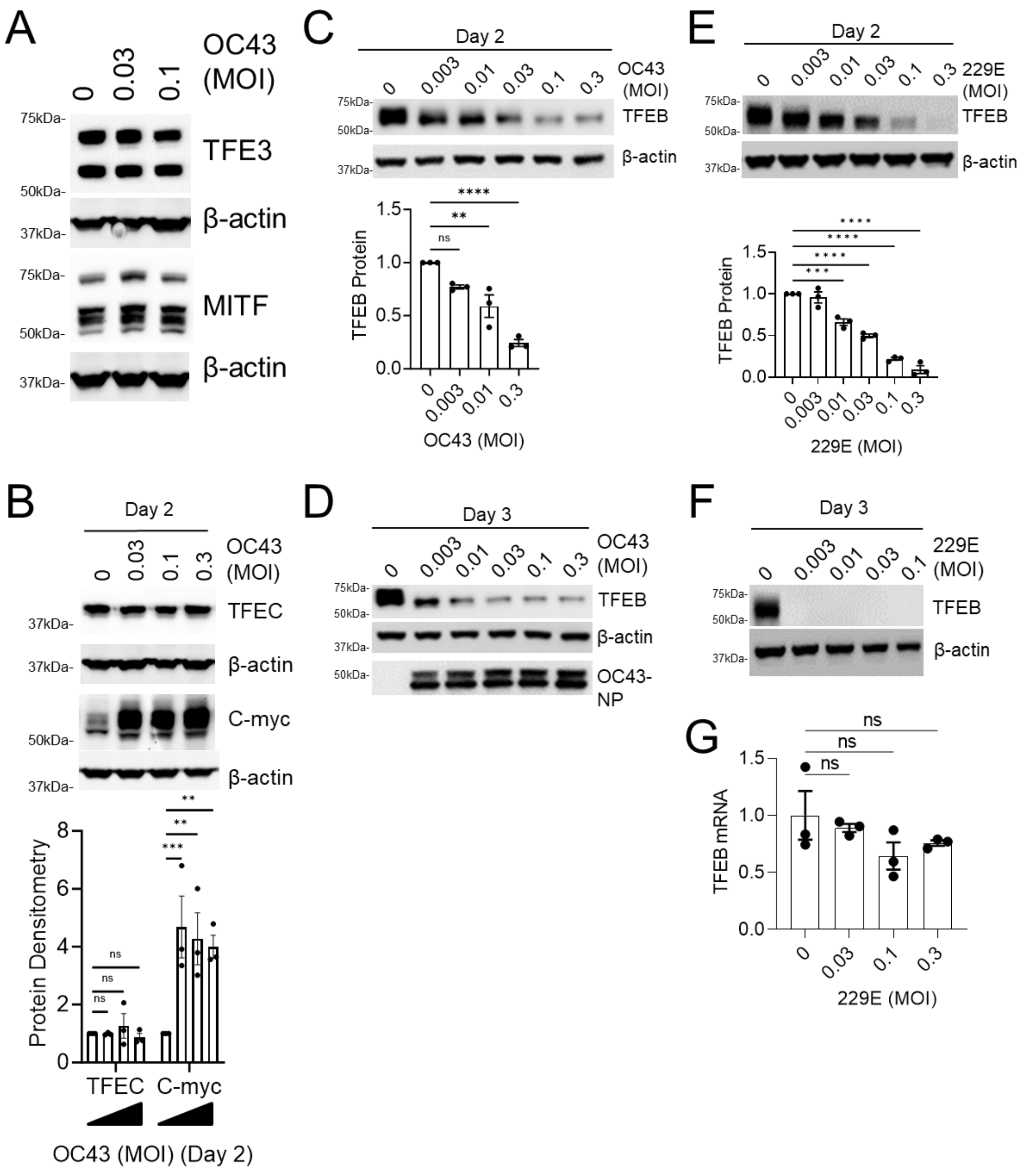

Supplementary Figure 1. Viral infection selectively reduces TFEB protein. A-B. Immunoblot analysis of TFEB homologues from BEAS-2B cell lysate following OC43 infection (48 h) at indicated MOI. Densitometry of protein blots shown in Fig. 1C or below. Data represent mean ± SEM (n=3). **C-F.** Immunoblot analysis of human bronchial airway BEAS-2B cells infected with *beta*-coronavirus OC43 (C-D) or human lung fibroblast MRC-5 cells infected with *alpha*-coronavirus 229E (E-F) at the indicated MOI at (48 or 72 h post inoculation). TFEB protein densitometry was quantified; data represent mean ± SEM (n=3). **G**. qPCR analysis of TFEB mRNA from MRC-5 cells treated with increasing MOI of 229E (48 h). Data represent a fold change in TFEB mRNA levels relative to MOI=0 condition; mean ±SEM (n=3). * $p<0.05$; *** $p<0.001$; **** $p<0.0001$; compared to control or as indicated by two-way ANOVA with Tukey's multiple comparisons (B) or one-way ANOVA with Dunnett's multiple comparisons (C, E, G).

Figure S2

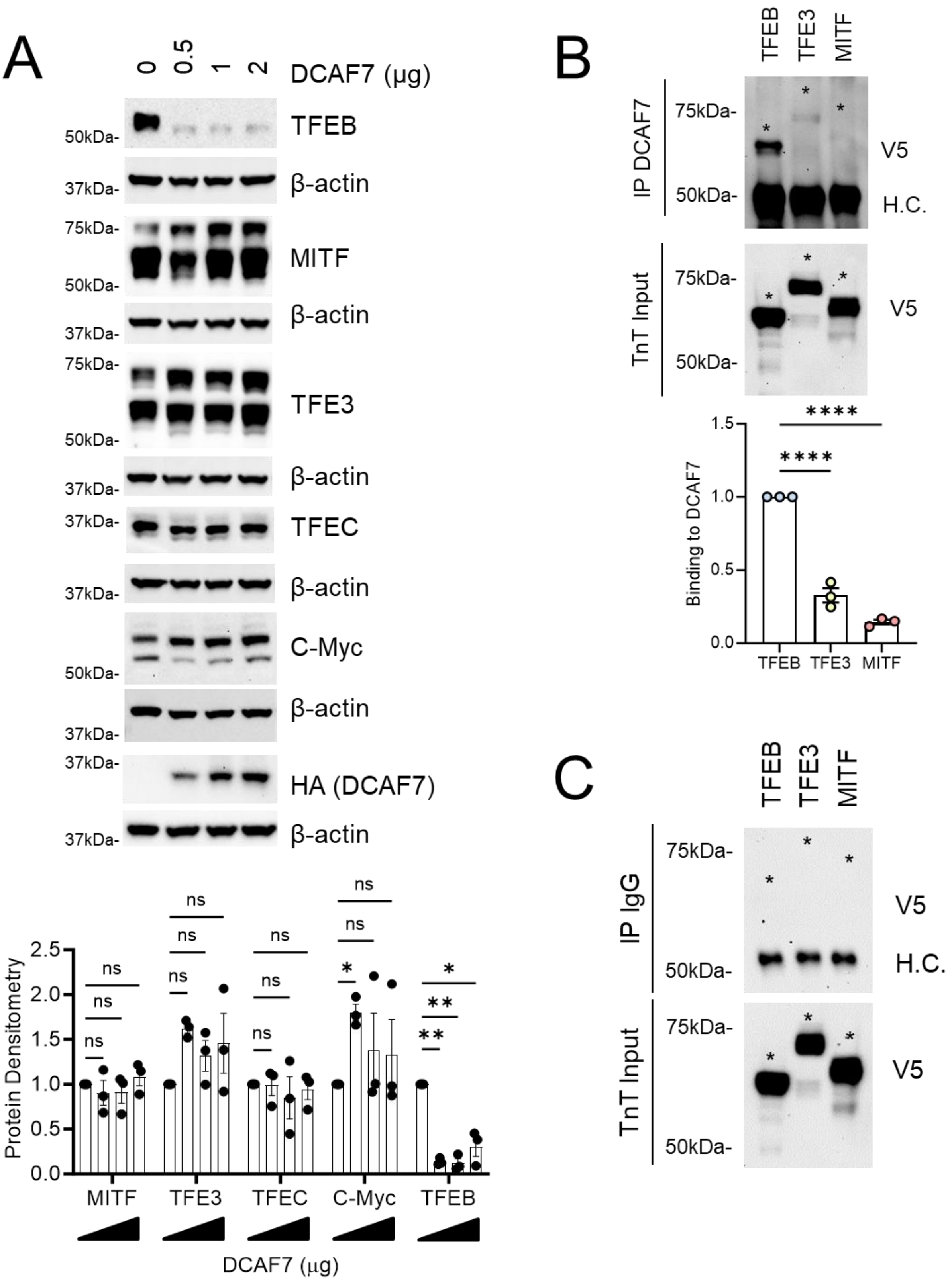
A
0
0.5
1
2
DCAF7 (µg)
TFEB
β-actin
MITF
TFE3
TFEC
C-Myc
HA (DCAF7)
50kDa-
37kDa-
75kDa-
Protein Densitometry
MITF
TFE3
TFEC
C-Myc
TFEB
DCAF7 (µg)
ns
B
TFEB
TFE3
MITF
IP DCAF7
TnT Input
V5
H.C.
Binding to DCAF7
****
C
IP IgG
TnT Input
V5
H.C.

Supplementary Figure 2. TFEB protein is a preferential target of DCAF7 among MiT family proteins. A. Immunoblot analysis of MiT family proteins in BEAS-2B cells transfected with increasing amounts of DCAF7. Densitometry of proteins was calculated and normalized below. Data represent mean ± SEM (n=3). **B.** Protein binding assay between DCAF7 and TFEB and its homologues. DCAF7 preferentially binds TFEB protein, densitometry below. Data represent mean ± SEM (n=3). **C.** Protein binding assay using IgG as a negative control, demonstrating the absence of non-selective binding by TFEB and related homologues to IP or beads. NS $p>0.05$; * $p<0.05$; ** $p<0.01$; *** $p<0.001$; **** $p<0.0001$; compared to control or as indicated or two-way ANOVA with Tukey's multiple comparisons (A) or one-way ANOVA with Dunnett's multiple comparisons (B).

Figure S3

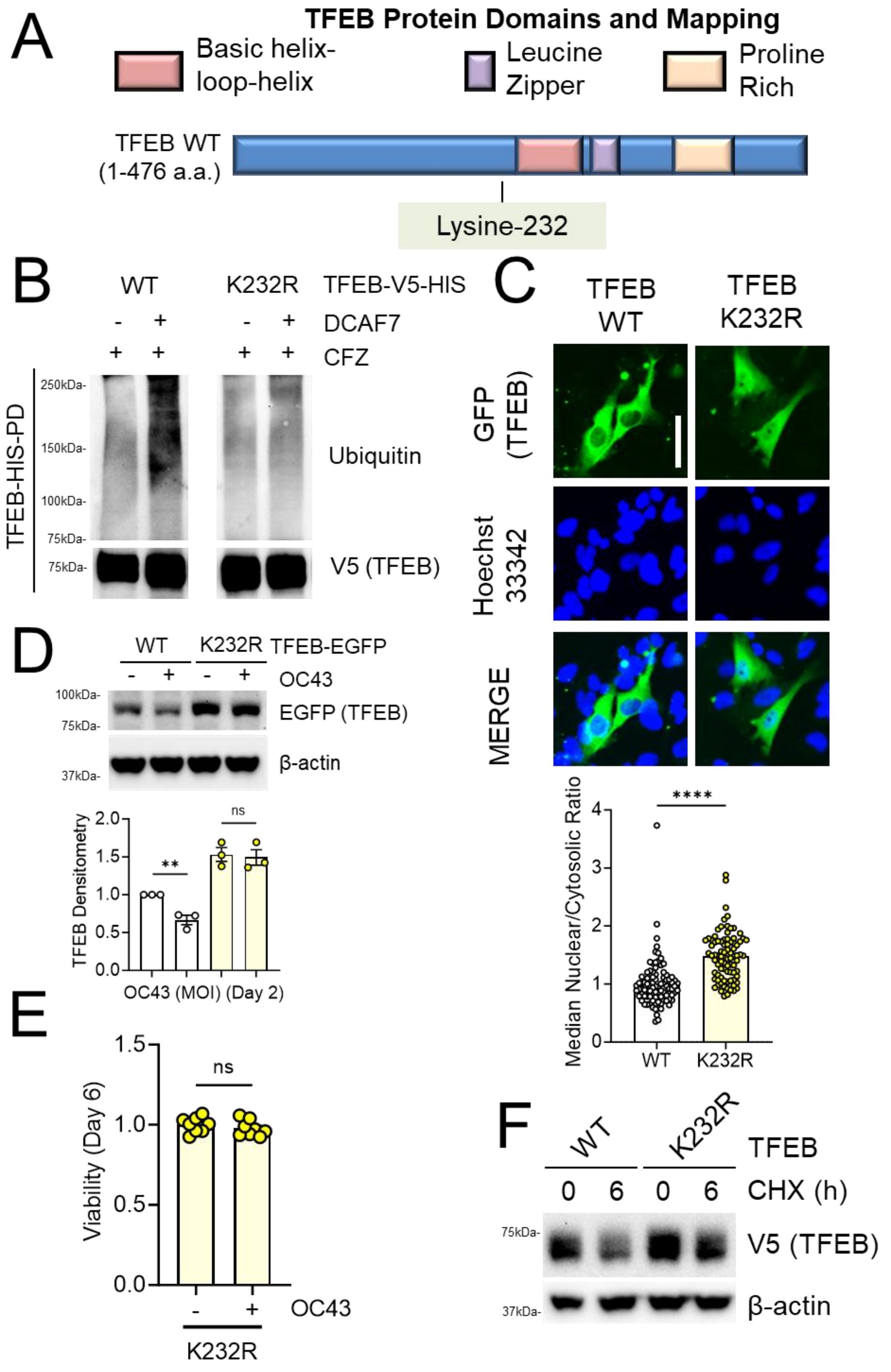

A
TFEB Protein Domains and Mapping
Basic helix-loop-helix
Leucine Zipper
Proline Rich
TFEB WT (1-476 a.a.)
Lysine-232
B
WT
K232R
TFEB-V5-HIS
DCAF7
CFZ
TFEB-HIS-PD
250kDa-
150kDa-
100kDa-
75kDa-
Ubiquitin
V5 (TFEB)
C
TFEB WT
TFEB K232R
GFP (TFEB)
Hoechst 33342
MERGE
Median Nuclear/Cytosolic Ratio
****
WT
K232R
D
TFEB-EGFP
OC43
EGFP (TFEB)
β-actin
100kDa-
75kDa-
37kDa-
TFEB Densitometry
**
ns
OC43 (MOI) (Day 2)
E
Viability (Day 6)
ns
OC43
K232R
F
TFEB
CHX (h)
V5 (TFEB)
β-actin

Supplementary Figure 3. Lys-232 is a putative ubiquitin acceptor site within TFEB. A. Schematic of TFEB protein domains. Lysine-232 is a putative ubiquitination site. **B.** TFEB protein pulldown in BEAS-2B cells co-transfected with DCAF7 and WT TFEB or TFEB K232R mutant. Cells were treated with CFZ and ubiquitin signal was detected by immunoblotting. **C.** Immunofluorescent microscopy of BEAS-2B cells expressing EGFP-tagged WT TFEB or K232R mutant TFEB. The ratio of nuclear/cytosolic TFEB signal was quantified, data represent median ratio of a well's population of cells ± SEM (n=85-91 fields). Scale bar indicates 50 μm. **D.** Immunoblot assay of either WT or K232R mutant TFEB prior to OC43 infection (0.1 MOI, 48 h). TFEB protein densitometry was quantified, data are mean ± SEM (n=3). **E.** Viability of BEAS-2B cells expressing TFEB-K232R and treated with control or OC43 (0.3 MOI) for 72 h. Data are mean ± SEM (n=8). **F.** Immunoblot analysis of TFEB WT and K232R expressed in BEAS-2B cells and treated with cycloheximide (CHX) demonstrating improved protein stability of the K232R mutant. NS $p<0.05$; ** $p<0.01$; **** $p<0.0001$; compared to control or as noted by unpaired two-sided t-test (C-E).

Figure S4

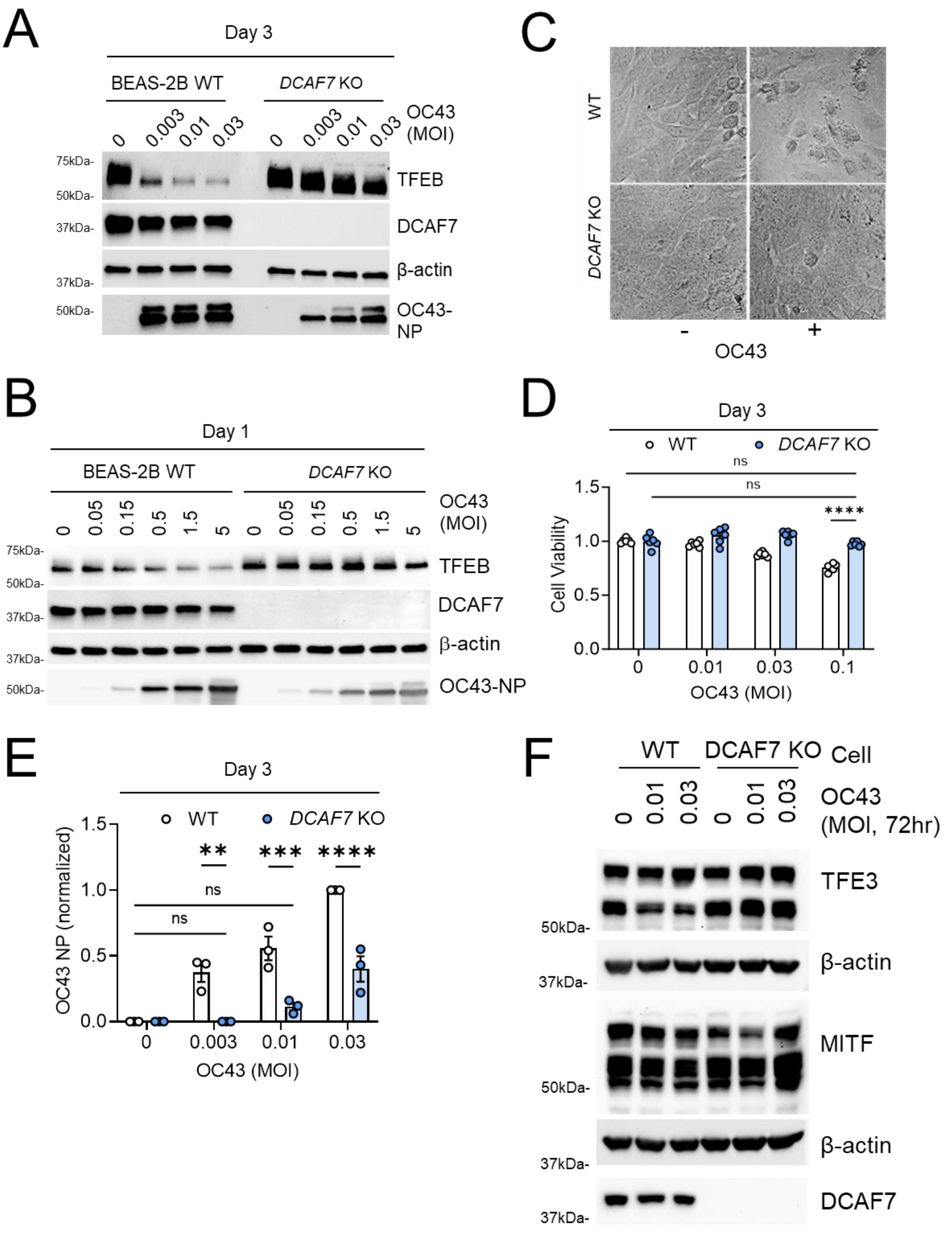

Supplementary Figure 4. DCAF7 deletion prevents viral-induced TFEB degradation and infection. A-B. Immunoblot analysis of WT or *DCAF7* KO BEAS-2B cells treated with the indicated MOI of OC43 and assessed at 3 dpi (A) or 1 dpi (B). **C.** Representative phase microscopy for WT or *DCAF7* KO BEAS-2B cells in the presence (+) or

absence (-) of OC43 infection (0.3 MOI, 72 h post infection). **D.** Quantification of cell viability at day 3 post OC43 infection. Data from CellTiterGlo2.0 readings were normalized to WT MOI=0, and represent mean ± SEM (n=6). **E.** In-cell ELISA analysis of WT or *DCAF7* KO BEAS-2B cells treated with increasing MOI of OC43 with subsequent detection of OC43 Nucleoprotein (NP) levels (72 h). Data were normalized to WT MOI=0.03 NP levels and represent mean ± SEM (n=6). **F.** Immunoblotting of TFEB homologues TFE3 and MITF in WT and DCAF7 KO BEAS-2B treated with dose course of OC43 for 72 h. NS $p>0.05$; * $p<0.05$; ** $p<0.01$; *** $p<0.001$; **** $p<0.0001$; compared to vehicle or control or as indicated by two-way ANOVA with Tukey's multiple comparisons (D-E).

Figure S5

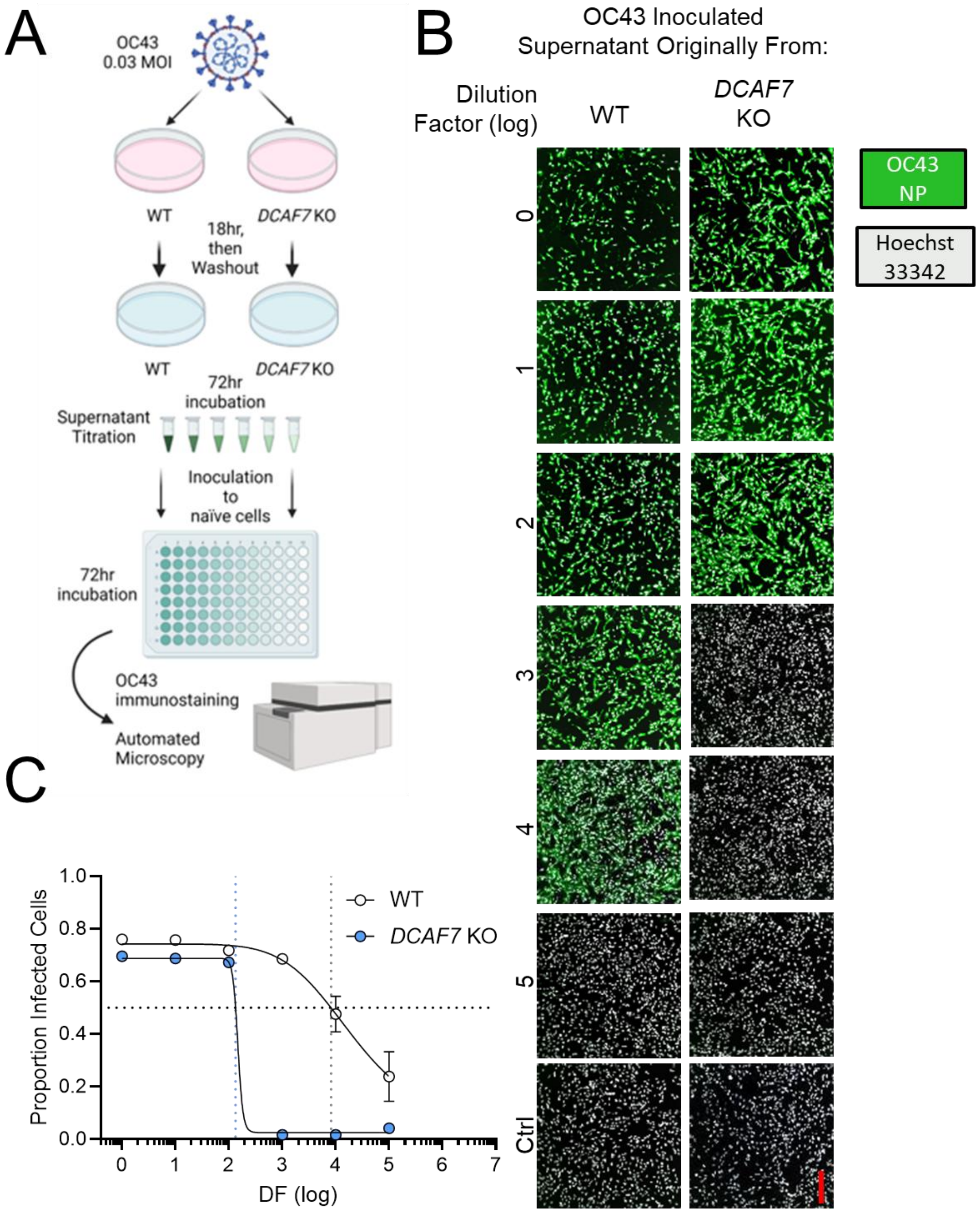
A
OC43
0.03 MOI
WT
DCAF7 KO
18hr, then Washout
WT
DCAF7 KO
72hr incubation
Supernatant Titration
Inoculation to naïve cells
72hr incubation
OC43 immunostaining
Automated Microscopy
B
OC43 Inoculated Supernatant Originally From:
Dilution Factor (log)
WT
DCAF7 KO
0
1
2
3
4
5
Ctrl
OC43 NP
Hoechst 33342
C
Proportion Infected Cells
1.0
0.8
0.6
0.4
0.2
0.0
0
1
2
3
4
5
6
7
DF (log)
WT
DCAF7 KO

Supplementary Figure 5. DCAF7 deletion reduces coronaviral infectivity. A. Schematic of infectivity assay, created with BioRender. WT or *DCAF7* KO BEAS-2B cells were inoculated with equal amounts of OC43 (0.03 MOI) for 18 h prior to washing and re-incubation in fresh media. Following 72 h of incubation in fresh media, equal amounts of supernatant were removed and diluted in a titration series prior to inoculation to naïve WT BEAS-2B cells for another 72 h of infection. Following incubation, cells were fixed and processed for OC43 NP immunostaining and automated microscopy. The percentage of infected cells per well was calculated with CellProfiler. **B.** Representative images from *in vitro* infectivity assay of supernatants stemming from OC43 infected WT or *DCAF7* KO BEAS-2B cells. Scale bar = 300 µm. **C.** Quantification of infected cells from supernatants stemming from OC43 infected WT or *DCAF7* KO BEAS-2B cells. Data represent mean ± SEM (n=12 biological replicates per treatment/dose).

Figure S6

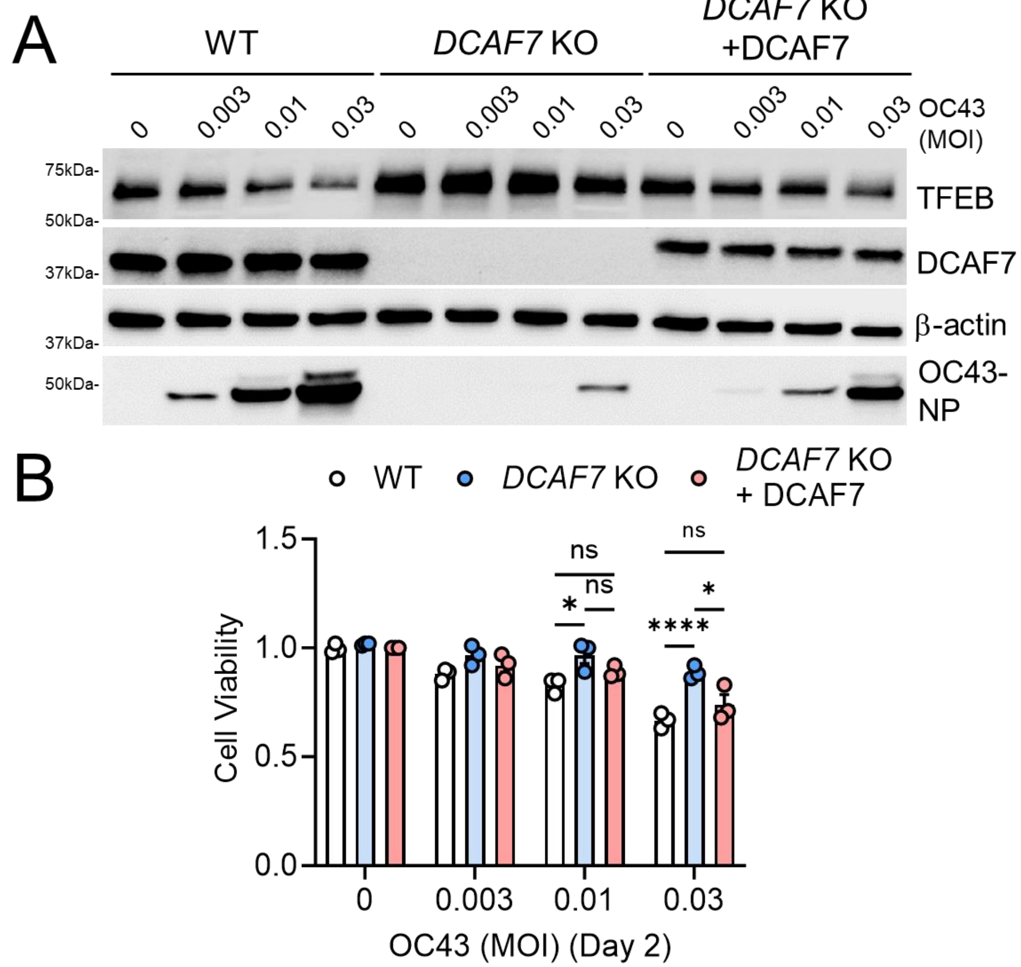

Supplementary Figure 6. Reconstitution of DCAF7 restores viral susceptibility in *DCAF7* KO cells. A. Immunoblot analysis of BEAS-2B WT, *DCAF7* KO, or reconstituted *DCAF7* KO cells treated with the indicated MOI of OC43 (48 h). **B.** Quantification of viability in WT, *DCAF7* KO, or reconstituted *DCAF7* KO cells following OC43 infection as measured with CellTiterGlo2.0. Data were normalized to individual cell MOI = 0 and represent mean ± SEM (n= 3). NS p>0.05; * p<0.05; **** p<0.0001; as compared to vehicle or control or as indicated by two-way ANOVA with Tukey's multiple comparisons (B).

Figure S7

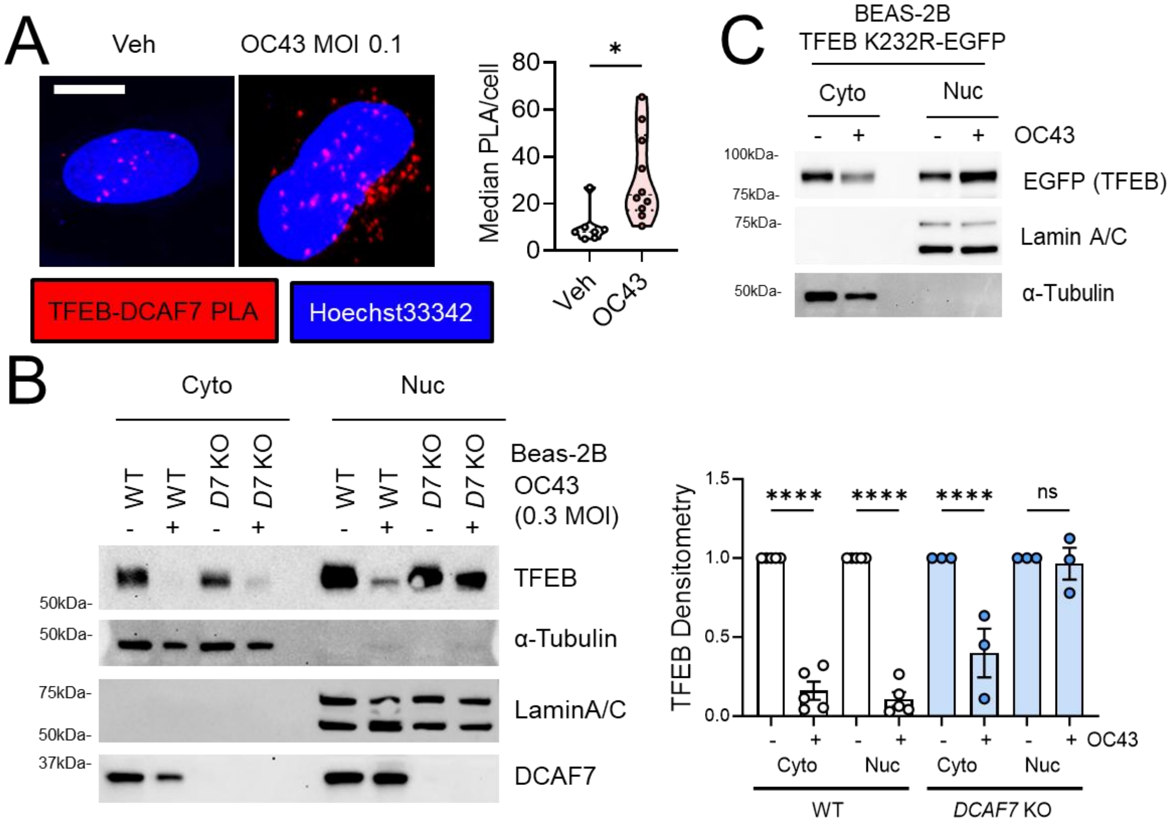

Supplementary Figure 7. TFEB protein is degraded in the nucleus in a DCAF7 dependent manner. A. Immunofluorescent microscopy of TFEB and DCAF7 Proximity Ligation Assay (PLA). BEAS-2B cells were infected with OC43 (0.1 MOI, 24 h). Data represent median PLA signal per cell and interquartile range in violin plots (n=7-10 fields). Scale bar indicates 10 µm. **B.** Immunoblotting of cytosolic and nuclear fractions from WT or *DCAF7* KO BEAS-2B cells infected with OC43 (0 or 0.3 MOI, 72 h). TFEB protein densitometry was quantified and normalized to MOI = 0, data represent mean ± SEM (n=3-5). **C.** Immunoblot analysis of cytosolic and nuclear fractions from BEAS-2B cells expressing TFEB K232R, and infected with OC43 (0.3 MOI, 72 h). NS $p>0.05$; * $p<0.05$; **** $p<0.001$, by two-sided t-test (A) or one-way ANOVA with Tukey's multiple comparisons (B).

Figure S8

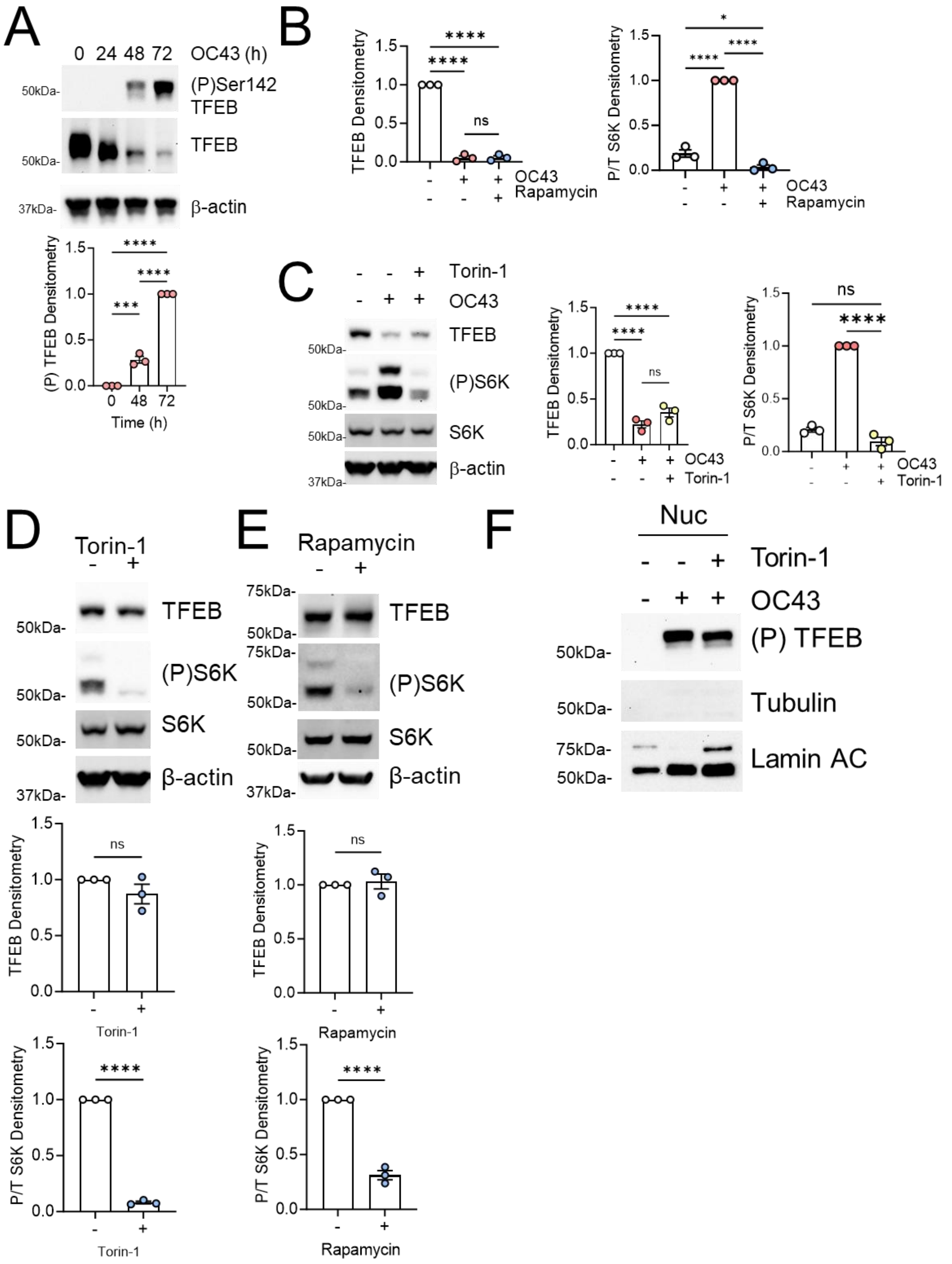
A
0 24 48 72 OC43 (h)
(P)Ser142 TFEB
TFEB
β-actin
50kDa-
37kDa-
(P) TFEB Densitometry
Time (h)
B
TFEB Densitometry
P/T S6K Densitometry
OC43
Rapamycin
C
Torin-1
OC43
TFEB
(P)S6K
S6K
β-actin
D
Torin-1
E
Rapamycin
F
Nuc
(P) TFEB
Tubulin
Lamin AC
75kDa-
ns

Supplementary Figure 8. TFEB phosphorylation increases upon viral infection in the nucleus independent of mTOR activity. A. Immunoblotting of phosphorylated TFEB (Ser-142) following time course of OC43 viral infection (0.03 MOI). Phosphorylated TFEB densitometry was quantified and normalized to 72 h, data represent mean ± SEM (n=3). **B.** Quantification of total TFEB and phospho-T389 S6K densitometry from Fig. 2B. Data are mean ± SEM (n=3). **B.** Immunoblot analysis of TFEB protein level following viral infection (0.03 MOI, 72 h) without and with mTOR inhibition by Torin-1 (10 nM). TFEB levels are not rescued by mTOR inhibition. TFEB and Phospho-S6K protein densitometry was calculated, data represent mean ± SEM (n=3). **D-E.** TFEB protein level was measured following mTOR inhibition with Torin-1 (10 nM, 72 h) (D) or rapamycin (10 nM, 72 h) (E) of cells at baseline uninfected conditions. TFEB and Phospho-S6K protein densitometry was calculated, data represent mean ± SEM (n=3). **F.** Immunoblotting of cytosolic and nuclear fractions from BEAS-2B cells infected with OC43 (0.3 MOI, 72 h) and with concurrent mTOR inhibition (Torin-1, 10 nM). NS $p>0.05$; *** $p<0.001$; **** $p<0.0001$; as compared to vehicle or control or as indicated by one-way ANOVA with Tukey's multiple comparisons (A-C), or two-sided unpaired t-test (D-E).

Figure S9

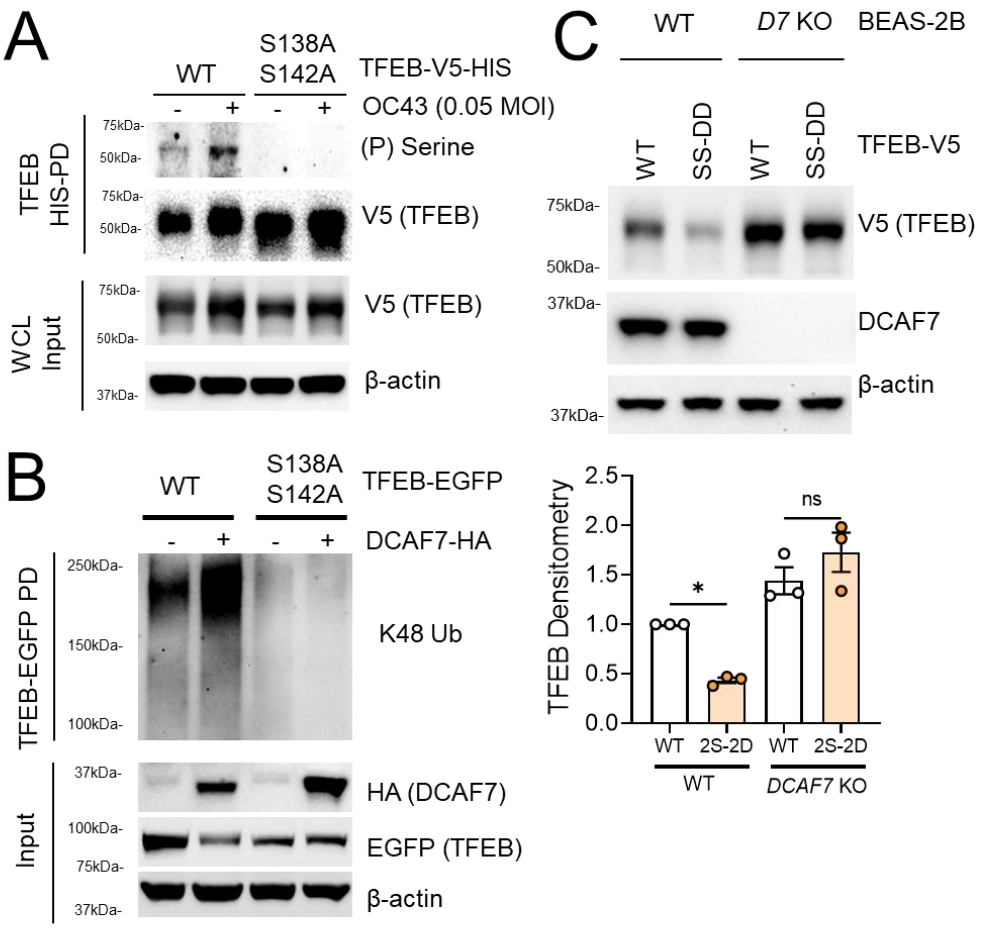

Supplementary Figure 9. TFEB phosphorylation serves as an E3 ubiquitin binding site and degron. A. Immunoblot analysis of BEAS-2B cells transfected with WT or serine-alanine (S-A) mutant TFEB. Transfected cells were infected with OC43 (0.05 MOI, 48 h) along with CFZ co-treatment (10 nM). TFEB-HIS-pull down (PD) was used to measure phospho-serine levels for the indicated TFEB WT or mutant proteins. **B.** TFEB protein was pulled down from BEAS-2B cells co-transfected with EGFP-tagged WT or TFEB phospho-mutant and control vector or DCAF7 tagged with HA. K48 ubiquitin mediated TFEB ubiquitination was detected by immunoblotting. **C.** Immunoblotting of WT or *DCAF7* KO BEAS-2B cells expressing WT or phospho-mimetic (S138D/S142D) TFEB. TFEB densitometry was calculated, data represent mean ± SEM (n=3). NS p>0.05; * p<0.05; *** p<0.001; **** p<0.0001; as compared to vehicle or control or as indicated by one-way ANOVA with Tukey's multiple comparisons (C).

Figure S10

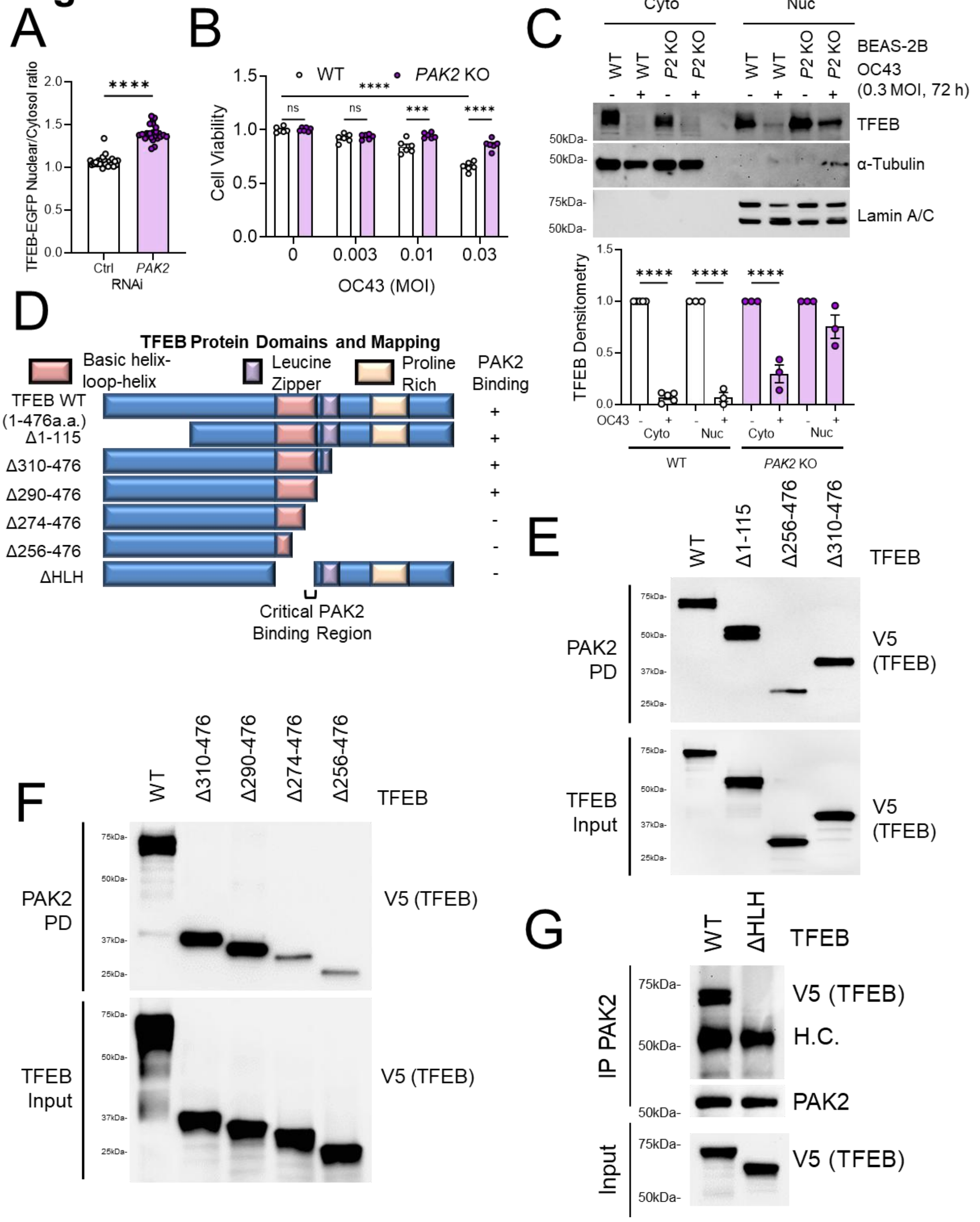
A
TFEB-EGFP Nuclear/Cytosol ratio
****
Ctrl
PAK2
RNAi
B
WT
PAK2 KO
Cell Viability
ns
ns
***
****
OC43 (MOI)
0
0.003
0.01
0.03
C
Cyto
Nuc
BEAS-2B
OC43
(0.3 MOI, 72 h)
WT
P2 KO
TFEB
α-Tubulin
Lamin A/C
50kDa-
75kDa-
TFEB Densitometry
Cyto
Nuc
WT
PAK2 KO
D
TFEB Protein Domains and Mapping
Basic helix-loop-helix
Leucine Zipper
Proline Rich
PAK2 Binding
TFEB WT (1-476a.a.)
Δ1-115
Δ310-476
Δ290-476
Δ274-476
Δ256-476
ΔHLH
Critical PAK2 Binding Region
E
WT
Δ1-115
Δ256-476
Δ310-476
TFEB
PAK2 PD
TFEB Input
V5 (TFEB)
F
WT
Δ310-476
Δ290-476
Δ274-476
Δ256-476
TFEB
PAK2 PD
TFEB Input
V5 (TFEB)
G
WT
ΔHLH
TFEB
IP PAK2
Input
V5 (TFEB)
H.C.
PAK2
75kDa-
50kDa-

Supplementary Figure 10. Kinase PAK2 phosphorylates TFEB protein to regulate its protein stability. A. Quantification of TFEB nuclear localization from Fig. 2G, data represent mean ± SEM (n=24). **B.** Viability of cells treated as in (Fig. 2K) was measured with CellTiterGlo2.0. Data were normalized to control and represent mean ± SEM (n=6). **C.** Immunoblotting of cytosolic and nuclear fractions from WT or *PAK2* KO BEAS-2B infected with OC43 (0.3 MOI, 72 h). TFEB protein densitometry was quantified, data represent mean ±SEM (n=3-5). **D.** Schematic of TFEB protein domains and deletion mapping to uncover the critical region for PAK2 binding. **E-G.** Binding assays between TFEB deletion mutants and immunoprecipitated PAK2 protein from BEAS-2B WCL. TFEB WT and mutant proteins were *in vitro* synthesized using transcription and translation (TnT) kit (Promega) and were incubated with PAK2 immunoprecipitate overnight. Following binding, the pulldown was washed and eluted for immunoblot analysis. NS p>0.05; * p<0.05; *** p<0.001; **** p<0.0001; as compared to vehicle or control or as indicated by two-sided unpaired t-test (A), two-way ANOVA with Tukey's multiple comparisons (B) or one-way ANOVA with Tukey's multiple comparisons (C).

Figure S11

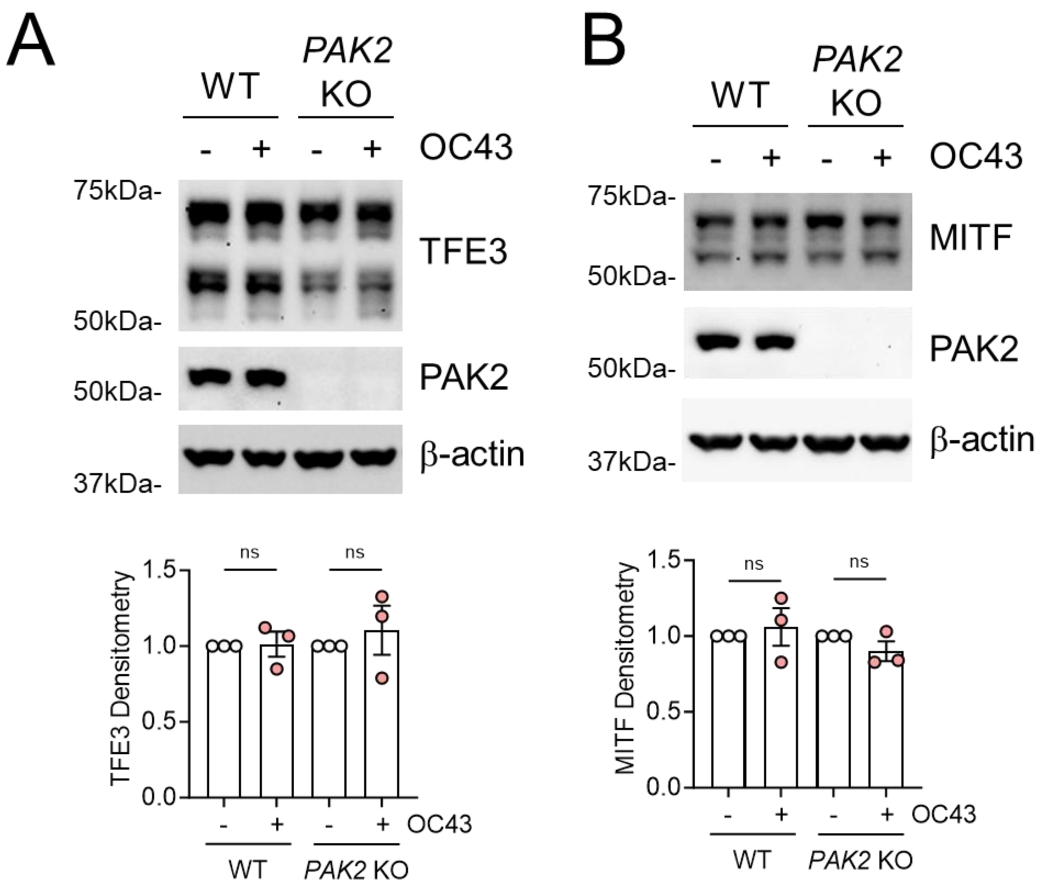

Supplementary Figure 11. TFEB homologue protein levels are not affected by PAK2. A-B. Immunoblotting of lysate from WT and PAK2 KO BEAS-2B cells following OC43 infection (72 h, MOI = 0 or 0.3). TFE3 and MITF protein densitometry were quantified, data represent mean ± SEM (n=3). NS, $p>0.05$; as compared to MOI = 0 by one-way ANOVA with Tukey's multiple comparisons (A-B).

Figure S12

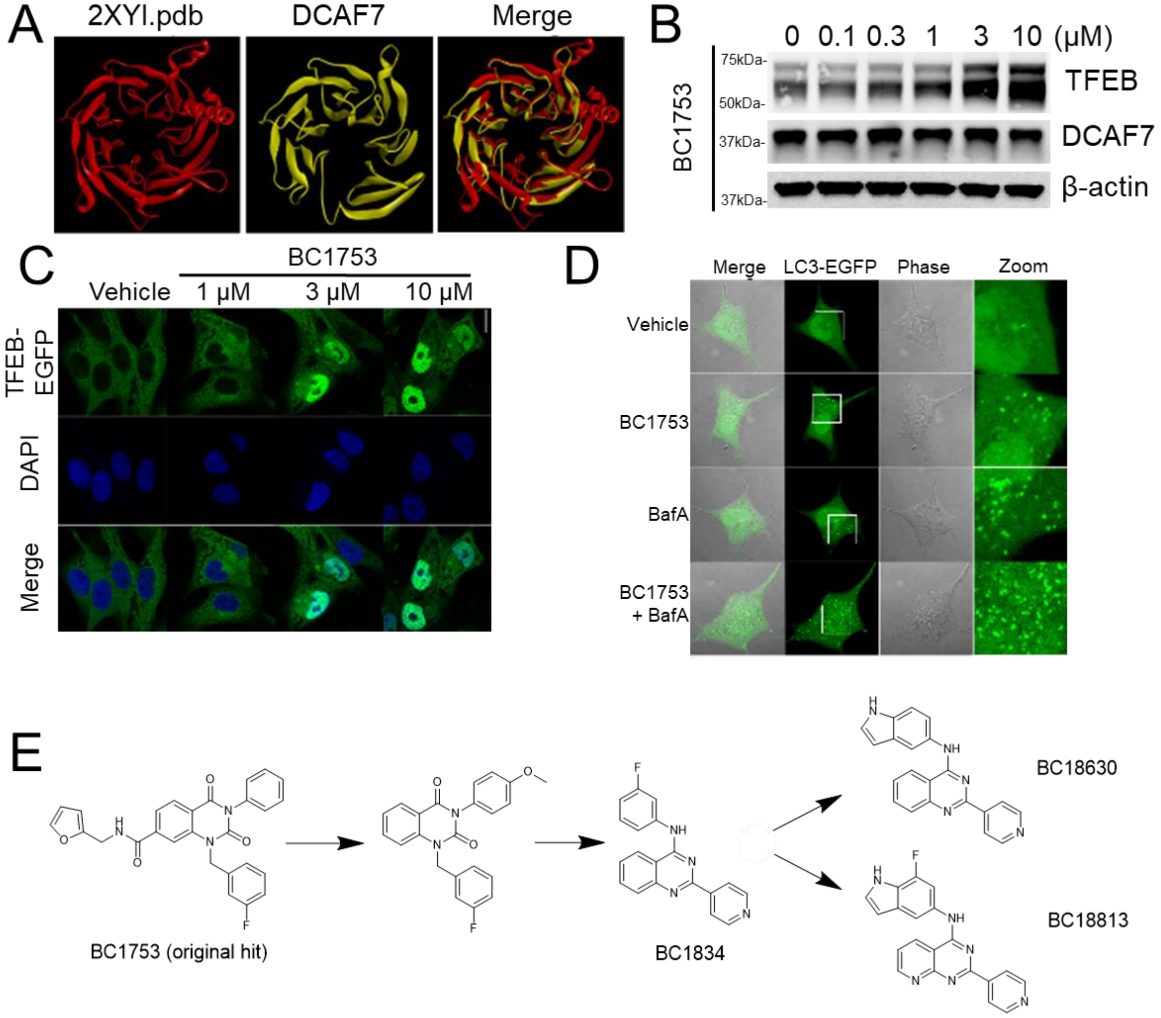

Supplementary Figure 12. Development and validation of DCAF7 small molecule inhibitors. A. An *in silico* homology modeling of DCAF7 WD-repeat protein domain using the corresponding region in NURF55 (PDB: 2XYI). Modeling identified a potential cavity for small molecule binding. **B.** Immunoblot analysis of BEAS-2B cells treated with hit compound BC1753 (18 h). **C.** Immunofluorescent microscopy of BEAS-2B cells stably expressing TFEB-EGFP treated with increasing dose of BC1753 (18 h) to detect TFEB nuclear localization. **D.** BC1753 increases autophagic flux as measured by EGFP-tagged LC3 punctae in the presence or absence of bafilomycin A (BafA; 10 μM). **E.** Hit-to-lead development of hit compound BC1753 to BC18630 and BC18813.

Figure S13

A

Synthesis of 7-fluoro-1H-indol-5-amine

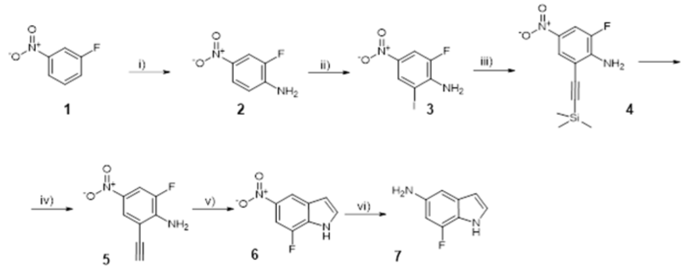

B

Synthesis of *N*-(7-fluoro-1*H*-indol-5-yl)-2-pyridin-4-ylpyrido[2,3-*d*]pyrimidin-4-amine hydrochloride

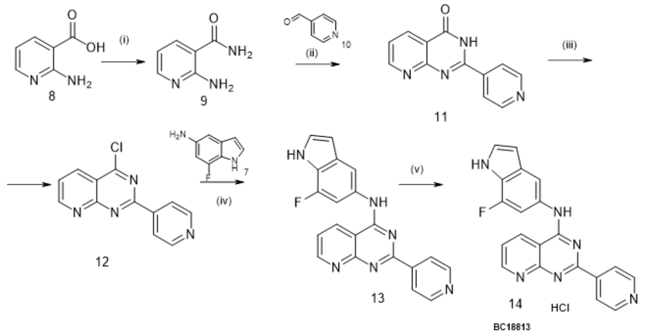

C

Synthesis of N-1H-indol-5-yl-2-pyridin-4-ylquinazolin-4-amine, hydrochloride

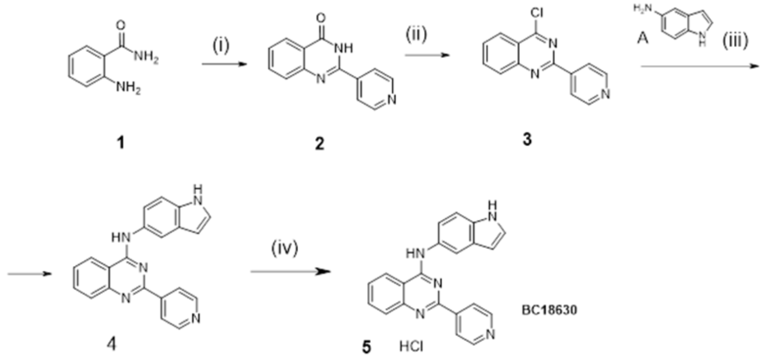

D

BC18813

Category	Parameter
Formula	C20H13FN6
Molecular weight	356.36 g/mol
Num. rotatable bonds	3
Num. H-bond acceptors	5
Num. H-bond donors	2
TPSA	79.38 Å²

E

BC18630

Category	Parameter
Formula	C21H15N5
Molecular weight	337.38 g/mol
Num. rotatable bonds	3
Num. H-bond acceptors	3
Num. H-bond donors	2
TPSA	66.49 Å²

Supplementary Figure 13. Synthesis and Characteristics of key DCAF7 inhibitor compounds. A-C. Synthesis pathway for BC18813 and BC18630. **D-E.** Key chemical parameters of DCAF7 inhibitors (A) BC18630 and (B) BC18813.

Figure S14

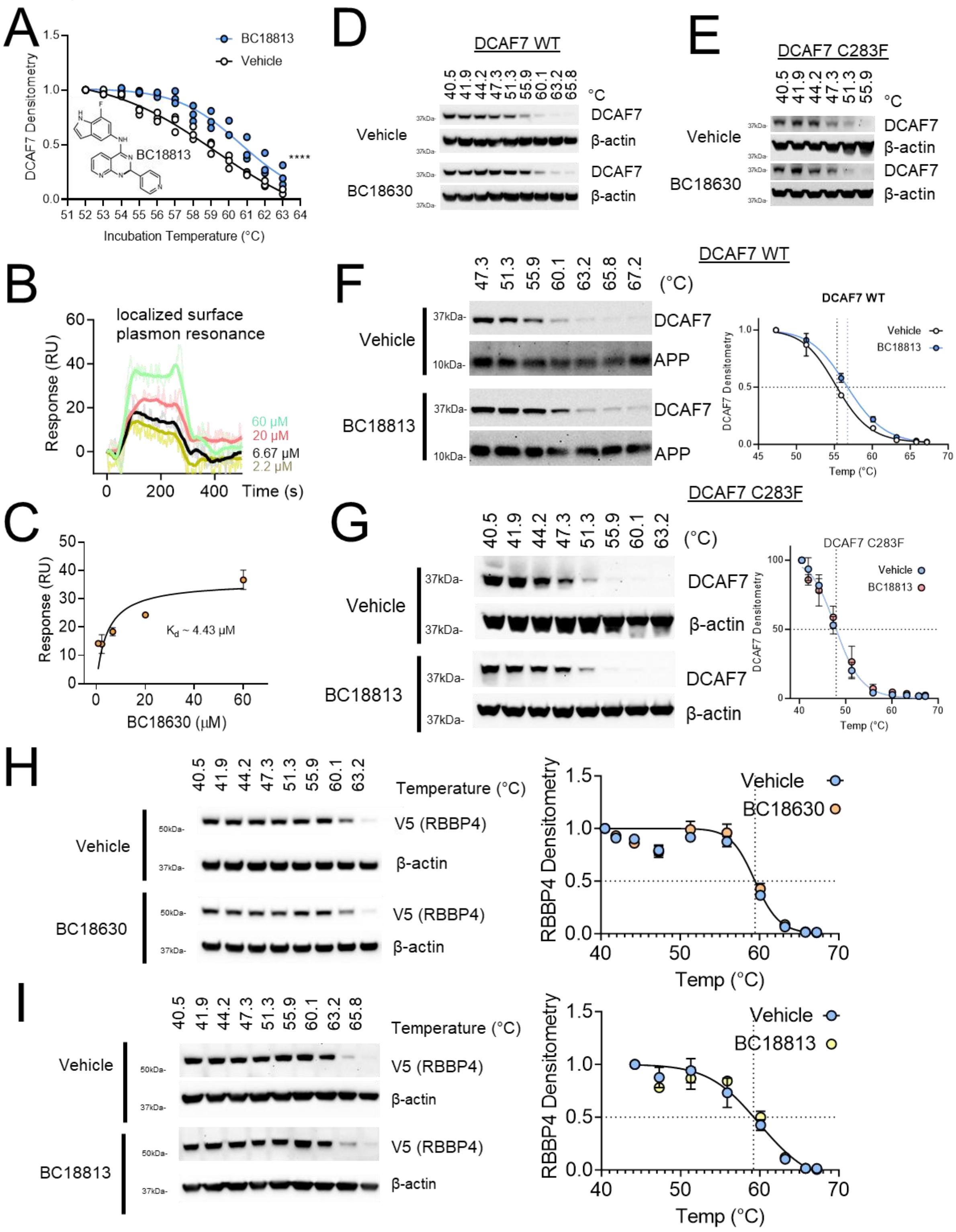
A
BC18813
Vehicle
DCAF7 Densitometry
BC18813
****
Incubation Temperature (°C)
B
localized surface plasmon resonance
Response (RU)
60 μM
20 μM
6.67 μM
2.2 μM
Time (s)
C
Response (RU)
Kd ~ 4.43 μM
BC18630 (μM)
D
DCAF7 WT
40.5 41.9 44.2 47.3 51.3 55.9 60.1 63.2 65.8 °C
Vehicle
BC18630
DCAF7
β-actin
E
DCAF7 C283F
40.5 41.9 44.2 47.3 51.3 55.9 °C
Vehicle
BC18630
DCAF7
β-actin
F
DCAF7 WT
47.3 51.3 55.9 60.1 63.2 65.8 67.2 (°C)
Vehicle
BC18813
DCAF7
APP
37kDa-
10kDa-
DCAF7 WT
Vehicle
BC18813
DCAF7 Densitometry
Temp (°C)
G
DCAF7 C283F
40.5 41.9 44.2 47.3 51.3 55.9 60.1 63.2 (°C)
Vehicle
BC18813
DCAF7
β-actin
37kDa-
DCAF7 C283F
Vehicle
BC18813
DCAF7 Densitometry
Temp (°C)
H
40.5 41.9 44.2 47.3 51.3 55.9 60.1 63.2 Temperature (°C)
Vehicle
BC18630
V5 (RBBP4)
β-actin
50kDa-
37kDa-
Vehicle
BC18630
RBBP4 Densitometry
Temp (°C)
I
40.5 41.9 44.2 47.3 51.3 55.9 60.1 63.2 65.8 Temperature (°C)
Vehicle
BC18813
V5 (RBBP4)
β-actin
50kDa-
37kDa-
Vehicle
BC18813
RBBP4 Densitometry
Temp (°C)

Supplementary Figure 14. Biophysical characterization of DCAF7 inhibitor interaction with DCAF7. A. Quantification of a cellular thermal shift assay (CETSA) of ectopically expressed DCAF7 protein in BEAS-2B cells incubated with vehicle (DMSO) or BC18813 and heated prior to precipitation and immunoblot analysis. DCAF7 protein densitometry was normalized to the lowest tested temperature (n=3). **B.** Sensorgram and regression plot of DCAF7 localized surface plasmon resonance (SPR). Recombinant DCAF7 protein was immobilized to a carboxyl sensor in OpenSPR-XT system (Nicoya) and BC18630 was titrated over the sensor. Sensorgram represents smoothed response units (RU) of DCAF7 channel corrected to reference channel using different BC18630 concentrations. **C.** SPR regression data from (B). Data were analyzed with TraceDrawer and binding kinetics calculated with a 1:1 Langmuir kinetic analysis modeling. **D-E.** Immunoblotting of CETSA assay of endogenous DCAF7 (D), and reconstituted DCAF7 with mutation of the predicted key interacting site (Cys-283-Phe) (E) expressed in DCAF7 KO cells incubated with vehicle or BC18630 (1 μM, 18 h). DCAF7 protein densitometry was quantified in Fig. 5D (n=3). **F-G.** Immunoblotting of CETSA assay of endogenous DCAF7 (F), and reconstituted DCAF7 with mutation of the predicted key interacting site (Cys-283-Phe) (G) expressed in DCAF7 KO cells incubated with vehicle or BC18813 (1 μM, 18 h). DCAF7 protein densitometry was quantified (n=3). **H-I**. Cellular thermal shift assay (CETSA) of RBBP4 (human paralogue of NURF55) from BEAS-2B cells incubated with vehicle (DMSO) or BC18813 (1 μM, 18 h) and heated prior to precipitation and immunoblot analysis. RBBP4 protein densitometry was normalized to lowest tested temperature (n=3).

Figure S15

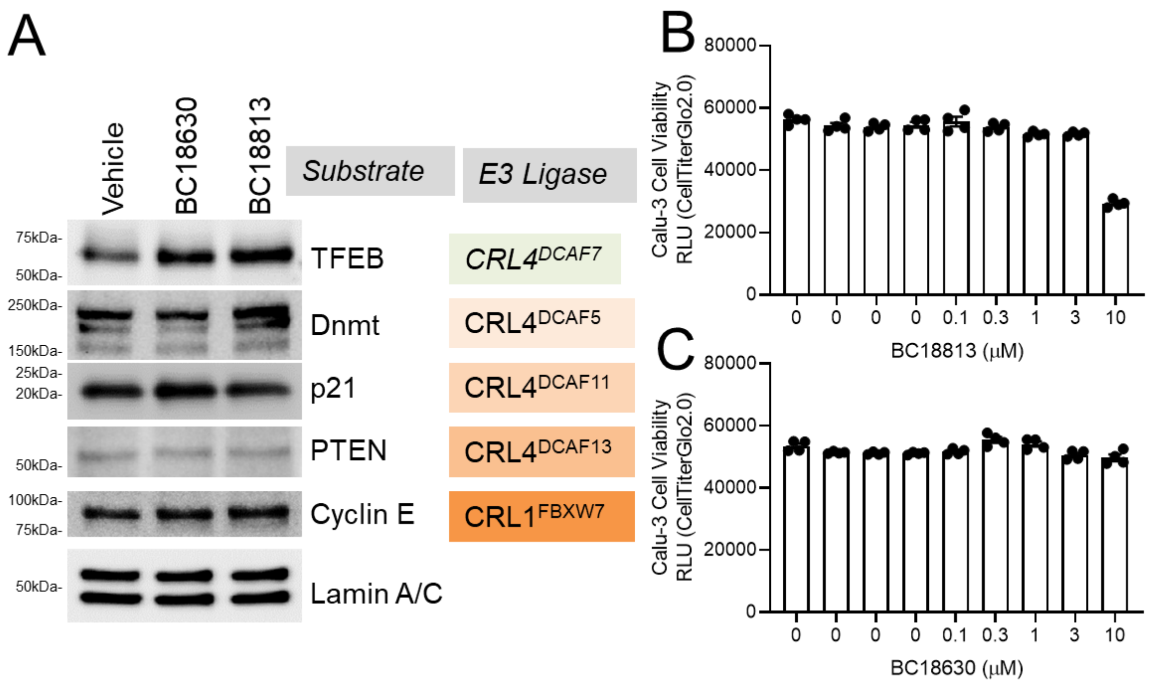

Supplementary Figure 15. DCAF7 inhibitors are specific for modulating TFEB protein levels and do not cause cell death. A. Specificity assay for DCAF7 inhibitors BC18630 and BC18813 in BEAS-2B cells. Known protein substrates of the various Cullin-RING E3 ligases denoted in the figure. These E3 ligases were chosen since their substrate recruiting proteins exhibit the highest structural homology to DCAF7. **B-C**. Calu-3 cells were treated with increasing concentrations of BC18813 (B) or BC18630 (C) for 96 h prior to cell viability measurements with CellTiterGlo2.0. Data represent mean ± SEM (n=4).

Figure S16

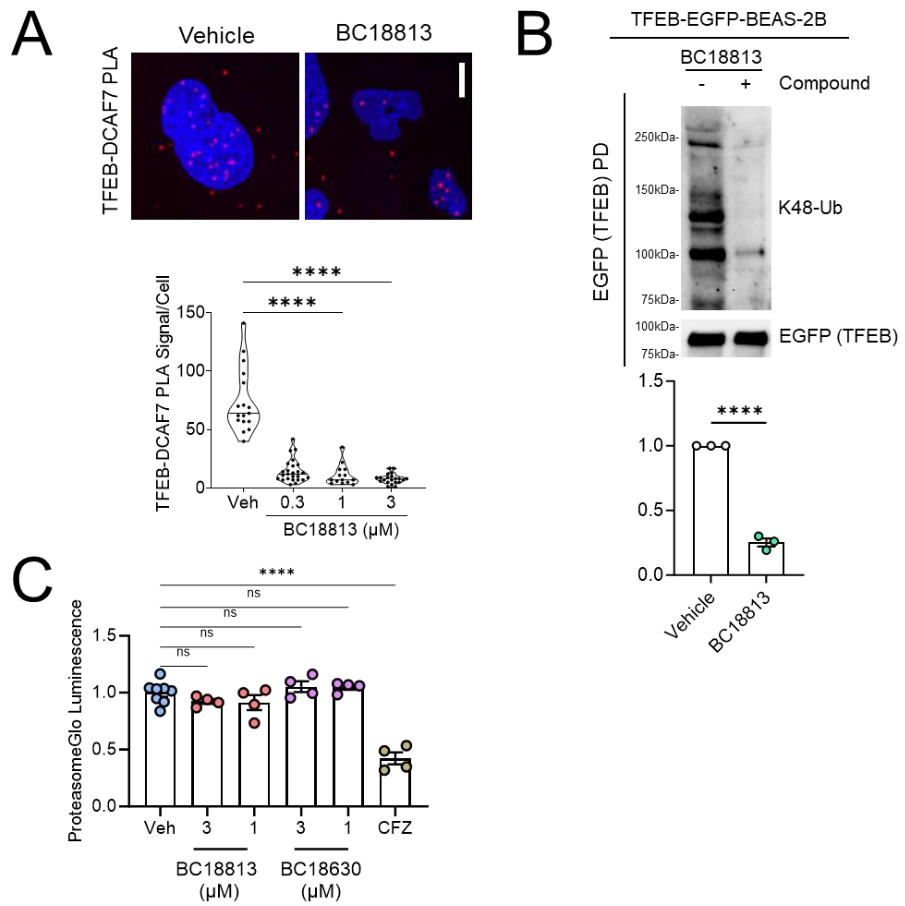

Supplementary Figure 16. Small Molecule DCAF7 inhibitors prevent DCAF7-TFEB interaction and TFEB ubiquitination. A. Proximity Ligation Assay of TFEB and DCAF7. BEAS-2B cells were treated with BC18813 and nucleus was stained with Hoechst 33342. Quantification of PLA signal per cell. Data represent mean and interquartile range in a violin plot (n=11-31 cells). **B.** TFEB cellular ubiquitination assay. BEAS-2B cells stably expressing TFEB-EGFP were treated with BC18813 (1 μM, 18 h). TFEB-EGFP protein was pulled down and ubiquitination was detected by immunoblotting. Ubiquitinated TFEB signal was quantified, data represent mean ± SEM (n=3). **C.** Proteasome activity assay (Promega) from BEAS-2B cells treated with DCAF7 inhibitors or carfilzomib at the indicated concentrations. DCAF7 inhibitors do not impair global proteasome activity. Data represent mean ±SEM (n=4). NS p>0.05; **** p<0.001; as compared to control or as indicated by two-sided t-test (B), or one-way ANOVA with Dunnett's multiple comparisons (C).

Figure S17

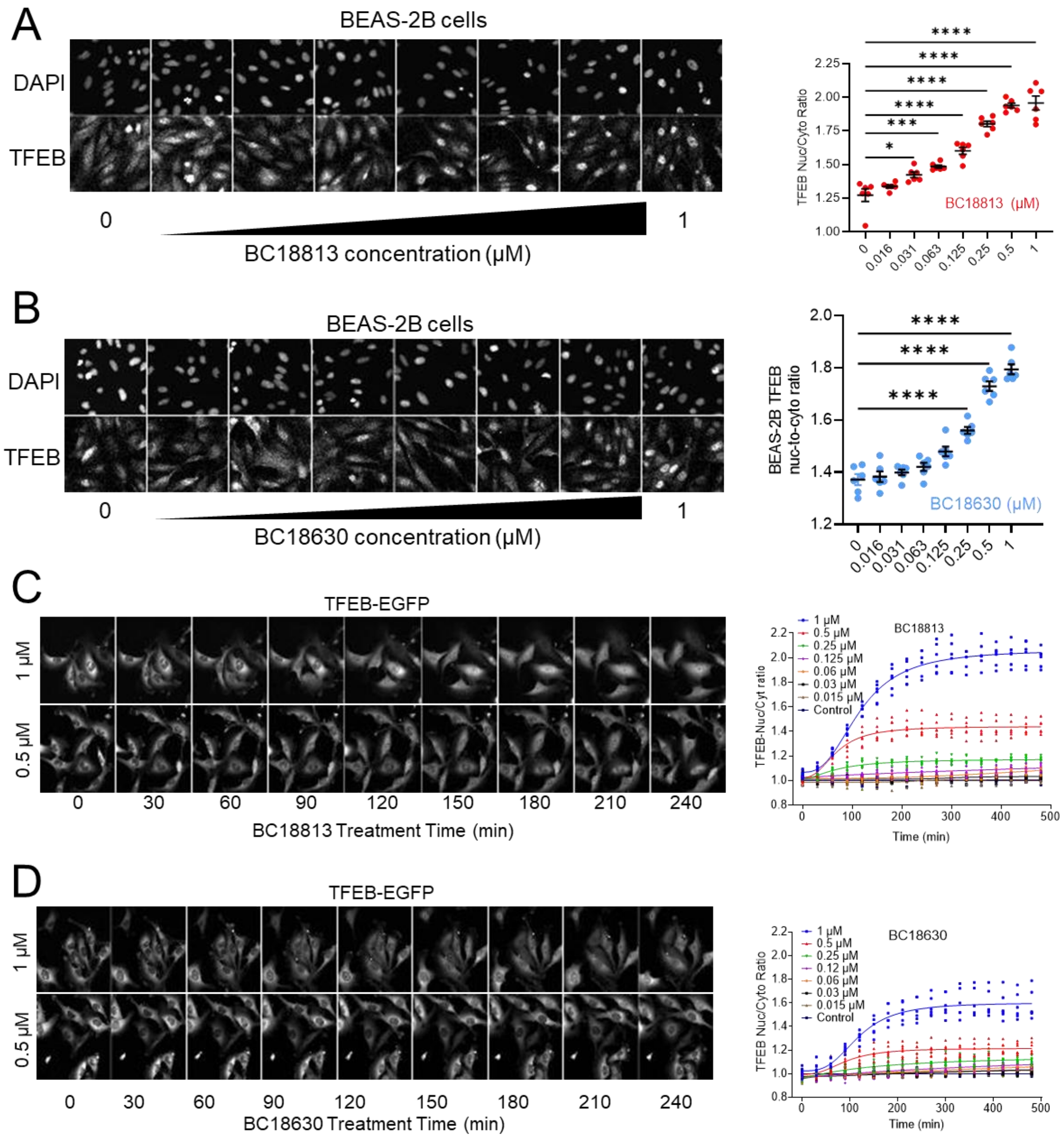

A
BEAS-2B cells
DAPI
TFEB
0
1
BC18813 concentration (μM)
TFEB Nuc/Cyto Ratio
BC18813 (μM)
B
BEAS-2B cells
DAPI
TFEB
0
1
BC18630 concentration (μM)
BEAS-2B TFEB nuc-to-cyto ratio
BC18630 (μM)
C
TFEB-EGFP
1 μM
0.5 μM
BC18813 Treatment Time (min)
BC18813
TFEB-Nuc/Cyt ratio
Time (min)
D
TFEB-EGFP
1 μM
0.5 μM
BC18630 Treatment Time (min)
BC18630
TFEB Nuc/Cyto Ratio
Time (min)

Supplementary Figure 17. Small molecule DCAF7 inhibitors increase TFEB nuclear localization A-B. Fluorescent imaging of endogenous TFEB nuclear localization in BEAS-2B cells treated with increasing concentrations of BC18813 (A) or BC18630 (B). TFEB nuclear-cytosolic ratio was quantified; data represent mean ± SEM (n=6). **C-D.** Fluorescent imaging of the kinetics of TFEB-EGFP nuclear localization with increasing concentrations of BC18813 (C) or BC18630 (D). TFEB nuclear to cytosolic ratio was calculated and normalized to time 0, data represent mean ± SEM, (n=6). * $p<0.05$; ** $p<0.01$; *** $p<0.001$; **** $p<0.0001$; as compared to vehicle/control or as indicated by one-way ANOVA with Dunnett's multiple comparisons (A-B).

Figure S18

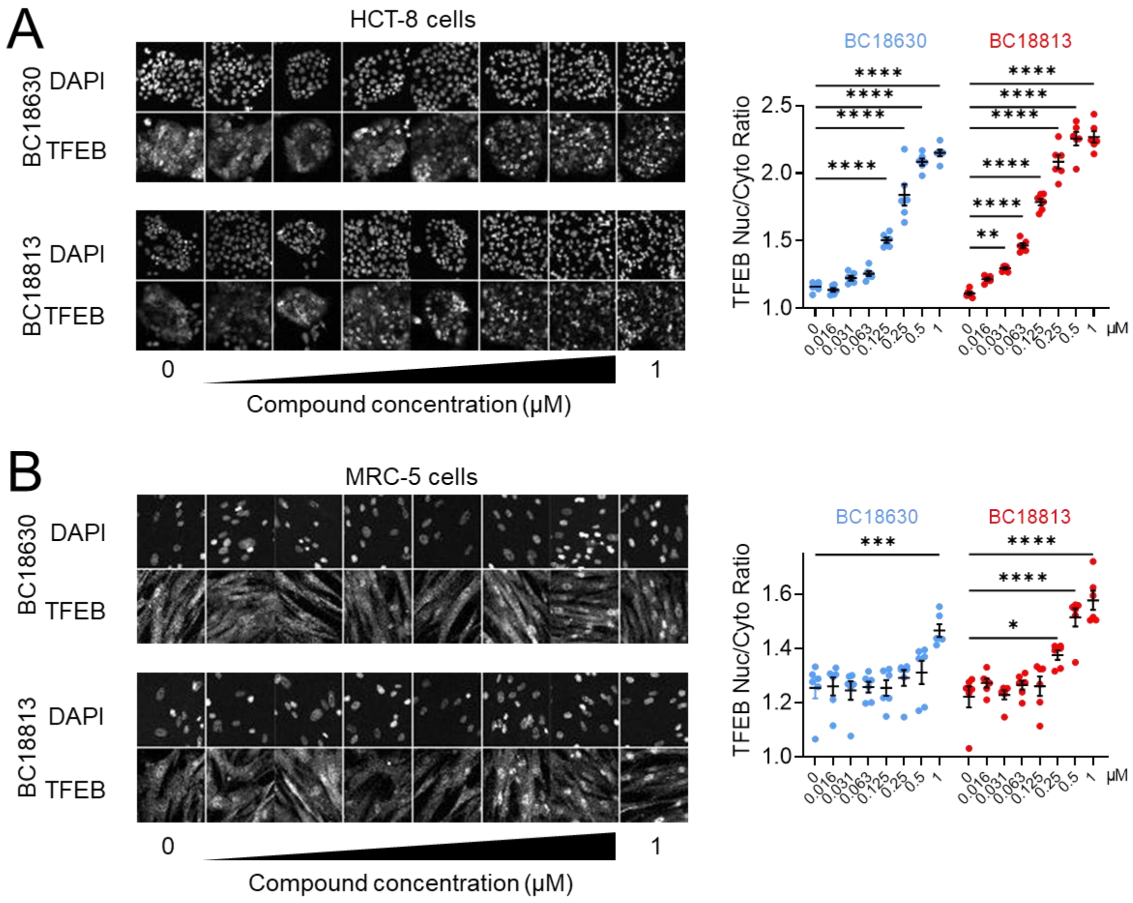

Supplementary Figure 18. DCAF7 inhibitors dose-dependently increase TFEB nuclear localization in multiple cell lines. **A-B.** Fluorescent microscopy of endogenous TFEB protein localization in HCT-8 (A), and MRC-5 (B) cells treated with the indicated concentrations of BC18813 or BC18630 (18 h). TFEB nuclear localization was quantified. Data represent TFEB nuclear to cytosolic ratio, mean ±SEM (n=6). * $p<0.05$; ** $p<0.01$; ** $p<0.001$; **** $p<0.0001$; as indicated by one-way ANOVA with Tukey's multiple comparisons (A-B).

Figure S19

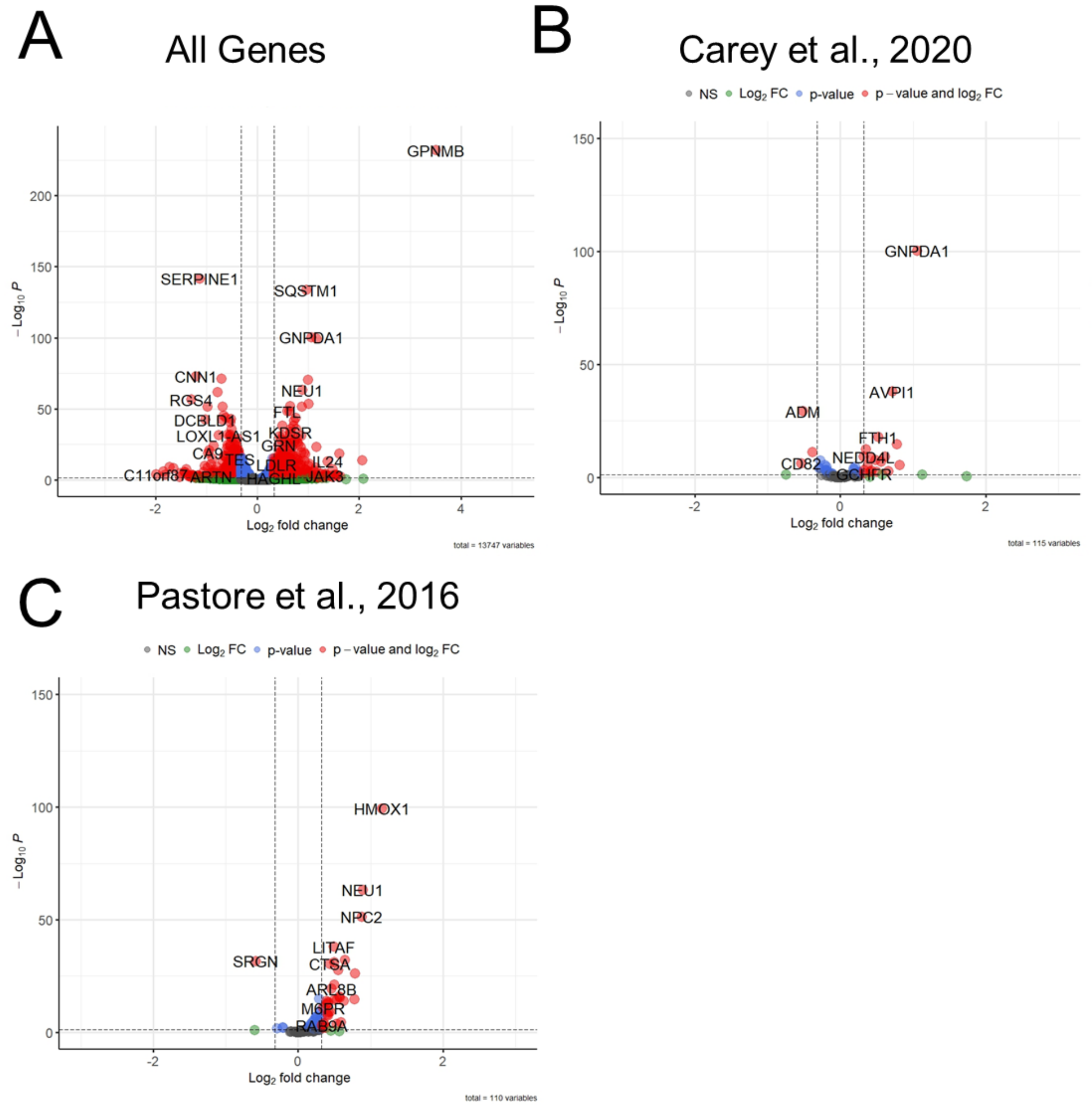

Supplementary Figure 19. DCAF7 inhibitor BC18630 induces transcriptional activation of autophagy-lysosome biogenesis. A-C. RNAseq results from BEAS-2B cells treated with BC18630 (3 μM) or vehicle for 18 h. Data are represented as a volcano plot with significance plotted against fold change (n=3 per group). Genes plotted include all genes (A), TFEB targets annotated by Carey et al., 2020, (*104*) (B), and TFEB targets annotated by Pastore et al., 2016 (*65*) (C).

Figure S20

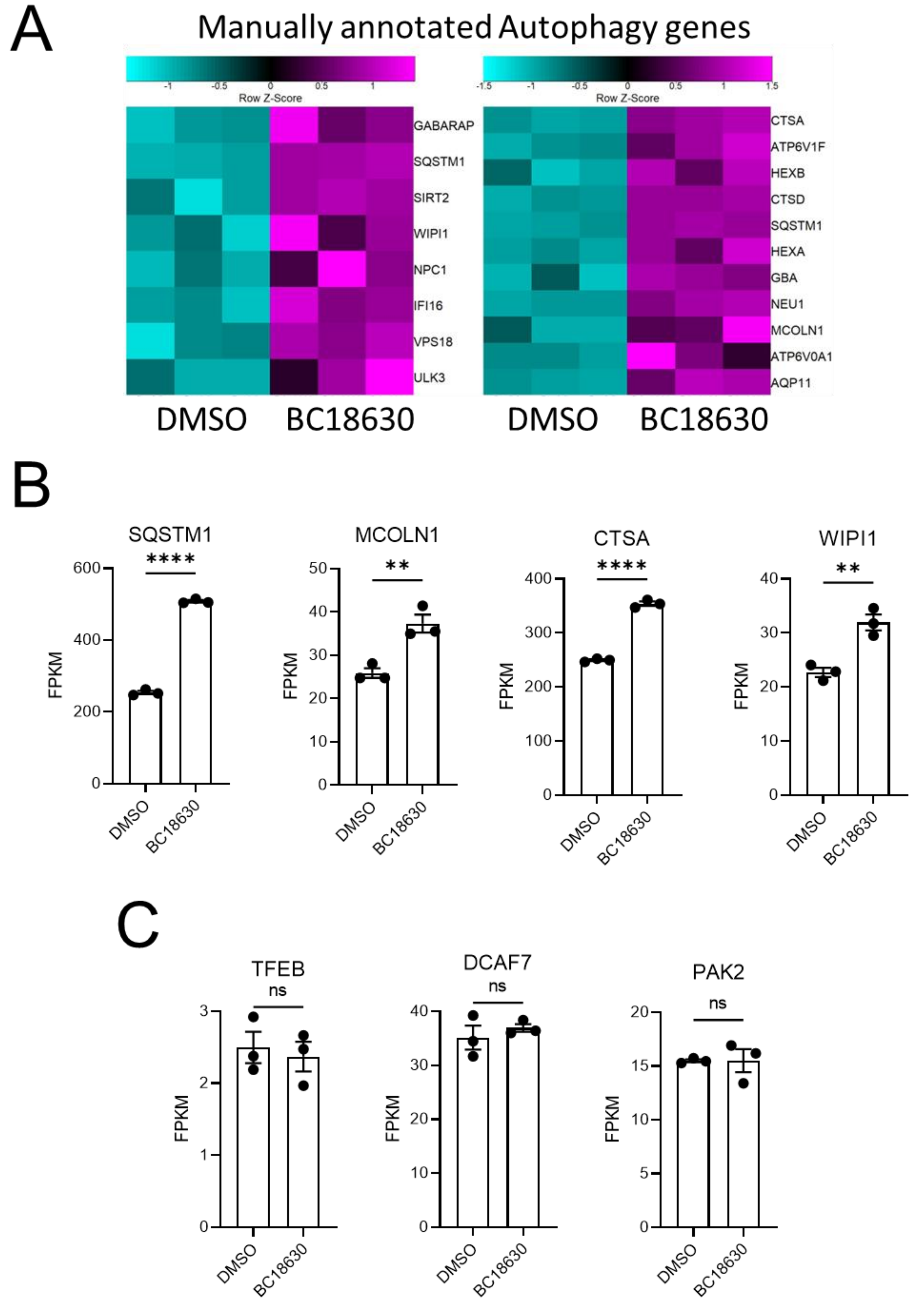

A
Manually annotated Autophagy genes
Row Z-Score
GABARAP
SQSTM1
SIRT2
WIPI1
NPC1
IFI16
VPS18
ULK3
CTSA
ATP6V1F
HEXB
CTSD
SQSTM1
HEXA
GBA
NEU1
MCOLN1
ATP6V0A1
AQP11
DMSO
BC18630
DMSO
BC18630
B
SQSTM1
****
MCOLN1
**
CTSA
****
WIPI1
**
FPKM
DMSO
BC18630
C
TFEB
ns
DCAF7
ns
PAK2
ns
FPKM
DMSO
BC18630

Supplementary Figure 20. DCAF7 inhibitor BC18630 specifically enhances TFEB target genes. A. Heat map of autophagy genes from RNAseq (Fig. S19) in BEAS-2B cells treated with DMSO or BC18630 (3 μM). **B-C**. Quantitative PCR validation of TFEB targeted genes (B), and TFEB, DCAF7, PAK2 themselves (C). Data represent mean ± SEM, (n=3). NS $p>0.05$; ** $p<0.01$; **** $p<0.0001$; as compared to control by two-sided unpaired t-test (B-C).

Figure S21

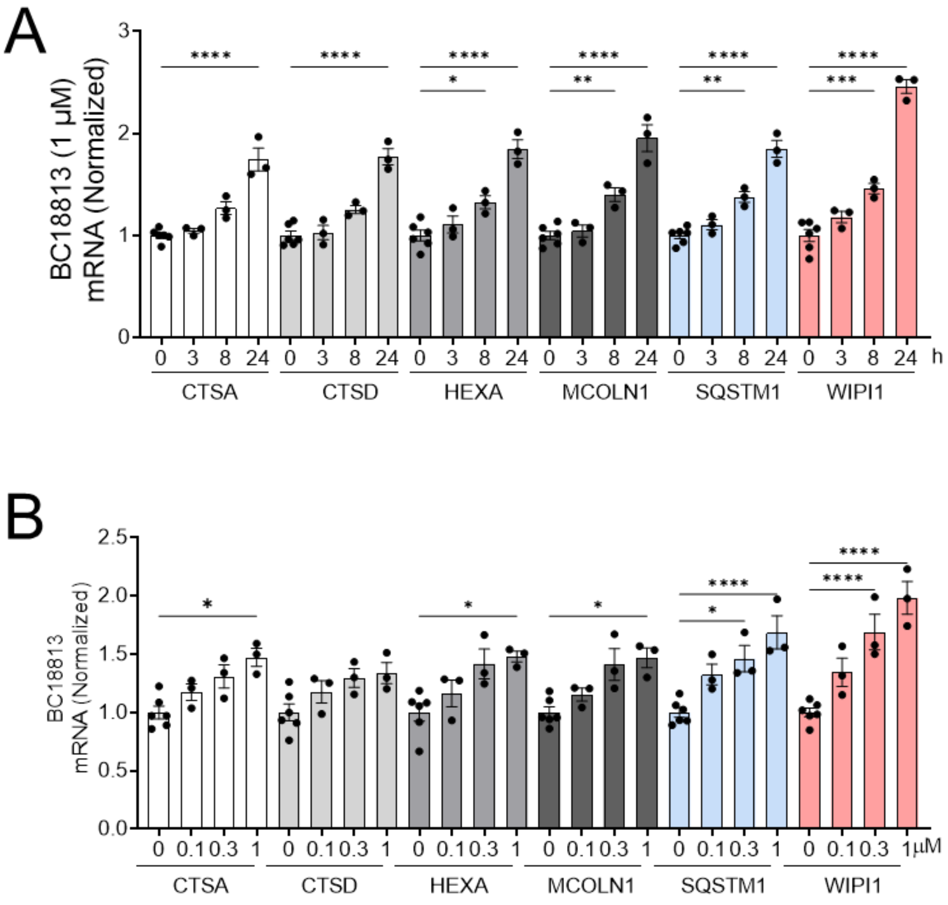

Supplementary Figure 21. DCAF7 inhibitors stimulate expression of TFEB transcription targets. A-B. Quantitative PCR analysis of known lysosomal transcriptional targets of TFEB obtained from BEAS-2B cells treated with BC18813 (1 µM) for the indicated time or with increasing concentrations and harvested at 18 h. Data represent fold change in indicated target mRNA levels relative to control treatment; mean ±SEM (n=3-6). * $p<0.05$; ** $p<0.01$; *** $p<0.001$; **** $p<0.0001$; as compared to vehicle or control or as indicated by one-way ANOVA with Dunnett's multiple comparisons (A-B).

Figure S22

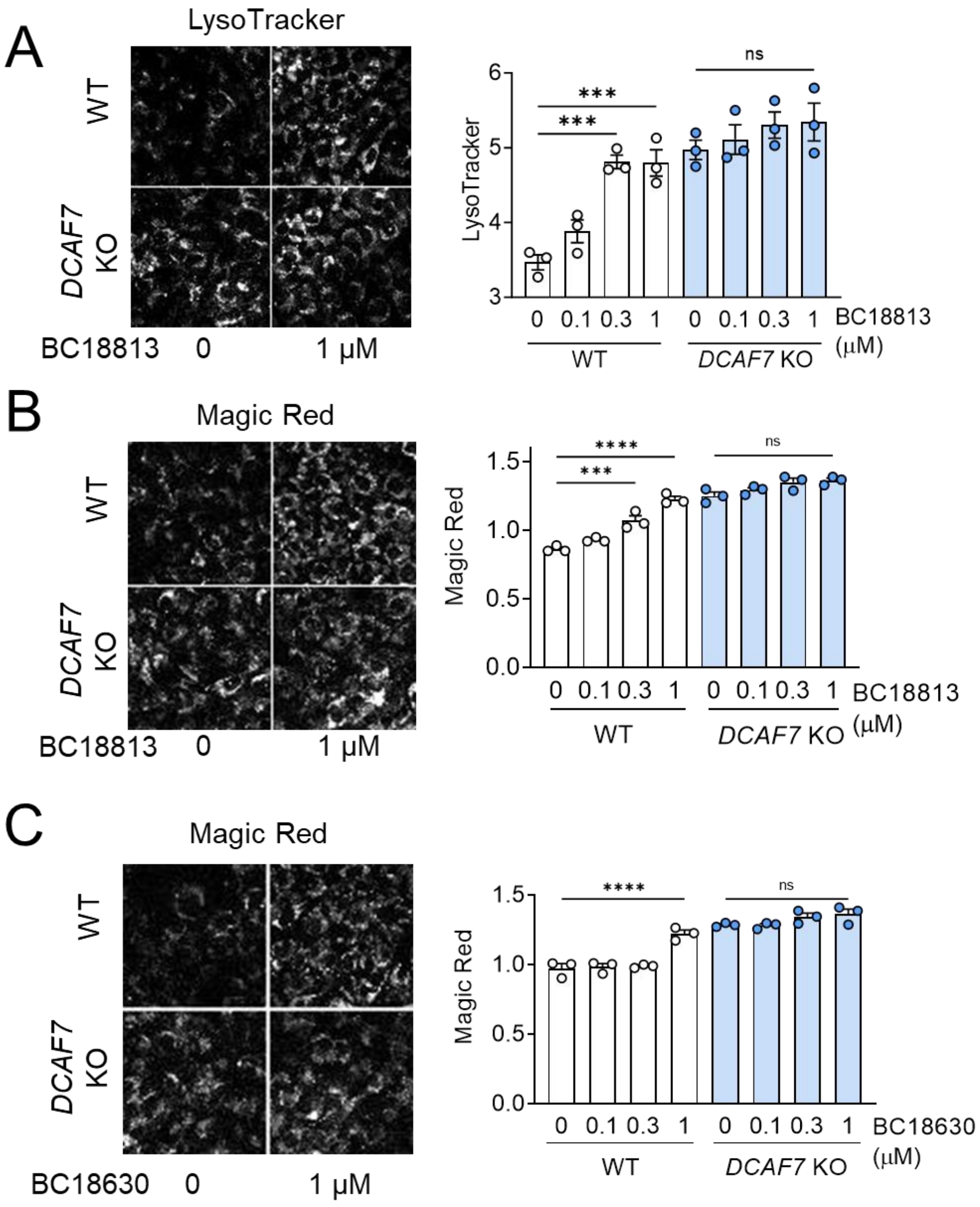
A
LysoTracker
WT
DCAF7 KO
BC18813 0 1 μM
ns
***
***
LysoTracker
0 0.1 0.3 1 0 0.1 0.3 1 BC18813 (μM)
WT
DCAF7 KO
B
Magic Red
WT
DCAF7 KO
BC18813 0 1 μM
****
***
ns
Magic Red
0 0.1 0.3 1 0 0.1 0.3 1 BC18813 (μM)
WT
DCAF7 KO
C
Magic Red
WT
DCAF7 KO
BC18630 0 1 μM
****
ns
Magic Red
0 0.1 0.3 1 0 0.1 0.3 1 BC18630 (μM)
WT
DCAF7 KO

Supplementary Figure 22. DCAF7 small molecule inhibitors increase lysosomal number and activity. A. Fluorescent micrograph of WT or *DCAF7* KO BEAS-2B cells treated with BC18630 and live stained with LysoTracker, a measure of lysosomal number. LysoTracker fluorescence was quantified, data represent median LysoTracker signal from each sample, mean ± SEM (n=3). **B-C.** Fluorescent micrograph of lysosomal activity in WT or *DCAF7* KO BEAS-2B cells treated with BC18813 (B) or BC18630 (C) as assessed by Magic Red intensity. Magic Red fluorescence was quantified; data represent each well's median Magic Red signal intensity per cell, mean ± SEM (n=3). NS $p>0.05$; * $p<0.05$; ** $p<0.01$; *** $p<0.001$; **** $p<0.0001$; as indicated by one-way ANOVA with Tukey's multiple comparisons (A-C).

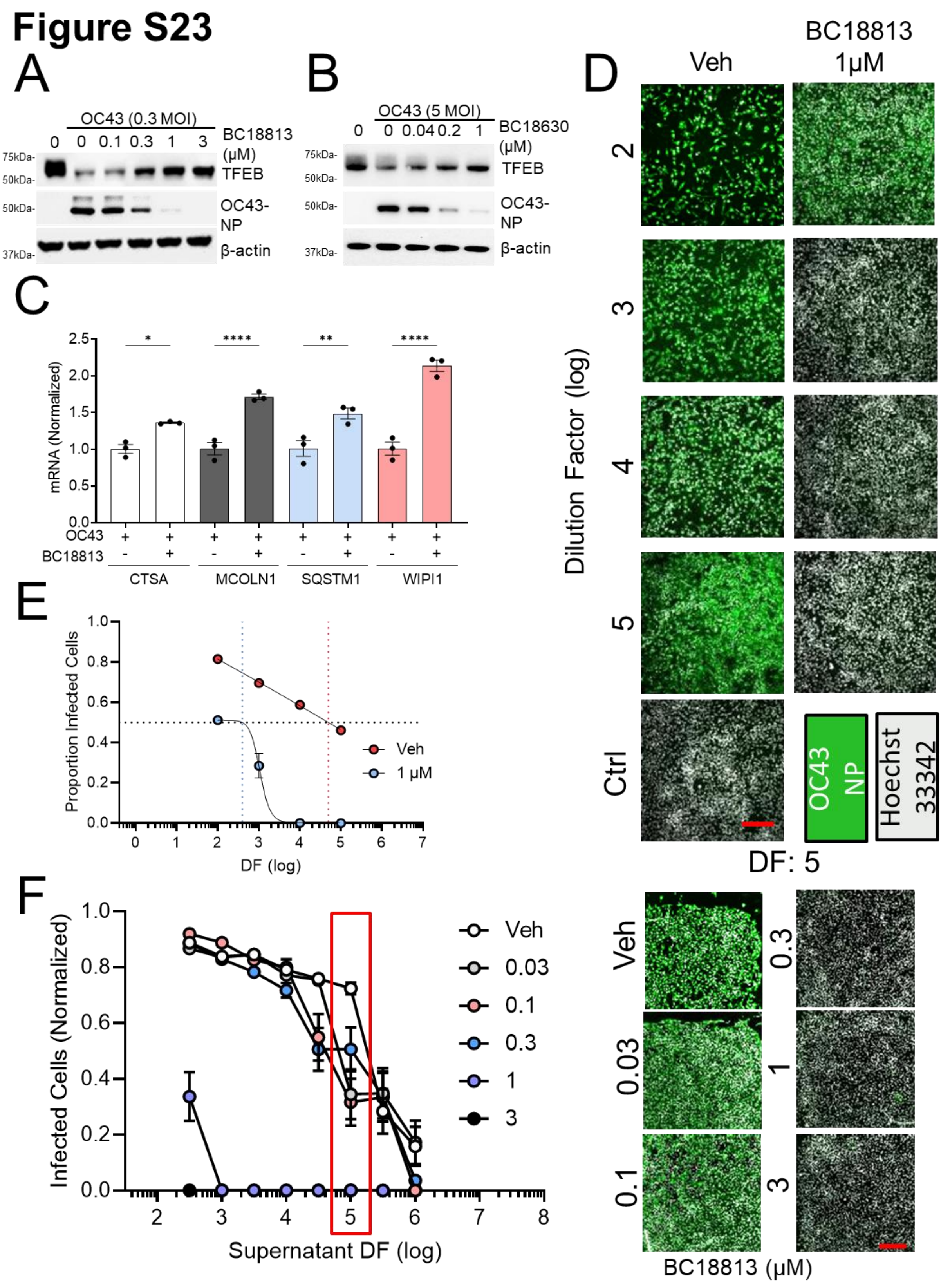

Supplementary Figure 23. DCAF7 inhibitors decrease coronaviral infectivity. A-B. Immunoblot analysis of OC43 infected BEAS-2B cells treated with increasing concentration of BC18813 for 72h (A), or BC18630 for 24h (B). TFEB protein levels and OC43 viral protein levels were measured by immunoblotting. **C.** Transcription analysis via qPCR of

key TFEB transcriptional targets in BEAS-2B cells infected with OC43 (0.3 MOI) and co-treated with BC18813 (1 μM, 72 h). Data represent mean ± SEM (n=3). **D-E.** *in vitro* infectivity assay with BC18813 pre-treatment. Briefly, WT BEAS-2B cells were pre-treated with vehicle or BC18813 (18 h) before infection with equal amounts of OC43 (0.03 MOI) virus for 18 h. Cells were washed and re-incubated in fresh media for an additional 72 h. Equal amounts of supernatant were then collected and diluted in 10-fold titration series prior to use as inoculates to naïve WT BEAS-2B cells for another 72 h of infection. Following incubation, cells were fixed and processed for OC43 NP immunostaining and automated microscopy. Representative images were captured (D), and percentage of infected cells per well was calculated with CellProfiler (E). Data represent mean ± SEM (n=12). **F.** Quantification and representative images of *in vitro* infectivity assay with extended BC18813 dose course as described above. Data represent mean ±SEM (n=16). NS $p>0.05$; * $p<0.05$; ** $p<0.01$; *** $p<0.001$; **** $p<0.0001$; as indicated by one-way ANOVA with Tukey's multiple comparisons (C). Scale bar = 300 μm.

Figure S24

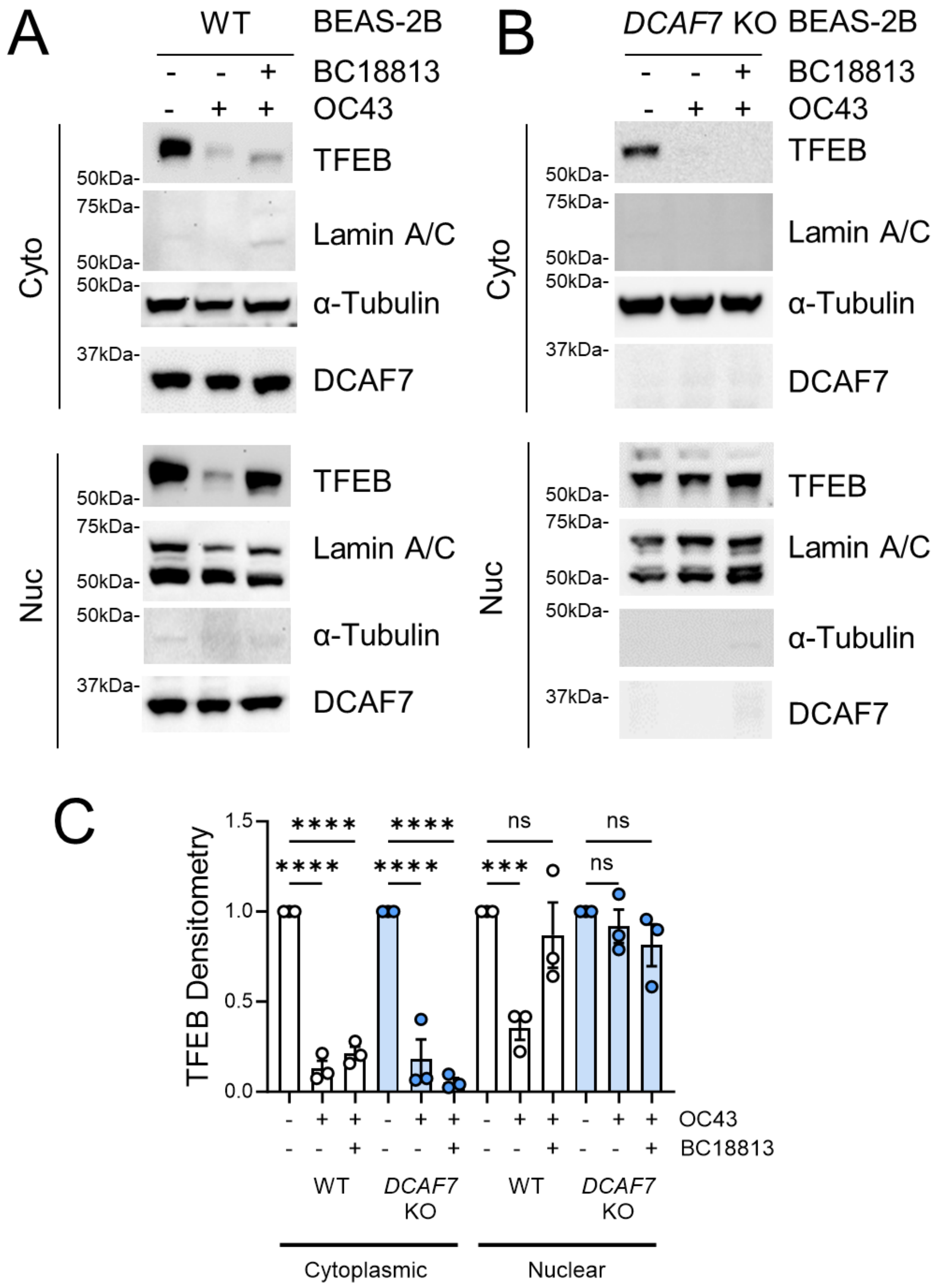

A
WT
BEAS-2B
BC18813
OC43
TFEB
Lamin A/C
α-Tubulin
DCAF7
Cyto
Nuc
50kDa-
75kDa-
37kDa-
B
DCAF7 KO BEAS-2B
BC18813
OC43
TFEB
Lamin A/C
α-Tubulin
DCAF7
Cyto
Nuc
C
TFEB Densitometry
1.5
1.0
0.5
0.0
****
***
ns
OC43
BC18813
WT
DCAF7 KO
Cytoplasmic
Nuclear

Supplementary Figure 24. DCAF7 inhibitors preserve TFEB nuclear localization during infection. A-B. Immunoblotting of cytosolic and nuclear fractions from WT (A) or *DCAF7* KO (B) BEAS-2B cells treated with BC18813 (1 μM) and infected with OC43 (72 h, MOI = 0, 0.3). **C.** TFEB protein densitometry was quantified and normalized to MOI = 0 from experiments in A-B and data represent mean ±SEM (n=3-5).

Figure S25

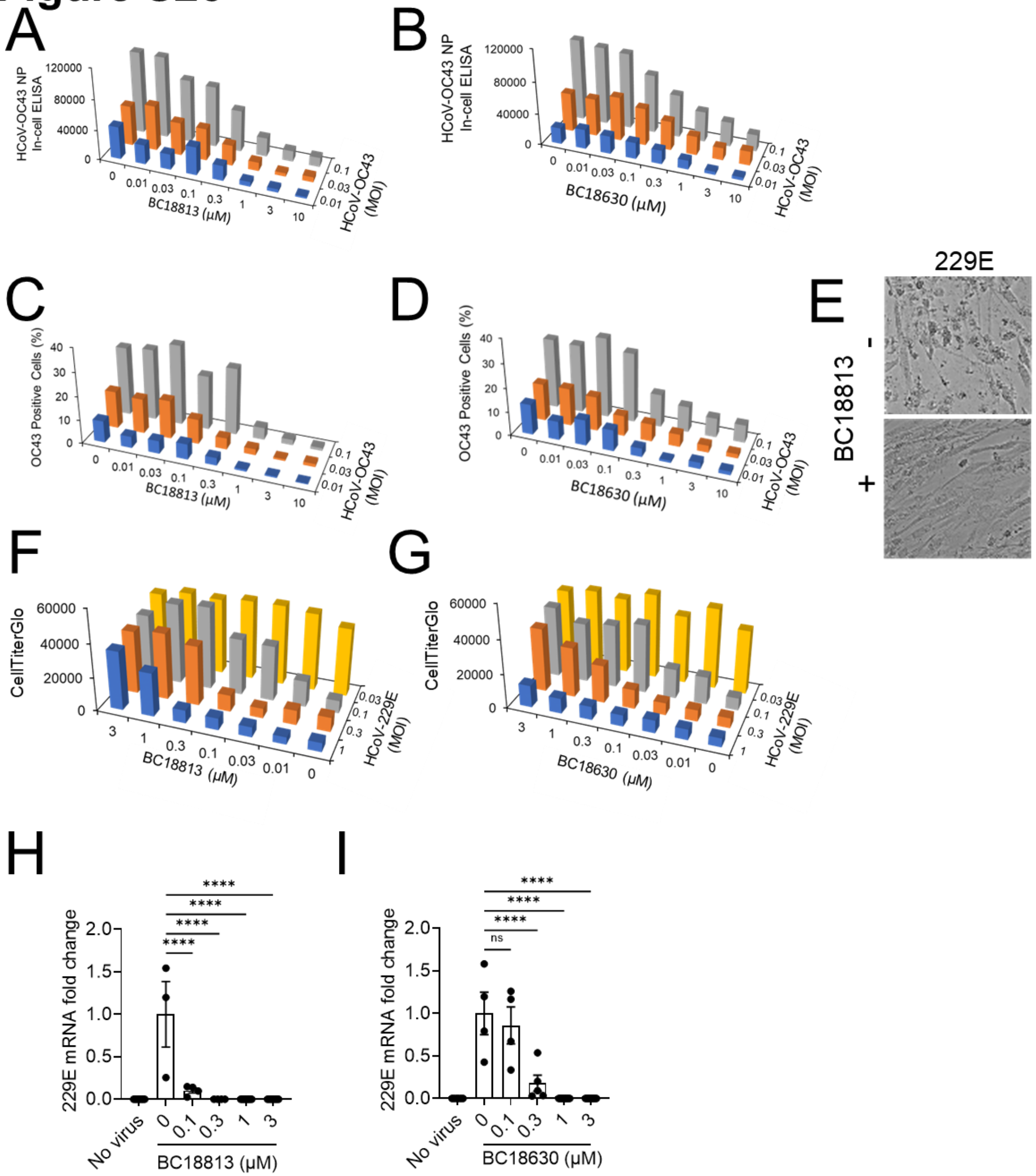
A
HCoV-OC43 NP In-cell ELISA
BC18813 (µM)
HCoV-OC43 (MOI)
B
HCoV-OC43 NP In-cell ELISA
BC18630 (µM)
HCoV-OC43 (MOI)
C
OC43 Positive Cells (%)
BC18813 (µM)
HCoV-OC43 (MOI)
D
OC43 Positive Cells (%)
BC18630 (µM)
HCoV-OC43 (MOI)
E
229E
BC18813
-
+
F
CellTiterGlo
BC18813 (µM)
HCoV-229E (MOI)
G
CellTiterGlo
BC18630 (µM)
HCoV-229E (MOI)
H
229E mRNA fold change
No virus
BC18813 (µM)
****
I
229E mRNA fold change
No virus
BC18630 (µM)
ns
****

Supplementary Figure 25. Small molecule DCAF7 inhibitors are protective against coronaviral infection *in vitro*. A-B. In-cell ELISA analysis of HCT-8 colon carcinoma cells treated with the indicated concentrations of BC18813 (A) and BC18630 (B) and infected with OC43 at the indicated MOI (48 h). OC43 infectivity was detected by OC43 Nucleoprotein (NP) expression. Data represent mean value (n=4). **C-D.** HCT-8 cells treated as in panel (A-B) but assessed by quantifying the percentage of OC43-positive, HCT-8 cells through high-content fluorescent imaging of OC43 nucleoprotein. Data represent mean value (n=3-4). **E.** Phase micrograph of cytopathic effects from *alpha* coronavirus 229E infection in MRC-5 fetal lung fibroblast cells in the absence (-) or presence (+) of BC18813 (1 µM, 72 h). **F-G.** Quantification of cell number by CellTiterGlo2.0 following infection with *alpha* coronavirus 229E (MOI = 0.1, 48 h) and treatment with BC18813 (F) or BC18630 (G) at the indicated doses. Data represent mean (n= 3-4). **H-I.** qPCR analysis of 229E viral RNA following infection and treatment with increasing concentrations of BC18813 (H) or BC18630 (I). Data represent mean ±SEM (n=3). NS, $p>0.05$; ****, $p<0.0001$; as indicated by one-way ANOVA with Tukey's multiple comparisons (H-I).

Figure S26

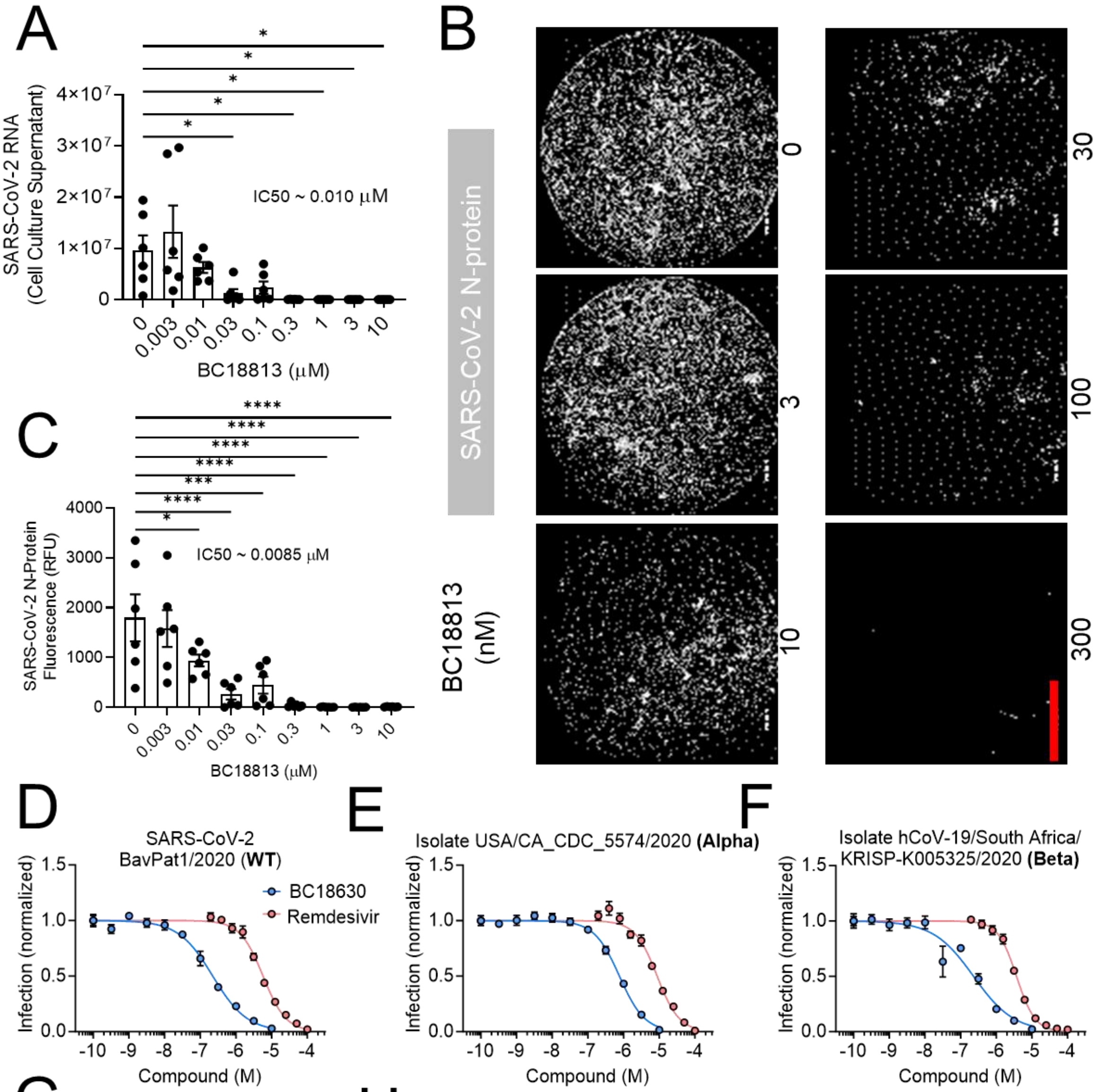

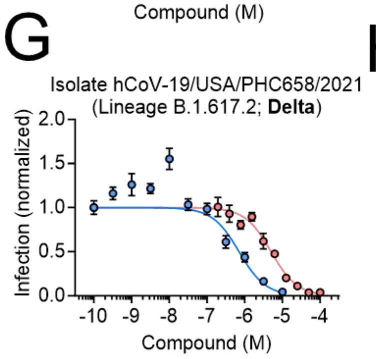

H

	Anti SARS-CoV-2 IC50 (nM)				Cytotoxicity CC50 (nM)	
	Wild type	Alpha	Beta	Delta	Vero E6 DMEM	Vero E6 MEM
BC18813	406.6	286.9	320.9	187.8	>10,000	>10,000

Supplementary Figure 26. DCAF7 Inhibitors decrease SARS-CoV-2 infection in vitro

A-C. Cell-based SARS-CoV-2 infection assay. Briefly, Calu-3 human lung carcinoma cells were pre-treated with the indicated concentrations of BC18813 for 24 h prior to media swap and incubation with SARS-CoV-2 virus without DCAF7 inhibitors (USA-WA-1/2020, MOI 0.01). After a 75 min inoculation, media was replaced with fresh media containing the indicated concentrations of DCAF7 inhibitor compound. Supernatant samples were taken after an additional 48 h incubation for viral RNA detection (**A**). Data represent mean ± SEM (n=6), and IC50 values were determined by sigmoidal nonlinear regression. Cells were fixed and stained for SARS-CoV-2 nucleoprotein (NP) for fluorescent microscopy and quantification of viral signal (**B-C**). Data represent mean ± SEM (n=6), and IC50 values were determined by sigmoidal nonlinear regression. **D-H.** Vero E6 kidney cells were infected with SARS-CoV-2 WT (D) and WHO previously-annotated Variants of Concern *Alpha* (E), *Beta* (F), and *Delta* (G) along with a titration of BC18630 or Remdesivir at indicated concentrations. Infection was quantified by Virospot reduction assay that quantified proportion of well surface area covered by viral-positive immunostaining. Data represent mean ± SEM, n=4 per condition. **H.** IC50 calculation of DCAF7 inhibitor *in vitro* efficacy in Vero E6 cells against SARS-CoV-2 variants of concern. IC50 values calculated from nonlinear regression with variable Hill slope. n=4 samples per condition (D-G). Cytotoxicity (CC50) of DCAF7 inhibitor was estimated to be larger than 10 µM, the highest concentration tested. Data represent mean from n=4 per condition. * $p<0.05$; *** $p<0.001$; **** $p<0.0001$; as indicated by one-way ANOVA with Dunnett's multiple comparisons (A, C). Scale bar = 2000 µm.

Figure S27

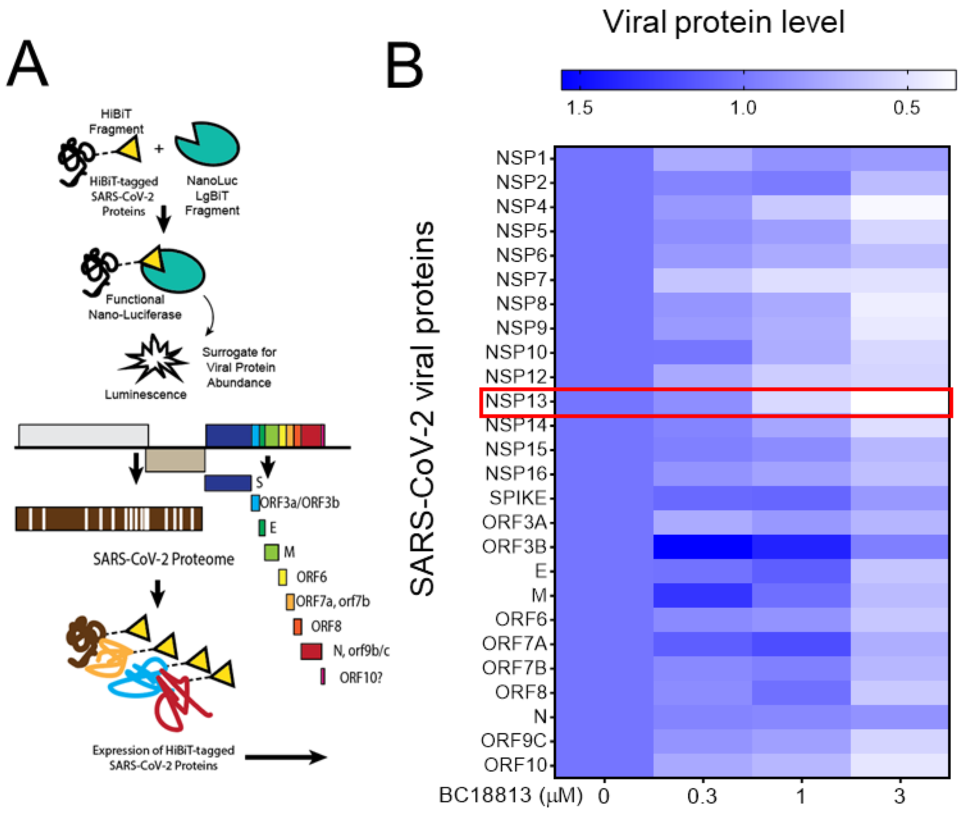

Supplementary Figure 27. DCAF7 inhibitor treatment decrease SARS-CoV-2 viral protein levels in cells. A. Schematic of SARS-CoV-2 viral protein screening strategy, HiBiT schematic after Chen et al., 2021 (*105*). Codon optimized SARS-CoV-2 protein sequences (*66*) were cloned into HiBiT-tagged expression vectors and expressed in BEAS-2B. **B**. Protein stability profile of SARS-CoV-2 viral proteins expressed in BEAS-2B for 18 h, and then treated with BC18813 (24 h) with increasing doses. Data are represented by mean values of n=2-4 biological replicates.

Figure S28

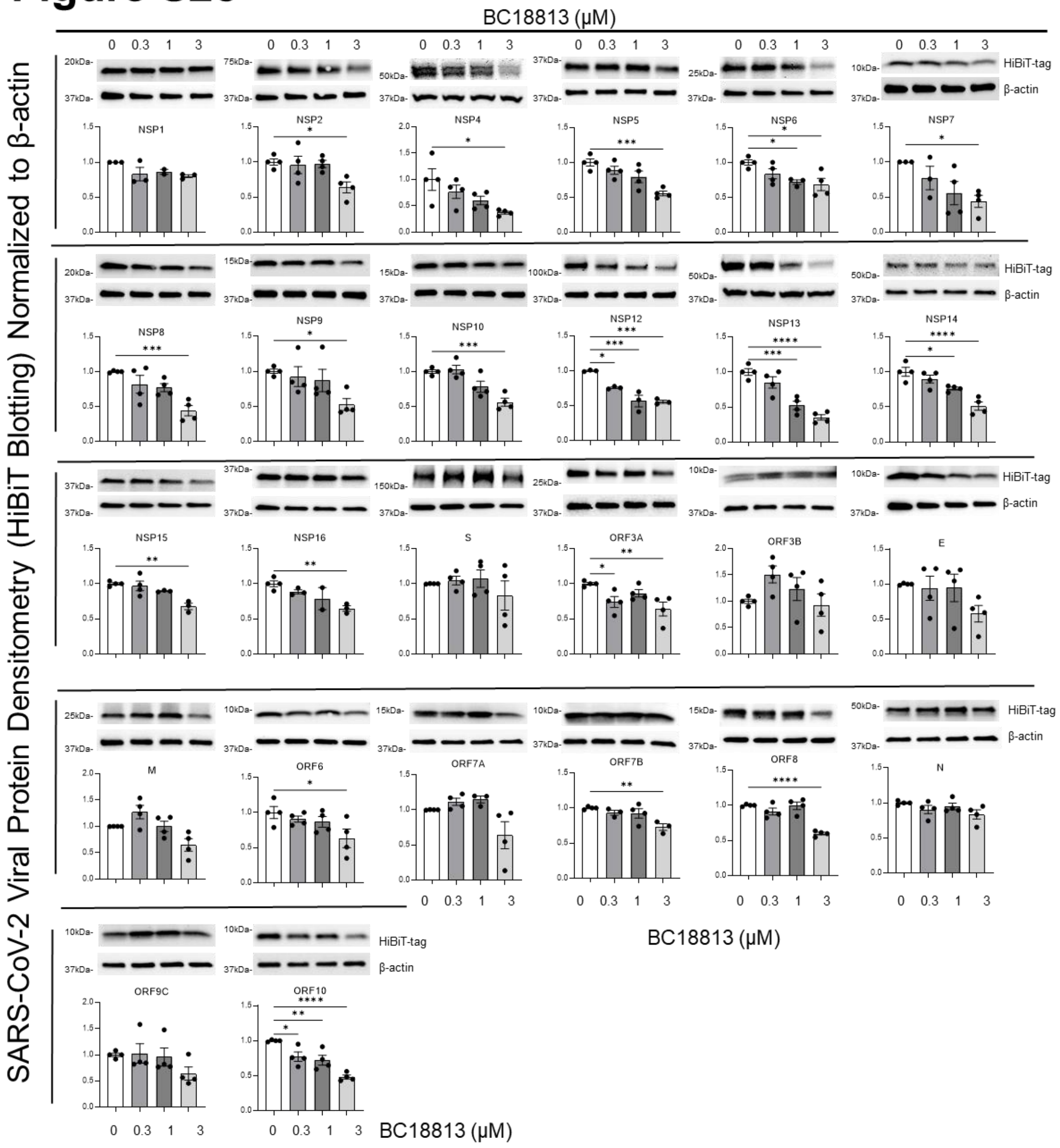

BC18813 (μM)
SARS-CoV-2 Viral Protein Densitometry (HiBiT Blotting) Normalized to β-actin
0 0.3 1 3
HiBiT-tag
β-actin
NSP1
NSP2
NSP4
NSP5
NSP6
NSP7
NSP8
NSP9
NSP10
NSP12
NSP13
NSP14
NSP15
NSP16
S
ORF3A
ORF3B
E
M
ORF6
ORF7A
ORF7B
ORF8
N
ORF9C
ORF10

Supplementary Figure 28. Several SARS-CoV-2 viral proteins decrease in response to DCAF7 inhibitor treatment. Immunoblots and quantification of individual HiBiT-tagged SARS-CoV-2 viral protein levels shown in Fig. S27B. Proteins were transiently expressed in BEAS-2B cells followed by treatment with increasing concentrations of BC18813. After 18 h viral protein abundance was measured by HiBiT blotting. Representative blots are shown, and data represent mean ± SEM, * $p<0.05$; ** $p<0.01$; *** $p<0.001$; **** $p<0.0001$; as indicated by one-way ANOVA with Dunnett's multiple comparisons (n=2-4).

Figure S29

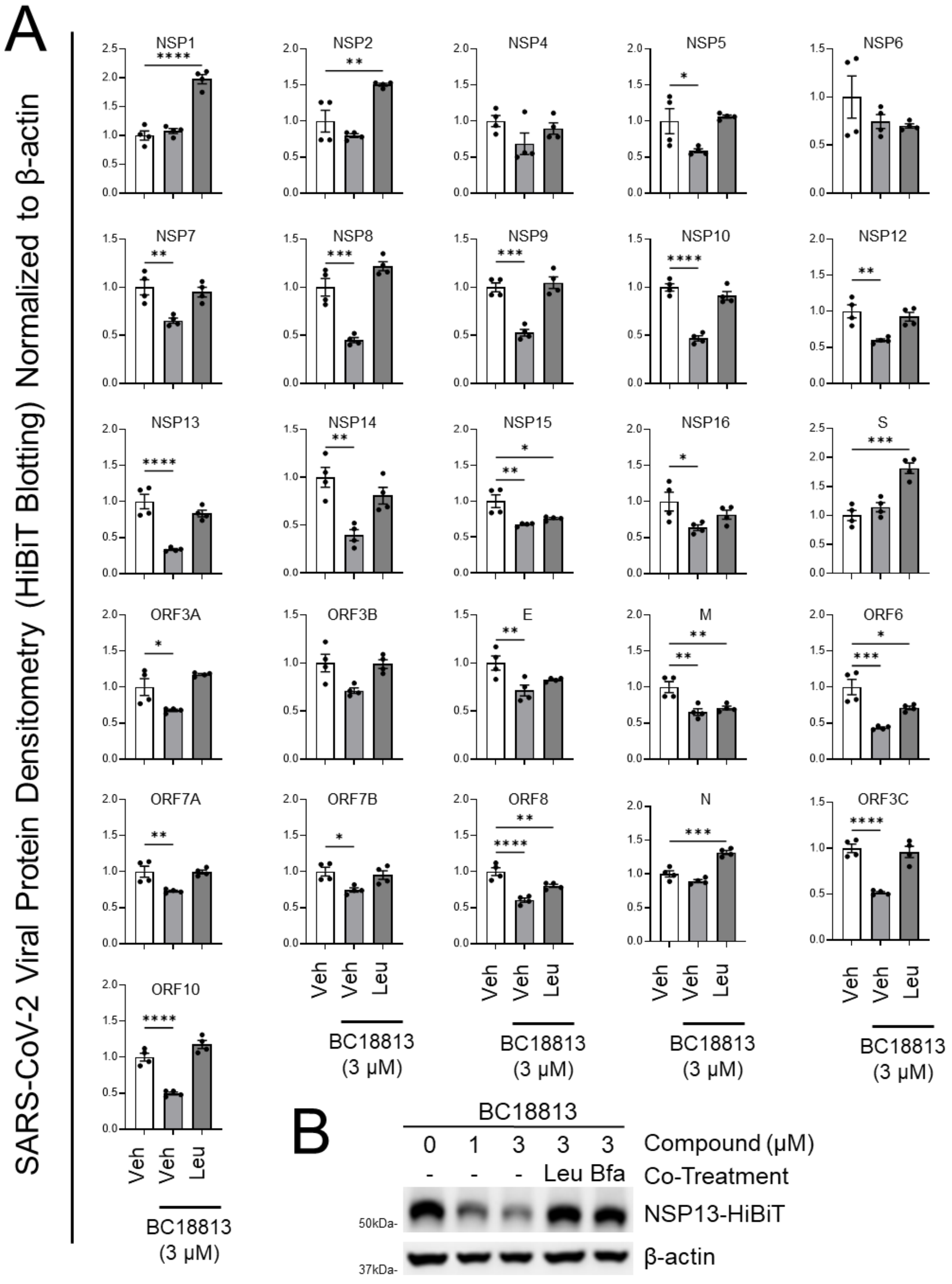
A
SARS-CoV-2 Viral Protein Densitometry (HiBiT Blotting) Normalized to β-actin
NSP1
NSP2
NSP4
NSP5
NSP6
NSP7
NSP8
NSP9
NSP10
NSP12
NSP13
NSP14
NSP15
NSP16
S
ORF3A
ORF3B
E
M
ORF6
ORF7A
ORF7B
ORF8
N
ORF3C
ORF10
Veh
Veh
Leu
BC18813
(3 μM)
B
BC18813
0 1 3 3 3
Compound (μM)
- - - Leu Bfa
Co-Treatment
50kDa-
NSP13-HiBiT
37kDa-
β-actin

Supplementary Figure 29. DCAF7 inhibitor effect on SARS-CoV-2 viral protein stability is lysosomal dependent. A. Quantification of individual HiBiT-tagged SARS-CoV-2 viral protein levels shown in Fig. 7G. Individual HiBiT-tagged SARS-CoV-2 viral proteins were transiently expressed BEAS-2B cells followed by treatment of BC18813 without or with co-treatment of the lysosomal protease inhibitor leupeptin (Leu, 2 μM). After 24 h treatment, viral protein abundance was measured by HiBiT blotting. Data represent mean ± SEM. **B.** Immunoblot analysis of BEAS-2B cells transfected with NSP13-HiBiT and treated with DCAF7 inhibitor dose course (24 h) without or with lysosomal inhibitor treatment using leupeptin (Leu, 2 μM) or bafilomycin A1 (BFA, 0.2 μM). * $p<0.05$; ** $p<0.01$; *** $p<0.001$; **** $p<0.0001$; as indicated by one-way ANOVA with Dunnett's multiple comparisons (n=4).

Figure S30

A

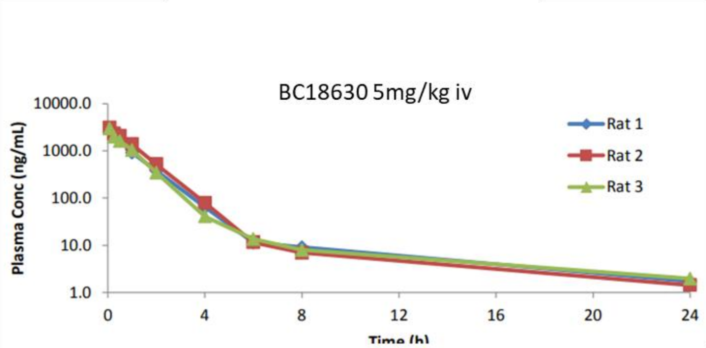

B

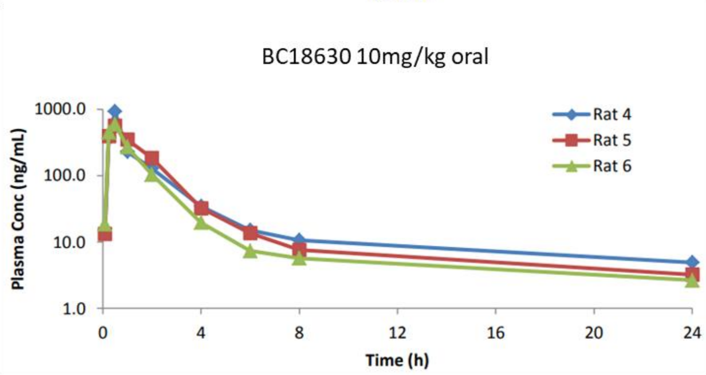

C

Analyte	Matrix	Time (h)	Plasma or Tissue Homogenate Conc (ng/mL)			Mean (ng/ml)	Mean Tissue (ng/g)	SD (ng/ml)	CV (%)	Mean Tissue/Plasma Conc Ratio
BC18630	Plasma	1	4440	4710	3430	**4193**	N/A	675	16.1	N/A
		2	3830	3590	2610	**3343**		646	19.3	
		4	2680	2830	1910	**2473**		494	20.0	
		8	2200	1610	1700	**1837**		318	17.3	
		24	196	170	128	**165**		34.3	20.8	
	Lung	1	2660	3410	2390	**2820**	**11280**	528	18.7	**2.69**
		2	2740	2600	2030	**2457**	**9827**	376	15.3	**2.94**
		4	1920	2170	1720	**1937**	**7747**	225	11.6	**3.13**
		8	1680	1230	1410	**1440**	**5760**	226	15.7	**3.14**
		24	150	124	111	**128**	**513**	19.9	15.5	**3.12**

N/A=not applicable

D

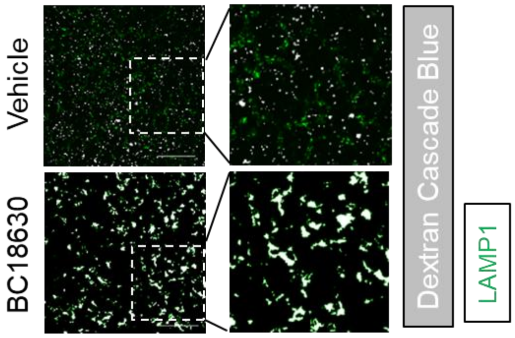

Supplementary Figure 30. BC18630 pharmacokinetics profile and lysosomal efficacy *in vivo*. A-B. Pharmacokinetics of BC18630 in male rats administered at 5 mg/kg intravenously (**A**) or 10 mg/kg through oral gavage (**B**). Data represent plasma concentrations at different time points from individual animals (n=3 per dose/route). **C.** Pharmacokinetic data of BC18630 in C57Bl/6 mice administered 80 mg/kg through oral gavage. Data represent plasma or tissue (lung) concentrations at different time points from different animals (n=3). Tissue/plasma ratio of BC18630 is reported. **D.** Confocal imaging of mouse hepatic tissue following treatment with vehicle or BC18630 (i.p. 50 mg/kg for 4 days). 2 h after the last dose, mice were i.v. injected with Dextran cascade blue, a fluorescent dye that accumulates in the hepatic endo-lysosomal compartment. Liver samples were fixed, embedded, sectioned, and prepared for immunofluorescence detection of lysosomal abundance by LAMP1 staining.

Figure S31

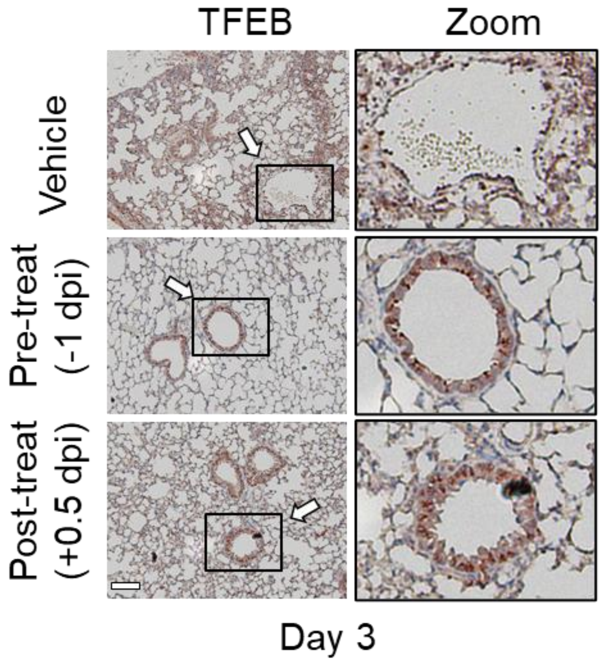

Supplementary Figure 31. BC18630 treatment increases immunoreactive lung TFEB signal during *in vivo* model of SARS-CoV-2 infection. Immunohistochemical staining of host TFEB protein level in lung tissue of infected mice on day 3. Arrows indicate TFEB staining in airways. Scale bar = 100 μm.

Figure S32

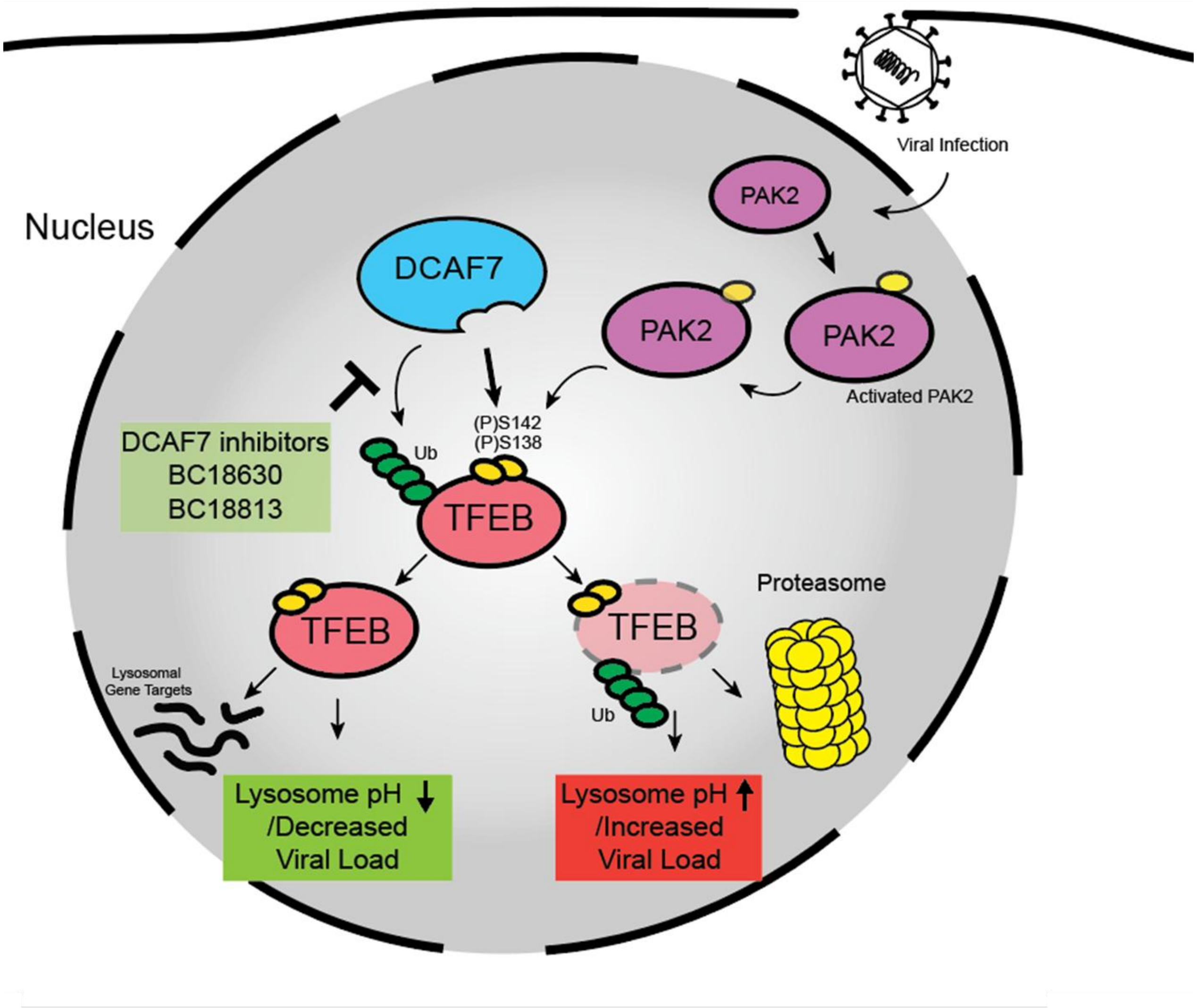

Supplementary Figure 32. Schematic of proposed model. Viral infection potentiates PAK2 kinase that then phosphorylates TFEB to create a phospho-degron. Phosphorylated TFEB is then recognized as a substrate for the CRL4-DCAF7 E3 ligase complex, in which the DCAF7 E3 subunit facilitates the ubiquitination and subsequent proteasomal degradation of TFEB. Chemical inhibition of DCAF7 by small molecules preserves TFEB protein, and increases expression of key TFEB transcription targets, thus enhancing lysosomal biogenesis and activity. Inhibiting pathogen-induced TFEB protein degradation maintains lysosomal activity including lysosomal acidification thereby limiting viral infection.

Supplemental Table 1. Mass Spectrometry Determination of TFEB Post-translation modification. BEAS-2B cells were treated with vehicle or the proteasomal inhibitor MG132 for 4 h before collection and processing for mass spectrometry determination of TFEB phosphorylation status. Inhibition of TFEB proteasomal degradation revealed several modified serine residues that may serve as phospho-degron signals for ubiquitination. Note, S138 and S142 were only observed in the presence of MG132.

	Site	Modification	Best A score	Localization Probability
Vehicle	S122	Phosphorylation	120.35	1
Carfilzomib	S109	Phosphorylation	37.16	0.548994
	S114	Phosphorylation	42.94	0.999949
	S122	Phosphorylation	120.35	1
	S138	Phosphorylation	17.99	0.993003
	S142	Phosphorylation	17.99	0.993003
	S332	Phosphorylation	20.19	0.980938

Supplemental Table 2. Panlabs off targeting of DCAF7 inhibitors.

Compound	Target Class	Assay Name	Assay Target	Mode	Result Type	Value Prefix	RC50 (µM)	Hill	Curve Bottom	Curve Top	Max Response
BC18630	GPCR	Calcium Flux	ADORA2A	Agonist	EC50	>	10				3.09
BC18630	GPCR	Calcium Flux	ADRA1A	Agonist	EC50	>	10				0
BC18630	GPCR	Calcium Flux	AVPR1A	Agonist	EC50	>	10				8.97
BC18630	GPCR	Calcium Flux	CCKAR	Agonist	EC50	>	10				1.69
BC18630	GPCR	Calcium Flux	CHRM1	Agonist	EC50	>	10				0
BC18630	GPCR	Calcium Flux	CHRM3	Agonist	EC50	>	10				0
BC18630	GPCR	Calcium Flux	EDNRA	Agonist	EC50	>	10				0
BC18630	GPCR	Calcium Flux	HRH1	Agonist	EC50	>	10				0
BC18630	GPCR	Calcium Flux	HTR2A	Agonist	EC50	>	10				4.44
BC18630	GPCR	Calcium Flux	HTR2B	Agonist	EC50	>	10				5.41
BC18630	GPCR	Calcium Flux	ADORA2A	Antagonist	IC50	>	10				29.81
BC18630	GPCR	Calcium Flux	ADRA1A	Antagonist	IC50	>	10				0
BC18630	GPCR	Calcium Flux	AVPR1A	Antagonist	IC50	>	10				15.91
BC18630	GPCR	Calcium Flux	CCKAR	Antagonist	IC50	>	10				0
BC18630	GPCR	Calcium Flux	CHRM1	Antagonist	IC50	>	10				7.76
BC18630	GPCR	Calcium Flux	CHRM3	Antagonist	IC50	>	10				13.68
BC18630	GPCR	Calcium Flux	EDNRA	Antagonist	IC50	>	10				3.72
BC18630	GPCR	Calcium Flux	HRH1	Antagonist	IC50	>	10				0
BC18630	GPCR	Calcium Flux	HTR2A	Antagonist	IC50	>	10				22.52
BC18630	GPCR	Calcium Flux	HTR2B	Antagonist	IC50	>	10				9.48
BC18630	GPCR	cAMP	ADRA2A	Agonist	EC50	>	10				0
BC18630	GPCR	cAMP	ADRB1	Agonist	EC50	>	10				0.54
BC18630	GPCR	cAMP	ADRB2	Agonist	EC50	>	10				0
BC18630	GPCR	cAMP	CHRM2	Agonist	EC50	>	10				31.64
BC18630	GPCR	cAMP	CNR1	Agonist	EC50	>	10				0.8
BC18630	GPCR	cAMP	CNR2	Agonist	EC50	>	10				27.82
BC18630	GPCR	cAMP	DRD1	Agonist	EC50	>	10				0
BC18630	GPCR	cAMP	DRD2S	Agonist	EC50	>	10				0.87
BC18630	GPCR	cAMP	HRH2	Agonist	EC50	>	10				0
BC18630	GPCR	cAMP	HTR1A	Agonist	EC50	>	10				9.36
BC18630	GPCR	cAMP	HTR1B	Agonist	EC50	>	10				12.5
BC18630	GPCR	cAMP	OPRD1	Agonist	EC50	>	10				42.59
BC18630	GPCR	cAMP	OPRK1	Agonist	EC50	>	10				8.98
BC18630	GPCR	cAMP	OPRM1	Agonist	EC50	>	10				8.65

BC18630	GPCR	cAMP	ADRA2A	Antagonist	IC50	>	10				0
BC18630	GPCR	cAMP	ADRB1	Antagonist	IC50	>	10				44.83
BC18630	GPCR	cAMP	ADRB2	Antagonist	IC50	>	10				37.28
BC18630	GPCR	cAMP	CHRM2	Antagonist	IC50	>	10				0.25
BC18630	GPCR	cAMP	CNR1	Antagonist	IC50	>	10				26.85
BC18630	GPCR	cAMP	CNR2	Antagonist	IC50	>	10				0
BC18630	GPCR	cAMP	DRD1	Antagonist	IC50	=	8.4454	1.45	0	100	55.07
BC18630	GPCR	cAMP	DRD2S	Antagonist	IC50	>	10				24.29
BC18630	GPCR	cAMP	HRH2	Antagonist	IC50	=	6.009	1	-8.58	73.68	43.2
BC18630	GPCR	cAMP	HTR1A	Antagonist	IC50	>	10				5.7
BC18630	GPCR	cAMP	HTR1B	Antagonist	IC50	>	10				5.74
BC18630	GPCR	cAMP	OPRD1	Antagonist	IC50	>	10				9.94
BC18630	GPCR	cAMP	OPRK1	Antagonist	IC50	>	10				1
BC18630	GPCR	cAMP	OPRM1	Antagonist	IC50	>	10				49.35
BC18630	Ion Channel	Ion Channel	CAV1.2	Blocker	IC50	>	10				0
BC18630	Ion Channel	Ion Channel	GABAA	Blocker	IC50	>	10				27.61
BC18630	Ion Channel	Ion Channel	hERG	Blocker	IC50	>	10				6.14
BC18630	Ion Channel	Ion Channel	HTR3A	Blocker	IC50	=	6.4306	1.9	0	100	69.94
BC18630	Ion Channel	Ion Channel	KvLQT1/minK	Blocker	IC50	>	10				21.38
BC18630	Ion Channel	Ion Channel	nAChR(a4/b2)	Blocker	IC50	>	10				12.42
BC18630	Ion Channel	Ion Channel	NAV1.5	Blocker	IC50	>	10				29.98
BC18630	Ion Channel	Ion Channel	NMDAR (1A/2B)	Blocker	IC50	>	10				0
BC18630	Ion Channel	Ion Channel	GABAA	Opener	EC50	>	10				0.97
BC18630	Ion Channel	Ion Channel	HTR3A	Opener	EC50	>	10				0
BC18630	Ion Channel	Ion Channel	KvLQT1/minK	Opener	EC50	>	10				6.18
BC18630	Ion Channel	Ion Channel	nAChR (a4/b2)	Opener	EC50	>	10				0
BC18630	Ion Channel	Ion Channel	NMDAR (1A/2B)	Opener	EC50	>	10				0
BC18630	Kinases	Binding	INSR	Inhibitor	IC50	>	10				15.58
BC18630	Kinases	Binding	LCK	Inhibitor	IC50	>	10				49.1
BC18630	Kinases	Binding	ROCK1	Inhibitor	IC50	>	10				20.77
BC18630	Kinases	Binding	VEGFR2	Inhibitor	IC50	>	10				13.66
BC18630	NHR	NHR Nuclear Translocation	AR	Agonist	EC50	>	10				0
BC18630	NHR	NHR Nuclear Translocation	AR	Antagonist	IC50	>	10				15.63
BC18630	NHR	NHR Protein Interaction	GR	Agonist	EC50	>	10				0
BC18630	NHR	NHR Protein Interaction	GR	Antagonist	IC50	>	10				0
BC18630	Non-Kinase Enzymes	Enzymatic	AChE	Inhibitor	IC50	>	10				21.24
BC18630	Non-Kinase Enzymes	Enzymatic	COX1	Inhibitor	IC50	>	10				21.8

BC18630	Non-Kinase Enzymes	Enzymatic	COX2	Inhibitor	IC50	>	10				8.53
BC18630	Non-Kinase Enzymes	Enzymatic	MAOA	Inhibitor	IC50	>	10				57.04
BC18630	Non-Kinase Enzymes	Enzymatic	PDE3A	Inhibitor	IC50	>	10				8.9
BC18630	Non-Kinase Enzymes	Enzymatic	PDE4D2	Inhibitor	IC50	>	10				52.31
BC18630	Transporter	Transporter	DAT	Blocker	IC50	>	10				37.03
BC18630	Transporter	Transporter	NET	Blocker	IC50	>	10				0
BC18630	Transporter	Transporter	SERT	Blocker	IC50	>	10				0

Compound	Target Class	Assay Name	Assay Target	Mode	Result Type	Value Prefix	RC50 (µM)	Hill	Curve Bottom	Curve Top	Max Response
BC18813	GPCR	Calcium Flux	ADORA2A	Agonist	EC50	>	10				1.05
BC18813	GPCR	Calcium Flux	ADRA1A	Agonist	EC50	>	10				5.5
BC18813	GPCR	Calcium Flux	AVPR1A	Agonist	EC50	>	10				1.07
BC18813	GPCR	Calcium Flux	CCKAR	Agonist	EC50	>	10				2.97
BC18813	GPCR	Calcium Flux	CHRM1	Agonist	EC50	>	10				0
BC18813	GPCR	Calcium Flux	CHRM3	Agonist	EC50	>	10				0.56
BC18813	GPCR	Calcium Flux	EDNRA	Agonist	EC50	>	10				5.5
BC18813	GPCR	Calcium Flux	HRH1	Agonist	EC50	>	10				1.49
BC18813	GPCR	Calcium Flux	HTR2A	Agonist	EC50	>	10				0.95
BC18813	GPCR	Calcium Flux	HTR2B	Agonist	EC50	>	10				2.91
BC18813	GPCR	Calcium Flux	ADORA2A	Antagonist	IC50	>	10				37.71
BC18813	GPCR	Calcium Flux	ADRA1A	Antagonist	IC50	>	10				1.12
BC18813	GPCR	Calcium Flux	AVPR1A	Antagonist	IC50	>	10				15.97
BC18813	GPCR	Calcium Flux	CCKAR	Antagonist	IC50	>	10				0
BC18813	GPCR	Calcium Flux	CHRM1	Antagonist	IC50	>	10				15.26
BC18813	GPCR	Calcium Flux	CHRM3	Antagonist	IC50	>	10				16.56
BC18813	GPCR	Calcium Flux	EDNRA	Antagonist	IC50	>	10				2.32
BC18813	GPCR	Calcium Flux	HRH1	Antagonist	IC50	>	10				6.52
BC18813	GPCR	Calcium Flux	HTR2A	Antagonist	IC50	>	10				25.6
BC18813	GPCR	Calcium Flux	HTR2B	Antagonist	IC50	>	10				31.33
BC18813	GPCR	cAMP	ADRA2A	Agonist	EC50	>	10				38.75
BC18813	GPCR	cAMP	ADRB1	Agonist	EC50	>	10				2.22
BC18813	GPCR	cAMP	ADRB2	Agonist	EC50	>	10				0
BC18813	GPCR	cAMP	CHRM2	Agonist	EC50	>	10				47.69
BC18813	GPCR	cAMP	CNR1	Agonist	EC50	=	6.114	1.07	0	100	60.97
BC18813	GPCR	cAMP	CNR2	Agonist	EC50	=	4.592	1.05	0	100	63.59
BC18813	GPCR	cAMP	DRD1	Agonist	EC50	>	10				0

BC18813	GPCR	cAMP	DRD2S	Agonist	EC50	>	10				13.51
BC18813	GPCR	cAMP	HRH2	Agonist	EC50	>	10				0
BC18813	GPCR	cAMP	HTR1A	Agonist	EC50	>	10				12.97
BC18813	GPCR	cAMP	HTR1B	Agonist	EC50	>	10				27.35
BC18813	GPCR	cAMP	OPRD1	Agonist	EC50	>	10				54.84
BC18813	GPCR	cAMP	OPRK1	Agonist	EC50	>	10				24.29
BC18813	GPCR	cAMP	OPRM1	Agonist	EC50	>	10				24.43
BC18813	GPCR	cAMP	ADRA2A	Antagonist	IC50	>	10				0
BC18813	GPCR	cAMP	ADRB1	Antagonist	IC50	>	10				42.89
BC18813	GPCR	cAMP	ADRB2	Antagonist	IC50	>	10				41.94
BC18813	GPCR	cAMP	CHRM2	Antagonist	IC50	>	10				12.25
BC18813	GPCR	cAMP	CNR1	Antagonist	IC50	>	10				0
BC18813	GPCR	cAMP	CNR2	Antagonist	IC50	>	10				0
BC18813	GPCR	cAMP	DRD1	Antagonist	IC50	=	9.454	1.19	0	100	50.15
BC18813	GPCR	cAMP	DRD2S	Antagonist	IC50	>	10				5.93
BC18813	GPCR	cAMP	HRH2	Antagonist	IC50	>	10				45.81
BC18813	GPCR	cAMP	HTR1A	Antagonist	IC50	>	10				0
BC18813	GPCR	cAMP	HTR1B	Antagonist	IC50	>	10				0.13
BC18813	GPCR	cAMP	OPRD1	Antagonist	IC50	>	10				0
BC18813	GPCR	cAMP	OPRK1	Antagonist	IC50	>	10				0
BC18813	GPCR	cAMP	OPRM1	Antagonist	IC50	=	7.462	1.01	-9.93	100	53.32
BC18813	Ion Channel	Ion Channel	CAV1.2	Blocker	IC50	>	10				0
BC18813	Ion Channel	Ion Channel	GABAA	Blocker	IC50	>	10				19.98
BC18813	Ion Channel	Ion Channel	hERG	Blocker	IC50	>	10				4.86
BC18813	Ion Channel	Ion Channel	HTR3A	Blocker	IC50	>	10				0
BC18813	Ion Channel	Ion Channel	KvLQT1/minK	Blocker	IC50	>	10				12.95
BC18813	Ion Channel	Ion Channel	nAChR(a4/b2)	Blocker	IC50	>	10				0
BC18813	Ion Channel	Ion Channel	NAV1.5	Blocker	IC50	>	10				8.29
BC18813	Ion Channel	Ion Channel	NMDAR (1A/2B	Blocker	IC50	>	10				13.85
BC18813	Ion Channel	Ion Channel	GABAA	Opener	EC50	>	10				0.77
BC18813	Ion Channel	Ion Channel	HTR3A	Opener	EC50	>	10				0.03
BC18813	Ion Channel	Ion Channel	KvLQT1/minK	Opener	EC50	>	10				3.89
BC18813	Ion Channel	Ion Channel	nAChR(a4/b2)	Opener	EC50	>	10				0
BC18813	Ion Channel	Ion Channel	NMDAR (1A/2B	Opener	EC50	>	10				0
BC18813	Kinases	Binding	INSR	Inhibitor	IC50	>	10				0
BC18813	Kinases	Binding	LCK	Inhibitor	IC50	>	10				11.15
BC18813	Kinases	Binding	ROCK1	Inhibitor	IC50	>	10				22.8
BC18813	Kinases	Binding	VEGFR2	Inhibitor	IC50	>	10				0.27
BC18813	NHR	NHR Nuclear Translocation	AR	Agonist	EC50	>	10				0

BC18813	NHR	NHR Nuclear Translocation	AR	Antagonist	IC50	>	10				0
BC18813	NHR	NHR Protein Interaction	GR	Agonist	EC50	>	10				4.56
BC18813	NHR	NHR Protein Interaction	GR	Antagonist	IC50	>	10				7.56
BC18813	Non-Kinase Enzymes	Enzymatic	AChE	Inhibitor	IC50	>	10				6.26
BC18813	Non-Kinase Enzymes	Enzymatic	COX1	Inhibitor	IC50	>	10				29.33
BC18813	Non-Kinase Enzymes	Enzymatic	COX2	Inhibitor	IC50	>	10				17.86
BC18813	Non-Kinase Enzymes	Enzymatic	MAOA	Inhibitor	IC50	>	10				22.22
BC18813	Non-Kinase Enzymes	Enzymatic	PDE3A	Inhibitor	IC50	>	10				28.22
BC18813	Non-Kinase Enzymes	Enzymatic	PDE4D2	Inhibitor	IC50	>	10				44.38
BC18813	Transporter	Transporter	DAT	Blocker	IC50	>	10				16.84
BC18813	Transporter	Transporter	NET	Blocker	IC50	>	10				9.94
BC18813	Transporter	Transporter	SERT	Blocker	IC50	>	10				0

Supplemental Table 3. Antibody and Resources

REAGENT or RESOURCE	SOURCE	IDENTIFIER
**Antibodies**		
β-actin	Invitrogen	Cat# MA5-15739, RRID:AB_10979409
GFP (4B10)	Cell Signaling Technologies	Cat# 2955, RRID:AB_1196614
OC43 (541-8F)	EMD Millipore	Cat# MAB9012, RRID:AB_95424
TFEB	Cell Signaling Technologies	Cat# 4240, RRID:AB_11220225
TFEB	Bethyl Laboratories	Cat# A303-673A, RRID:AB_11204751
TFE3	Cell Signaling Technologies	Cat# 14779, RRID:AB_2687582
MITF	Cell Signaling Technologies	Cat# 12590
TFEC	Abcam	Cat# ab185226
C-Myc	Cell Signaling Technologies	Cat# 9402, RRID:AB_2151827
Phospho TFEB (Ser142)	Sigma-Aldrich	Cat# ABE1971-I, RRID:AB_2928101
Phospho-p70 S6 kinase (Thr-389) (108D2)	Cell Signaling Technologies	Cat# 9234, RRID:AB_2269803
p70 S6 kinase (49D7)	Cell Signaling Technologies	Cat#2708, RRID:AB_390722)
Normal rabbit IgG	Santa Cruz Biotechnology	Cat# sc-2027, RRID:AB_737197
GAPDH Antibody (ZG003)	Invitrogen	Cat# 39-8600, RRID:AB_2533438
HA Tag (2-2.2.14)	Invitrogen	Cat# 26183, RRID:AB_10978021
V5 Tag	Invitrogen	Cat# R960-25, RRID:AB_2556564
K48-linkage Specific Polyubiquitin	Cell Signaling Technologies	Cat# 4289, RRID:AB_10557239
PAK2 (3B5)	Cell Signaling Technologies	Cat# 4825, RRID:AB_10547135
Phospho-PAK2 (Ser20)	Cell Signaling Technologies	Cat# 2607, RRID:AB_2158759
Anti-WDR68 (DCAF7)	Abcam	Cat# ab70148, RRID:AB_1271483
Phospho-Serine	Millipore	Cat# AB1603, RRID:AB_390205
Lamin A/C (4C11)	Cell Signaling Technologies	Cat# 4777, RRID:AB_10545756
Cyclin E (HE111)	Santa Cruz Biotechnology	Cat# sc-248, RRID:AB_627362
Dnmt (H-12)	Santa Cruz Biotechnology	Cat# sc-271729, RRID:AB_10710384
PTEN (A2B1)	Santa Cruz Biotechnology	Cat# sc-7974, RRID:AB_628187
Influenza A Virus Nucleoprotein (C43)	Abcam	Cat# ab128193, RRID:AB_11143769
p21 Waf1/Cip1 (12D1)	Cell Signaling Technologies	Cat# 2947, RRID:AB_823586
SARS-CoV-2 Nucleoprotein	Invitrogen	Cat# MA1-7403, RRID:AB_1018420
TFEB (C-6)	Santa Cruz Biotechnology	Cat# sc-166736, RRID:AB_2255943
DCAF7	Invitrogen	Cat# PA5-54642, RRID:AB_2640409
LAMP1	Novus	Cat# NB100-77683, RRID:AB_1083191
Goat anti-Rat IgG (H+L) Cross-Adsorbed Secondary Antibody, Alexa Fluor 488	Invitrogen	Cat# A-11006, RRID:AB_2534074)
Goat anti-Rabbit IgG (H+L), Superclonal Recombinant Secondary Antibody, Alexa Fluor 647	Invitrogen	Cat# A27040, RRID:AB_2536101
Goat anti-Rabbit IgG (H+L) HRP	Invitrogen	Cat# 31460, RRID:AB_228341
Goat anti-Mouse IgG (H+L) HRP	Invitrogen	Cat# 31430, RRID:AB_228307
**Bacterial and Virus Strains**		
TOP10	Invitrogen	C404010
Pseudomonas aeruginosa	ATCC	Strain PA103
Staphylococcus aureus	ATCC	Strain 29213
Stbl3	Invitrogen	C737303
Human Betacoronavirus 1 (OC43)	ATCC	VR-1558
Human coronavirus 229E	ATCC	VR-740
SARS-CoV-2 (in vitro)	IIT Research Institute (Chicago, USA)	USA-WA1/2020

SARS-CoV-2 (in vitro)	Viroclinics (Rotterdam, The Netherlands)	USA-WA1/2020
SARS-CoV-2 (in vitro) Alpha	Viroclinics (Rotterdam, The Netherlands)	Isolate USA/CA_CDC_5574/2020 (Alpha)
SARS-CoV-2 (in vitro) Beta	Viroclinics (Rotterdam, The Netherlands)	Isolate hCoV-19/South Africa/KRISP-K005325/2020 (Beta)
SARS-CoV-2 (in vitro) Delta	Viroclinics (Rotterdam, The Netherlands)	Isolate hCoV-19/USA/PHC658/2021 (Lineage B.1.617.2; Delta Variant)
SARS-CoV-2 (in vivo)	ChemDIV (San Diego, USA)	Victoria/1/2020
SARS-CoV-2 (in vivo)	BEI Resources ATCC	NR-52281
**Chemicals, Peptides, and Recombinant Proteins**		
FDA-Approved Compound Library	Selleck	L1300
BC1753	ChemDiv	
BC18813	This Study	
BC18630	This Study	
Carfilzomib	Cayman Chemical	17554
Leupeptin	Cayman Chemical	14026
MG132	UBPBio	F1101
Bafilomycin A1	Cayman Chemical	11038
LysoTracker Red DND-99	Invitrogen	L7528
CellTracker Green CMFDA	Invitrogen	C2925
Lysosensor Yellow/Blue DND-160	Invitrogen	L7545
Hoechst 33342	Invitrogen	H3570
DMEM/F-12	Gibco	11320082
EMEM	Gibco	670086
Fetal Bovine Serum	Gibco	26140079
Opti-MEM I Reduced Serum Medium	Gibco	31985062
Ubiquitination kit	Enzo	BML-UW9920-0001
Recombinant Human CUL4A/NEDD8/RBX1	R&D Systems	E3-441
ProQinase™ PAK2	Reaction Biology	0304-0000-1
Phos-tag Biotin Probe	FujiFilm	BTL-104
Critical Commercial Assays		
Kinase esiRNA library (Custom)	MiliporeSigma	
pcDNA3.1 Directional TOPO Expression Kit	Invitrogen	K490001
Duolink In Situ Orange Starter Kit Mouse/Rabbit	MilliporeSigma	DUO92102
Dynabeads His-Tag Isolation and Pulldown	Invitrogen	10103D
Pierce Protein A/G Magnetic Beads	Thermo Scientific	88802
High-Capacity cDNA Reverse Transcription Kit	Applied Biosystems	4368814
SYBR Green PCR Master Mix	Applied Biosystems	4364344
In-Fusion Cloning	Takara	638910
Gateway Cloning	ThermoFisher	11791019
Phusion polymerase	New England Biolabs	M0530
Quick CIP	New England Biolabs	M0525
Quick Ligation Kit	New England Biolabs	M2200
Easy Prep RNA Miniprep Plus Kit	Bioland Scientific	R01-04
RNeasy Plus Mini Kit	Qiagen	74134
DC Protein Assay Reagent A/B/S	BioRad	500-0113/ 0114/ 0115
GFP-Trap Magnetic Agarose	ChromoTek	gtma-20
NE-PER Nuclear and Cytoplasmic Extraction Reagents	Thermo Scientific	78833
QuikChange II XL Site-Directed Mutagenesis Kit	Aglient	200521
X-tremeGENE siRNA Transfection Reagent	Sigma-Aldrich	4476115001
X-tremeGENE HP DNA Transfection Reagent	Sigma-Aldrich	6366244001
Lipofectamine 3000 Transfection Reagents	Invitrogen	L3000015
MagicRed Cathepsin B activity assay	ImmunoChemistry Technologies	937
CellTiter-Glo 2.0 Cell Viability Assay	Promega	G9243
TnT Quick Coupled Transcription/Translation kit	Promega	L1170
TUBE 2 Magnetic Beads	LifeSensors	UM402M
High Sensitivity Carboxyl Sensors SPR	Nicoya	SEN-HS-8-COOH
Amine Coupling Kit	Nicoya	AMINE-10
Experimental Models: Cell Lines		
MLE-12	ATCC	CRL-2110

BEAS-2B	ATCC	CRL-9609
BEAS-2B TFEB-EGFP stable	This Study	
BEAS-2B DCAF7 KO	This Study	
BEAS-2B DCAF7 KO, Re-expressed DCAF7	This Study	
BEAS-2B PAK2 KO	This Study	
BEAS-2B GFP-LC3	This Study	
HEK293A GFP-LC3 cell	Sigma	14050801
MRC-5	ATCC	CCL-171
HCT-8	ATCC	CCL-244
HEK293T	ATCC	CRL-1573
Calu-3	ATCC	HTB-55
Experimental Models: Organisms/Strains		
C57BL/6J male mice	Jackson Laboratories	
B6.Cg-Tg(K18-ACE2)2Prlmn/J	Jackson Laboratories	#:034860; RRID:IMSR_JAX:034860
Sprague-Dawley rats	Touchstone Biosciences (in house)	
Syrian hamsters	ChemDIV (in house)	
**Recombinant DNA**		
Item	Source	Identifier
pLENTI-c-mGFP-P2A-Puro	Origene	PS100093
WT-DCAF7, pcDNA3.1D-V5-HIS	This study	
WT-DCAF7, pcDNA3.1D-HA	This study	
WT-TFEB, pcDNA3.1D-V5-HIS	This study	
Δ1-115 TFEB, pcDNA3.1D-V5-HIS	This study	
Δ256-476 TFEB, pcDNA3.1D-V5-HIS	This study	
Δ310-476 TFEB, pcDNA3.1D-V5-HIS	This study	
Δ290-476 TFEB, pcDNA3.1D-V5-HIS	This study	
Δ274-476 TFEB, pcDNA3.1D-V5-HIS	This study	
S138A TFEB, pcDNA3.1D-V5-HIS	This study	
S142A TFEB, pcDNA3.1D-V5-HIS	This study	
K232R TFEB, pcDNA3.1D-V5-HIS	This study	
S138A/S138A TFEB, pcDNA3.1D-V5-HIS	This study	
WT-TFEB, pEGFP-N1	Plasmid was a gift from Shawn Ferguson (*106*)	Addgene plasmid: 38119
S138A TFEB, pEGFP-N1	This study	
S142A TFEB, pEGFP-N1	This study	
S138A/S138A TFEB, pEGFP-N1	This study	
lentiCRISPR v2	Plasmid was a gift from Feng Zhang (*88*)	Addgene plasmid: 52961
psPAX2	Plasmid was a gift from Didier Trono	Addgene plasmid: 12260
pMD2.G	Plasmid was a gift from Didier Trono	Addgene plasmid: 12259
LAMP1-mGFP	Plasmid was a gift from Esteban Dell'Angelica (*107*)	Addgene plasmid: 34831
pLVX-EF1alpha-SARS-CoV-2-nsp1-2xStrep-IRES-Puro	Plasmid was a gift from Nevan Krogan (*66*)	Addgene plasmid: 141367
pLVX-EF1alpha-SARS-CoV-2-nsp2-2xStrep-IRES-Puro	Plasmid was a gift from Nevan Krogan (*66*)	Addgene plasmid: 141368
pLVX-EF1alpha-SARS-CoV-2-nsp4-2xStrep-IRES-Puro	Plasmid was a gift from Nevan Krogan (*66*)	Addgene plasmid: 141369
pLVX-EF1alpha-SARS-CoV-2-nsp5-2xStrep-IRES-Puro	Plasmid was a gift from Nevan Krogan (*66*)	Addgene plasmid: 141370
pDONR223 SARS-CoV-2 NSP6	Plasmid was a gift from Fritz Roth (*109*)	Addgene plasmid: 141260
pLVX-EF1alpha-SARS-CoV-2-nsp7-2xStrep-IRES-Puro	Plasmid was a gift from Nevan Krogan (*66*)	Addgene plasmid: 141373
pLVX-EF1alpha-SARS-CoV-2-nsp8-2xStrep-IRES-Puro	Plasmid was a gift from Nevan Krogan (*66*)	Addgene plasmid: 141374
pLVX-EF1alpha-SARS-CoV-2-nsp9-2xStrep-IRES-Puro	Plasmid was a gift from Nevan Krogan (*66*)	Addgene plasmid: 141375
pLVX-EF1alpha-SARS-CoV-2-nsp10-2xStrep-IRES-Puro	Plasmid was a gift from Nevan Krogan (*66*)	Addgene plasmid: 141376
pLVX-EF1alpha-SARS-CoV-2-nsp12-2xStrep-IRES-Puro	Plasmid was a gift from Nevan Krogan (*66*)	Addgene plasmid: 141378
pLVX-EF1alpha-SARS-CoV-2-nsp13-2xStrep-IRES-Puro	Plasmid was a gift from Nevan Krogan (*66*)	Addgene plasmid: 141379
pLXV-EF1alpha-2xStrep-SARS-CoV-2-nsp14-IRES-Puro	Plasmid was a gift from Nevan Krogan (*66*)	Addgene plasmid: 141380

pLVX-EF1alpha-SARS-CoV-2-nsp15-2xStrep-IRES-Puro	Plasmid was a gift from Nevan Krogan (*66*)	Addgene plasmid: 141381
pDONR223 SARS-CoV-2 NSP16	Plasmid was a gift from Fritz Roth (*109*)	Addgene plasmid: 141269
SARS-CoV-2 (2019-nCoV) Spike ORF mammalian expression plasmid (Codon Optimized)	Sinobiological	VG40589UT
pLVX-EF1alpha-SARS-CoV-2-orf3a-2xStrep-IRES-Puro	Plasmid was a gift from Nevan Krogan (*66*)	Addgene plasmid: 141383
pLXV-EF1alpha-2xStrep-SARS-CoV-2-orf3b-IRES-Puro	Plasmid was a gift from Nevan Krogan (*66*)	Addgene plasmid: 141384
pLVX-EF1alpha-SARS-CoV-2-E-2xStrep-IRES-Puro	Plasmid was a gift from Nevan Krogan (*66*)	Addgene plasmid: 141385
pLVX-EF1alpha-SARS-CoV-2-M-2xStrep-IRES-Puro	Plasmid was a gift from Nevan Krogan (*66*)	Addgene plasmid: 141386
pLVX-EF1alpha-SARS-CoV-2-orf6-2xStrep-IRES-Puro	Plasmid was a gift from Nevan Krogan (*66*)	Addgene plasmid: 141387
pLVX-EF1alpha-SARS-CoV-2-orf7a-2xStrep-IRES-Puro	Plasmid was a gift from Nevan Krogan (*66*)	Addgene plasmid: 141388
pLXV-EF1alpha-2xStrep-SARS-CoV-2-orf7b-IRES-Puro	Plasmid was a gift from Nevan Krogan (*66*)	Addgene plasmid: 141389
pLVX-EF1alpha-SARS-CoV-2-orf8-2xStrep-IRES-Puro	Plasmid was a gift from Nevan Krogan (*66*)	Addgene plasmid: 141390
pLVX-EF1alpha-SARS-CoV-2-N-2xStrep-IRES-Puro	Plasmid was a gift from Nevan Krogan (*66*)	Addgene plasmid: 141391
pLXV-EF1alpha-2xStrep-SARS-CoV-2-orf9c-IRES-Puro	Plasmid was a gift from Nevan Krogan (*66*)	Addgene plasmid: 141393
pLVX-EF1alpha-SARS-CoV-2-orf10-2xStrep-IRES-Puro	Plasmid was a gift from Nevan Krogan (*66*)	Addgene plasmid: 141394
SARS-CoV-2 NSP1 in pFC37K-HiBiT	This Study	
SARS-CoV-2 NSP2 in pFC37K-HiBiT	This Study	
SARS-CoV-2 NSP4 in pFC37K-HiBiT	This Study	
SARS-CoV-2 NSP5 in pFC37K-HiBiT	This Study	
SARS-CoV-2 NSP6 in pFC37K-HiBiT	This Study	
SARS-CoV-2 NSP7 in pFC37K-HiBiT	This Study	
SARS-CoV-2 NSP8 in pFC37K-HiBiT	This Study	
SARS-CoV-2 NSP9 in pFC37K-HiBiT	This Study	
SARS-CoV-2 NSP10 in pFC37K-HiBiT	This Study	
SARS-CoV-2 NSP12 in pFC37K-HiBiT	This Study	
SARS-CoV-2 NSP13 in pFC37K-HiBiT	This Study	
SARS-CoV-2 NSP14 in pFC37K-HiBiT	This Study	
SARS-CoV-2 NSP15 in pFC37K-HiBiT	This Study	
SARS-CoV-2 NSP16 in pFC37K-HiBiT	This Study	
SARS-CoV-2 Spike in pFC37K-HiBiT	This Study	
SARS-CoV-2 ORF3A in pFC37K-HiBiT	This Study	
SARS-CoV-2 ORF3B in pFC37K-HiBiT	This Study	
SARS-CoV-2 E in pFC37K-HiBiT	This Study	
SARS-CoV-2 M in pFC37K-HiBiT	This Study	
SARS-CoV-2 ORF6 in pFC37K-HiBiT	This Study	
SARS-CoV-2 ORF7A in pFC37K-HiBiT	This Study	
SARS-CoV-2 ORF7B in pFC37K-HiBiT	This Study	
SARS-CoV-2 ORF8 in pFC37K-HiBiT	This Study	
SARS-CoV-2 N in pFC37K-HiBiT	This Study	
SARS-CoV-2 ORF9C in pFC37K-HiBiT	This Study	
SARS-CoV-2 ORF10 in pFC37K-HiBiT	This Study	
Software and Algorithms		
Fiji ImageJ	(*93*)	
Graphpad	Prism	9.0
Gen5	Biotek	3.0
Discovery Studio	BIOVIA	3.5
CellProfiler	(*92*)	3.0
BioRender		

Supplemental Table 4. Oligonucleotides

Oligonucleotides	Sequence	Source
Ctrl siRNA	Control DsiRNA Negative Control (DS NC1)<br>rCrGrUrUrArArUrCrGrCrGrUrArUrArArUrArCrGrCrGrUAT<br>rArUrArCrGrCrGrUrArUrUrArUrArCrGrCrGrArUrUrArArCrGrArC	IDT
DCAF7 siRNA	hs.Ri.DCAF7.13.1<br>rCrCrUrCrArArGrCrArUrUrGrArUrArCrGrArCrArUrGrCAC<br>rGrUrGrCrArUrGrUrCrGrUrArUrCrArArUrGrCrUrUrGrArGrGrUrA	IDT
PAK2 siRNA	hs.Ri.PAK2.13.1<br>rGrGrUrGrArGrCrUrCrUrGrArUrArCrArGrUrGrArGrArAGA<br>rUrCrUrUrCrUrCrArCrUrGrUrArUrCrArGrArGrCrUrCrArCrCrArA	IDT
DCAF7 sgRNA	GCGGTGACTATCTCCGTGTG	IDT
PAK2 sgRNA	GTGTGCTCAAAATCAGATGG	IDT
MISSION® siRNA Human Gene Family Set	Kinase panel, 719 targets from Sigma-Aldrich	SI02100
WT TFEB in pcDNA3.1D-V5-HIS	Fwd: caccATGGCGTCACGCATAGGGT<br>Rev: CAGCACATCGCCCTCCTC	
TFEB S138A	Fwd: CTCCGCTGGCAACGCTGCTCCCAATAG<br>Rev: CTATTGGGAGCAGCGTTGCCAGCGGAG	IDT
TFEB S142A	Fwd: CAACAGTGCTCCCAATGCACCCATGGCCATGCTGC<br>Rev: GCAGCATGGCCATGGGTGCATTGGGAGCACTGTTG	IDT
TFEB S138A/S142A	Fwd: CTCCGCTGGCAACGCAGCTCCCAATGCAC (using S142A as template)<br>Rev: GTGCATTGGGAGCTGCGTTGCCAGCGGAG (using S142A as template)	IDT
TFEB to K232R	Fwd: GGCCCTGGCCAGAGAGCGGCAGAAGAAAGACAAT<br>Rev: ATTGTCTTTCTTCTGCCGCTCTCTGGCCAGGGCC	IDT
Δ1-115 TFEB in pcDNA3.1D-V5-HIS	Fwd: caccATGCCCCCACCAGCC<br>Rev: CAGCACATCGCCCTCCTC	IDT
Δ310-476 TFEB in pcDNA3.1D-V5-HIS	Fwd: caccATGGCGTCACGCATAGGGT<br>Rev: GTTGGTCATCTCCAGGCGG	IDT
Δ290-476 TFEB in pcDNA3.1D-V5-HIS	Fwd: caccATGGCGTCACGCATAGGGT<br>Rev: CTGCATCCTCCGGATGTAATCC	IDT
Δ274-476 TFEB in pcDNA3.1D-V5-HIS	Fwd: caccATGGCGTCACGCATAGGGT<br>Rev: GTTCCAGCGCACGTCCA	IDT
Δ256-476 TFEB in pcDNA3.1D-V5-HIS	Fwd: caccATGGCGTCACGCATAGGGT<br>Rev: GATGCGGTCATTGATGTTGAAC	IDT
WT DCAF7 in pcDNA3.1D-V5-HIS	Fwd: cacc ATGTCCCTGCACGGCAAAC<br>Rev: CACTCTGAGTATCTCCAGGCAGTTGTT	IDT
DCAF7 C-terminal HA-tag in pcDNA3.1D-V5-HIS	Fwd: cacc ATGTCCCTGCACGGCAAAC<br>Rev: TTA agcgtaatctggaacatcgtatgggta CACTCTGAGTATCTCCAGGCAGTTGTT	IDT
qPCR CTSA	Fwd: CAGGCTTTGGTCTTCTCTCCA<br>Rev: TCACGCATTCCAGGTCTTTG	IDT
qPCR CTSD	Fwd: AACTGCTGGACATCGCTTGCT<br>Rev: CATTCTTCACGTAGGTGCTGGA	IDT
qPCR HEXA	Fwd: CAACCAACACATTCTTCTCCA<br>Rev: CGCTATCGTGACCTGCTTTT	IDT
qPCR MCOLN1	Fwd: TTGCTCTCTGCCAGCGGTACTA<br>Rev: GCAGTCAGTAACCACCATCGGA	IDT
qPCR SQSTM1	Fwd: AAGCTGCCTTGTACCCAC<br>Rev: CGCTCCGATGTCATAGTTCTTG	IDT
qPCR WIPI1	Fwd: CTTCAAGCTGGAACAGGTCACC<br>Rev: CGGAGAAGTTCAAGCGTGCAGT	IDT
qPCR GAPDH	Fwd: TGCACCACCAACTGCTTAGC<br>Rev: GGCATGGACTGTGGTCATGAG	IDT
qPCR Actin	Fwd: CACCATTGGCAATGAGCGGTTC<br>Rev: AGGTCTTTGCGGATGTCCACGT	IDT
qPCR TFEB	Fwd: CAATACCCCCGTCCACTTCC<br>Rev: CCAGGTAGGACTGCACCTTC	IDT
qPCR 229E	Fwd: CAC TCG TTA TGT GGG TGA TG<br>Rev: CAC GGT TGT GAC AGT GAT T<br>Probe: 56-FAM/TT GGG CAT GGA ATC CTG AGG TCA A/36-TAMSp	IDT
qPCR OC43	Fwd: ATG TTA GGC CGA TAA TTG AGG ACT AT<br>Rev: AAT GTA AAG ATG GCC GCG TAT T<br>Probe: 56-FAM/CA TAC TCT GAC GGT CAC AAT /36-TAMSp/	IDT

Primers used for SARS-CoV-2 detection	IITRI<br>2019-nCoV_N1-F 5’-GACCCCAAAATCAGCGAAAT-3’<br>2019-nCoV_N1-R 5’-TCTGGTTACTGCCAGTTGAATCTG-3’<br>Probe:<br>2019-nCoV_N1-P<br>5’-FAM-ACCCCGCATTACGTTTGGTGGACC-BHQ1-3’.	N/A
NSP1_Flexi_fwd	aggagcgatcgcc ATGGAGAGTCTTGTACCCGGCTTTAACGAG	This Study
NSP1_Flexi_rev	cgcggtttaaac GCCGCCGTTCAGTTCGCGCA	This Study
NSP2_Flexi_fwd	cttagcgatcgcc ATGGCCTACACGCGATATGTTG	This Study
NSP2_Flexi_rev	aacagtttaaac GCCGCCCTTAAGGGTAAAGGT	This Study
NSP4_Flexi_fwd	gcgtgcgatcgcc ATGAAGATTGTAAACAATTGGCT	This Study
NSP4_Flexi_rev	aactgtttaaac CTGGAGTACGGCTGAGGTTAT	This Study
NSP5/3CLPro_Flexi_fwd	gcgtgcgatcgcc ATGTCTGGTTTTAGGAAAATGGCGT	This Study
NSP5/3CLPro_Flexi_Rev	cttggtttaaac CTGGAAAGTGACCCCACTGC	This Study
NSP6_FLEXI_fwd	Agctgcgatcgcc ATGAGCGCCGTGAAGAGAACCAT	This Study
NSP6_Flexi_rev	caaagtttaaac CTGCACGGTGGCCACCTTGA	This Study
NSP7_Flexi_fwd	ctaagcgatcgcc ATGAGCAAGATGAGCGACGTAAAAT	This Study
NSP7_Flexi_rev	ttgtgtttaaac CTGCAATGTCGCCCGGTTGT	This Study
NSP8_Flexi_fwd	gaccgcgatcgcc ATGGCAATCGCATCTGAATTTTCT	This Study
NSP8_Flexi_rev	gcaggtttaaac CTGCAGTTTGACTGCGCTGTTAG	This Study
NSP9_Flexi_fwd	gaccgcgatcgcc ATGAATAATGAGCTTTCTCCGGTTGCTCT	This Study
NSP9_Flexi_rev	tggtgtttaaac TTGGAGTCGCACGGTCGCAG	This Study
NSP10_Flexi_fwd	atatgcgatcgcc ATGGCTGGTAATGCAACTGAGGTTC	This Study
NSP10_Flexi_rev	gtcggtttaaac CTGCAACATCGGCTCTCGCAA	This Study
NSP12/RDRP_Flexi_fwd	cgaagcgatcgcc ATGTCAGCAGACGCACAAAG	This Study
NSP12/RDRP_Flexi_rev	gtcggtttaaac CTGCAGGACGGTGTGAGGCG	This Study
NSP13/Helicase_Flexi_fwd	taaagcgatcgcc ATGGCTGTTGGGGCGTGTGT	This Study
NSP13/Helicase_Flexi_rev	ggttgtttaaac TTGAAGCGTCGCAACGTTCCTG	This Study
NSP14/3-5Exo_Flexi_fwd	gaccgcgatcgcc ATGGCTGAAAATGTAACGGGCTT	This Study
NSP14/3-5Exo_Flexi_rev	ttgtgtttaaac CTGCAATCGGGTAAATGTGT	This Study
NSP15/endo_flexi_fwd	taaagcgatcgcc ATGAGTCTTGAAAACGTCGCC	This Study
NSP15/endo_flexi_rev	gatggtttaaac TTGCAACTTTGGATAGAAGGTCTC	This Study
NSP16_Flexi_fwd	Tcaggcgatcgcc ATGTCTTCCCAGGCTTGGCAG	This Study
NSP16_Flexi_rev	Acgtgtttaaac GTTGTTCACCAGCACGTCAGAGG	This Study
Spike protein-hibit_flexi_F	GCGTGCGATCGCCATGTTTGTGTTCCTGGTGCT	This Study
Spike protein-hibit_flexi_R	GGTTGTTTAAACGGTGTAGTGCAGTTTCACTCCT	This Study
orf3a_Flexi_fwd	taaagcgatcgcc ATGGACCTTTTTATGAGAATCTTTACCATCGGG	This Study
orf3a_Flexi_rev	ggttgtttaaac CAGGGGCACAGATGTGGTCG	This Study
Orf3b_flexi_fwd	gcaggcgatcgcc ATGGCATACTGCTGGAGATGCACAAGCTGTTGTTTTAG	This Study
Orf3b_Flexi_rev	tgttgtttaaacCGGCCAGCAGCATCGAGCGAA	This Study
ORF4/Env_flexi_fwd	AGCTGCGATCGCCATGTACAGCTTCGTATCAGAAGAAACCGGGACACT	This Study
ORF4/Env_flexi_rev	GGTTGTTTAAACAACGAGGAGATCCGGCACCCTG	This Study
Mprotein_Flexi_fwd	GACGGCGATCGCCATGGCCGACTCAAATGGGAC	This Study
Mprotein_Flexi_rev	TGTTGTTTAAACCTGGACGAGCAAAGCAATGT	This Study
orf6_Flexi_fwd	atatgcgatcgcc ATGTTTCATCTTGTCGATTTTC	This Study
orf6_Flexi_rev	ggcggtttaaac GTCTATTTCCATAGGCTGCT	This Study
orf7a_Flexi_fwd	gaccgcgatcgcc ATGAAGATCATTTTGTTTCTGGCCC	This Study
orf7a_Flexi_rev	gacggtttaaac CTCAGTCTTTCTTTTCAGTGTAAAGC	This Study
orf7b_Flexi_fwd	cccggcgatcgcc ATGATTGAGCTGTCTCTCATCGATTTCTATCTCTG	This Study
orf7b_Flexi_rev	gatggtttaaac GGCGTGGCATGTCTCGTTATGG	This Study
orf8_Flexi_fwd	cgcggcgatcgcc ATGAAGTTTCTCGTGTTCTTGGGCA	This Study
orf8_Flexi_rev	gggggtttaaac TATAAAGTCAAGGACCACCCTGACA	This Study
Nprotein_Flexi_fwd	cgaggcgatcgcc ATGAGCGATAACGGCCCCCA	This Study
Nprotein_Flexi_rev	acgtgtttaaac CGCCTGAGTAGAATCGGCTGAG	This Study
Orf9c_Flexi_fwd	acaggcgatcgccATGGCTCTTCAATCCTGCTATAACTTC	This Study
Orf9c_Flexi_rev	gggggtttaaacATCCGTAAGACAGCAGCACA	This Study
orf10_Flexi_fwd	gccggcgatcgcc ATGGGCTACATCAATGTCTTCGCCTTTCCT	This Study
orf10_Flexi_rev	gacggtttaaac TGTGAGATTGAAGTTTACAACATCGACCTGAGC	This Study

Uncropped Blots

Fig 1

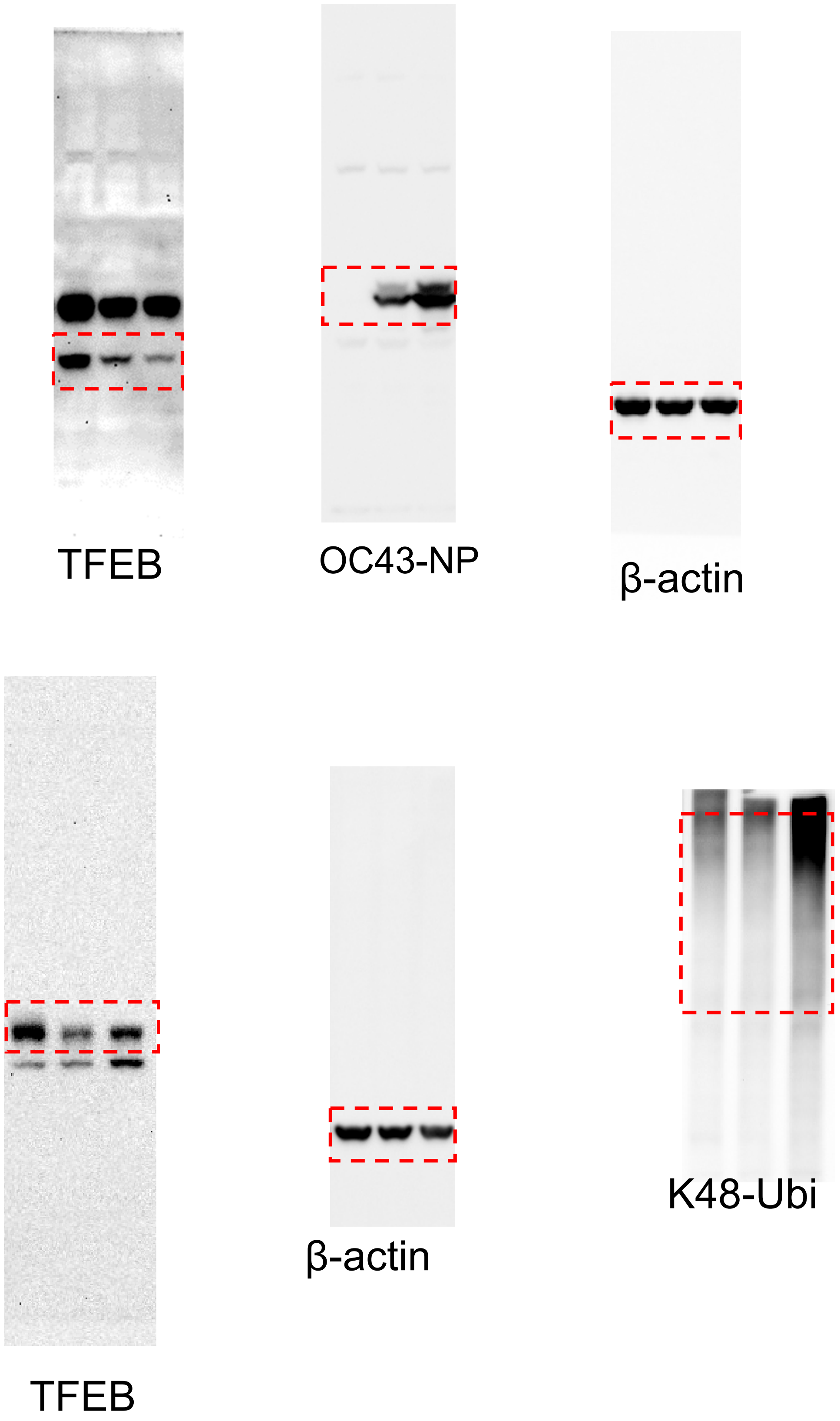

Fig. 1

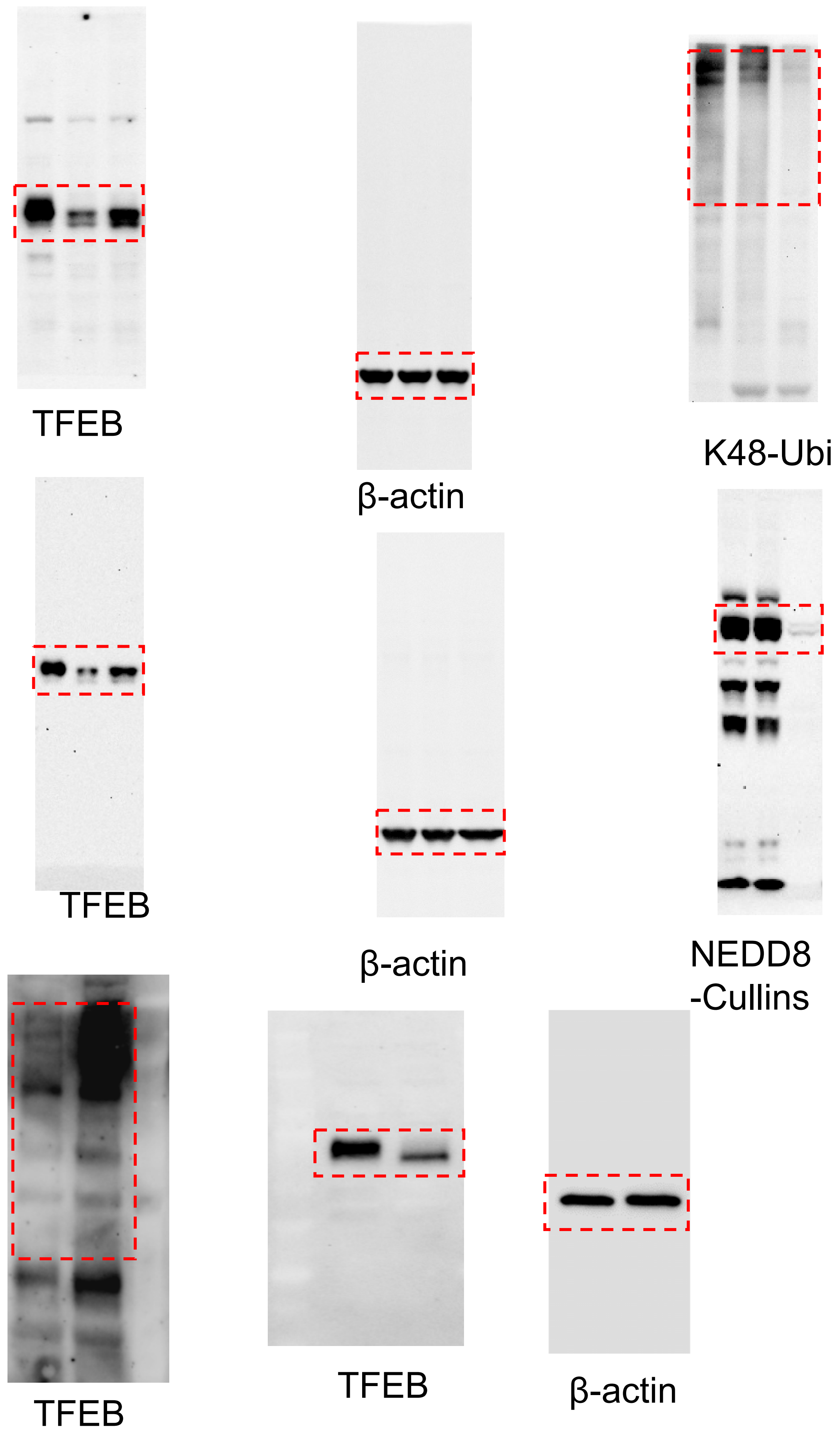
TFEB
β-actin
K48-Ubi
TFEB
β-actin
NEDD8
-Cullins
TFEB
TFEB
β-actin

Fig. 2

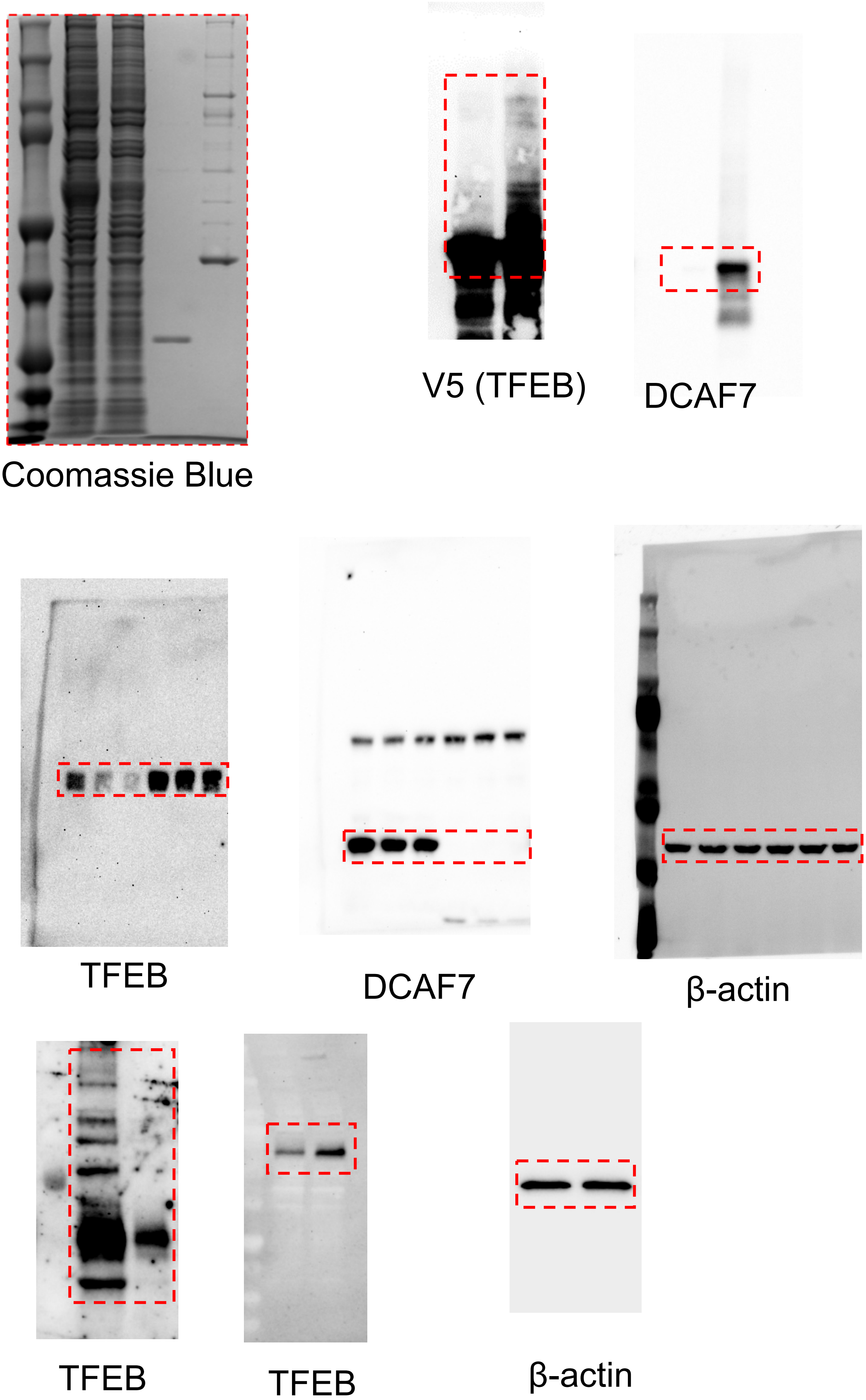
Coomassie Blue
V5 (TFEB)
DCAF7
TFEB
DCAF7
β-actin
TFEB
TFEB
β-actin

Fig. 2

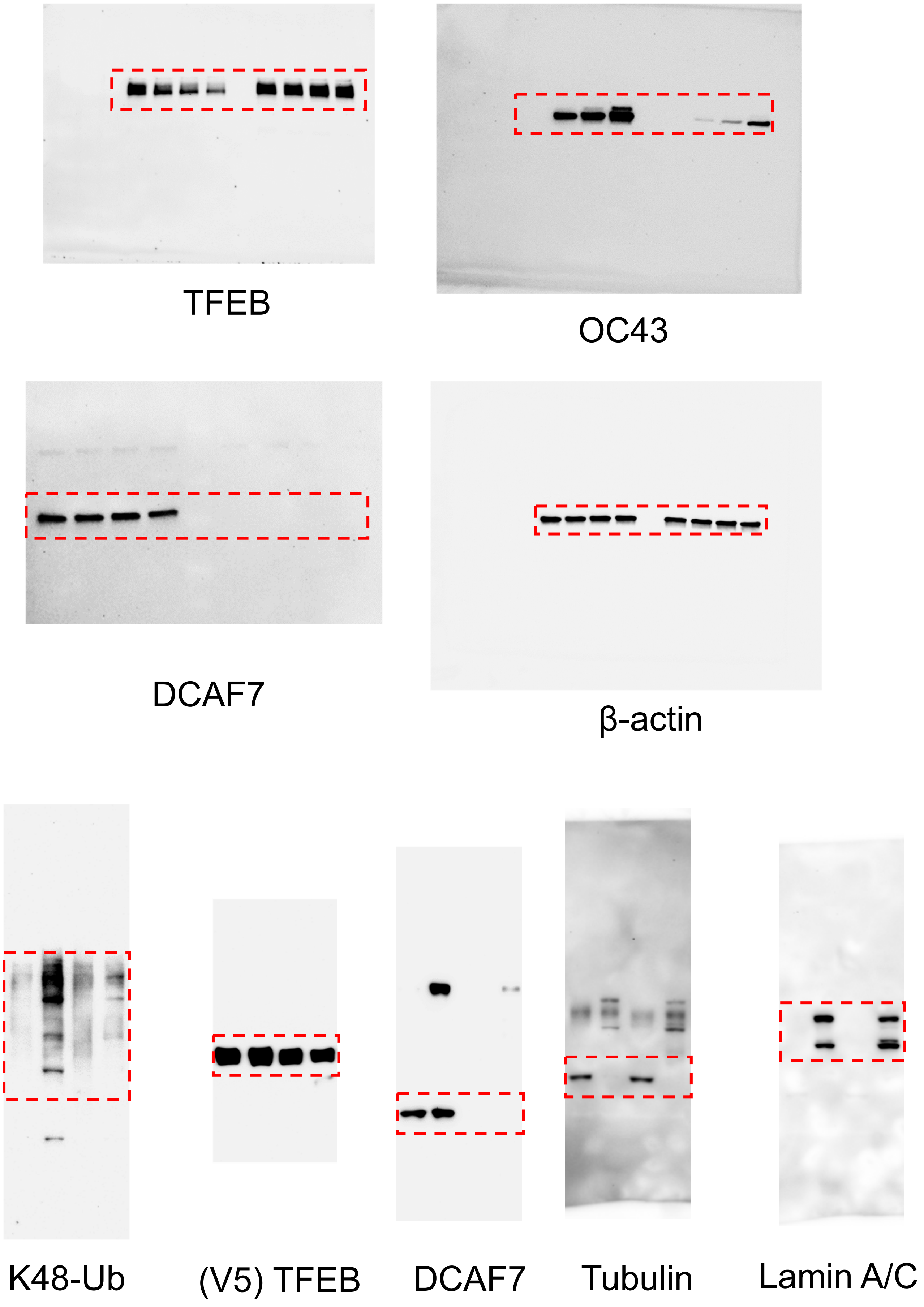

Fig. 3

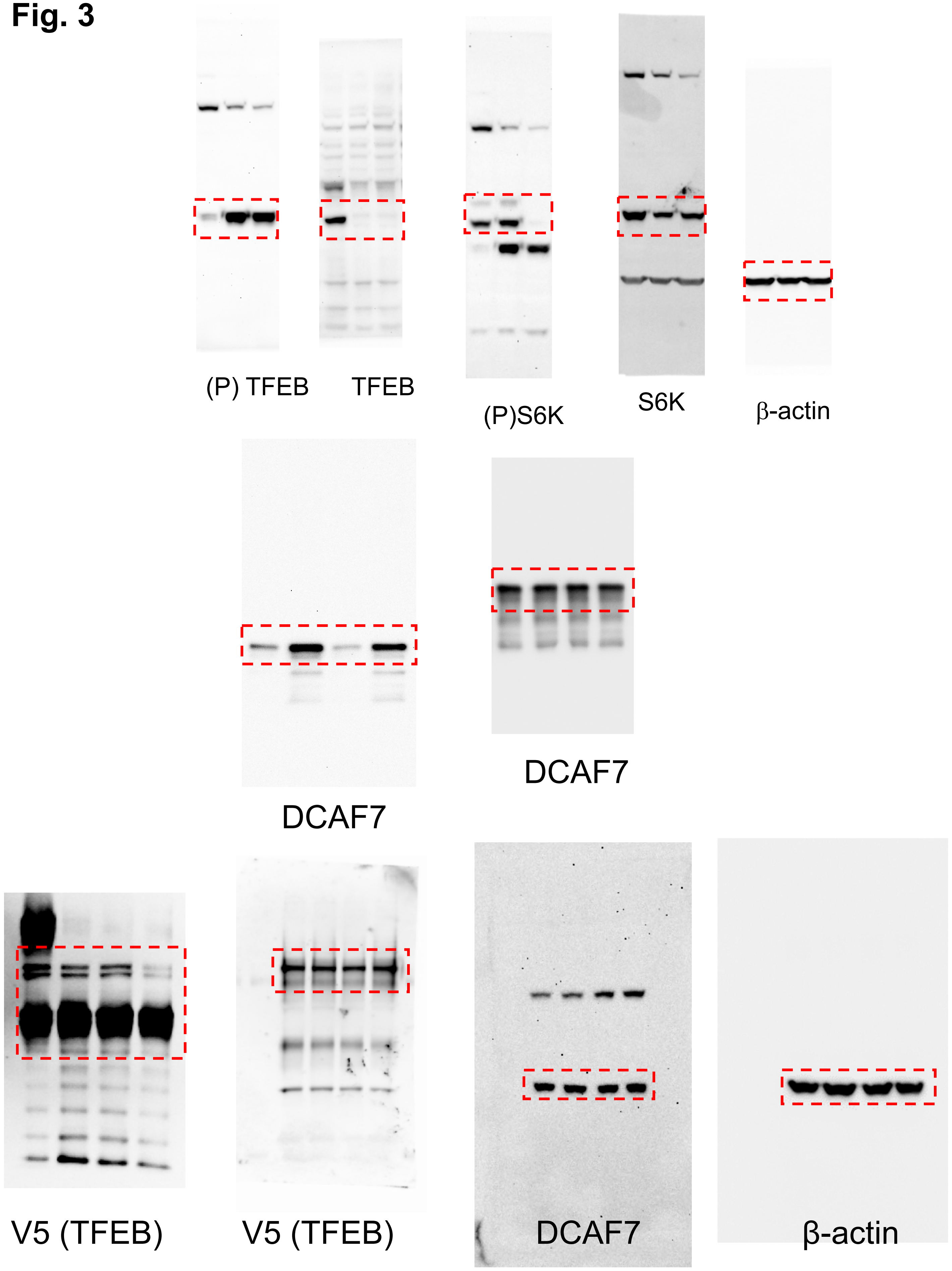
(P) TFEB
TFEB
(P)S6K
S6K
β-actin
DCAF7
DCAF7
V5 (TFEB)
V5 (TFEB)
DCAF7
β-actin

Fig. 3

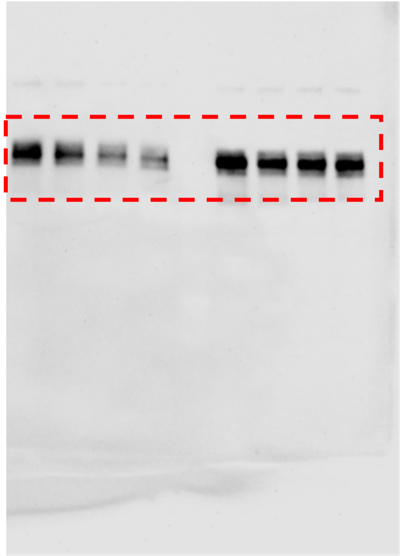

EGFP (TFEB)

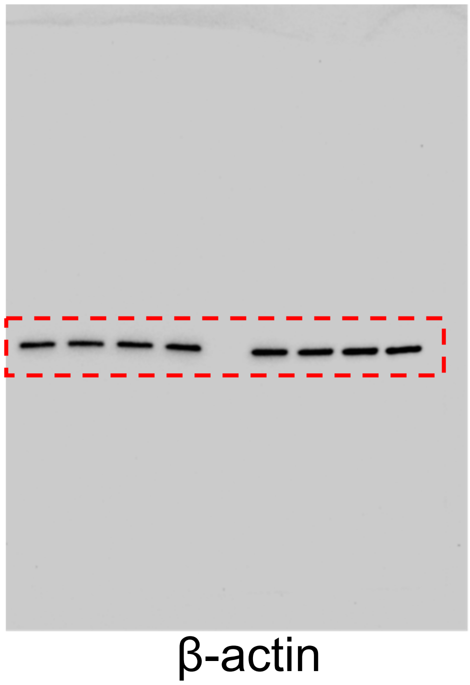

β-actin

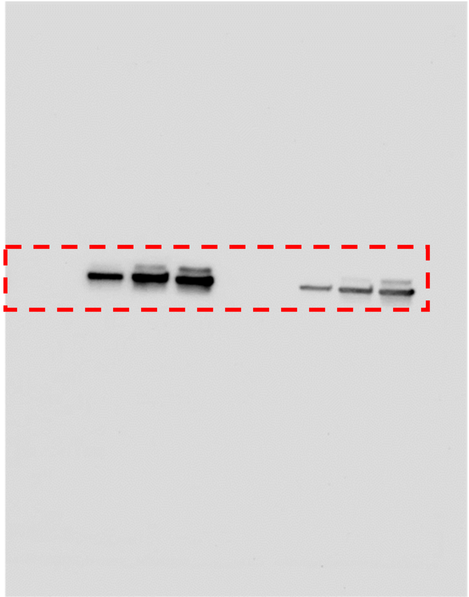

OC43

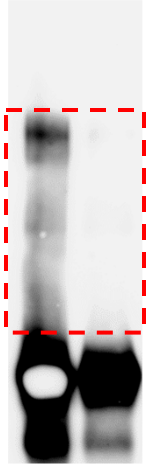

K48-Ub

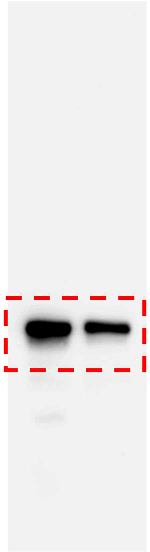

V5 (TFEB)

Fig. 4

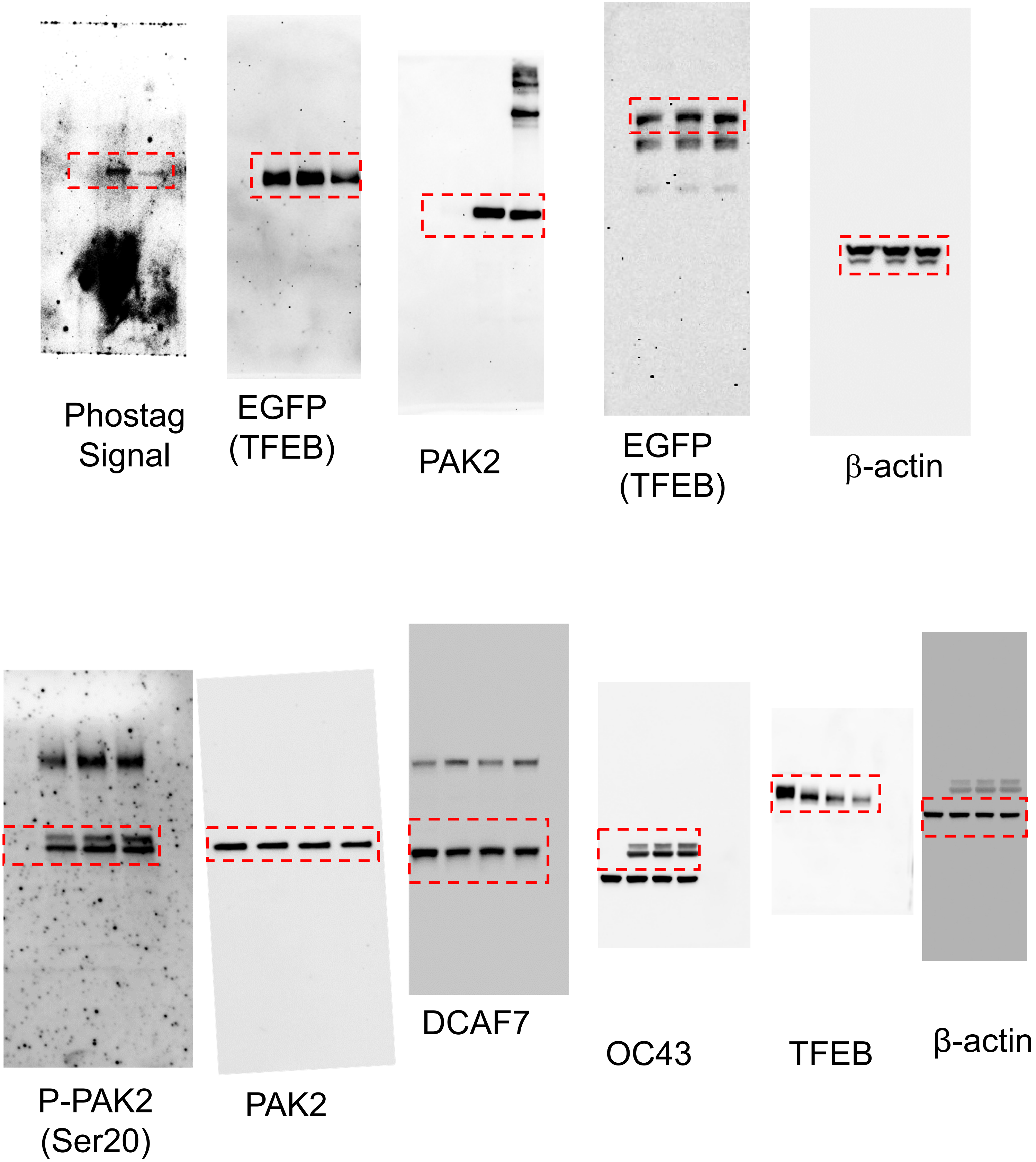
Phostag Signal
EGFP (TFEB)
PAK2
EGFP (TFEB)
β-actin
P-PAK2 (Ser20)
PAK2
DCAF7
OC43
TFEB
β-actin

Fig. 4

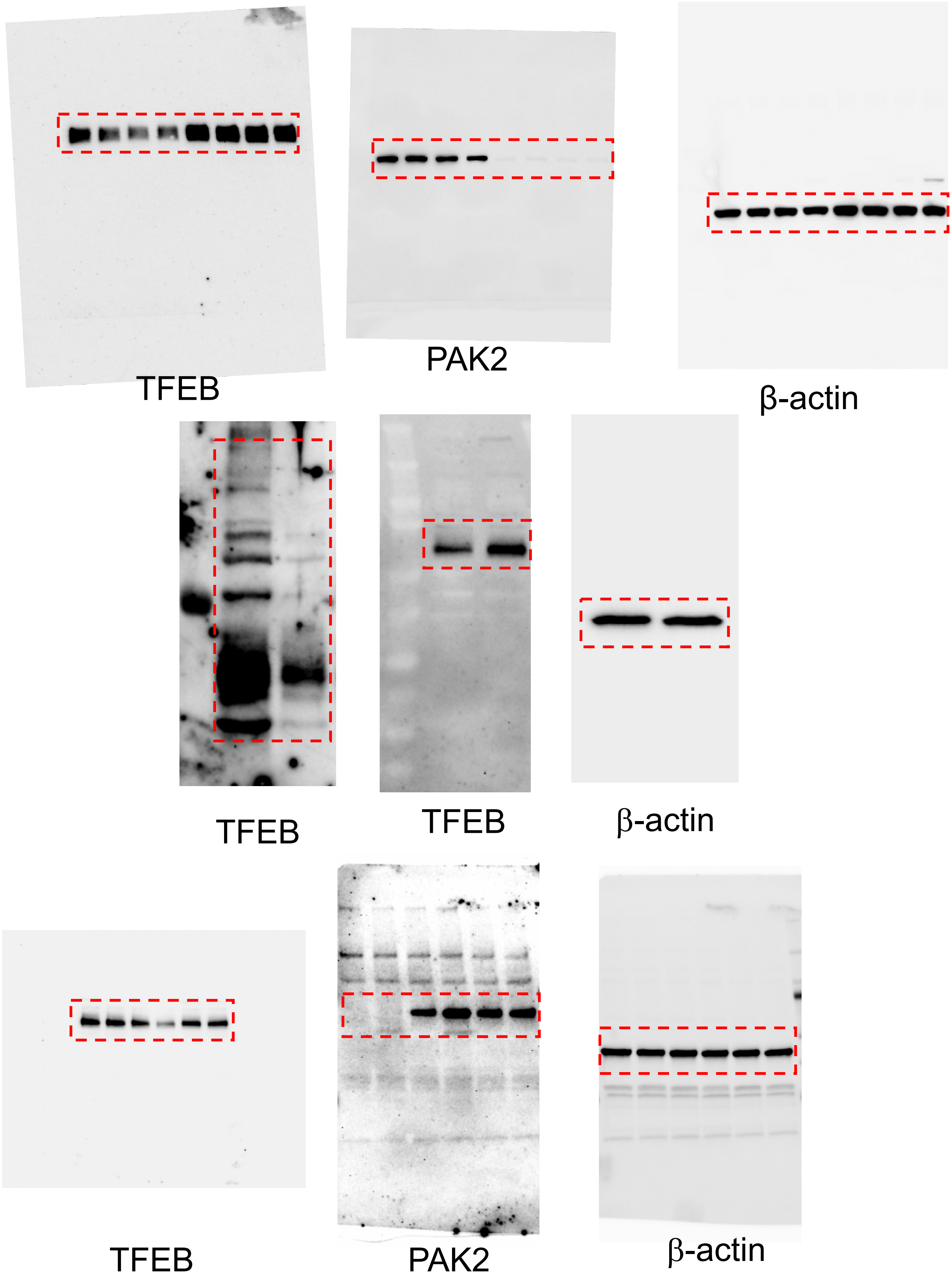

Fig. 5

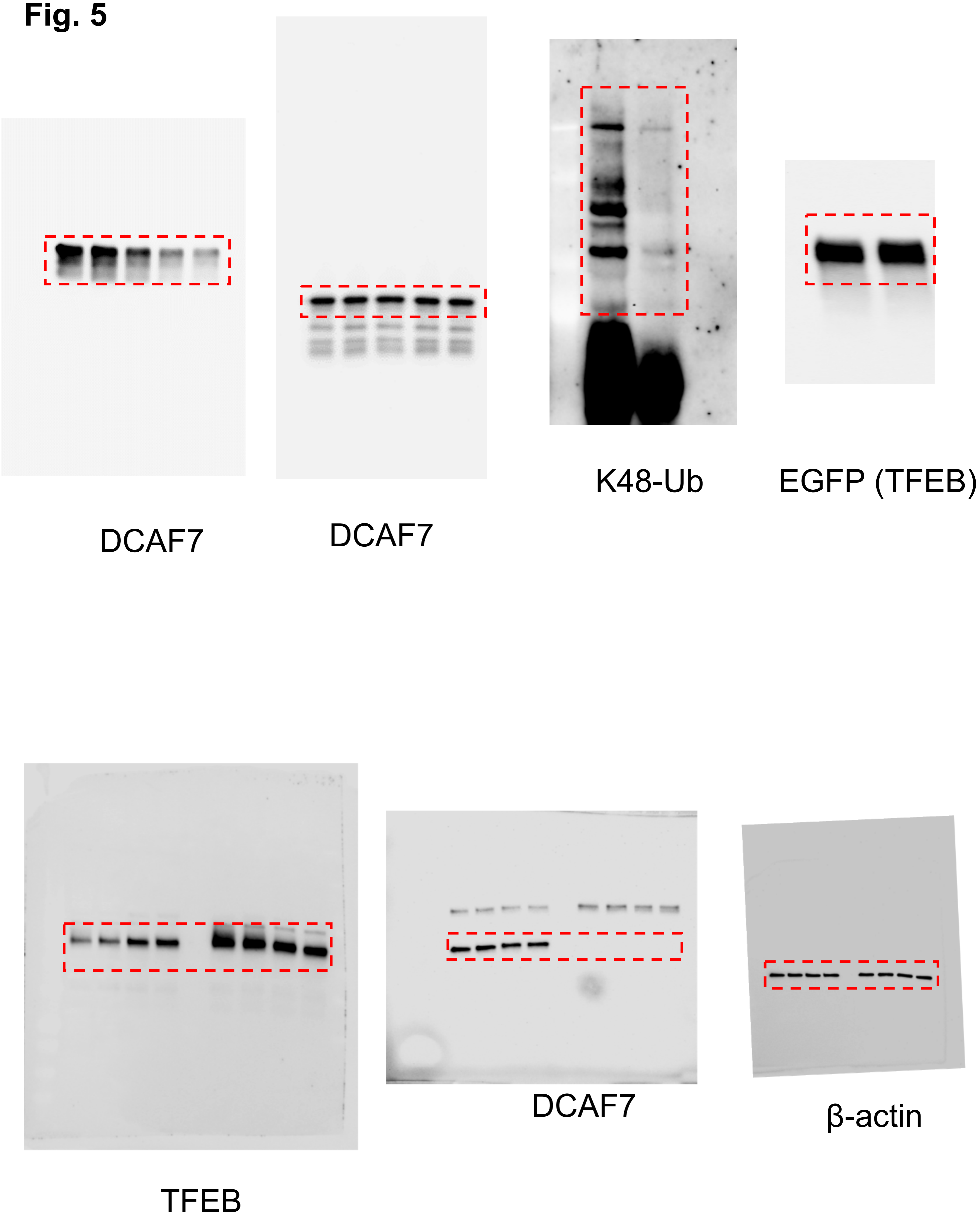

Fig. 7

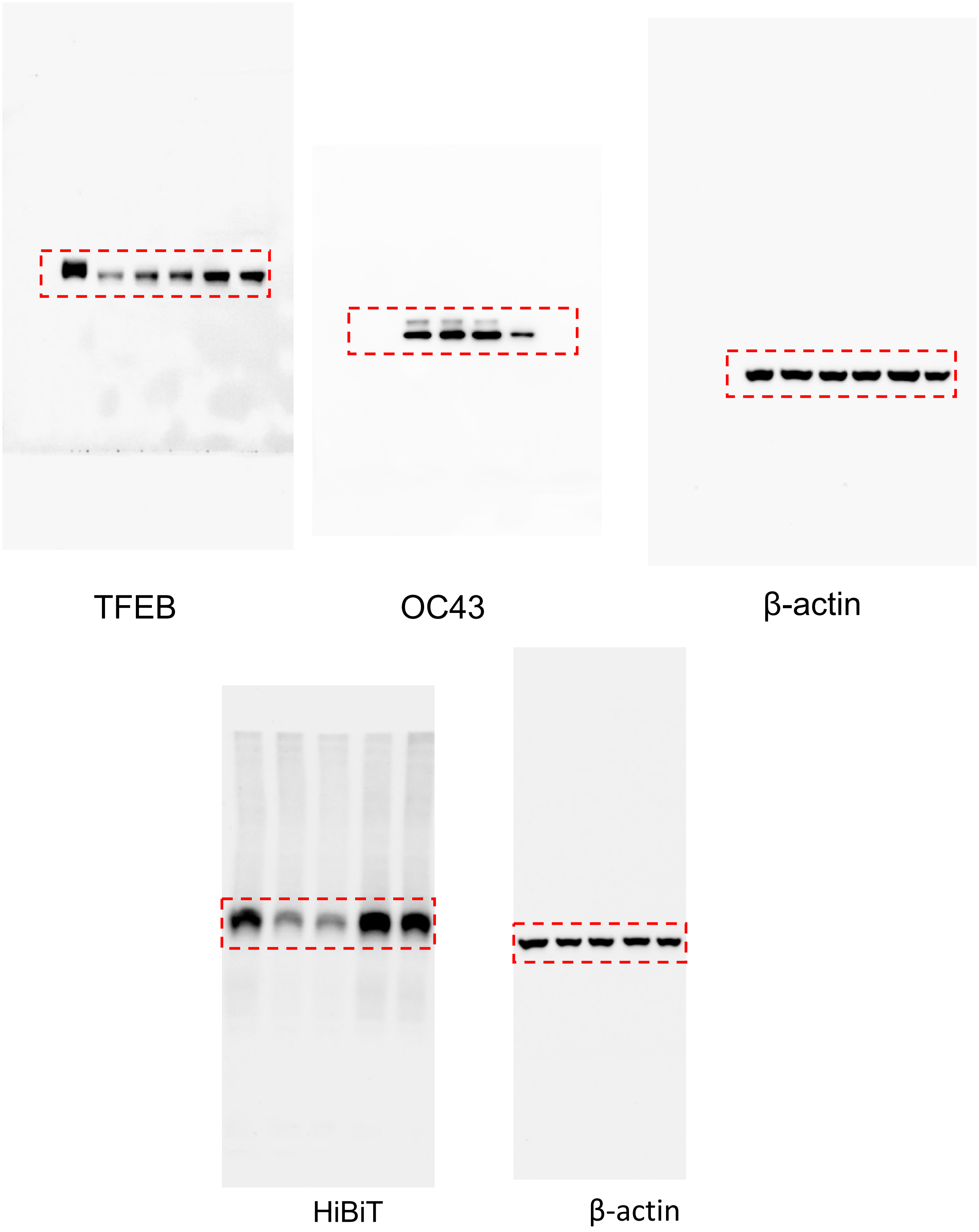

Supplementary

Fig. S1

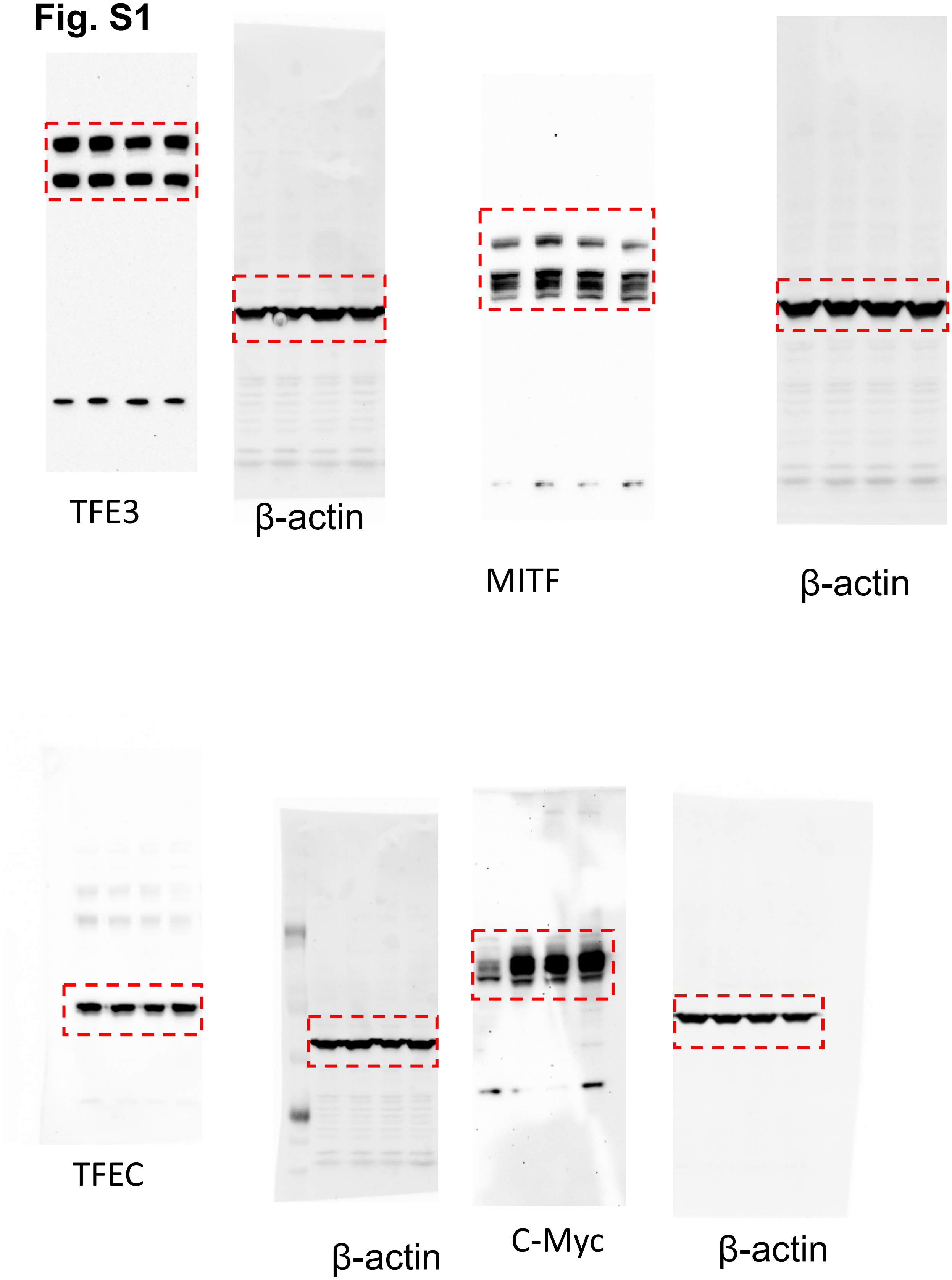

Fig. S1

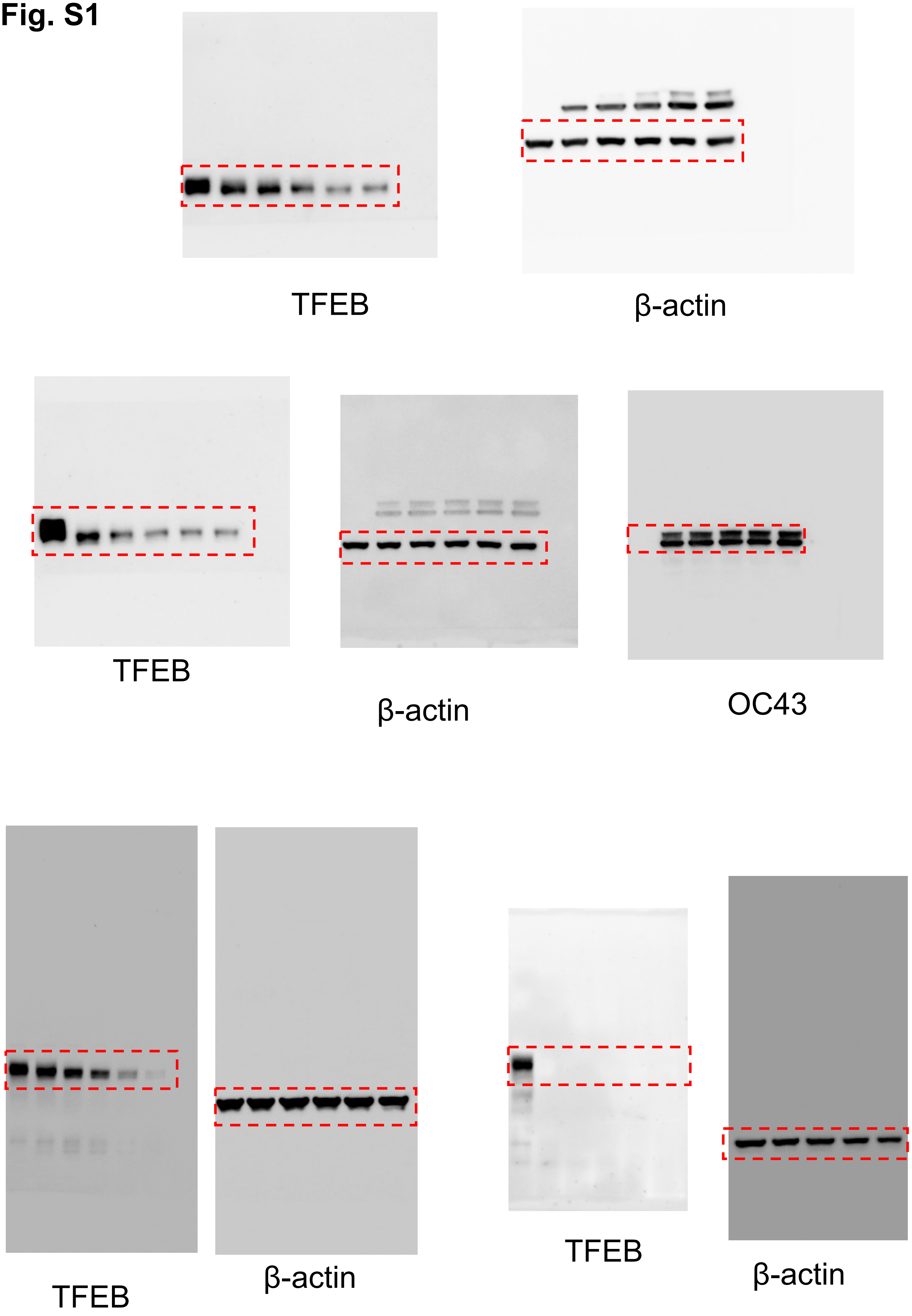

Fig. S2

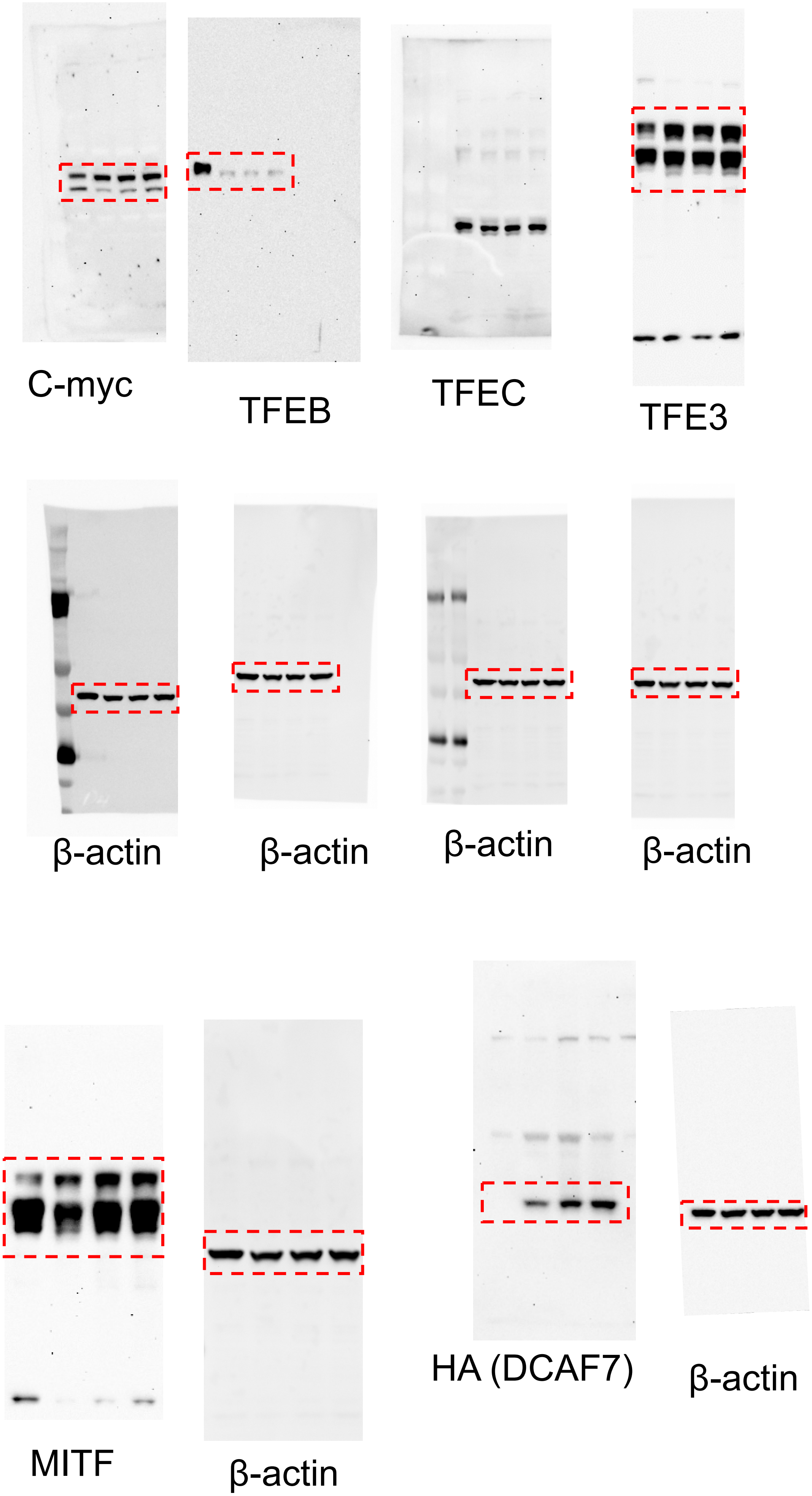
C-myc
TFEB
TFEC
TFE3
β-actin
β-actin
β-actin
β-actin
MITF
β-actin
HA (DCAF7)
β-actin

Fig. S2

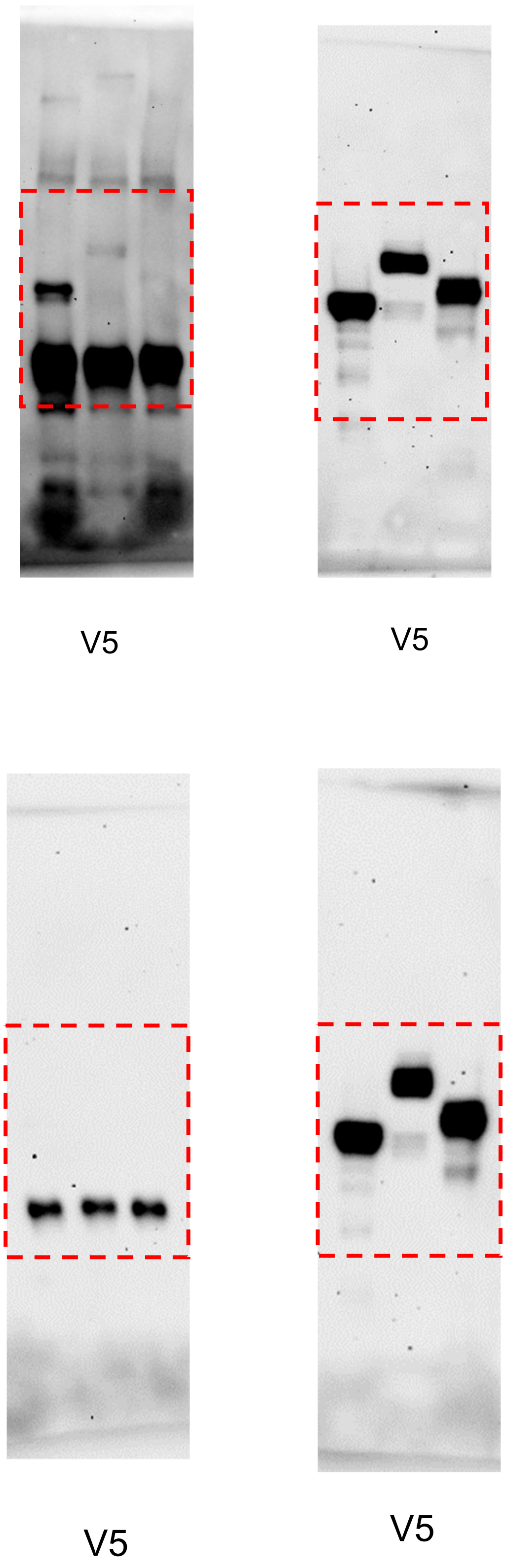

Fig. S3

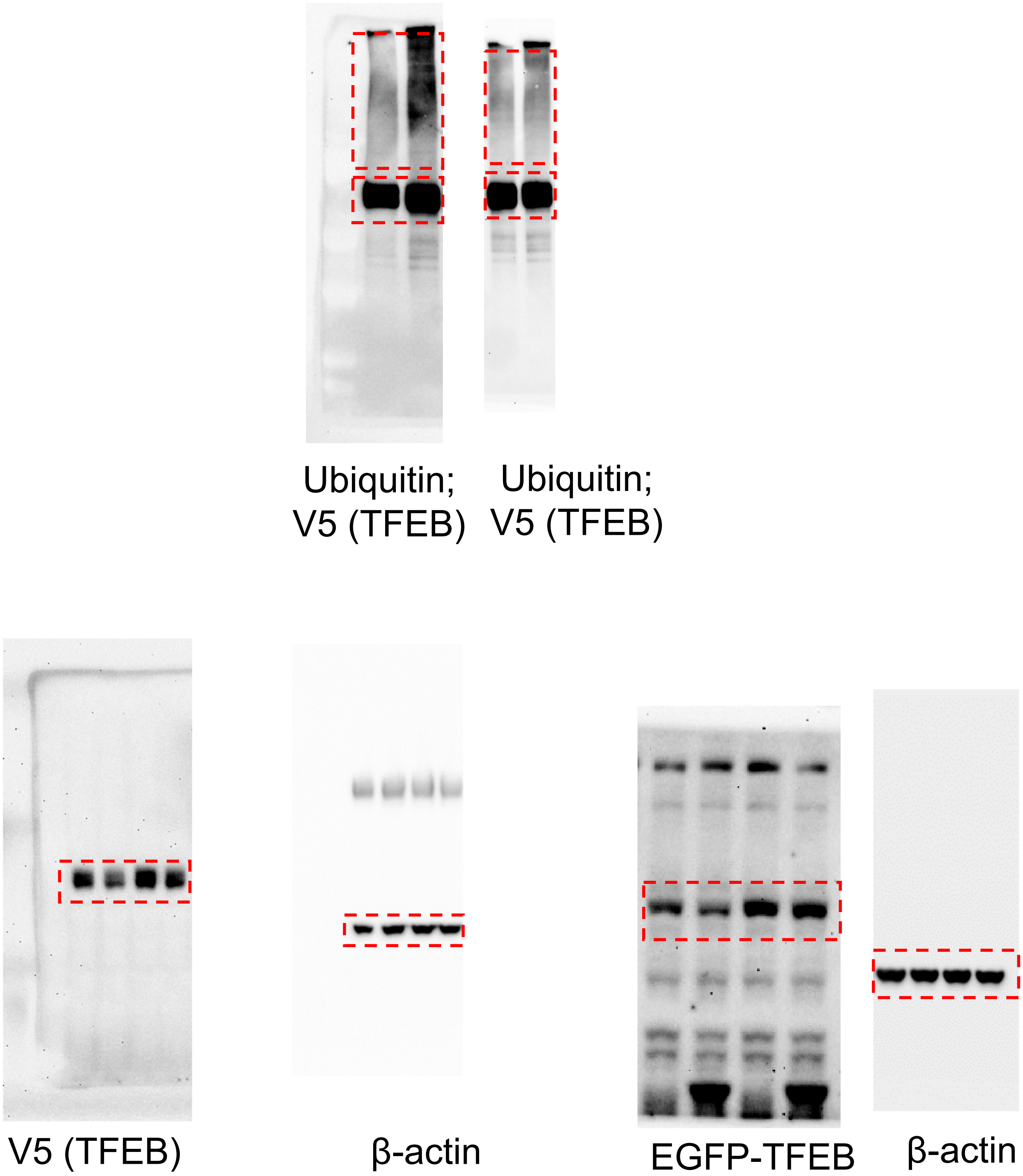
Ubiquitin;
V5 (TFEB)
Ubiquitin;
V5 (TFEB)
V5 (TFEB)
β-actin
EGFP-TFEB
β-actin

Fig. S4

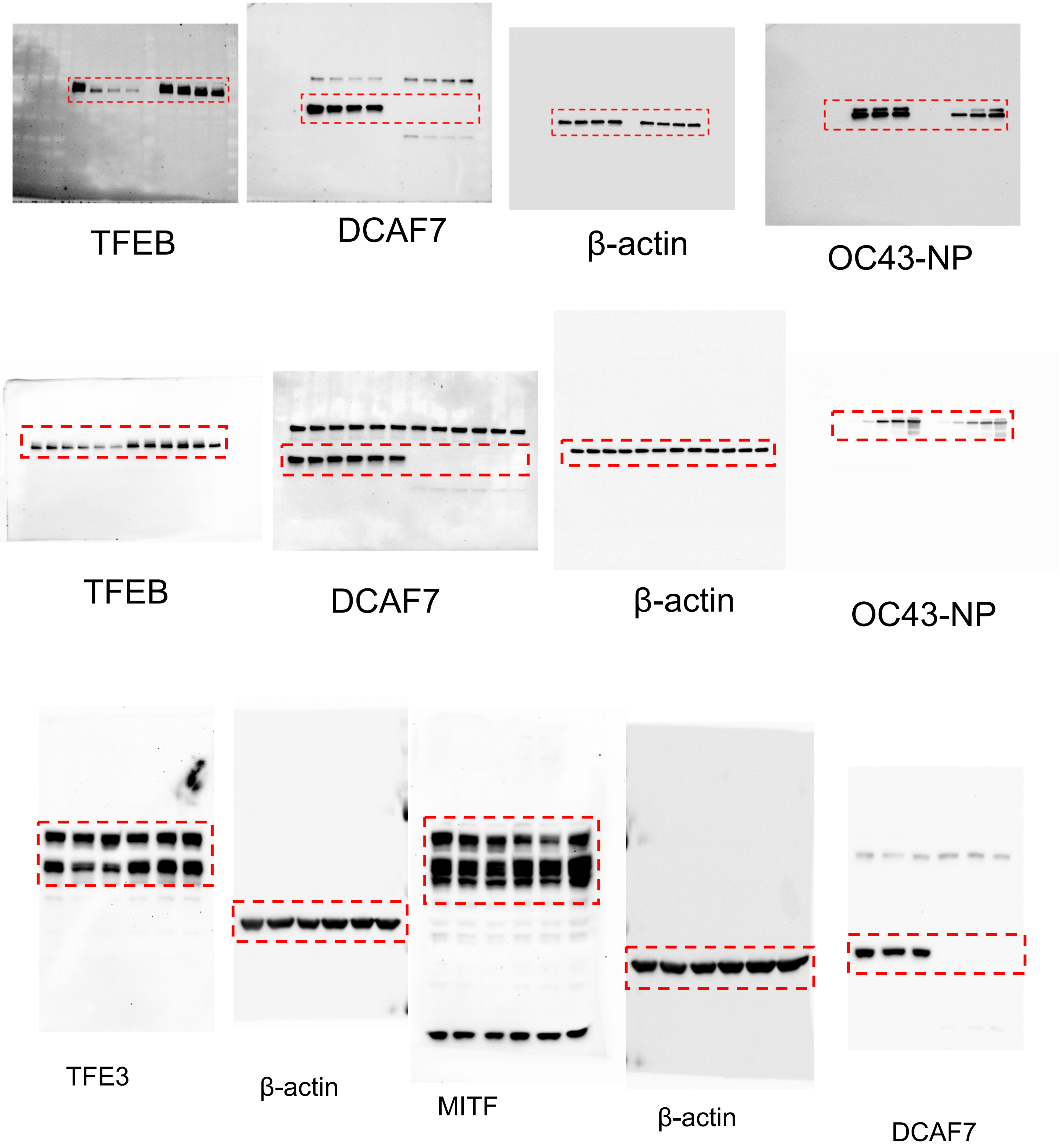

TFEB
DCAF7
β-actin
OC43-NP
TFEB
DCAF7
β-actin
OC43-NP
TFE3
β-actin
MITF
β-actin
DCAF7

Fig. S6

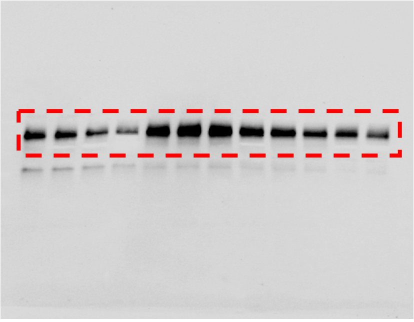

TFEB

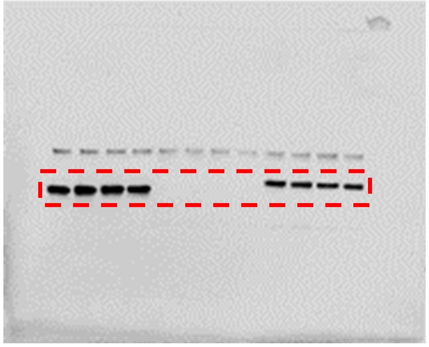

DCAF7

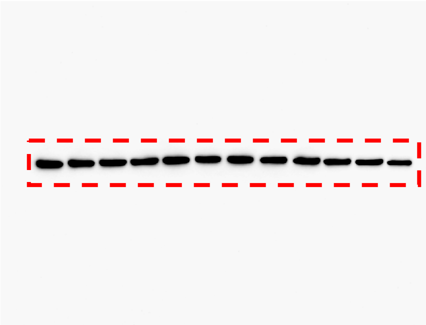

β-actin

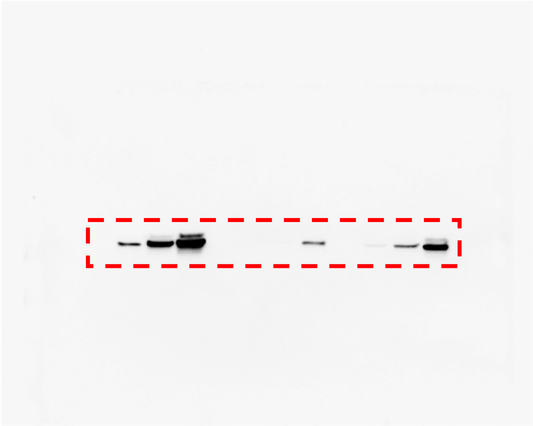

OC43

Fig. S7

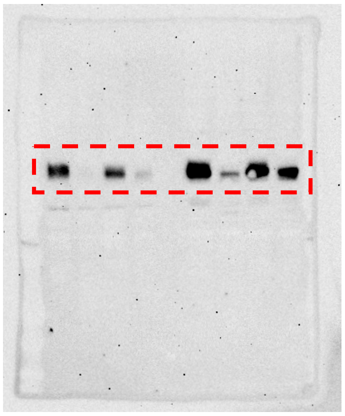

TFEB

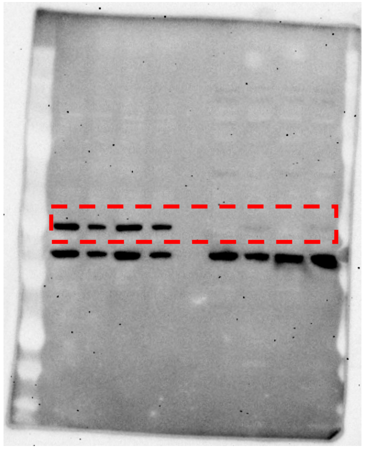

α-Tubulin

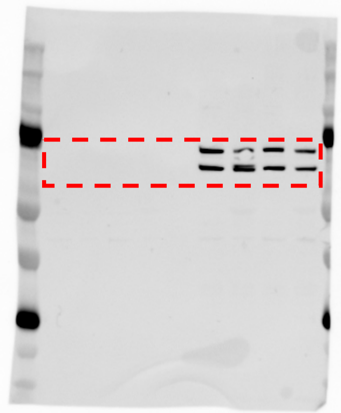

LaminA/C

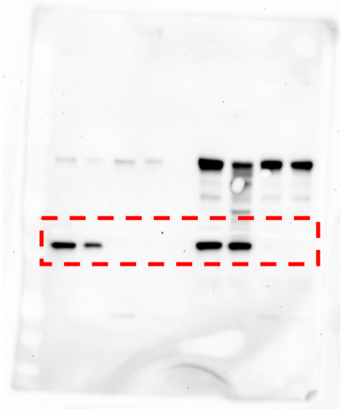

DCAF7

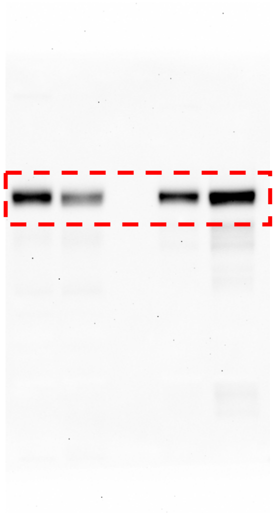

EGFP (TFEB)

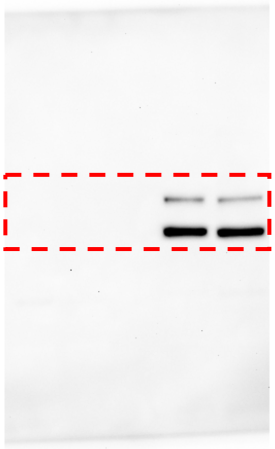

Lamin A/C

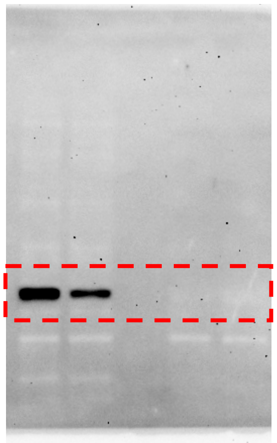

α-Tubulin

Fig. S8

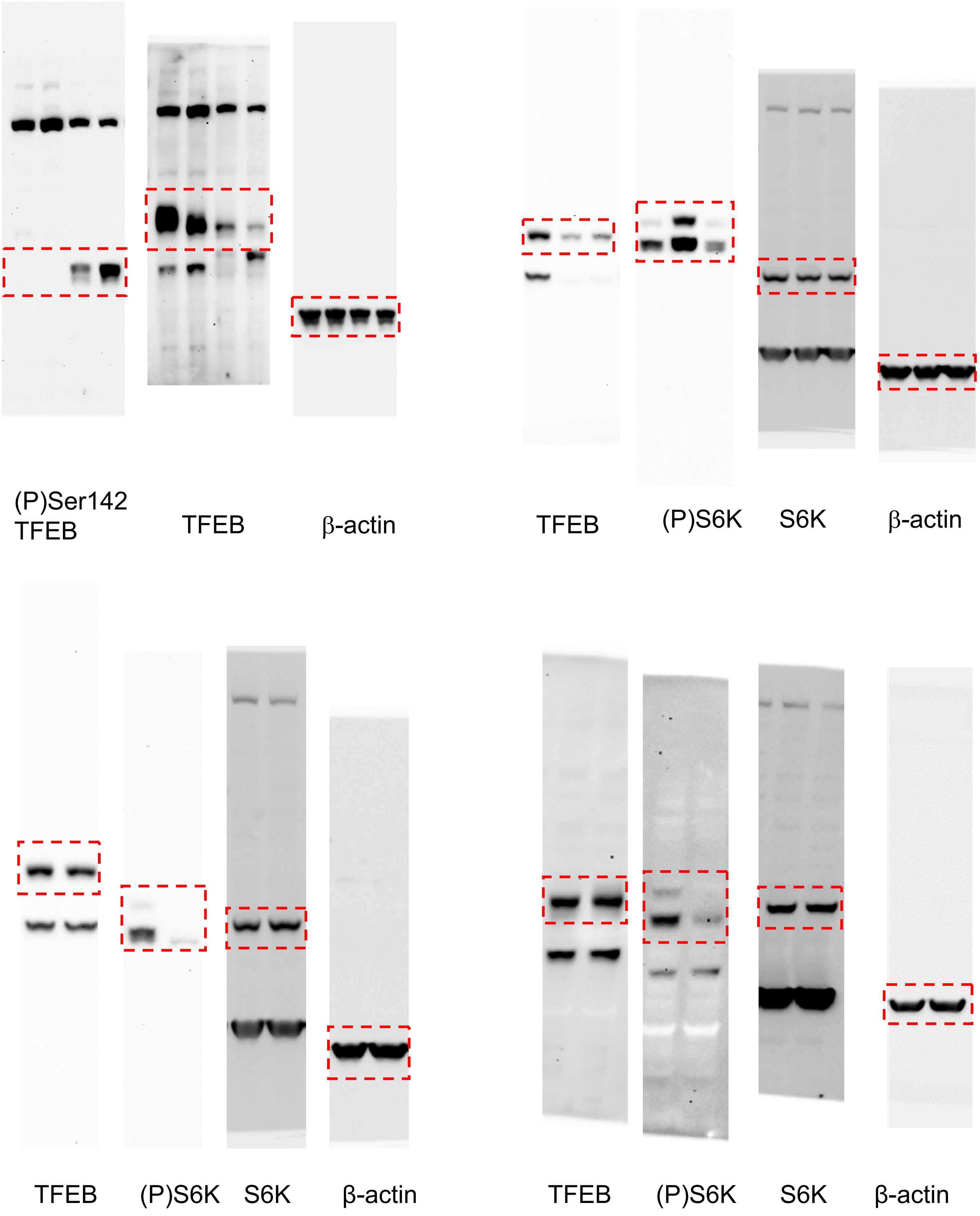

(P)Ser142 TFEB
TFEB
β-actin
TFEB
(P)S6K
S6K
β-actin
TFEB
(P)S6K
S6K
β-actin
TFEB
(P)S6K
S6K
β-actin

Fig. S8

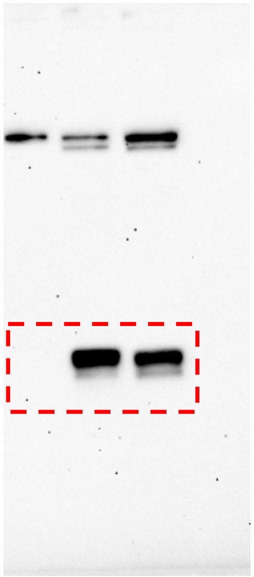

(P) TFEB

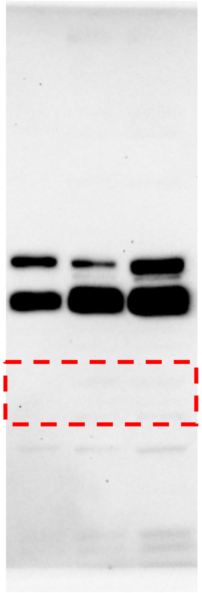

Tubulin

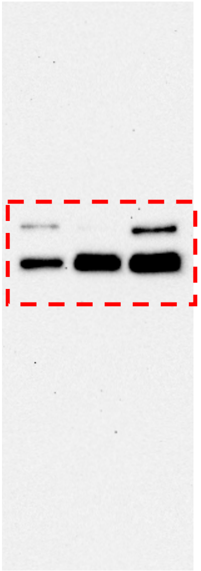

Lamin AC

Fig. S9

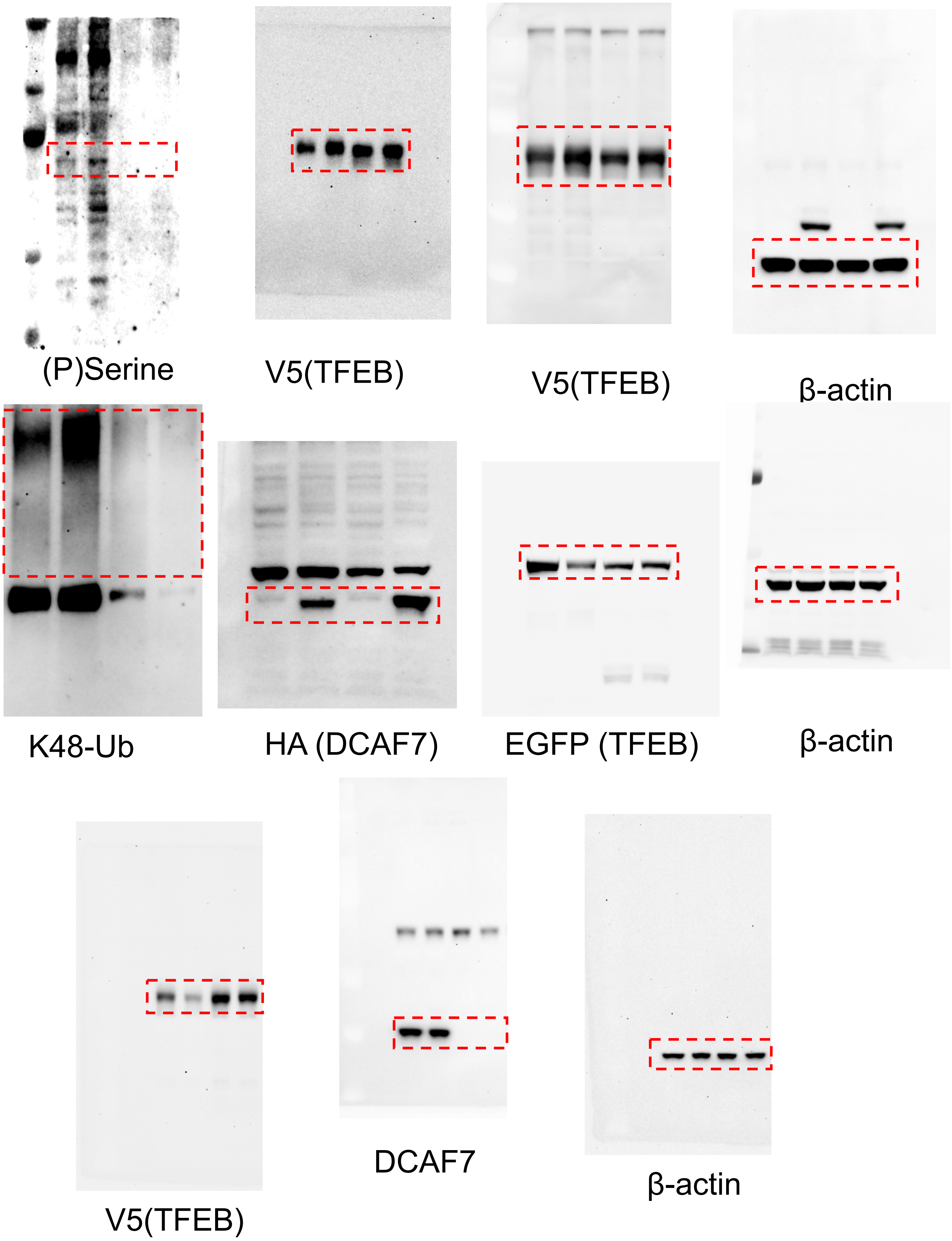
(P)Serine
V5(TFEB)
V5(TFEB)
β-actin
K48-Ub
HA (DCAF7)
EGFP (TFEB)
β-actin
V5(TFEB)
DCAF7
β-actin

Fig. S10

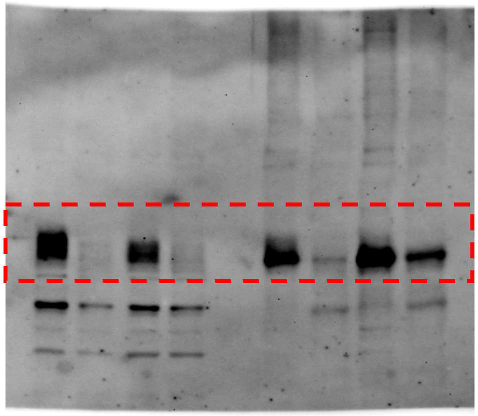

TFEB

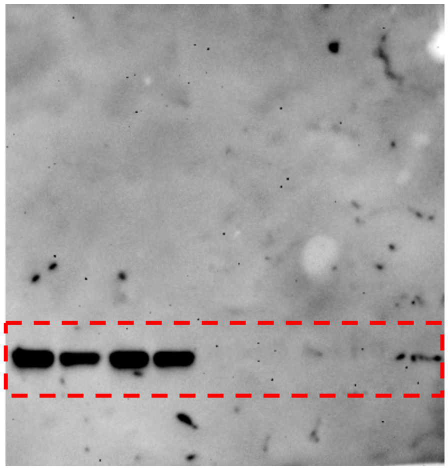

α-Tubulin

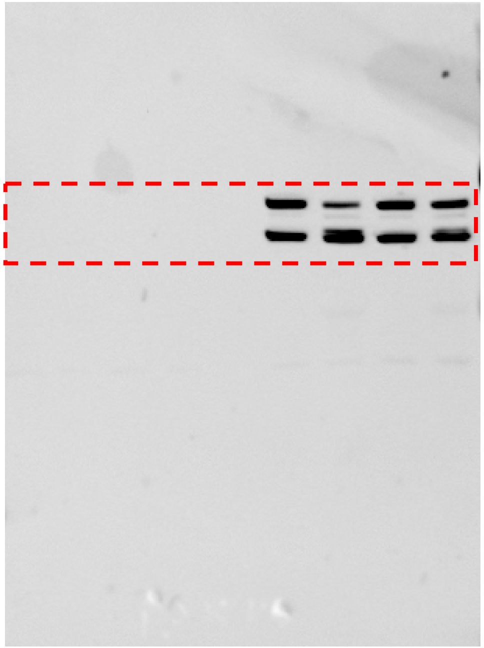

Lamin A/C

Fig. S10

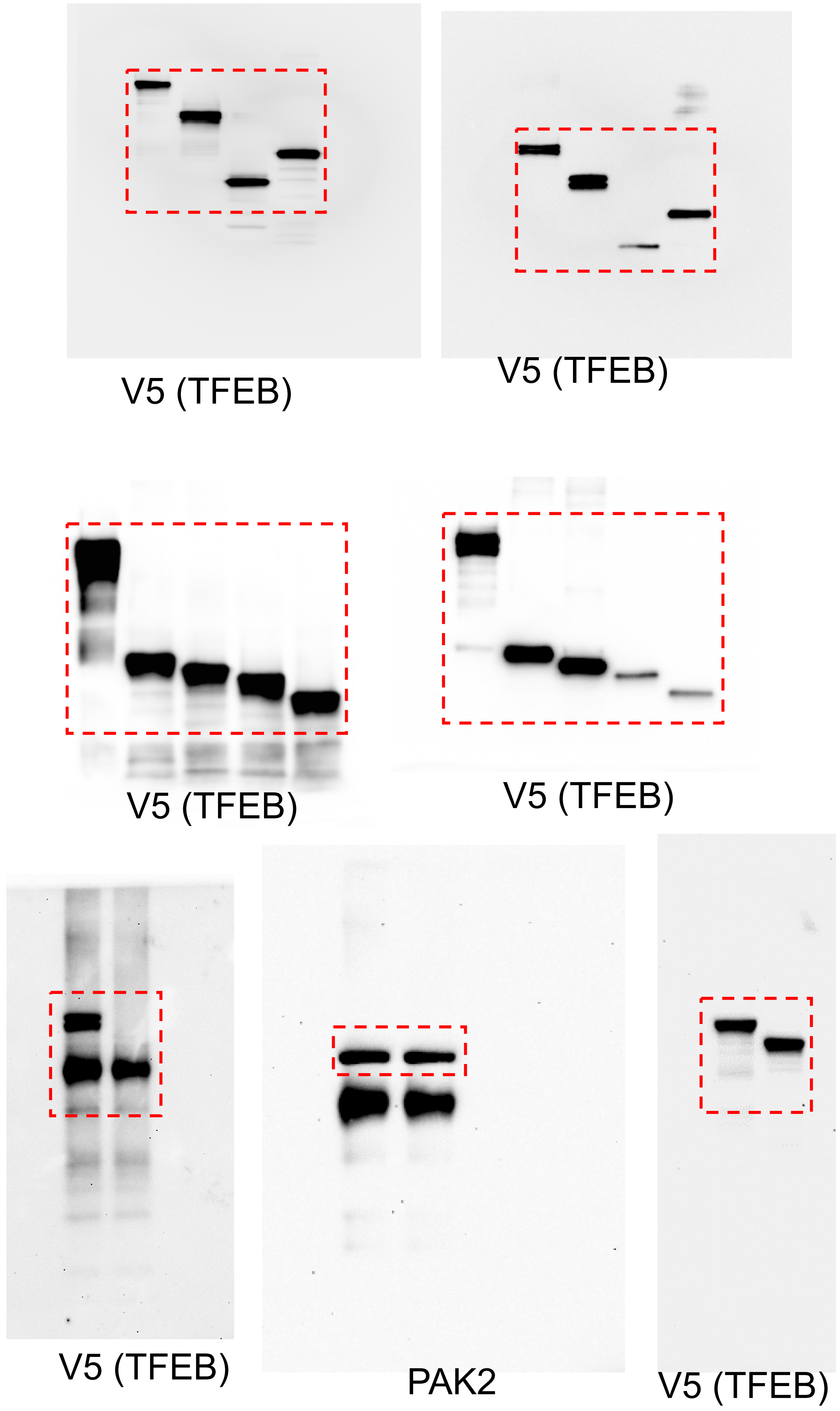

Fig. S11

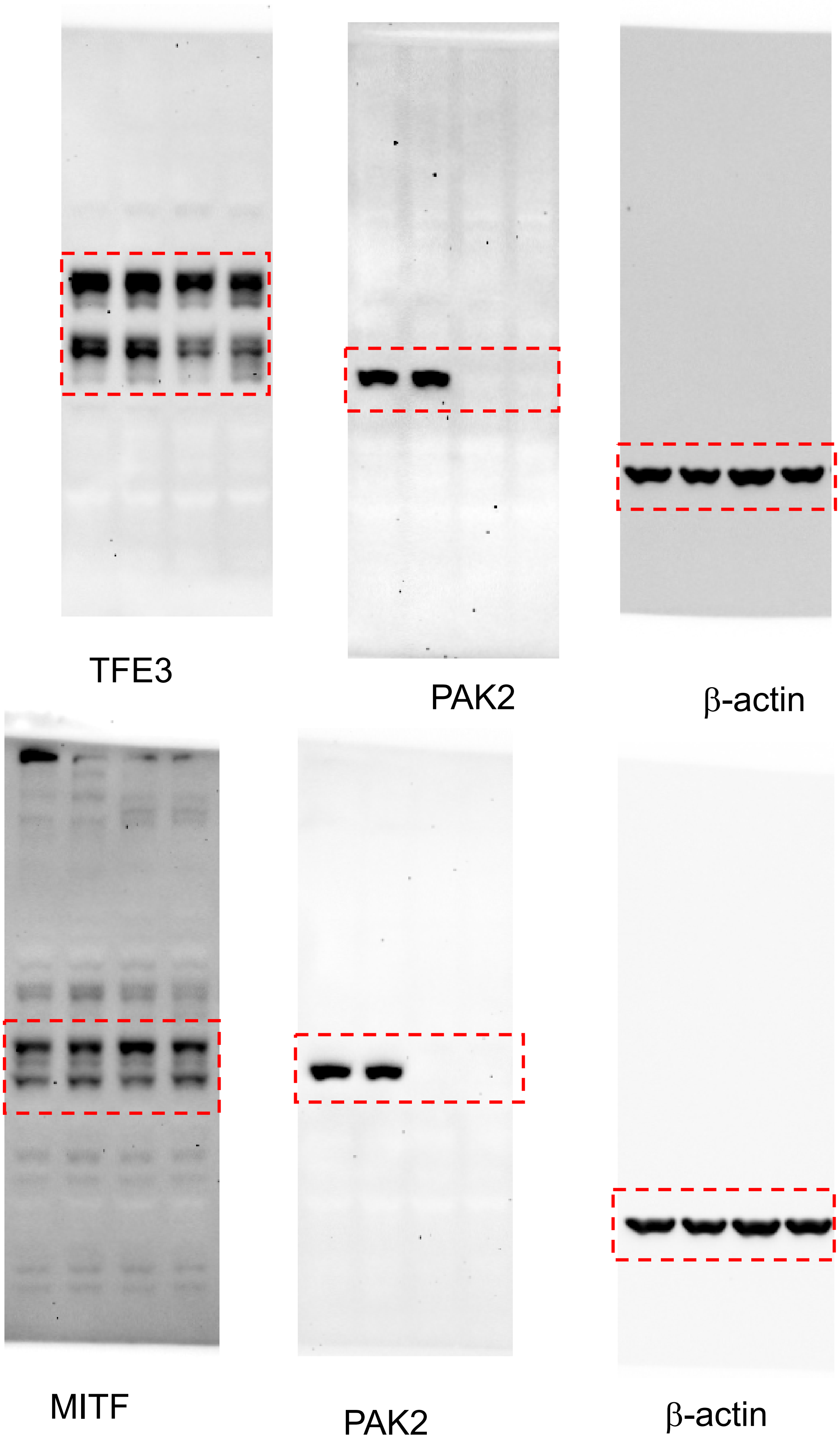

Fig. S12

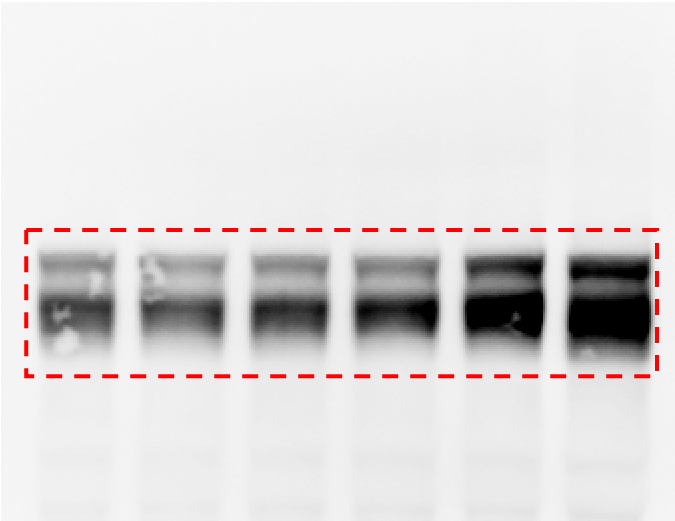

TFEB

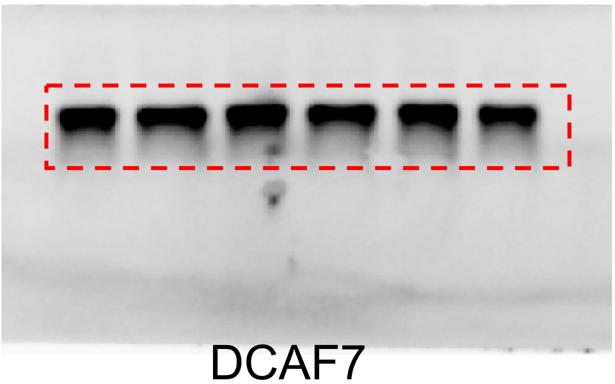

DCAF7

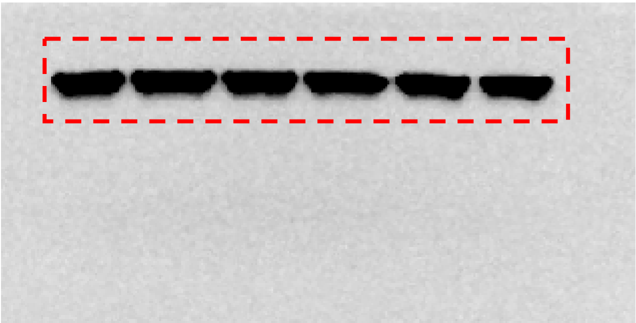

β-actin

Fig S14

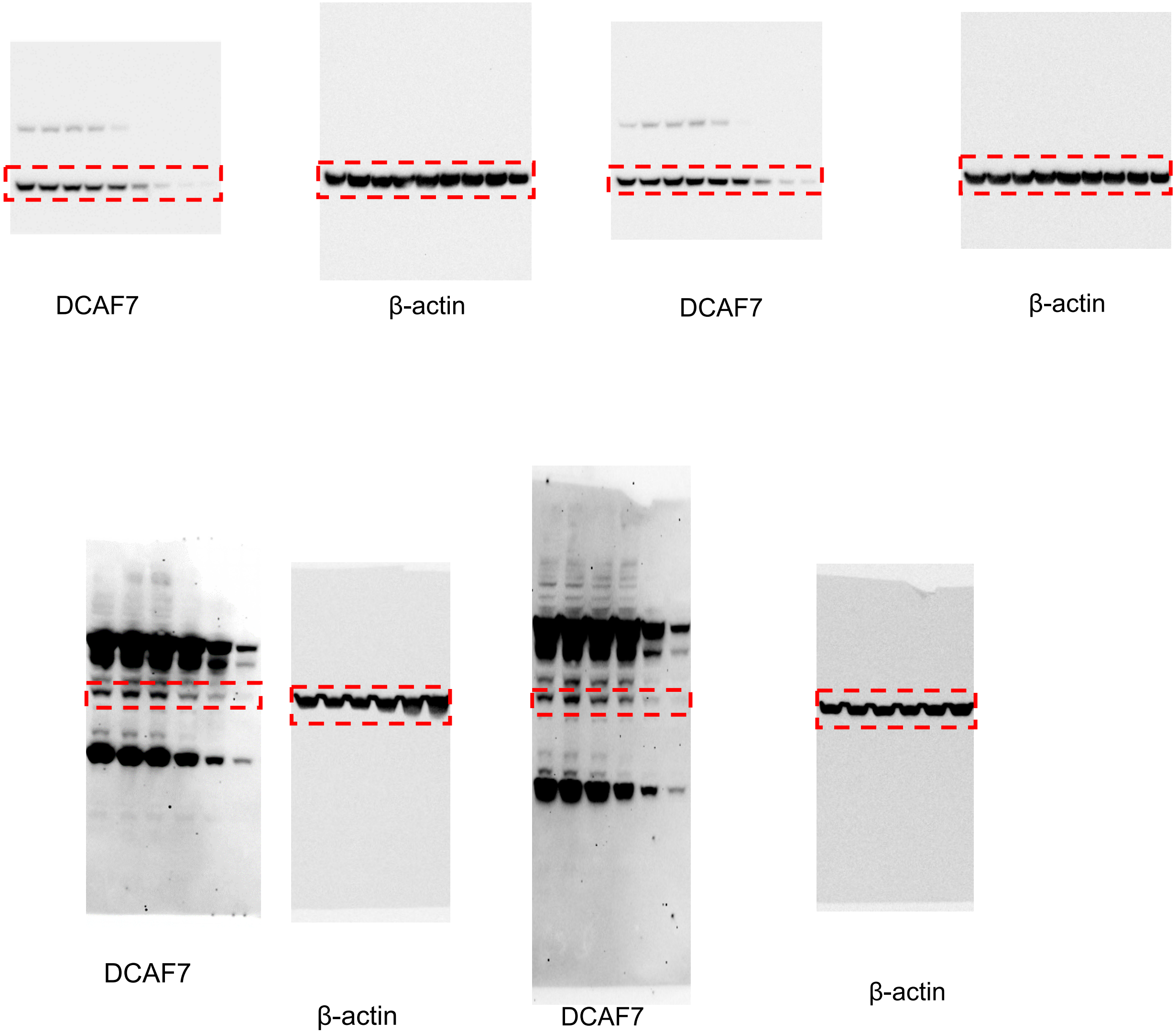

Fig. S14

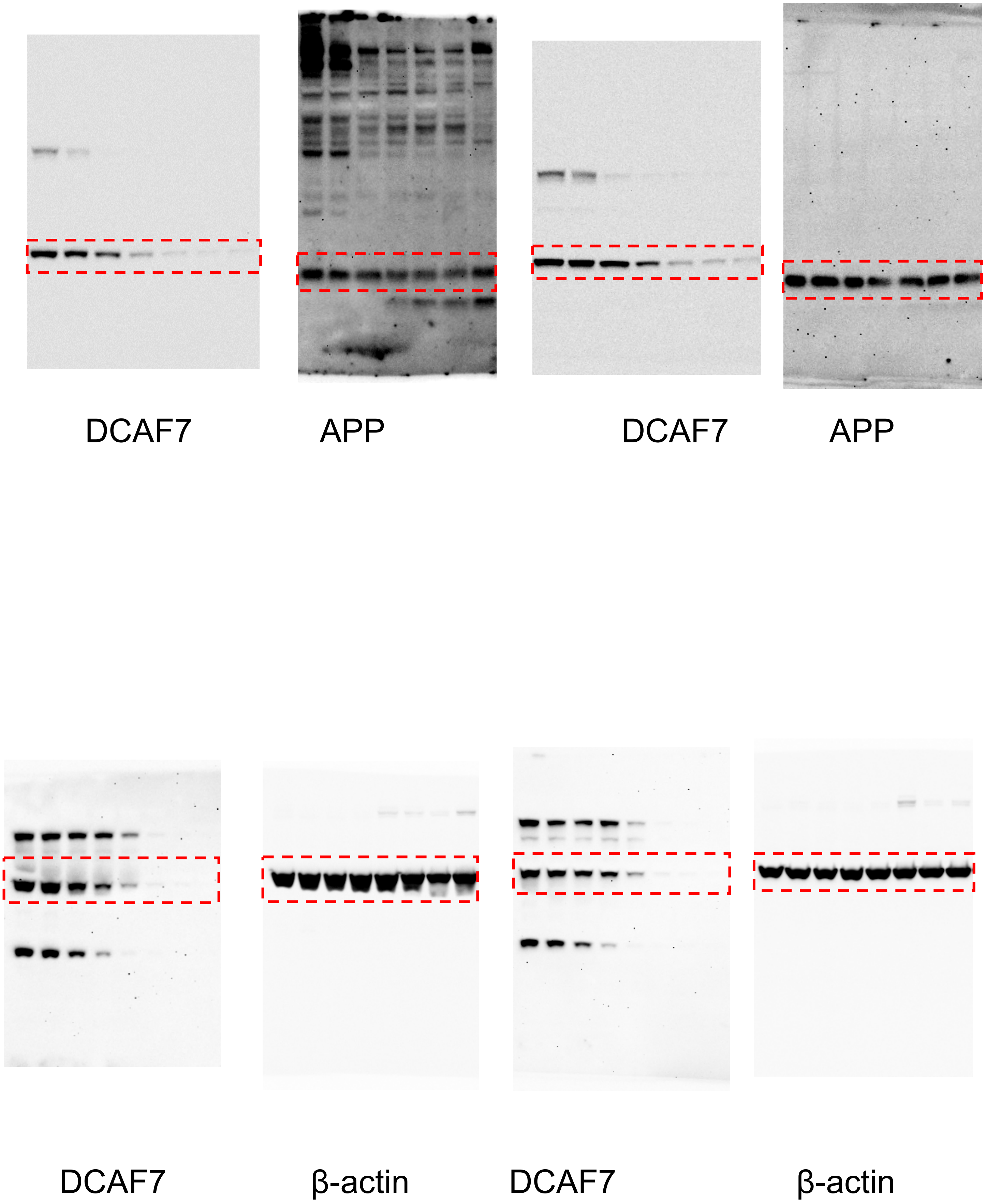

Fig. S14

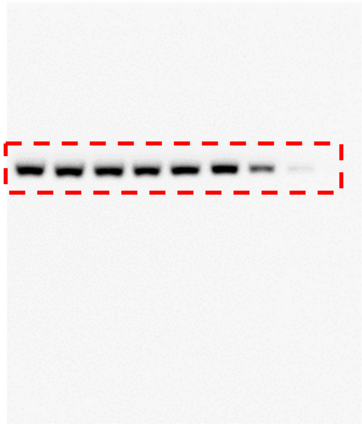

V5 (RBBP4)

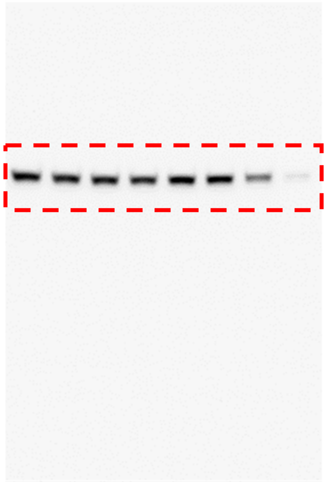

V5 (RBBP4)

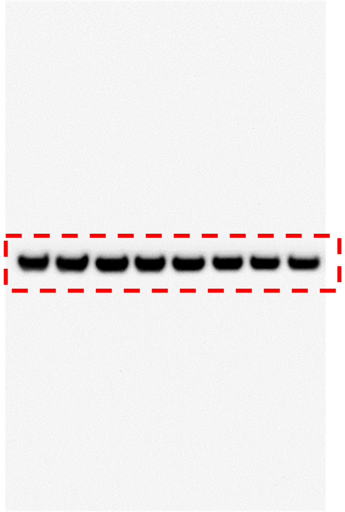

β-actin

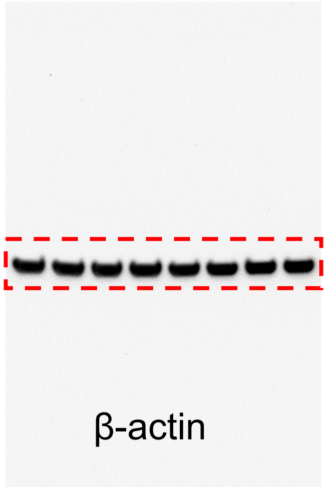

β-actin

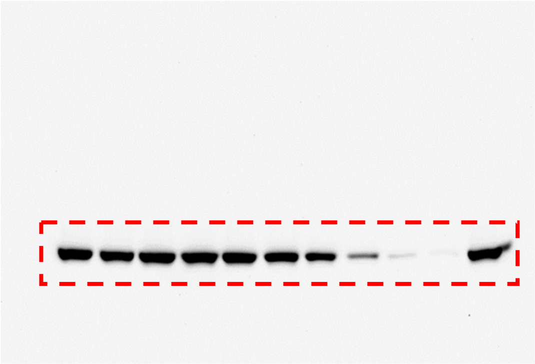

V5 (RBBP4)

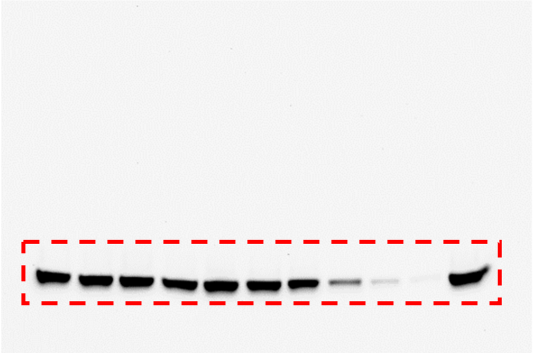

V5 (RBBP4)

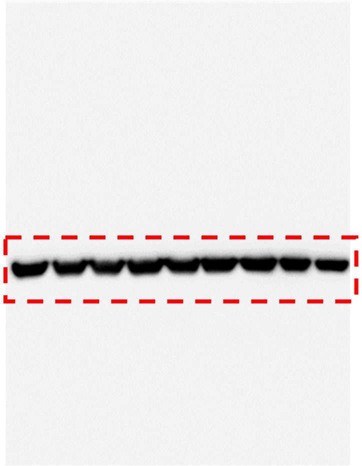

β-actin

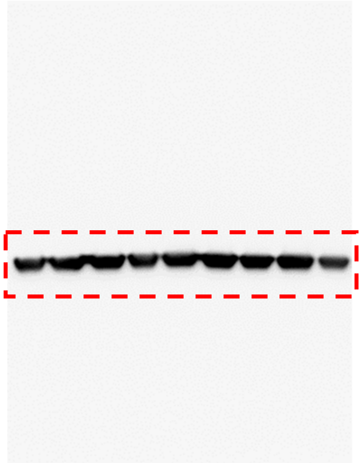

β-actin

Fig. S15

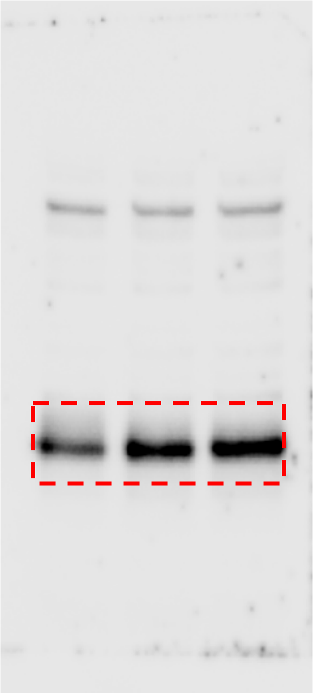

TFEB

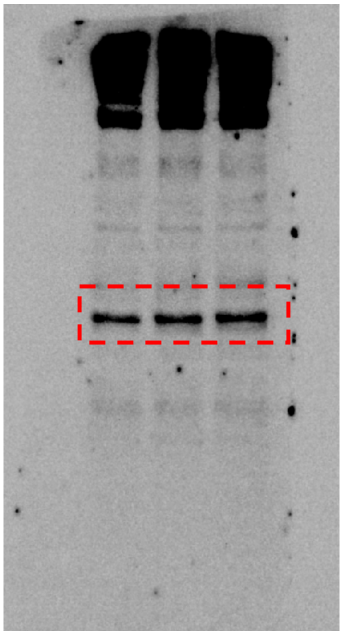

CyclinE

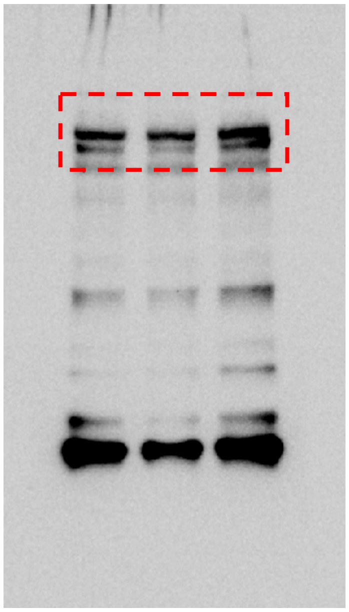

DNMT

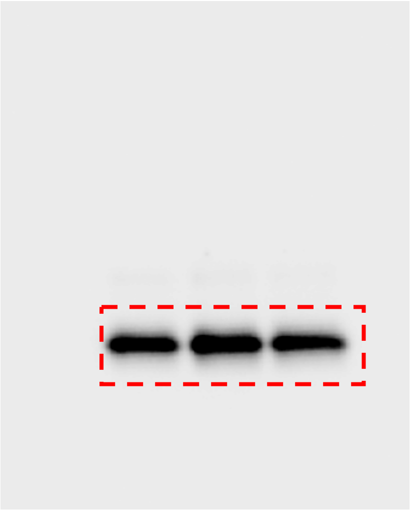

P21

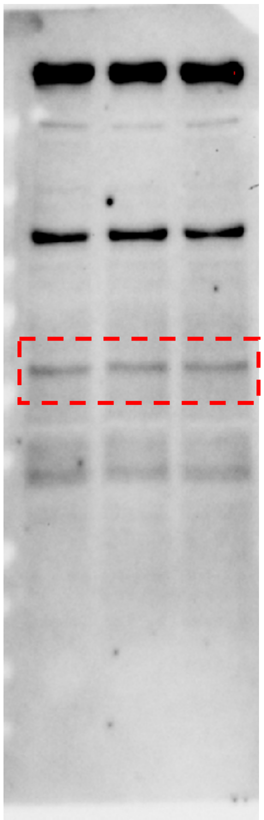

PTEN

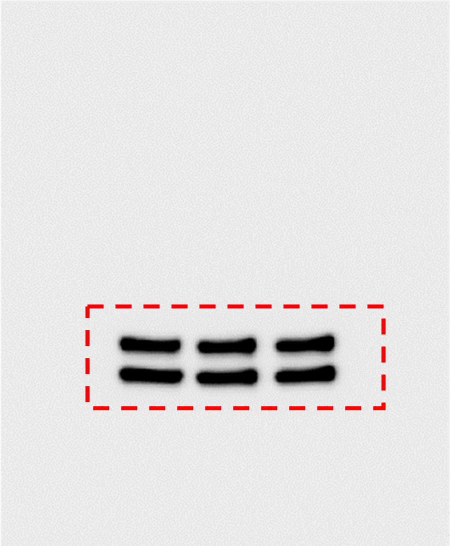

Lamin A/C

Fig. S16

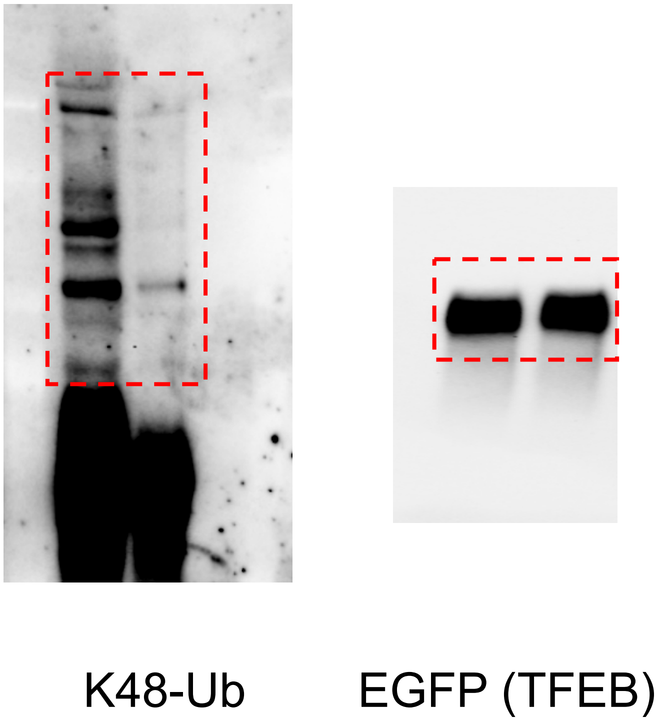

Fig. S23

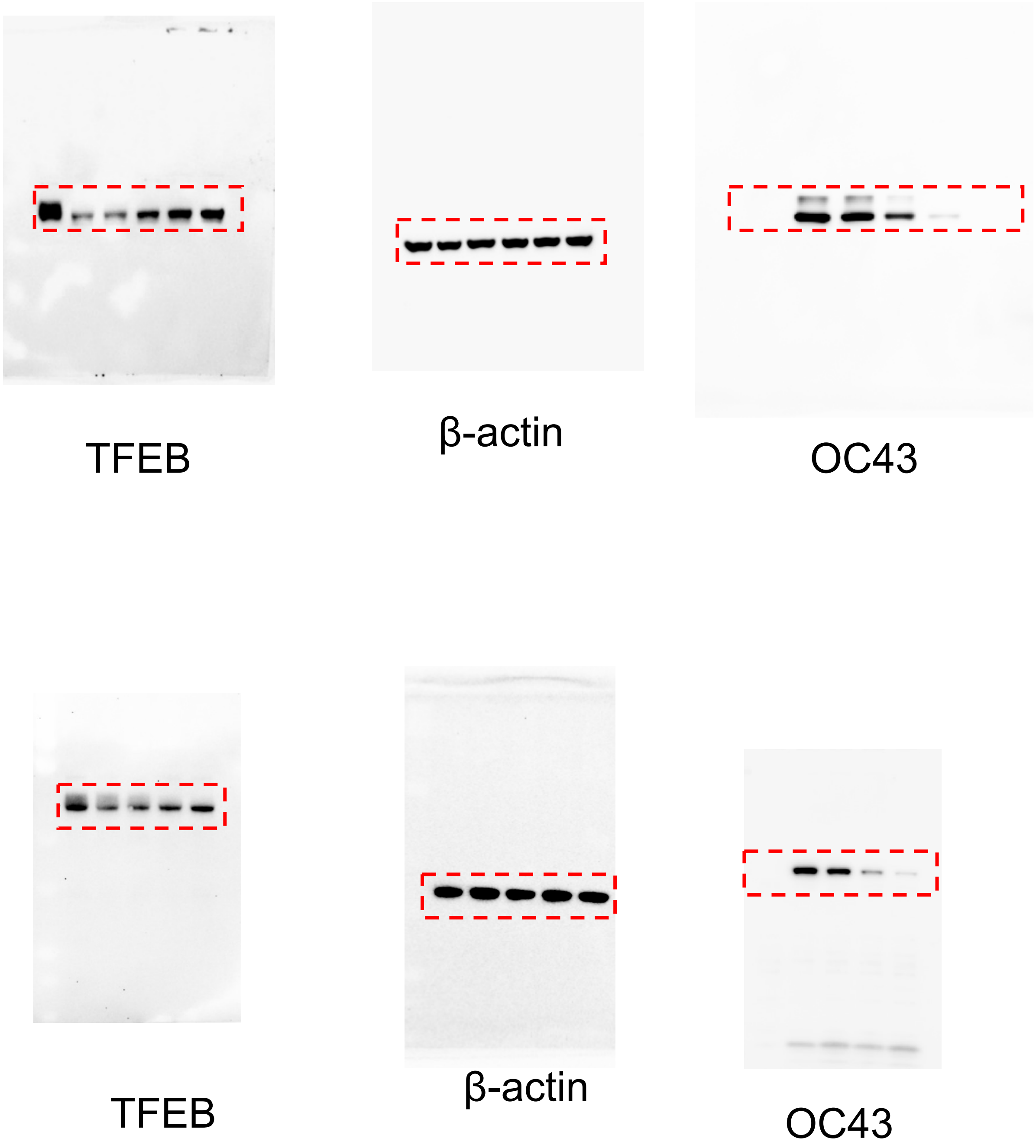

Fig. S24

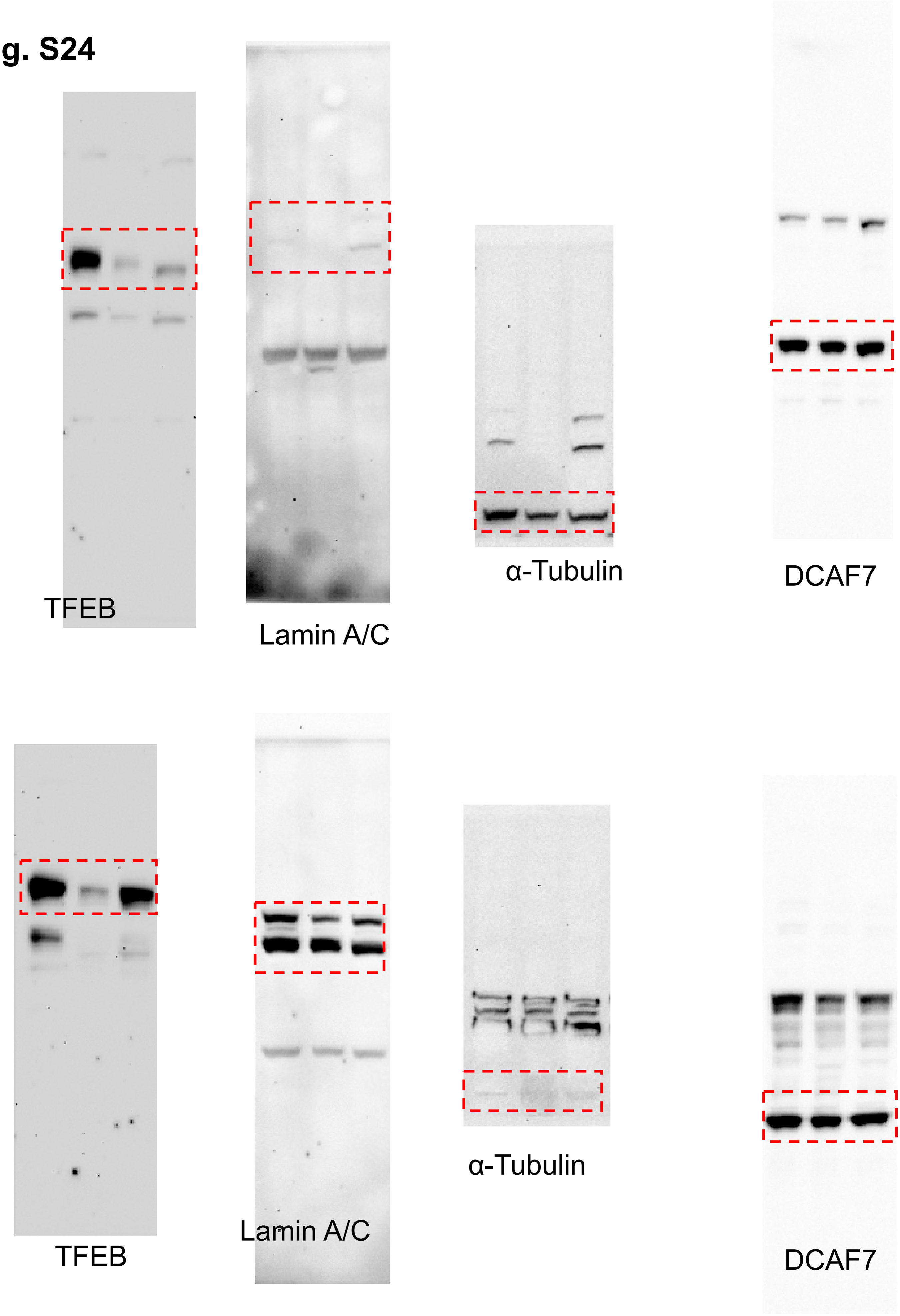

Fig. S24

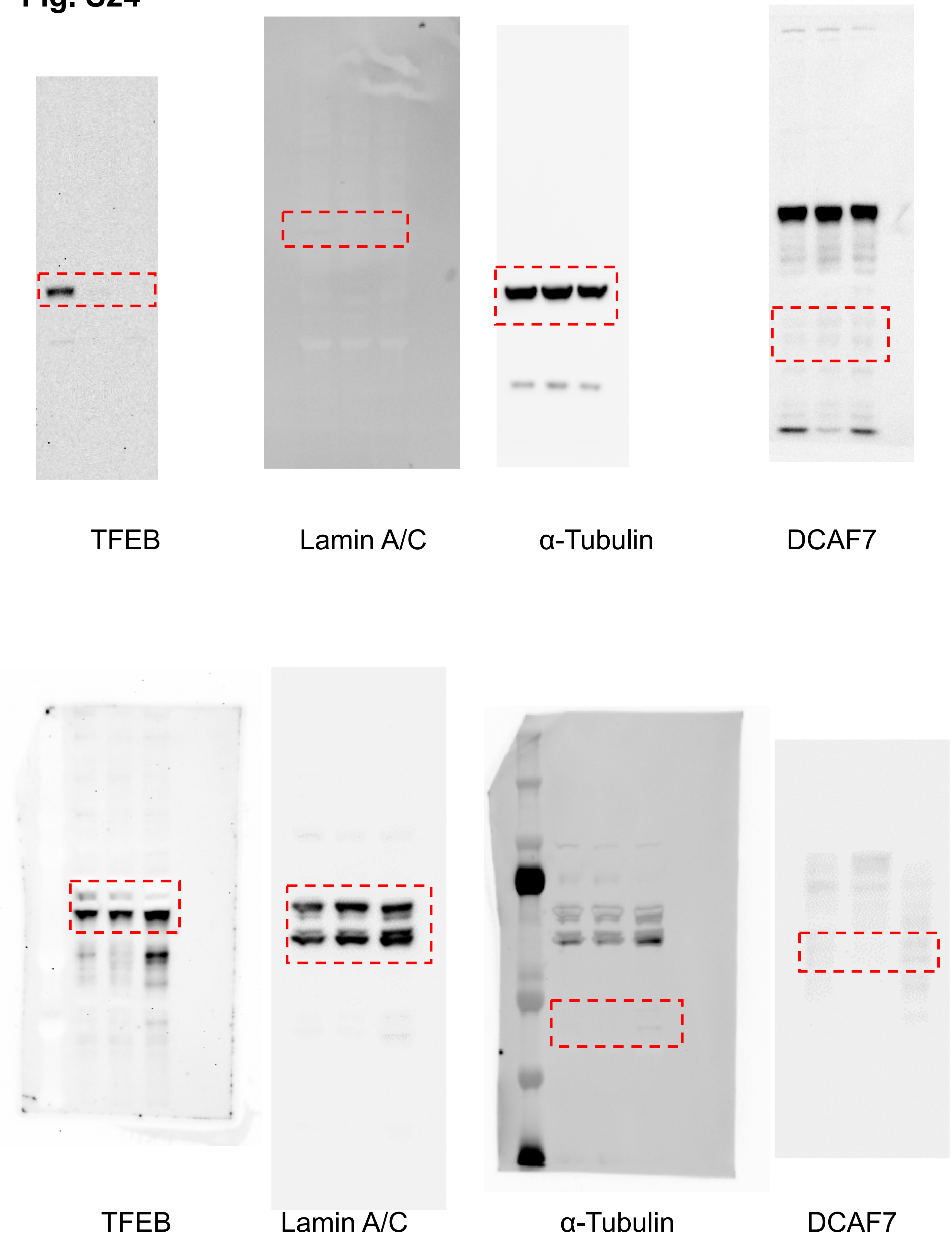

Fig. S28

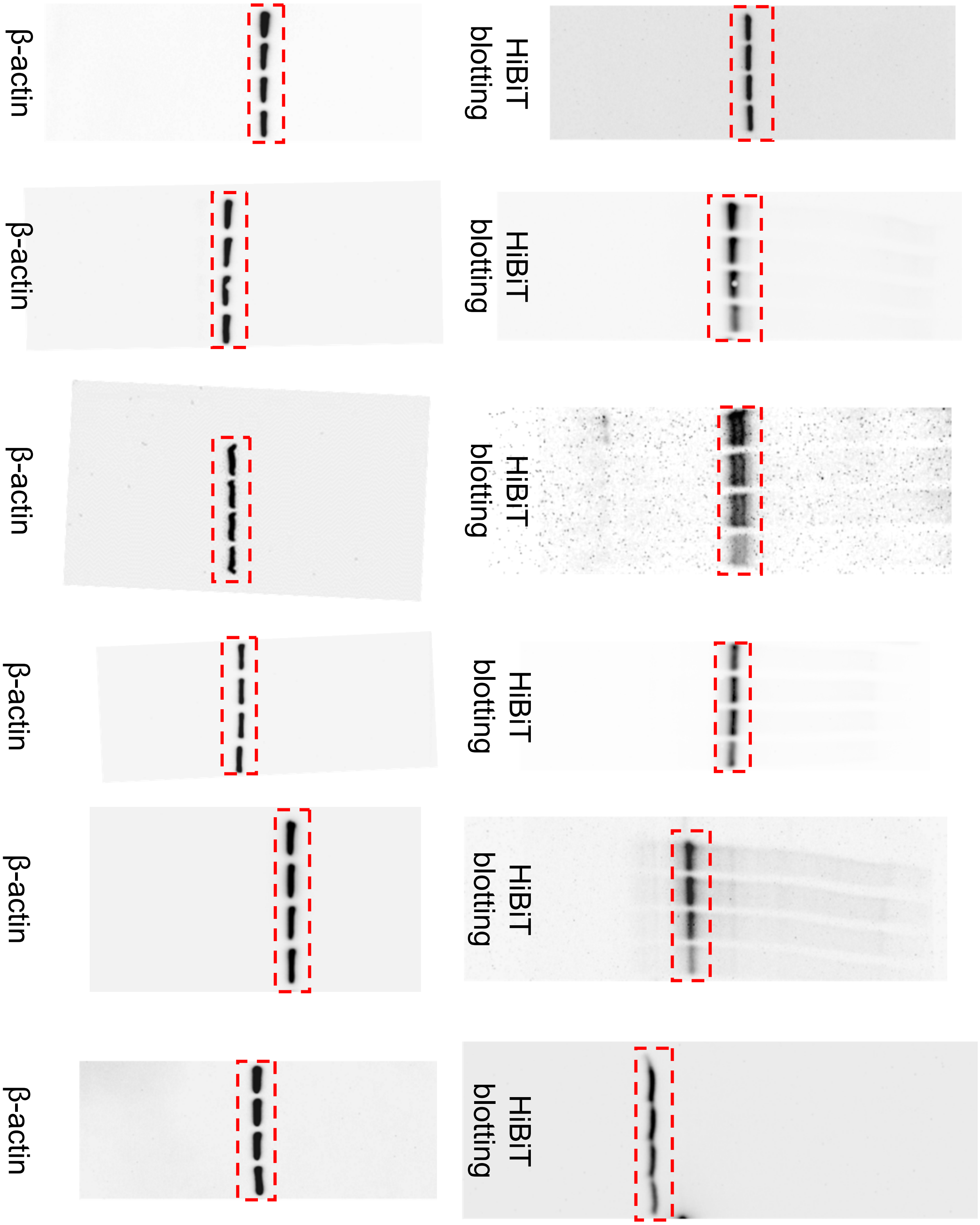
β-actin
HiBiT blotting
β-actin
HiBiT blotting
β-actin
HiBiT blotting
β-actin
HiBiT blotting
β-actin
HiBiT blotting
β-actin
HiBiT blotting

Fig. S28

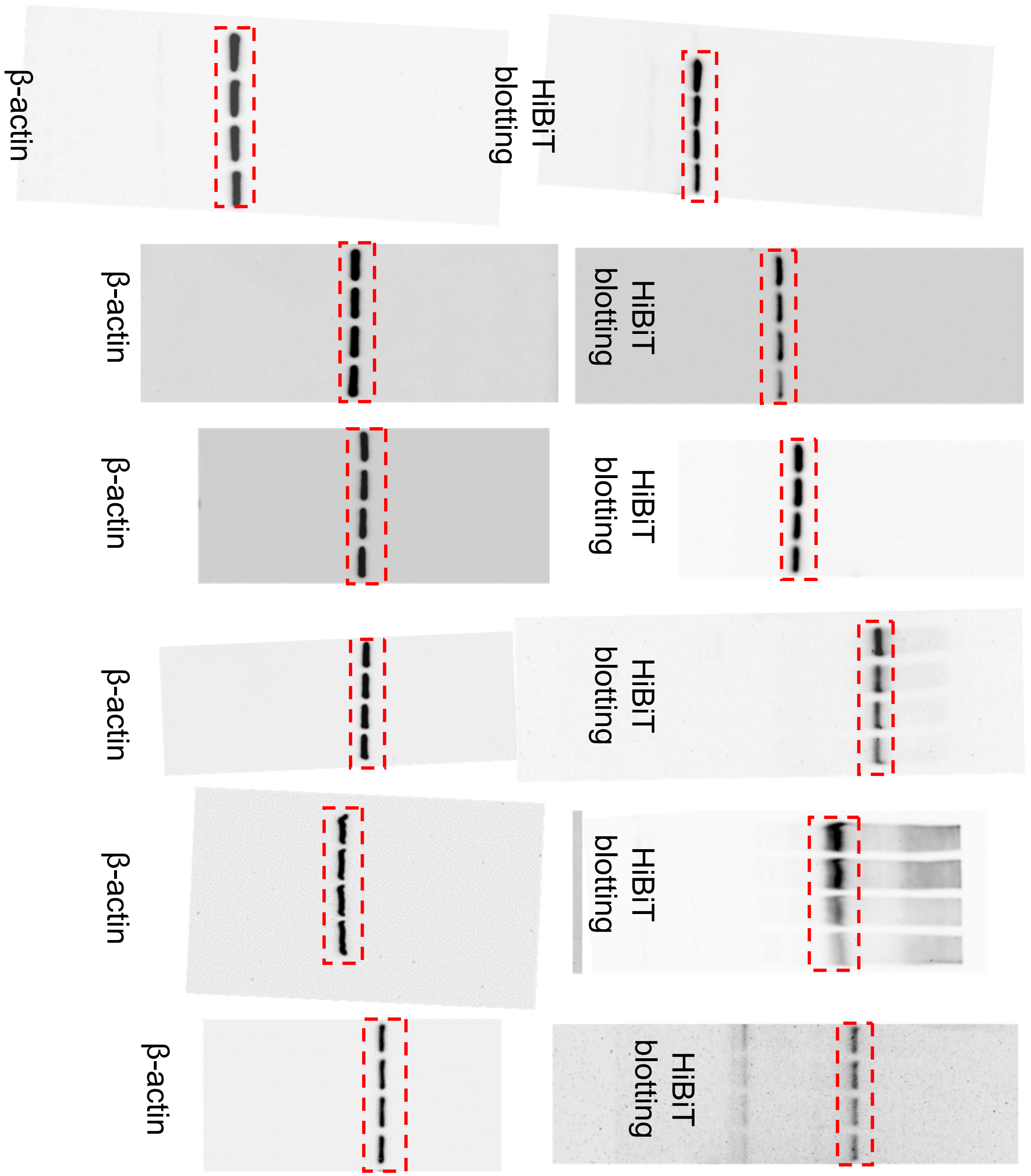
β-actin
HiBiT blotting
β-actin
HiBiT blotting
β-actin
HiBiT blotting
β-actin
HiBiT blotting
β-actin
HiBiT blotting
β-actin
HiBiT blotting

Fig. S28

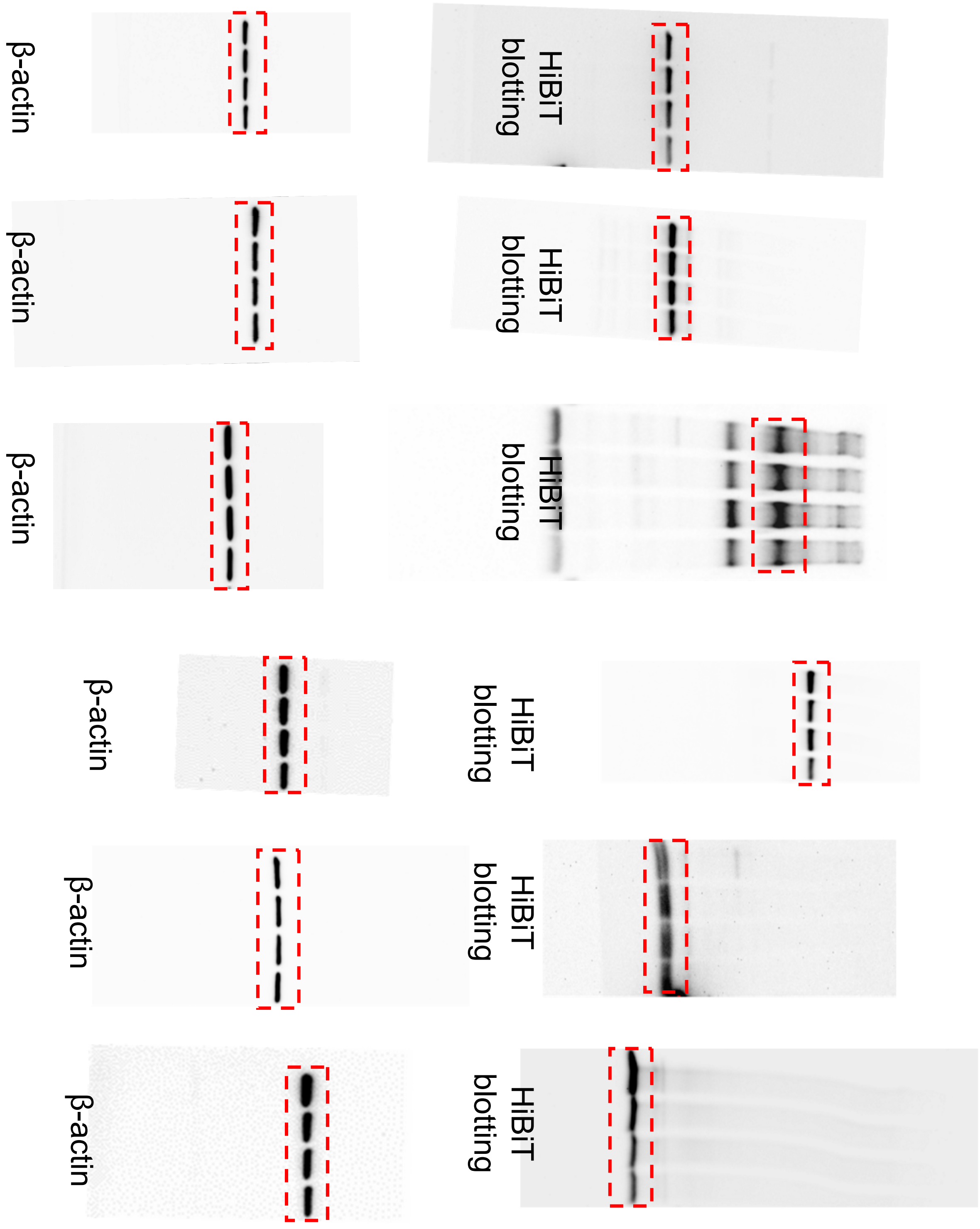
β-actin
HiBiT blotting
β-actin
HiBiT blotting
β-actin
HiBiT blotting
β-actin
HiBiT blotting
β-actin
HiBiT blotting
β-actin
HiBiT blotting

Fig. S28

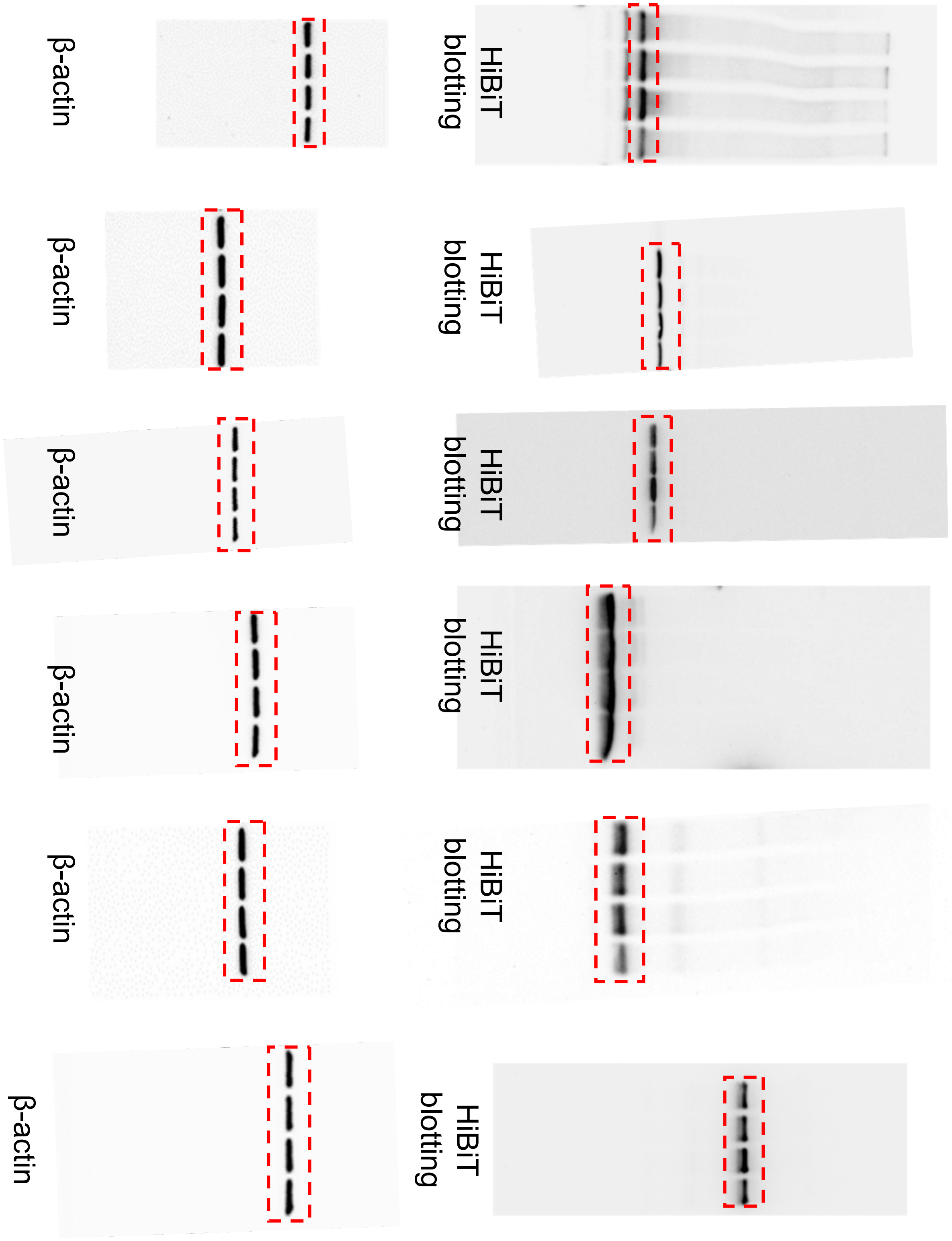
β-actin
HiBiT blotting
β-actin
HiBiT blotting
β-actin
HiBiT blotting
β-actin
HiBiT blotting
β-actin
HiBiT blotting
β-actin
HiBiT blotting

Fig. S28

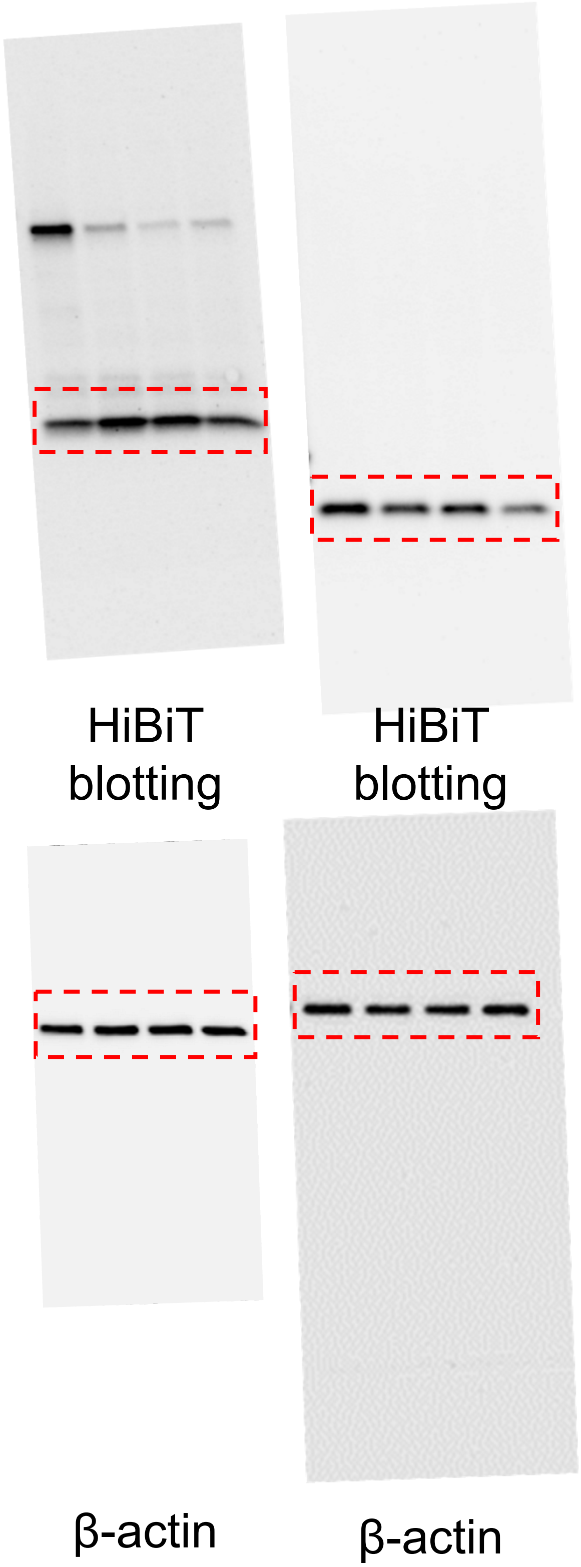

Fig. S29

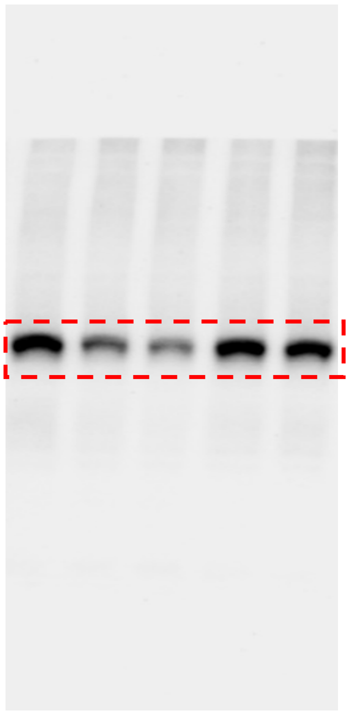

HiBiT

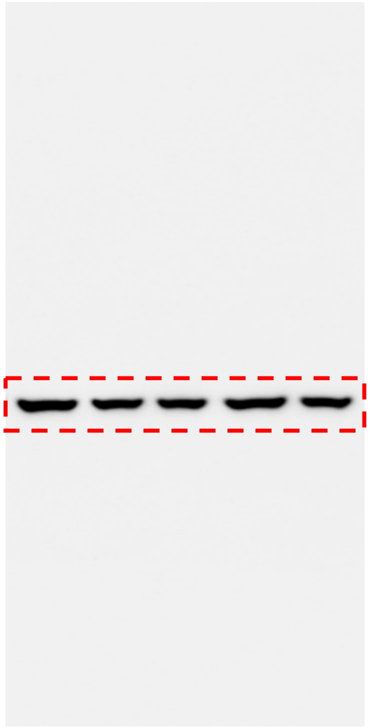

β-actin

REFERENCES AND NOTES

1. World Health Organization, WHO Coronavirus Disease (COVID-19) Dashboard (2024); https://data.who.int/dashboards/covid19/.

2. M. S. Diamond, T. D. Kanneganti, Innate immunity: The first line of defense against SARS-CoV-2. *Nat. Immunol.* **23**, 165–176 (2022).

3. M. Z. Tay, C. M. Poh, L. Rénia, P. A. MacAry, L. F. P. Ng, The trinity of COVID-19: Immunity, inflammation and intervention. *Nat. Rev. Immunol.* **20**, 363–374 (2020).

4. M. Merad, C. A. Blish, F. Sallusto, A. Iwasaki, The immunology and immunopathology of COVID-19. *Science* **375**, 1122–1127 (2022).

5. X. Wang, L. Lu, S. Jiang, SARS-CoV-2 evolution from the BA.2.86 to JN.1 variants: Unexpected consequences. *Trends Immunol.* **45**, 81–84 (2024).

6. A. Telenti, E. B. Hodcroft, D. L. Robertson, The evolution and biology of SARS-CoV-2 variants. *Cold Spring Harb. Perspect. Med.* **12**, a041390 (2022).

7. A. Jayk Bernal, M. M. Gomes da Silva, D. B. Musungaie, E. Kovalchuk, A. Gonzalez, V. Delos Reyes, A. Martín-Quirós, Y. Caraco, A. Williams-Diaz, M. L. Brown, J. Du, A. Pedley, C. Assaid, J. Strizki, J. A. Grobler, H. H. Shamsuddin, R. Tipping, H. Wan, A. Paschke, J. R. Butterton, M. G. Johnson, C. De Anda, MOVe-OUT Study Group, Molnupiravir for oral treatment of Covid-19 in nonhospitalized patients. *N. Engl. J. Med.* **386**, 509–520 (2022).

8. D. R. Owen, C. M. N. Allerton, A. S. Anderson, L. Aschenbrenner, M. Avery, S. Berritt, B. Boras, R. D. Cardin, A. Carlo, K. J. Coffman, A. Dantonio, L. Di, H. Eng, R. A. Ferre, K. S. Gajiwala, S. A. Gibson, S. E. Greasley, B. L. Hurst, E. P. Kadar, A. S. Kalgutkar, J. C. Lee, J. Lee, W. Liu, S. W. Mason, S. Noell, J. J. Novak, R. S. Obach, K. Ogilvie, N. C. Patel, M. Pettersson, D. K. Rai, M. R. Reese, M. F. Sammons, J. G. Sathish, R. S. P. Singh, C. M. Steppan, A. E. Stewart, J. B. Tuttle, L. Updyke, P. R. Verhoest, L. Wei, Q. Yang, Y. Zhu, An oral SARS-CoV-2 M^{pro} inhibitor clinical candidate for the treatment of COVID-19. *Science* **374**, 1586–1593 (2021).

9. A. von Delft, M. D. Hall, A. D. Kwong, L. A. Purcell, K. S. Saikatendu, U. Schmitz, J. A. Tallarico, A. A. Lee, Accelerating antiviral drug discovery: Lessons from COVID-19. *Nat. Rev. Drug Discov.* **22**, 585–603 (2023).

10. Y. Hu, E. M. Lewandowski, H. Tan, X. Zhang, R. T. Morgan, X. Zhang, L. M. C. Jacobs, S. G. Butler, M. V. Gongora, J. Choy, X. Deng, Y. Chen, J. Wang, Naturally occurring mutations of SARS-CoV-2 main protease confer drug resistance to nirmatrelvir. *ACS Cent. Sci.* **9**, 1658–1669 (2023).

11. J. J. Arnold, S. D. Sharma, J. Y. Feng, A. S. Ray, E. D. Smidansky, M. L. Kireeva, A. Cho, J. Perry, J. E. Vela, Y. Park, Y. Xu, Y. Tian, D. Babusis, O. Barauskus, B. R. Peterson, A. Gnatt, M. Kashlev, W. Zhong, C. E. Cameron, Sensitivity of mitochondrial transcription and resistance of RNA polymerase II dependent nuclear transcription to antiviral ribonucleosides. *PLOS Pathog.* **8**, e1003030 (2012).

12. C. T. R. Vegivinti, K. W. Evanson, H. Lyons, I. Akosman, A. Barrett, N. Hardy, B. Kane, P. R. Keesari, Y. S. Pulakurthi, E. Sheffels, P. Balasubramanian, R. Chibbar, S. Chittajallu, K. Cowie, J. Karon, L. Siegel, R. Tarchand, C. Zinn, N. Gupta, K. M. Kallmes, K. Saravu, J. Touchette, Efficacy of antiviral therapies for COVID-19: A systematic review of randomized controlled trials. *BMC Infect. Dis.* **22**, 107 (2022).

13. N. Chaudhary, D. Weissman, K. A. Whitehead, mRNA vaccines for infectious diseases: Principles, delivery and clinical translation. *Nat. Rev. Drug Discov.* **20**, 817–838 (2021).

14. S. H. E. Kaufmann, A. Dorhoi, R. S. Hotchkiss, R. Bartenschlager, Host-directed therapies for bacterial and viral infections. *Nat. Rev. Drug Discov.* **17**, 35–56 (2018).

15. J. Baggen, E. Vanstreels, S. Jansen, D. Daelemans, Cellular host factors for SARS-CoV-2 infection. *Nat. Microbiol.* **6**, 1219–1232 (2021).

16. S. Ghosh, T. A. Dellibovi-Ragheb, A. Kerviel, E. Pak, Q. Qiu, M. Fisher, P. M. Takvorian, C. Bleck, V. W. Hsu, A. R. Fehr, S. Perlman, S. R. Achar, M. R. Straus, G. R. Whittaker, C. A. M. de Haan, J. Kehrl, G. Altan-Bonnet, N. Altan-Bonnet, β-Coronaviruses use lysosomes for egress instead of the biosynthetic secretory pathway. *Cell* **183**, 1520–1535.e14 (2020).

17. Y. Choi, J. W. Bowman, J. U. Jung, Autophagy during viral infection—A double-edged sword. *Nat. Rev. Microbiol.* **16**, 341–354 (2018).

18. V. Sharma, S. Verma, E. Seranova, S. Sarkar, D. Kumar, Selective autophagy and xenophagy in infection and disease. *Front. Cell Dev. Biol.* **6**, 147 (2018).

19. M. Sardiello, M. Palmieri, A. di Ronza, D. L. Medina, M. Valenza, V. A. Gennarino, C. Di Malta, F. Donaudy, V. Embrione, R. S. Polishchuk, S. Banfi, G. Parenti, E. Cattaneo, A. Ballabio, A gene network regulating lysosomal biogenesis and function. *Science* **325**, 473–477 (2009).

20. C. Settembre, C. Di Malta, V. A. Polito, M. Garcia Arencibia, F. Vetrini, S. Erdin, S. U. Erdin, T. Huynh, D. Medina, P. Colella, M. Sardiello, D. C. Rubinsztein, A. Ballabio, TFEB links autophagy to lysosomal biogenesis. *Science* **332**, 1429–1433 (2011).

21. J. A. Mindell, Lysosomal acidification mechanisms. *Annu. Rev. Physiol.* **74**, 69–86 (2012).

22. C. Settembre, R. Zoncu, D. L. Medina, F. Vetrini, S. Erdin, S. Erdin, T. Huynh, M. Ferron, G. Karsenty, M. C. Vellard, V. Facchinetti, D. M. Sabatini, A. Ballabio, A lysosome-to-nucleus signalling mechanism senses and regulates the lysosome via mTOR and TFEB. *EMBO J.* **31**, 1095–1108 (2012).

23. G. Napolitano, A. Ballabio, TFEB at a glance. *J. Cell Sci.* **129**, 2475–2481 (2016).

24. O. Visvikis, N. Ihuegbu, S. A. Labed, L. G. Luhachack, A.-M. F. Alves, A. C. Wollenberg, L. M. Stuart, G. D. Stormo, J. E. Irazoqui, Innate host defense requires TFEB-mediated transcription of cytoprotective and antimicrobial genes. *Immunity* **40**, 896–909 (2014).

25. A. Choy, J. Dancourt, B. Mugo, T. J. O'Connor, R. R. Isberg, T. J. Melia, C. R. Roy, The *Legionella* effector RavZ inhibits host autophagy through irreversible Atg8 deconjugation. *Science* **338**, 1072–1076 (2012).

26. S. Köster, S. Upadhyay, P. Chandra, K. Papavinasasundaram, G. Yang, A. Hassan, S. J. Grigsby, E. Mittal, H. S. Park, V. Jones, F. F. Hsu, M. Jackson, C. M. Sassetti, J. A. Philips, Mycobacterium tuberculosis is protected from NADPH oxidase and LC3-associated

phagocytosis by the LCP protein CpsA. *Proc. Natl. Acad. Sci. U.S.A.* **114**, E8711–E8720 (2017).

27. Y. Xu, P. Zhou, S. Cheng, Q. Lu, K. Nowak, A.-K. Hopp, L. Li, X. Shi, Z. Zhou, W. Gao, D. Li, H. He, X. Liu, J. Ding, M. O. Hottiger, F. Shao, A bacterial effector reveals the V-ATPase-ATG16L1 axis that initiates xenophagy. *Cell* **178**, 552–566.e20 (2019).

28. M. Takla, S. Keshri, D. C. Rubinsztein, The post-translational regulation of transcription factor EB (TFEB) in health and disease. *EMBO Rep.* **24**, e57574 (2023).

29. J. A. Martina, Y. Chen, M. Gucek, R. Puertollano, MTORC1 functions as a transcriptional regulator of autophagy by preventing nuclear transport of TFEB. *Autophagy* **8**, 903–914 (2012).

30. A. Roczniak-Ferguson, C. S. Petit, F. Froehlich, S. Qian, J. Ky, B. Angarola, T. C. Walther, S. M. Ferguson, The transcription factor TFEB links mTORC1 signaling to transcriptional control of lysosome homeostasis. *Sci. Signal.* **5**, ra42-ra42 (2012).

31. G. Napolitano, A. Esposito, H. Choi, M. Matarese, V. Benedetti, C. Di Malta, J. Monfregola, D. L. Medina, J. Lippincott-Schwartz, A. Ballabio, mTOR-dependent phosphorylation controls TFEB nuclear export. *Nat. Commun.* **9**, 3312 (2018).

32. S. Vega-Rubin-de-Celis, S. Peña-Llopis, M. Konda, J. Brugarolas, Multistep regulation of TFEB by MTORC1. *Autophagy* **13**, 464–472 (2017).

33. D. L. Medina, S. Di Paola, I. Peluso, A. Armani, D. De Stefani, R. Venditti, S. Montefusco, A. Scotto-Rosato, C. Prezioso, A. Forrester, C. Settembre, W. Wang, Q. Gao, H. Xu, M. Sandri, R. Rizzuto, M. A. De Matteis, A. Ballabio, Lysosomal calcium signalling regulates autophagy through calcineurin and TFEB. *Nat. Cell Biol.* **17**, 288–299 (2015).

34. T. Fujita, S. Kubo, T. Shioda, A. Tokumura, S. Minami, M. Tsuchiya, Y. Isaka, H. Ogawa, M. Hamasaki, L. Yu, T. Yoshimori, S. Nakamura, THOC4 regulates energy homeostasis by stabilizing *TFEB* mRNA during prolonged starvation. *J. Cell Sci.* **134**, jcs248203 (2021).

35. Z. Zhang, Q. Qian, M. Li, F. Shao, W. X. Ding, V. A. Lira, S. X. Chen, S. C. Sebag, G. S. Hotamisligil, H. Cao, L. Yang, The unfolded protein response regulates hepatic autophagy by sXBP1-mediated activation of TFEB. *Autophagy* **17**, 1841–1855 (2021).

36. Y. Sha, L. Rao, C. Settembre, A. Ballabio, N. T. Eissa, STUB1 regulates TFEB-induced autophagy-lysosome pathway. *EMBO J.* **36**, 2544–2552 (2017).

37. M. Ferron, C. Settembre, J. Shimazu, J. Lacombe, S. Kato, D. J. Rawlings, A. Ballabio, G. Karsenty, A RANKL-PKCβ-TFEB signaling cascade is necessary for lysosomal biogenesis in osteoclasts. *Genes Dev.* **27**, 955–969 (2013).

38. T. Li, L. Yin, X. Kang, W. Xue, N. Wang, J. Zhang, P. Yuan, L. Lin, Y. Li, TFEB acetylation promotes lysosome biogenesis and ameliorates Alzheimer's disease-relevant phenotypes in mice. *J. Biol. Chem.* **298**, 102649 (2022).

39. A. Hershko, A. Ciechanover, The ubiquitin system. *Annu. Rev. Biochem.* **67**, 425–479 (1998).

40. J. R. Skaar, J. K. Pagan, M. Pagano, Mechanisms and function of substrate recruitment by F-box proteins. *Nat. Rev. Mol. Cell Biol.* **14**, 369–381 (2013).

41. H.-C. S. Yen, Q. Xu, D. M. Chou, Z. Zhao, S. J. Elledge, Global protein stability profiling in mammalian cells. *Science* **322**, 918–923 (2008).

42. F. Lampert, M. M. Brodersen, M. Peter, Guard the guardian: A CRL4 ligase stands watch over histone production. *Nucleus* **8**, 134–143 (2017).

43. S. Jackson, Y. Xiong, CRL4s: The CUL4-RING E3 ubiquitin ligases. *Trends Biochem. Sci.* **34**, 562–570 (2009).

44. B. Fabre, I. Livneh, T. Ziv, A. Ciechanover, Modulation of the cell cycle regulating transcription factor E2F1 pathway by the proteasome following amino acid starvation. *Biochem. Biophys. Res. Commun.* **513**, 721–725 (2019).

45. I. Dikic, Proteasomal and autophagic degradation systems. *Annu. Rev. Biochem.* **86**, 193–224 (2017).

46. L. Baird, M. Yamamoto, The molecular mechanisms regulating the KEAP1-NRF2 pathway. *Mol. Cell. Biol.* **40**, e00099-20 (2020).

47. G. L. Kirkpatrick, The common cold. *Prim. Care* **23**, 657–675 (1996).

48. F. Glenewinkel, M. J. Cohen, C. R. King, S. Kaspar, S. Bamberg-Lemper, J. S. Mymryk, W. Becker, The adaptor protein DCAF7 mediates the interaction of the adenovirus E1A oncoprotein with the protein kinases DYRK1A and HIPK2. *Sci. Rep.* **6**, 28241 (2016).

49. E. Alvarado, M. Yousefelahiyeh, G. Alvarado, R. Shang, T. Whitman, A. Martinez, Y. Yu, A. Pham, A. Bhandari, B. Wang, R. M. Nissen, Wdr68 mediates dorsal and ventral patterning events for craniofacial development. *PLOS ONE* **11**, e0166984 (2016).

50. J. Xiang, S. Yang, N. Xin, M. A. Gaertig, R. H. Reeves, S. Li, X. J. Li, DYRK1A regulates Hap1-Dcaf7/WDR68 binding with implication for delayed growth in down syndrome. *Proc. Natl. Acad. Sci. U.S.A.* **114**, E1224–E1233 (2017).

51. C. Nardone, B. A. Palanski, D. C. Scott, R. T. Timms, K. W. Barber, X. Gu, A. Mao, Y. Leng, E. V. Watson, B. A. Schulman, P. A. Cole, S. J. Elledge, A central role for regulated protein stability in the control of TFE3 and MITF by nutrients. *Mol. Cell* **83**, 57–73.e9 (2023).

52. W. Qiu, C. Xu, X. Xiao, D. Xu, Computational prediction of ubiquitination proteins using evolutionary profiles and functional domain annotation. *Curr. Genomics* **20**, 389–399 (2019).

53. B. B. Chen, T. A. Coon, J. R. Glasser, B. J. McVerry, J. Zhao, Y. Zhao, C. Zou, B. Ellis, F. C. Sciurba, Y. Zhang, R. K. Mallampalli, A combinatorial F box protein directed pathway controls TRAF adaptor stability to regulate inflammation. *Nat. Immunol.* **14**, 470–479 (2013).

54. T. Mori, S. Gotoh, M. Shirakawa, T. Hakoshima, Structural basis of DDB1-and-Cullin 4-associated Factor 1 (DCAF1) recognition by merlin/NF2 and its implication in tumorigenesis by CD44-mediated inhibition of merlin suppression of DCAF1 function. *Genes Cells* **19**, 603–619 (2014).

55. C. Zhang, B. Chen, K. Jiang, L. Lao, H. Shen, Z. Chen, Activation of TNF-α/NF-κB axis enhances $CRL4B^{DCAF11}$ E3 ligase activity and regulates cell cycle progression in human osteosarcoma cells. *Mol. Oncol.* **12**, 476–494 (2018).

56. H. Robertson, J. D. Hayes, C. Sutherland, A partnership with the proteasome; the destructive nature of GSK3. *Biochem. Pharmacol.* **147**, 77–92 (2018).

57. L. Li, H. J. Friedrichsen, S. Andrews, S. Picaud, L. Volpon, K. Ngeow, G. Berridge, R. Fischer, K. L. B. Borden, P. Filippakopoulos, C. R. Goding, A TFEB nuclear export signal integrates amino acid supply and glucose availability. *Nat. Commun.* **9**, 2685 (2018).

58. J. Rudolph, L. J. Murray, C. O. Ndubaku, T. O'Brien, E. Blackwood, W. Wang, I. Aliagas, L. Gazzard, J. J. Crawford, J. Drobnick, W. Lee, X. Zhao, K. P. Hoeflich, D. A. Favor, P. Dong, H. Zhang, C. E. Heise, A. Oh, C. C. Ong, H. La, P. Chakravarty, C. Chan, D. Jakubiak, J. Epler, S. Ramaswamy, R. Vega, G. Cain, D. Diaz, Y. Zhong, Chemically diverse group I p21-activated kinase (PAK) inhibitors impart acute cardiovascular toxicity with a narrow therapeutic window. *J. Med. Chem.* **59**, 5520–5541 (2016).

59. Y. Su, P. Song, H. Wang, B. Hu, J. Wang, M. S. Cheng, Precise design of highly isoform-selective p21-activated kinase 4 inhibitors: Computational insights into the selectivity mechanism through molecular dynamics simulation and binding free energy calculation. *J. Biomol. Struct. Dyn.* **38**, 3825–3837 (2020).

60. T. A. Coon, A. C. McKelvey, T. Lear, S. Rajbhandari, S. R. Dunn, W. Connelly, J. Y. Zhao, S. Han, Y. Liu, N. M. Weathington, B. J. McVerry, Y. Zhang, B. B. Chen, The proinflammatory role of HECTD2 in innate immunity and experimental lung injury. *Sci. Transl. Med.* **7**, 295ra109 (2015).

61. T. Lear, A. C. McKelvey, S. Rajbhandari, S. R. Dunn, T. A. Coon, W. Connelly, J. Y. Zhao, D. J. Kass, Y. Zhang, Y. Liu, B. B. Chen, Ubiquitin E3 ligase FIEL1 regulates fibrotic lung injury through SUMO-E3 ligase PIAS4. *J. Exp. Med.* **213**, 1029–1046 (2016).

62. A. J. Nowak, C. Alfieri, C. U. Stirnimann, V. Rybin, F. Baudin, N. Ly-Hartig, D. Lindner, C. W. Müller, Chromatin-modifying complex component Nurf55/p55 associates with histones

H3 and H4 and polycomb repressive complex 2 subunit Su(z)12 through partially overlapping binding sites. *J. Biol. Chem.* **286**, 23388–23396 (2011).

63. S. Orlicky, X. Tang, V. Neduva, N. Elowe, E. D. Brown, F. Sicheri, M. Tyers, An allosteric inhibitor of substrate recognition by the SCF(Cdc4) ubiquitin ligase. *Nat. Biotechnol.* **28**, 733–737 (2010).

64. N. Pastore, O. A. Brady, H. I. Diab, J. A. Martina, L. Sun, T. Huynh, J. A. Lim, H. Zare, N. Raben, A. Ballabio, R. Puertollano, TFEB and TFE3 cooperate in the regulation of the innate immune response in activated macrophages. *Autophagy* **12**, 1240–1258 (2016).

65. D. E. Gordon, G. M. Jang, M. Bouhaddou, J. Xu, K. Obernier, K. M. White, M. J. O'Meara, V. V. Rezelj, J. Z. Guo, D. L. Swaney, T. A. Tummino, R. Hüttenhain, R. M. Kaake, A. L. Richards, B. Tutuncuoglu, H. Foussard, J. Batra, K. Haas, M. Modak, M. Kim, P. Haas, B. J. Polacco, H. Braberg, J. M. Fabius, M. Eckhardt, M. Soucheray, M. J. Bennett, M. Cakir, M. J. McGregor, Q. Li, B. Meyer, F. Roesch, T. Vallet, A. M. Kain, L. Miorin, E. Moreno, Z. Z. C. Naing, Y. Zhou, S. Peng, Y. Shi, Z. Zhang, W. Shen, I. T. Kirby, J. E. Melnyk, J. S. Chorba, K. Lou, S. A. Dai, I. Barrio-Hernandez, D. Memon, C. Hernandez-Armenta, J. Lyu, C. J. P. Mathy, T. Perica, K. B. Pilla, S. J. Ganesan, D. J. Saltzberg, R. Rakesh, X. Liu, S. B. Rosenthal, L. Calviello, S. Venkataramanan, J. Liboy-Lugo, Y. Lin, X.-P. Huang, Y. F. Liu, S. A. Wankowicz, M. Bohn, M. Safari, F. S. Ugur, C. Koh, N. S. Savar, Q. D. Tran, D. Shengjuler, S. J. Fletcher, M. C. O'Neal, Y. Cai, J. C. J. Chang, D. J. Broadhurst, S. Klippsten, P. P. Sharp, N. A. Wenzell, D. Kuzuoglu-Ozturk, H.-Y. Wang, R. Trenker, J. M. Young, D. A. Cavero, J. Hiatt, T. L. Roth, U. Rathore, A. Subramanian, J. Noack, M. Hubert, R. M. Stroud, A. D. Frankel, O. S. Rosenberg, K. A. Verba, D. A. Agard, M. Ott, M. Emerman, N. Jura, M. von Zastrow, E. Verdin, A. Ashworth, O. Schwartz, C. d'Enfert, S. Mukherjee, M. Jacobson, H. S. Malik, D. G. Fujimori, T. Ideker, C. S. Craik, S. N. Floor, J. S. Fraser, J. D. Gross, A. Sali, B. L. Roth, D. Ruggero, J. Taunton, T. Kortemme, P. Beltrao, M. Vignuzzi, A. García-Sastre, K. M. Shokat, B. K. Shoichet, N. J. Krogan, A SARS-CoV-2 protein interaction map reveals targets for drug repurposing. *Nature* **583**, 459–468 (2020).

66. M. A. White, W. Lin, X. Cheng, Discovery of COVID-19 inhibitors targeting the SARS-CoV-2 Nsp13 helicase. *J. Phys. Chem. Lett.* **11**, 9144–9151 (2020).

67. L. Yan, Y. Zhang, J. Ge, L. Zheng, Y. Gao, T. Wang, Z. Jia, H. Wang, Y. Huang, M. Li, Q. Wang, Z. Rao, Z. Lou, Architecture of a SARS-CoV-2 mini replication and transcription complex. *Nat. Commun.* **11**, 5874 (2020).

68. C. S. Chen, G. Bach, R. E. Pagano, Abnormal transport along the lysosomal pathway in mucolipidosis, type IV disease. *Proc. Natl. Acad. Sci. U.S.A.* **95**, 6373–6378 (1998).

69. M. Koval, R. E. Pagano, Sorting of an internalized plasma membrane lipid between recycling and degradative pathways in normal and Niemann-Pick, type A fibroblasts. *J. Cell Biol.* **111**, 429–442 (1990).

70. N. Sun, J. Yun, J. Liu, D. Malide, C. Liu, I. I. Rovira, K. M. Holmström, M. M. Fergusson, Y. H. Yoo, C. A. Combs, T. Finkel, Measuring in vivo mitophagy. *Mol. Cell* **60**, 685–696 (2015).

71. A. Banerjee, S. Kulkarni, A. Mukherjee, Herpes simplex virus: The hostile guest that takes over your home. *Front. Microbiol.* **11**, 733 (2020).

72. J. Mao, E. Lin, L. He, J. Yu, P. Tan, Y. Zhou, Autophagy and viral infection. *Adv. Exp. Med. Biol.* **1209**, 55–78 (2019).

73. R. M. Hekman, A. J. Hume, R. K. Goel, K. M. Abo, J. Huang, B. C. Blum, R. B. Werder, E. L. Suder, I. Paul, S. Phanse, A. Youssef, K. D. Alysandratos, D. Padhorny, S. Ojha, A. Mora-Martin, D. Kretov, P. E. A. Ash, M. Verma, J. Zhao, J. J. Patten, C. Villacorta-Martin, D. Bolzan, C. Perea-Resa, E. Bullitt, A. Hinds, A. Tilston-Lunel, X. Varelas, S. Farhangmehr, U. Braunschweig, J. H. Kwan, M. McComb, A. Basu, M. Saeed, V. Perissi, E. J. Burks, M. D. Layne, J. H. Connor, R. Davey, J.-X. Cheng, B. L. Wolozin, B. J. Blencowe, S. Wuchty, S. M. Lyons, D. Kozakov, D. Cifuentes, M. Blower, D. N. Kotton, A. A. Wilson, E. Mühlberger, A. Emili, Actionable cytopathogenic host responses of human alveolar type 2 cells to SARS-CoV-2. *Mol. Cell* **80**, 1104–1122.e9 (2020).

74. C. Van den Broeke, M. Radu, J. Chernoff, H. W. Favoreel, An emerging role for p21-activated kinases (Paks) in viral infections. *Trends Cell Biol.* **20**, 160–169 (2010).

75. L. Bajaj, P. Lotfi, R. Pal, A. D. Ronza, J. Sharma, M. Sardiello, Lysosome biogenesis in health and disease. *J. Neurochem.* **148**, 573–589 (2019).

76. J. E. Irazoqui, Key roles of MiT transcription factors in innate immunity and inflammation. *Trends Immunol.* **41**, 157–171 (2020).

77. J. X. Song, J. Liu, Y. Jiang, Z. Y. Wang, M. Li, Transcription factor EB: An emerging drug target for neurodegenerative disorders. *Drug Discov. Today* **26**, 164–172 (2021).

78. P. V. Markov, M. Ghafari, M. Beer, K. Lythgoe, P. Simmonds, N. I. Stilianakis, A. Katzourakis, The evolution of SARS-CoV-2. *Nat. Rev. Microbiol.* **21**, 361–379 (2023).

79. G. Miao, H. Zhao, Y. Li, M. Ji, Y. Chen, Y. Shi, Y. Bi, P. Wang, H. Zhang, ORF3a of the COVID-19 virus SARS-CoV-2 blocks HOPS complex-mediated assembly of the SNARE complex required for autolysosome formation. *Dev. Cell* **56**, 427–442.e5 (2021).

80. P. S. Contreras, P. J. Tapia, E. Jeong, S. Ghosh, N. Altan-Bonnet, R. Puertollano, Beta-coronaviruses exploit cellular stress responses by modulating TFEB and TFE3 activity. *iScience* **26**, 106169 (2023).

81. C. S. Shi, N. R. Nabar, N. N. Huang, J. H. Kehrl, SARS-coronavirus open reading frame-8b triggers intracellular stress pathways and activates NLRP3 inflammasomes. *Cell Death Discov.* **5**, 101 (2019).

82. R. Wang, C. R. Simoneau, J. Kulsuptrakul, M. Bouhaddou, K. A. Travisano, J. M. Hayashi, J. Carlson-Stevermer, J. R. Zengel, C. M. Richards, P. Fozouni, J. Oki, L. Rodriguez, B. Joehnk, K. Walcott, K. Holden, A. Sil, J. E. Carette, N. J. Krogan, M. Ott, A. S. Puschnik, Genetic screens identify host factors for SARS-CoV-2 and common cold coronaviruses. *Cell* **184**, 106–119.e14 (2021).

83. J. Wei, M. M. Alfajaro, P. C. DeWeirdt, R. E. Hanna, W. J. Lu-Culligan, W. L. Cai, M. S. Strine, S. M. Zhang, V. R. Graziano, C. O. Schmitz, J. S. Chen, M. C. Mankowski, R. B. Filler, N. G. Ravindra, V. Gasque, F. J. de Miguel, A. Patil, H. Chen, K. Y. Oguntuyo, L. Abriola, Y. V. Surovtseva, R. C. Orchard, B. Lee, B. D. Lindenbach, K. Politi, D. van Dijk,

C. Kadoch, M. D. Simon, Q. Yan, J. G. Doench, C. B. Wilen, Genome-wide CRISPR screens reveal host factors critical for SARS-CoV-2 infection. *Cell* **184**, 76–91.e13 (2021).

84. A. W. Bernheimer, L. L. Schwartz, Lysosomal disruption by bacterial toxins. *J. Bacteriol.* **87**, 1100–1104 (1964).

85. K. A. Wani, D. Goswamy, S. Taubert, R. Ratnappan, A. Ghazi, J. E. Irazoqui, NHR-49/PPAR-α and HLH-30/TFEB cooperate for *C. elegans* host defense via a flavin-containing monooxygenase. *Elife* **10**, e62775 (2021).

86. H. K. Kim, S. Min, M. Song, S. Jung, J. W. Choi, Y. Kim, S. Lee, S. Yoon, H. H. Kim, Deep learning improves prediction of CRISPR-Cpf1 guide RNA activity. *Nat. Biotechnol.* **36**, 239–241 (2018).

87. N. E. Sanjana, O. Shalem, F. Zhang, Improved vectors and genome-wide libraries for CRISPR screening. *Nat. Methods* **11**, 783–784 (2014).

88. E. Zielinska, D. Liu, H. Y. Wu, J. Quiroz, R. Rappaport, D. P. Yang, Development of an improved microneutralization assay for respiratory syncytial virus by automated plaque counting using imaging analysis. *Virol. J.* **2**, 84 (2005).

89. J. Bowes, A. J. Brown, J. Hamon, W. Jarolimek, A. Sridhar, G. Waldron, S. Whitebread, Reducing safety-related drug attrition: The use of in vitro pharmacological profiling. *Nat. Rev. Drug Discov.* **11**, 909–922 (2012).

90. K. Kadimisetty, K. J. Sheets, P. H. Gross, M. J. Zerr, D. Ouazia, Tandem Ubiquitin Binding Entities (TUBEs) as tools to explore ubiquitin-proteasome system and PROTAC drug discovery. *Methods Mol. Biol.* **2365**, 185–202 (2021).

91. C. McQuin, A. Goodman, V. Chernyshev, L. Kamentsky, B. A. Cimini, K. W. Karhohs, M. Doan, L. Ding, S. M. Rafelski, D. Thirstrup, W. Wiegraebe, S. Singh, T. Becker, J. C. Caicedo, A. E. Carpenter, CellProfiler 3.0: Next-generation image processing for biology. *PLOS Biol.* **16**, e2005970 (2018).

92. J. Schindelin, I. Arganda-Carreras, E. Frise, V. Kaynig, M. Longair, T. Pietzsch, S. Preibisch, C. Rueden, S. Saalfeld, B. Schmid, J. Y. Tinevez, D. J. White, V. Hartenstein, K. Eliceiri, P. Tomancak, A. Cardona, Fiji: An open-source platform for biological-image analysis. *Nat. Methods* **9**, 676–682 (2012).

93. R. Jafari, H. Almqvist, H. Axelsson, M. Ignatushchenko, T. Lundbäck, P. Nordlund, D. Martinez Molina, The cellular thermal shift assay for evaluating drug target interactions in cells. *Nat. Protoc.* **9**, 2100–2122 (2014).

94. T. B. Lear, A. C. McKelvey, J. W. Evankovich, S. Rajbhandari, T. A. Coon, S. R. Dunn, J. D. Londino, B. J. McVerry, Y. Zhang, E. Valenzi, C. L. Burton, R. Gordon, S. Gingras, K. C. Lockwood, M. J. Jurczak, R. Lafyatis, M. J. Shlomchik, Y. Liu, B. B. Chen, KIAA0317 regulates pulmonary inflammation through SOCS2 degradation. *JCI Insight* **4**, e129110 (2019).

95. E. Kinoshita, E. Kinoshita-Kikuta, K. Takiyama, T. Koike, Phosphate-binding tag, a new tool to visualize phosphorylated proteins. *Mol. Cell. Proteomics* **5**, 749–757 (2006).

96. A. Real-Hohn, M. Groznica, N. Löffler, D. Blaas, H. Kowalski, nanoDSF: In vitro label-free method to monitor picornavirus Uncoating and test compounds affecting particle stability. *Front. Microbiol.* **11**, 1442 (2020).

97. Y. Chen, J. W. Evankovich, T. B. Lear, F. Tuncer, J. R. Kennerdell, D. P. Camarco, M. S. Shishido, Y. Liu, B. B. Chen, A small molecule NRF2 activator BC-1901S ameliorates inflammation through DCAF1/NRF2 axis. *Redox Biol.* **32**, 101485 (2020).

98. K. M. Riching, S. Mahan, C. R. Corona, M. McDougall, J. D. Vasta, M. B. Robers, M. Urh, D. L. Daniels, Quantitative live-cell kinetic degradation and mechanistic profiling of PROTAC mode of action. *ACS Chem. Biol.* **13**, 2758–2770 (2018).

99. C. Chen, J. W. Saville, M. M. Marti, A. Schäfer, M. H. Cheng, D. Mannar, X. Zhu, A. M. Berezuk, A. Banerjee, M. D. Sobolewski, A. Kim, B. R. Treat, P. M. Da Silva Castanha, N. Enick, K. D. McCormick, X. Liu, C. Adams, M. G. Hines, Z. Sun, W. Chen, J. L. Jacobs, S. M. Barratt-Boyes, J. W. Mellors, R. S. Baric, I. Bahar, D. S. Dimitrov, S. Subramaniam, D.

R. Martinez, W. Li, Potent and broad neutralization of SARS-CoV-2 variants of concern (VOCs) including omicron sub-lineages BA.1 and BA.2 by biparatopic human VH domains. *iScience* **25**, 104798 (2022).

100. A. R. Crowe, W. Yue, Semi-quantitative determination of protein expression using immunohistochemistry staining and analysis: An integrated protocol. *Bio Protoc.* **9**, e3465 (2019).

101. B. N. Walter, Z. Huang, R. Jakobi, P. T. Tuazon, E. S. Alnemri, G. Litwack, J. A. Traugh, Cleavage and activation of p21-activated protein kinase γ-PAK by CPP32 (caspase 3). Effects of autophosphorylation on activity. *J. Biol. Chem.* **273**, 28733–28739 (1998).

102. A. Subramanian, P. Tamayo, V. K. Mootha, S. Mukherjee, B. L. Ebert, M. A. Gillette, A. Paulovich, S. L. Pomeroy, T. R. Golub, E. S. Lander, J. P. Mesirov, Gene set enrichment analysis: A knowledge-based approach for interpreting genome-wide expression profiles. *Proc. Natl. Acad. Sci. U.S.A.* **102**, 15545–15550 (2005).

103. N. Pastore, T. Huynh, N. J. Herz, A. Calcagni', T. J. Klisch, L. Brunetti, K. H. Kim, M. De Giorgi, A. Hurley, A. Carissimo, M. Mutarelli, N. Aleksieva, L. D'Orsi, W. R. Lagor, D. D. Moore, C. Settembre, M. J. Finegold, S. J. Forbes, A. Ballabio, TFEB regulates murine liver cell fate during development and regeneration. *Nat. Commun.* **11**, 2461 (2020).

104. K. L. Carey, G. L. C. Paulus, L. Wang, D. R. Balce, J. W. Luo, P. Bergman, I. C. Ferder, L. Kong, N. Renaud, S. Singh, M. Kost-Alimova, B. Nyfeler, K. G. Lassen, H. W. Virgin, R. J. Xavier, TFEB transcriptional responses reveal negative feedback by BHLHE40 and BHLHE41. *Cell Rep.* **33**, 108371 (2020).

105. Y. Chen, T. B. Lear, J. W. Evankovich, M. B. Larsen, B. Lin, I. Alfaras, J. R. Kennerdell, L. Salminen, D. P. Camarco, K. C. Lockwood, F. Tuncer, J. Liu, M. M. Myerburg, J. F. McDyer, Y. Liu, T. Finkel, B. B. Chen, A high-throughput screen for TMPRSS2 expression identifies FDA-approved compounds that can limit SARS-CoV-2 entry. *Nat. Commun.* **12**, 3907 (2021).

106. J. M. Falcón-Pérez, R. Nazarian, C. Sabatti, E. C. Dell'Angelica, Distribution and dynamics of Lamp1-containing endocytic organelles in fibroblasts deficient in BLOC-3. *J. Cell Sci.* **118**, 5243–5255 (2005).

107. D.-K. Kim, J. J. Knapp, D. Kuang, A. Chawla, P. Cassonnet, H. Lee, D. Sheykhkarimli, P. Samavarchi-Tehrani, H. Abdouni, A. Rayhan, R. Li, O. Pogoutse, É. Coyaud, S. van der Werf, C. Demeret, A.-C. Gingras, M. Taipale, B. Raught, Y. Jacob, F. P. Roth, A comprehensive, flexible collection of SARS-CoV-2 coding regions. *G3 (Bethesda)* **10**, 3399–3402 (2020).
